# Cesarean section induced dysbiosis promotes type 2 immunity but not oxazolone-induced dermatitis in mice

**DOI:** 10.1080/19490976.2023.2271151

**Published:** 2023-10-27

**Authors:** Line Fisker Zachariassen, Maria Bernadette Bergh Ebert, Caroline Märta Junker Mentzel, Ling Deng, Lukasz Krych, Dennis Sandris Nielsen, Jakob Stokholm, Camilla Hartmann Friis Hansen

**Affiliations:** a Department of Veterinary and Animal Sciences, Faculty of Health and Medical Sciences, University of Copenhagen, Frederiksberg, Denmark; b Department of Food Science, Faculty of Science, University of Copenhagen, Frederiksberg, Denmark; c COPSAC, Copenhagen Prospective Studies on Asthma in Childhood, Herlev and Gentofte Hospital, Gentofte, Denmark

**Keywords:** Animal model, atopic dermatitis, birth mode, bacteroides, cesarean section, gut microbiota, IgE, type 2 immunity

## Abstract

Delivery by cesarean section (CS) is associated with an altered gut microbiota (GM) colonization and a higher risk of later chronic inflammatory diseases. Studies investigating the association between CS and atopic dermatitis (AD) are contradictive and often biased by confounding factors. The aim of this study was therefore to provide experimental evidence for the association between CS and AD in a mouse model and clarify the role of the GM changes associated with CS. It was hypothesized that CS-delivered mice, and human CS-GM transplanted mice develop severe dermatitis due to early dysbiosis. BALB/c mice delivered by CS or vaginally (VD) as well as BALB/c mice transplanted with GM from CS or VD human donors were challenged with oxazolone on the ear. The severity of dermatitis was evaluated by ear thickness and clinical and histopathological assessment which were similar between all groups. The immune response was assessed by serum IgE concentration, local cytokine response, and presence of immune cells in the draining lymph node. Both CS-delivered mice and mice inoculated with human CS-GM had a higher IgE concentration. A higher proportion of Th2 cells were also found in the CS-GM inoculated mice, but no differences were seen in the cytokine levels in the affected ears. In support of the experimental findings, a human cohort analysis from where the GM samples were obtained found that delivery mode did not affect the children’s risk of developing AD. In conclusion, CS-GM enhanced a Th2 biased immune response, but had no effect on oxazolone-induced dermatitis in mice.

## Introduction

Delivery by CS has progressively increased in recent decades, mainly due to a rise in elective CS performed without strict medical indication.^1–3^ The consequences of CS are given much attention since a growing number of epidemiological studies have associated CS with an increased risk of allergic diseases,^4^ inflammatory bowel disease,^5–7^ obesity,^8,9^ neurodegenerative disorders,^10–13^ among other chronic diseases. Due to the high CS rates this presents a significant public health concern, and in the search of potential underlying mechanisms and new preventative strategies, the focus has mainly been on the critical role of the GM for maturation of the immune system.^14–19^

CS changes the initial intestinal colonization often resulting in a higher abundance of e.g. *Clostridium* spp. and *Enterococcus* spp. and lower abundance of e.g. *Bacteroides* spp. and *Bifidobacterium* spp. compared to vaginally delivered (VD) infants.^14,15,20^ Many of these are well-known gut-colonizing bacteria with substantial impact on the immune system.^21^ CS-delivered mice also show microbiota-mediated immune disturbances with lower proportions of regulatory T cells together with an increased sensitivity to experimental colitis.^22,23^ The early GM is crucial for shifting the immune system from Th2 to Th1 biased pathways and induction of regulatory immunity.^24–27^ Dysbiosis in CS-delivered infants are therefore thought to result in insufficient maturation of the immune system with a skewed balance of Th1/Th2 cells which facilitates an increased risk of allergic diseases.

Atopic dermatitis (AD) is a common chronic inflammatory skin disease, often with early childhood onset. It is a widespread disease with an average prevalence of 20% among children in Europe.^28,29^ The skin inflammation is dominated by Th2 cells in the acute response with a higher expression of IL-4 and IL-13 and enhancement of serum IgE production.^30^ An imbalance in the GM diversity and composition is evident in patients with AD already before any manifestations of the disease occur,^31–33^ strongly indicating that the early GM is crucial for disease development. This has been supported by experimental evidence in an oxazolone-induced dermatitis mouse model, in which the sensitivity to disease was transferable to germ-free mice with fecal transplant of their respective microbiomes.^34^ However, contradictive results on whether CS is associated with AD exist as some studies found an increased relative risk in CS-delivered children,^35–38^ while others point to confounding factors to account for these findings.^4,39–44^ The aim of this study was therefore to clarify the importance of CS-induced dysbiosis in the development of AD.

## Results

Two experimental approaches were taken. Sensitivity to an induced model of AD was tested either in mice born by CS or VD or in mice transplanted with human fecal microbiomes from children born by CS or VD. The oxazolone-induced dermatitis mouse model was used due to its sensitivity to early microbial dysbiosis.^34,45^

### CS delivery enhanced the allergic immune response, but had no effect on the degree of oxazolone-induced dermatitis

**Figure 1.**
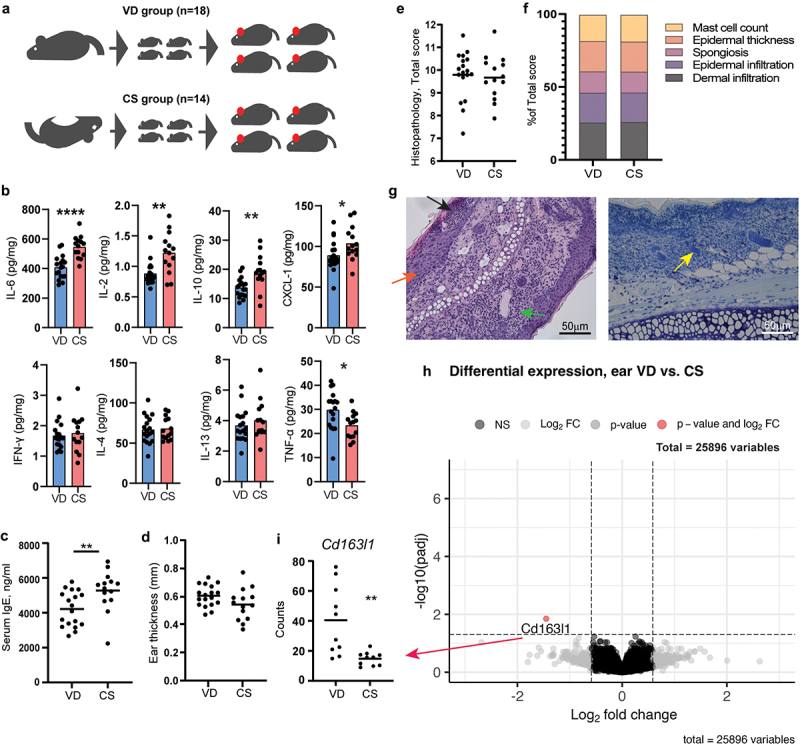
Cesarean section in mice induced a higher IgE response but had no effect on dermatitis. a) sensitivity to oxazolone-induced dermatitis was measured and compared in BALB/c mice delivered by vaginal delivery (VD) or cesarean section (CS). At 8 weeks of age, all mice offspring were sensitized with 0.8% oxazolone on the ear and after 1 week challenged with 0.4% every second day for a total of 5 times. b) multiplex mesoscale results of cytokine concentration (pg/ml/mg tissue) in the inflamed ear after oxazolone challenge. c) ELISA results showing serum concentration (ng/ml) of IgE in serum after oxazolone challenge. d) ear thickness of the inflamed ear in mm measured after oxazolone challenge. e) total histopathology score of hematoxylin and eosin staining cross section of the inflamed ear tissue after oxazolone challenge calculated as the sum of f) dermal infiltration, epidermal infiltration, spongiosis, epidermal thickness, and mast cells present which all were given a severity score from 0 to 3. g) Representative histological images of H&E stained inflamed ear section with mild spongiosis (orange arrow), epidermal infiltrations (black arrow), and dermal infiltration (green arrow), as well as Giemsa stained inflamed ear section with mast cells (yellow arrow). h) volcano plot based on the total transcriptome of the inflamed ear tissue. The red dot represents the *Cd163l1* gene for which gene counts are shown in (i). Bars represent mean. p*<.05, p**<.01, p****<.0001. The experiment was repeated in four litters per group reaching a total of VD, *n* = 18 and CS, *n* = 14 pups which are all shown. There were no litter/round effects in the statistical analyses.

Oxazolone-induced dermatitis was assessed in barrier-bred mice delivered either by CS or VD (Figure 1a). The concentration of IL-2, IL-6, CXCL-1, and IL-10 in the inflamed ear tissue was higher, while TNF-α was lower, in CS delivered mice compared to VD mice (Figure 1b). Also CS-delivered mice had a higher concentration of serum IgE after oxazolone challenge compared to VD mice (Figure 1c), and the proportion of CD8^+^ T cells in ALN was higher (Figure S1A). A lower proportion of CD4^+^ T helper cells in the spleen and of TCRγδ^+^ cells in ALN were present in CS delivered mice (Figure S1B and S1D). Hence, the immune response was increased in CS delivered pups, but not for Th2 cell markers in general, except for the high IgE levels.

In addition, CS had no effect on ear thickness (Figure 1d) or histopathological manifestations in the inflamed ear (Figure 1e,f). Furthermore, no significant differential gene expressions were shown by RNA sequencing of the total transcriptome of the inflamed ear, with the exception of a single gene *Cd163l1* (Figure 1g–h), which verified the lack of change in AD specific inflammatory markers. 16S rRNA gene amplicon sequencing of feces sampled before oxazolone sensitization (8 weeks of age) revealed no long-term differences in GM composition related to delivery mode (Figure S1G-H).

### Human GM was successfully transferred to recipient germ-free mice

**Figure 2.**
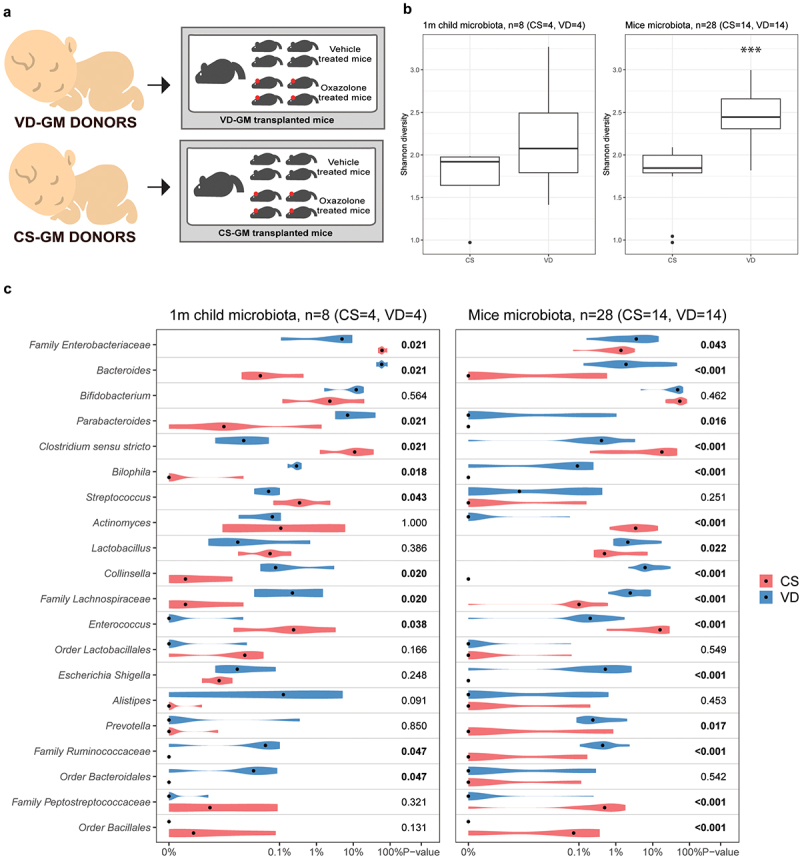
Cesarean section induced gut microbial changes were successfully transferred from human donors to germ-free recipient mice. a) illustration of study setup and how fecal gut microbiota (GM) samples from selected human donors delivered either by cesarean section (CS) or vaginal delivery (VD) were pooled and transferred into a germ-free dam and her offspring for a total of four times per group. Experimental dermatitis was induced in female offspring at the age of 8 weeks by sensitizing with 0.8% oxazolone and after 1 week challenge on both sides of the ear with 0.4% oxazolone every second day for a total of 6 times. b) Shannon α-diversity plot of taxa detected in feces samples from human GM donors (1 month of age, n = 4 per group) and GM transplanted mice (5 weeks of age, n = 14 per group). c) violin plot showing the top 20 overlapping genera in feces samples from human GM donors (1 month of age) and GM transplanted mice (5 weeks of age). Significant *p* values marked in bold indicate a difference in relative abundance according to delivery mode.

Selected human feces samples from CS and VD infants were inoculated into germ-free pregnant mice and the offspring were induced with experimental dermatitis (Figure 2a). Human CS donor samples (*n* = 4) were characterized by a lower abundance of *Bacteroides* spp., *Parabacteroides* spp., *Bilophila* spp., *Collinsella* spp., order of *Bacteroidales*, and family of *Lachnospiraceae* and *Ruminococcaceae*, together with a higher abundance of the family of *Enterobacteriaceae*, *Enterococcus* spp., and *Streptococcus* spp. compared to VD donor samples (*n* = 4) (Figure 2C).

16S gene amplicon sequencing of feces samples obtained from the transplanted mice (CS-GM, *n* = 14 and VD-GM, *n* = 14) at 5 weeks of age revealed that the GM from recipient mice resembled their respective donor GM. Figure 2c illustrates the top 20 most abundant genera within the overlapping genera between donor and recipient samples, in which the abundance of *Bacteroides* spp, *Parabacteroides* spp., *Bilophila* spp., *Collinsella* spp., *Enterococcus* spp and family of *Lachnospiraceae* and *Ruminococcaceae* are different according to delivery mode in both human donors and recipient mice. Moreover, the α-diversity Shannon index was higher in both the VD donors and VD-GM mice compared to the CS donors and CS-GM mice respectively (Figure 2b), though only significant in the mice due to the low sample size of the donors. QIIME2 analysis showed that CS-GM and VD-GM recipient mice clustered separately (Figure S2A-C), and ANCOM analysis revealed 88 genera being significantly different in abundance. Significantly different bacterial genera with a relative abundance above 1% in at least one of the two groups has been listed in supplementary Figure S2D.

### Inoculation with human CS-associated GM enhanced IgE sensitization, but had only minor effects on oxazolone-induced dermatitis

**Figure 3.**
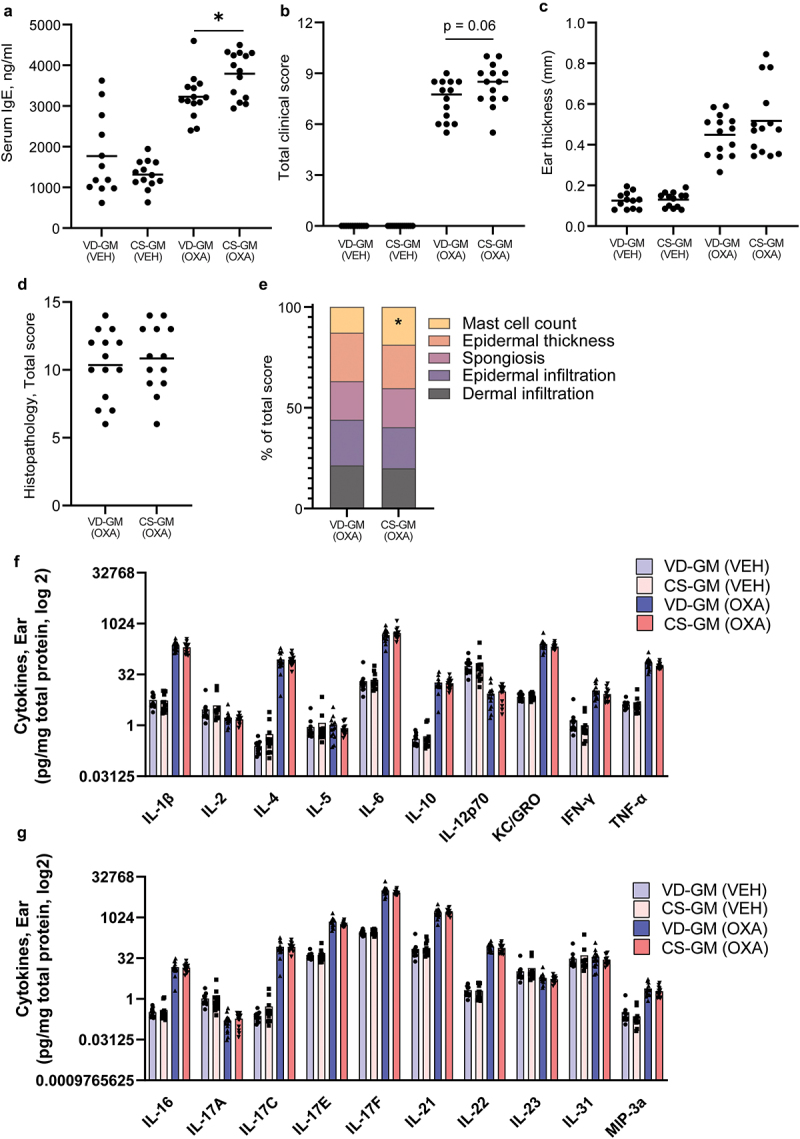
Mice transplanted with human gut microbiota from cesarean section delivered donors had a higher IgE response than vaginally delivered donors, but there was no effect on features of oxazolone-induced dermatitis. a) ELISA results of serum concentration (ng/ml) of IgE after oxazolone challenge. b) total dermatitis score calculated as the sum of flare hemorrhage, edema, excoriation and erosion, and incrustation and xerosis which were all given a score from 0 to 3 corresponding to 0 = no sign; 1 = mild; 2 = moderate; or 3 = severe of the ear of cesarean section gut microbiota (CS-GM) and vaginally delivered gut microbiota (VD-GM) associated mice with oxazolone-induced dermatitis (OXA) or vehicle treated (VEH). c) ear thickness of the inflamed ear in mm measured after oxazolone challenge. d) total histopathology score of hematoxylin and eosin stained cross section of the inflamed ear tissue after oxazolone challenge calculated as the sum of (e) dermal infiltration, epidermal infiltration, spongiosis, epidermal thickness, and mast cells present which all were given a severity score from 0 to 3. f+g) bar plots illustrate mean cytokine concentrations (pg/mg total protein) in the inflamed ear tissue of CS-GM and VD-GM mice with oxazolone-induced dermatitis. Bars represent mean. p*<.05. The experiment was repeated in four litters per group reaching a total of *n* = 14 pups per OXA group and *n* = 12–13 per VEH group which are all shown. There were no litter/round effects in the statistical analyses.

Similar to the CS delivered mice, oxazolone treated mice inoculated with CS-GM had a significantly higher concentration of serum IgE compared to VD-GM (Figure 3a). There was also a tendency (*p* =.06) toward a higher clinical score in the CS-GM mice compared to the VD-GM mice (Figure 3b), but no differences were observed in ear thickness (Figure 3c). There were also no differences in the majority of the histopathological manifestations (Figure 3d, e), except significantly more mast cells in the CS-GM mice compared to the VD-GM mice (Figure 3e).

### Oxazolone-induced dermatitis mice inoculated with CS-GM had more Th2 cells in the auricular lymph node (ALN), but the cytokine production was unaffected by CS-associated GM

**Figure 4.**
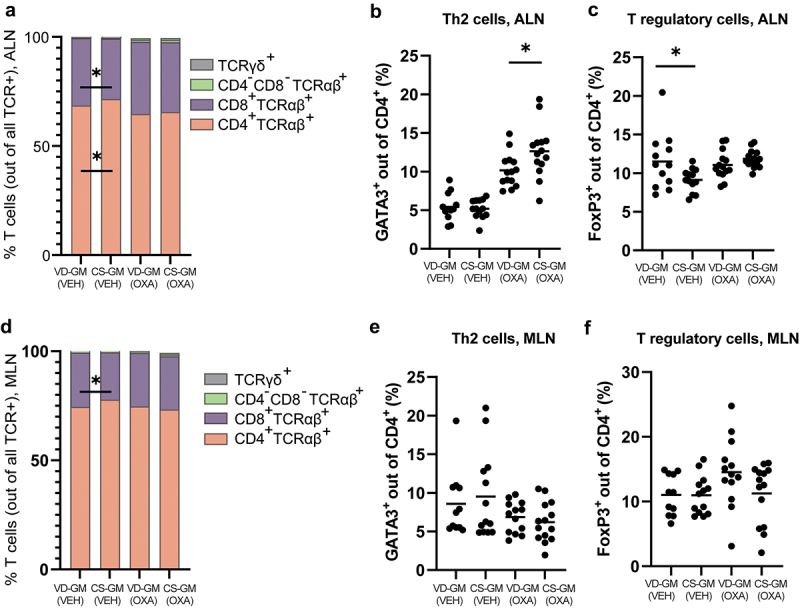
More Th2 and less regulatory T cells in mice transplanted with human gut microbiota from cesarean section delivered donors compared to vaginally delivered donors. a+d) distribution of TCRαβ^+^, TCRγδ^+^, CD4^+^, and CD8^+^ T cells, (b+e) GATA3^+^ Th2 cells, and (c+f) FoxP3^+^ T regulatory cells isolated from the auricular lymph node (ALN) and mesenteric lymph node (MLN) from cesarean section gut microbiota (CS-GM) and vaginally delivered gut microbiota (VD-GM) associated mice with oxazolone-induced dermatitis (OXA) or vehicle treated (VEH). Bars represent mean. p*<0.05. The experiment was repeated in four litters per group reaching a total of *n* = 14 pups per OXA group and *n* = 12–13 per VEH group which are all shown. There were no litter/round effects in the statistical analyses.

Vehicle treated mice inoculated with CS-GM had a higher proportion of CD4^+^TCRαβ^+^ Th cells and a lower proportion of cytotoxic CD8^+^ TCRαβ^+^ T cells in ALN compared to vehicle mice inoculated with VD-GM (Figure 4a). All mice with oxazolone-induced dermatitis had increased proportions of GATA3^+^ Th2 cells compared to the vehicle treated mice, CS-GM mice had a significantly higher proportion of Th2 cells compared to VD-GM mice (Figure 4b). FoxP3^+^ regulatory T cells were not affected by dermatitis induction and independent of GM (Figure 4c), except for the vehicle treated CS-GM mice which had a lower proportion of regulatory T cells in ALN compared to vehicle treated VD-GM mice (Figure 4c). Similar to ALN, a lower proportion of cytotoxic CD8^+^ TCRαβ^+^ T cells in vehicle treated CS-GM mice was also observed in MLN (Figure 4d), but despite that, no differences in T cell distribution were present in MLN according to GM (Figure 4d–f). Hence, the CS-GM induced a Th2 biased immune response in the draining lymph node of the oxazolone treated mice, possibly due to a microbiota-driven reduction in regulatory immunity as observed in the ALN of vehicle treated mice.

A broad panel of cytokines were measured in the ear tissue of both vehicle and oxazolone treated mice. All mice with oxazolone-induced dermatitis had a dramatic increase in IL-1β, IL-4, IL-6, IL-10, TNF-α, IFN-γ, IL-16, IL-17C, IL-17E, IL-17F, IL-21, IL-22, and MIP-3a, whereas the concentrations of IL-12p70, IL-2, IL-17A, and IL-23 were lower in dermatitis mice (Figure 3f,g). There were, however, no differences in the cytokine response between the two inoculated groups CS-GM and VD-GM (Figure 3f,g) supporting the lack of difference found in ear thickness and histological analysis.

### Human CS delivery and CS-associated GM at 1 month of age did not associate with later risk of AD in children

As both CS delivery mode and inoculation with CS-GM from human donors had no effect on the degree of oxazolone-induced dermatitis in the mice, we examined possible associations in the human COPSAC_2010_ cohort, from where the donor fecal samples originated. Among the 662 children with follow up to age 6 years, 210 children (32%) were diagnosed with AD. Of these, the 139 children born by CS (21%) were not in higher risk of developing AD during the first 6 years of life compared to VD children (odds ratio 1.04, 95% CI [0.95–1.13], *p* = 0.42). This was also found when evaluating the risk of AD based on our CS microbial score (degree of microbial perturbation^14^ at 1 month of age (*n* = 557), which is the time point and score used for selecting the human donor samples (odds ratio 1.02, 95% CI [0.98–1.06], *p* = .31).

## Discussion

CS has with considerable evidence been associated with increased risk of chronic diseases such as allergy and asthma, but contradictive results and challenges with numerous confounding factors present a major limitation in demonstrating causality. There is a growing scientific consensus that infants born by CS experience disturbances in their early life microbiome,^15–17^ which may have long-standing consequences on the developing immune system of the children, and hence play an instrumental role in determining their health and disease.^14,46,47^ In support, it has been shown in the COPSAC_2010_ cohort that the increased risk of asthma found in CS delivered children was only present among children, whose microbiome perturbation did not normalize through the first year of life.^14^ In this study, we investigated whether CS and the associated GM dysbiosis, which was not normalized in the first year of life, were associated with changes in the immune response and sensitivity to oxazolone-induced dermatitis in mice.

CS-delivered mice experienced increased serum IgE levels and increased cytokine response in the oxazolone treated ears, but besides that, CS had no effects on the clinical and histopathological phenotype. Especially the IgE response is influenced by early microbial perturbations and low diversity,^48–50^ and it is independent on the later microbiota composition.^45,51^ It has previously been shown that CS in mice has significant effects on the GM, however, the composition of bacteria often vary between studies,^22,23,52–54^ making it difficult to pinpoint certain causative bacteria and translatability to human findings that also vary. Some of the repeated findings in CS-delivered mice across studies are an underrepresentation of *Bacteroide*s spp., *Lactobacillus* spp. and *Ruminococcaceae* members, together with an overrepresentation of *Prevotella* spp. and S24–7 members.^23,52–54^ The low abundance of *Bacteroides* spp. is translational to the CS-induced GM changes often seen in human infants.^14,55,56^ In this study, no changes were found in the GM composition at 8 weeks of age in barrier-bred CS-delivered mice. However, CS-induced GM changes have previously shown to normalize with growing age in both humans^57^ and mice,^23,52^ so it is not unlikely that CS-associated GM changes were present if feces sampling had been done earlier. The high IgE level in CS delivered mice indicates that early dysbiosis was present, though other factors independent of the GM can also alter IgE levels.

In fecal microbiota transplant studies from human to mice the GM of the recipient mice will never resemble the donors 100% due to microbe-host specificity.^58^ Also in the current study, *Bifidobacterium* spp. e.g. propagated to a higher abundance than observed in the donors. However, it is of course essential that the differences in the GM between the groups are transferred. In this study, the donor samples were pooled to avoid the large individual variation between human samples that may transfer differences to the mice not representative to the two groups in the cohort, and the main GM differences observed between the two groups were successfully transferred from human donors to recipient mice with most taxa being present in a proportion resembling the donors.

In a longitudinal study of fecal microbiota maturation during childhood by Galazzo and colleagues,^33^ the initial microbiota around 1 month of age was mainly determined by birth mode. The selection of samples from 1-month-old infants in the current study was significantly enriched by *Bacteroides* spp. in the vaginally born group as in the study by Galazzo *et al*. AD patients show a low relative abundance of intestinal *Bacteroides* spp. compared to healthy children,^20,59–61^ and lack of *Bacteroides* spp. is strongly associated with an immune phenotype characterized by less regulatory T cells,^62^ which was also observed in the CS-GM transplanted vehicle treated mice. Perinatal microbial interventions with e.g. probiotic in experimental animal models for AD that enhances *Bacteroides* species also show alleviating effects on the symptoms,^63–65^ whereas others show the opposite association.^66,67^ Differences in *Bacteroides* strains, synergistic interactions with other bacteria, choice of animal model etc. can explain the varying results. Nonetheless, the CS-GM was not sufficient to exert any major effects on the severity of oxazolone-induced dermatitis in the recipient mice.

Inoculation with CS-GM did, however, induce a Th2 biased immune response with a higher proportion of Th2 cells in ALN, high systemic IgE levels, and increased presence of mast cells in the inflamed ears. The involvement of GM in the pathogenesis of AD is not fully understood, but *Bacteroides* spp. has been shown to suppress cell surface expression of the high-affinity IgE receptor FcεRI on mast cells important for allergen sensitivity.^68^ Moreover, *Bacteroides* spp. can correct the Th1/Th2 imbalance found in germ-free mice via polysaccharide A-activated dendritic cell signaling.^69^ Similar pathways may be involved in the CS-GM-induced changes in the offspring’s immune response. In our study, *Cd163l1* was downregulated in the CS-GM inoculated mice, which is a gene that encodes a scavenger receptor mainly found on immune cells and only slightly expressed in human skin.^70^ While CD163 molecule-like 1, that the gene encodes, has not been associated with AD, CD163 in the same family and a marker for alternatively activated macrophages is increased in mouse models and human patients with AD.^71,72^ This is interesting as the functional relevance of CD163 in dermatitis seems to be related to its immunoregulatory role and antimicrobial defense against staphylococcus aureus, a common microorganism isolated from skin of AD patients.^73^ A prominent Th2 cell response, as observed in the current study, is equivalent to acute stage AD and considering the possible involvement of *Cd163l1* in the initial stages of the disease, the dysbiosis induced by CS is not irrelevant for AD. However, it seems that additional mechanisms are required to predispose the children and mice born by CS to a higher risk of developing AD.

When the disease progresses into more chronic stages, the immune response is constituted of a mixed Th2/Th1/Th22 response,^74–76^ which is also present in the oxazolone-induced dermatitis mice in this study. Repeated oxazolone induces a strong Th2 response with elevated IL-4, IgE levels, and number of Th2 cells compared to vehicle treated mice and induces histopathological characteristic resembling AD in humans.^77^ However, the prominent Th2 response induced by CS related dysbiosis was not sufficient to increase the sensitivity of oxazolone-induced dermatitis in mice, and we were therefore not able to prove an association between CS and AD. In concordance with our findings, analysis of the human cohort used in this study did also not find any indications that CS delivery or CS-associated microbial dysbiosis enhance the risk of AD later in life.

It is important to note, that while some human cohort studies find less *Bifidobacterium* spp. related to birth more,^57^ we and others did not find such an association.^33^ *Bifidobacterium* spp. has never been associated with higher AD risk in CS delivered children, and hence the importance of *Bifidobacterium* spp. for such a link is highly questionable. Nonetheless, considering the fact that fecal microbiota compositions low in *Bifidobacterium* spp. has previously been associated with development of AD and use of probiotics shown to be advantageous,^78^ it would be interesting to repeat our study with samples from human cohorts in which lower abundance of *Bifidobacterium* spp. were detected in CS-delivered children. Differences in GM profiles between human birth cohort studies may explain the varying findings in relation to childhood eczema.

The CS-GM-induced Th2 biased immune response may likely have a more profound impact on allergic diseases driven mainly by Th2 cells and a high IgE response such as allergic asthma and food allergy rather than AD that has a more complex pathogenesis likely requiring more environmental factors involved other than microbiota dependent Th1/Th2 imbalance. In support of this notion, such an association has already been found in the same human cohort between birth by CS and asthma.^14^ Future studies testing the effect of CS-GM in other models of asthma, food allergy, or other AD models including the effect on late recovery would be of high relevance.

## Conclusion

Our aim was to investigate whether GM dysbiosis following birth by CS mitigates the development of AD in the children by combining an experimental approach using two complementary animal models and incorporating human cohort data. CS delivered mice with oxazolone-induced dermatitis had higher cytokine levels in the ear and higher serum IgE compared to VD mice, but these changes were not accompanied by detectable changes in ear thickness, histopathology, or gene expression patterns in the inflamed ear tissue. In the other experiment, germ-free mouse recipients of stool from CS infants, compared to VD infants, had slightly higher serum IgE, more mast cells in the ear, and more Th2 cells in the ALN. However, the majority of the measured features of oxazolone-induced dermatitis were unchanged between the groups despite significant changes in especially *Bacteroides* spp. in their fecal GM. Thus, birth mode and its associated GM seem to induce a Th2 skewed immune response, but do not alter features in an oxazolone-induced mouse model of AD. These results were further supported with an analysis of the human COPSAC_2010_ cohort, which also found that neither delivery mode nor the associated GM profiles of the donor samples affected the children’s risk of AD. In light of this work, preventative microbiota-directed interventions aimed specifically at reducing the incidence of AD in children born by CS seems irrelevant. However, clinical studies with the focus of restoring the GM of CS delivered children are still needed to prevent the microbiota-mediated Th2 biased immune response that may increase the risk of other allergic disorders, possibly more sensitive to the microbial changes following birth by CS.

## Materials and methods

See supplementary material for detailed description of ethics, and details on material and methods.

### Experimental setup

The aim was tested in two experimental setups. Either in mice delivered by VD or CS, or by fecal microbial transplant methods from CS-delivered human infant donors to germ-free recipients as a humanized GM mouse model. In the first experiment, barrier-bred BALB/c mice (Taconic, Lille Skensved, DK) were time-mated for 48 hours and CS was performed at pregnancy day 20 and pups transferred to a foster-mom within 30 minutes. VD pups were transferred to a foster-mom at day 0. In the second experiment, germ-free BALB/c mice (Taconic, Germantown, NY) were inoculated with feces obtained from 1-month-old CS and VD infants, which were selected based on 16S rRNA gene amplicon sequencing data of the COPSAC_2010_ cohort published by Stokholm et al.^14^ All donors were breastfed, and in addition to being born by CS or VD, the infants had a high or low CS microbial score respectively (as described in Stokholm *et al*, ^14^ both at 1 month and 1 year of age, to ensure that the fecal microbial composition in CS children was not restored to a normal microbial trajectory within the first year of life. See supplementary material and methods for further information regarding the COPSAC_2010_ cohort, selection criteria, inoculation procedures, and animal housing.

Only female pups were used for model induction due to space limitations in the isolators and the practical infeasibility of single housing males that frequently fight when the oxazolone model is induced. All dermatitis-induced mice were sensitized at 8 weeks of age with 100 µl 0.8% (w/v) oxazolone (Sigma-Aldrich, St. Louis, MO) dissolved 4:1 in acetone:olive oil on the abdomen. After one week the mice were challenged repeatedly every second day with 10 μl on both sides of the left ear with 0.4% (w/v) oxazolone dissolved 4:1 in acetone:olive oil for a total of 5 challenges for the barrier-bred mice and 6 challenges for the human GM transplanted mice. Euthanization was performed 10 hours after the last challenge. Vehicle mice were treated with the vehicle only in the human GM transplanted mice. In the first experiment vehicle animals were not included due to the lack of significant differences in any of the readouts between the two groups of oxazolone treated mice.

### Clinical and histological scoring of dermatitis

Before euthanasia, the inflamed ear of the human GM transplanted mice was scored blinded by two scientists for each of the clinical signs (1) hemorrhage (2) edema (3) excoriation and (4) incrustation as follows: 0 = no sign; 1 = mild; 2 = moderate; or 3 = severe,^79^ and added to a total dermatitis score. Ear thickness was measured using a micrometer (Mitutoyo Low Force Caliper Series 573, Aurora, Illinois); each measurement was repeated and the calculated mean used.

After euthanasia, the inflamed ear was split into three parts, and the same parts from each mouse were used for either histology, cytokines or RNA seq. Ear tissue for histology embedded in paraffin was cut in 1–2 µm sections using a histiotome MICROM HM355S (Sakura Finetek, Brøndby, Denmark), stained with hematoxylin/eosin and Giemsa, blinded and evaluated by two scientists and given a score from 0 to 3 according to dermal cell infiltration and epidermal spongiosis where 0 = no sign, 1 = mild infiltration/spongiosis, 2 = moderate infiltration/spongiosis, and 3 = severe infiltration/spongiosis. Epithelial thickness were measured and given a score where 0 = <25 μm, 1 = 25 μm-49 μm, 2 = 50 μm-74 μm, and 3 = >75 μm. The amount of mast cells was counted in 6*High power field (HPF) (40X) and the average number was given a score from 0 to 3, where 0 = <10 mast cells/HPF, 1 = 11–20 mast cells/HPF, 2 = 21–30 mast cells/HPF, and 3 = >30 mast cells/HPF. All scores were summarized to a total histopathological score.

### High throughput sequencing of the GM

Feces samples for sequencing were obtained from barrier-bred mice (8 weeks) and human GM transplanted mice (5 weeks). See supplementary material and methods for detailed information on DNA extraction, PCR amplification of 16S rRNA, and sequencing using the Oxford Nanopore GridION x 5 sequencing platform (Oxford Nanopore Technologies, Oxford, UK).

### Flow cytometry

Single cell suspension from the auricular lymph node (ALN), mesenteric lymph nodes (MLN), and spleen were prepared as previously described,^80^ and stained for selected T cell subpopulations for 30 min, 4°C in dark. See supplementary material and methods for antibody information.

### Cytokine analysis

Pre-weighted ear tissue was homogenized in 400 µl lysis buffer (stock solution: 10 ml Tris lysis buffer, 100 µl phosphatase inhibitor 1, 100 µl phosphatase inhibitor 2, and 200 µl protease inhibitor (MSD inhibitor pack, Mesoscale Discovery, Rockville, MD) using a tissue blender (POLYTRON PT 1200 E, Kinematica, Luzern, Switzerland), and centrifuged (7500 g; 4°C; 5 min). Samples were diluted 1:2 and analyzed for IFN-γ, IL-1β, IL-2, IL-4, IL-5, IL-6, IL-10, IL-12p70, KC/GRO, and TNF-α with V-PLEX Proinflammatory Panel 1 Mouse kit (Mesoscale Discovery) and for MIP-3α, IL-16, IL-17A, IL-17C, IL-17E, IL-17F, IL-21, IL-22, IL-23, and IL-31 with V-PLEX Th17 Panel 1 Mouse (Mesoscale Discovery) according to manufacturer’s instructions. Measurements out of detection range were assigned the value of lower or upper detection limit. Concentrations were extrapolated from a standard curve and normalized to total protein measured with Pierce Detergent Compatible Bradford Assay kit according to manufacturer’s protocol.

### Serum IgE

Serum IgE concentrations were measured in serum collected at euthanasia using the Mouse IgE ELISA Kit (Bethyl Laboratories, Montgomery, TX) as previously described.^45^

### Gene expression analysis

RNA from oxazolone treated ears was purified using RNeasy Lipid kit (Qiagen, Hilden, Germany) and send to RNA sequencing at Novogene (Beijing, China) as previously described.^81^ See supplementary material and methods for RNA extraction and sequencing details. Data was analyzed in R (v 4.0.5) using DeSeq2 (v 1.30.1) for differential expression analysis. All gene counts for the individual mice are provided in the Supplementary Table S1.

### Statistics

Statistical analysis was conducted in GraphPad Prism version 9.3.1 (GraphPad Software, San Diego, CA) in regards to clinical output, cytokines, IgE, flow cytometry, and histopathology. For the barrier-bred mice, significance between the two groups (CS vs. VD) was tested by student´s t-test for parametric data and Mann Whitney´s U-test for non-parametric data. D’Agostino-Pearson omnibus test was used for normality test. For the human GM transplanted mice, significance was tested by two-way ANOVA with GM (CS-GM vs. VD-GM) and treatment (oxazolone vs. vehicle) as variables and groups were compared by Šídák’s multiple comparisons test. Histopathology scores from were compared with Mann Whitney´s U-test. *p* values <.05 were considered significant. See supplementary material and methods for statistics used for high throughput data.

## Supplementary Material

Supplemental Material

## Data Availability

The GM 16S sequencing dataset generated for this study can be found in the BioProject database with ID: PRJNA890556. https://eur02.safelinks.protection.outlook.com/?url=https%3A%2F%2Fwww.ncbi.nlm.nih.gov%2Fsra%2FPRJNA890556&data=05%7C01%7Ckrych%40food.ku.dk%7C583eaab1d1694aee1d4b08daaf3fdc60%7Ca3927f91cda14696af898c9f1ceffa91%7C0%7C0%7C638014986071254893%7CUnknown%7CTWFpbGZsb3d8eyJWIjoiMC4wLjAwMDAiLCJQIjoiV2luMzIiLCJBTiI6Ik1haWwiLCJXVCI6Mn0%3D%7C3000%7C%7C%7C&sdata=5ifUQY8qHZEIoHy%2B6k8depDvB33WZfwKu2LfaJyfAi0%3D&reserved=0
