# Supplementary material for: Cesarean section induced dysbiosis promotes type 2 immunity but not oxazolone-induced dermatitis in mice
Source: Gut Microbes. 2023 Oct 27;15(2):2271151. doi: 10.1080/19490976.2023.2271151 (PMC10730161; doi:10.1080/19490976.2023.2271151)
Supplement: Supplemental Material [file KGMI_A_2271151_SM1070.zip › KGMI-SUPPLEMENTAL MATERIAL/Figure S1.docx]

# A B

## Cytotoxic T cells

**40 80**


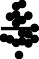

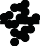


✱

✱✱

CD8α +out of all TCR+(%)

**75**

CD4+ out of CD3 + (%)

**30**

**70**

**20**

**65**

**10 60**

## T helper cells

**0**

# C

**100**

TCRαβ+out of all TCR +(%)

VD CS VD CS SPLEEN ALN

## TCRαβ T cells

**55**

VD CS VD CS SPLEEN ALN

# D

## TCRγδ T cells

**5**


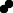


✱

TCRγδ +out of all TCR+(%)

**4**

**95**

**3**

**2**

**90**

**1**

**85**

VD CS VD CS SPLEEN ALN

# E

## T regulatory cells


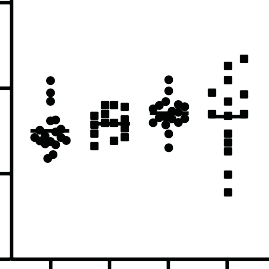
**15**

FoxP3+CD4+out of CD3+(%)

**10**

**5**

**0**

VD CS VD CS SPLEEN ALN

**0**

VD CS VD CS SPLEEN ALN

# F

## CD103+T regulatory cells

CD103+out of FoxP3+CD4+CD3+(%)


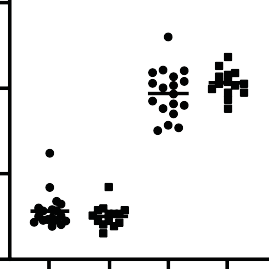
**60**

**40**

**20**

**0**

VD CS VD CS SPLEEN ALN

## G Bray Curtis PCoA H Jaccard PCoA

PC2 = 18%

PC2 = 28%

R2 = 0.031

p = 0.8

VD

CS

VD

CS

R2 = 0.027

p = 0.68

PC1 = 25% PC1 = 53%

Figure S1: T cell distribution in the spleen and auricular lymph node as well as gut microbiota composition in mice delivered by cesarean section or vaginal delivery. Plots illustrate flow cytometry data of the percentage of (A) CD8+ cytotoxic T cells, (B) CD4+ T helper cells, (C) TCRαβ+ T cells, (D) TCRγδ+ T cells, (E) FoxP3+CD4+ T regulatory cells, and (F) CD103+ activated FoxP3+CD4+ T regulatory cells isolated from the spleen and auricular lymph node (ALN) from mice delivered by cesarean section (CS) or vaginally (VD) with oxazolone-induced dermatitis. G) Principal coordinate analysis plot based on Bray Curtis, and (H) Jaccard distance matrix of 16s rRNA gene tag encoded amplicon sequencing of feces obtained at 8 weeks of age before oxazolone sensitization. Bars represent mean. p*<0.05, p**<0.01. The experiment was repeated in four litters per group reaching a total of VD, n=18 and CS, n=14 pups which are all shown. There were no litter/round effects in the statistical analyses.
