# Supplementary material for: Cesarean section induced dysbiosis promotes type 2 immunity but not oxazolone-induced dermatitis in mice
Source: Gut Microbes. 2023 Oct 27;15(2):2271151. doi: 10.1080/19490976.2023.2271151 (PMC10730161; doi:10.1080/19490976.2023.2271151)
Supplement: Supplemental Material [file KGMI_A_2271151_SM1070.zip › KGMI-SUPPLEMENTAL MATERIAL/Figure S2_R1.docx]

**A Bray curtis PCoA plot B Jaccard PCoA plot**

PC2 (16.0%)

PC2 (13.9%)


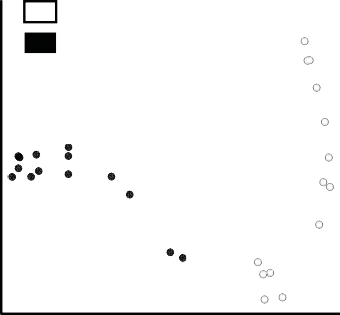


CS-GM VD-GM


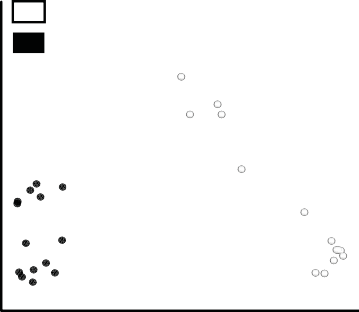


CS-GM VD-GM

PC1 (52.6%) PC1 (64.3%)


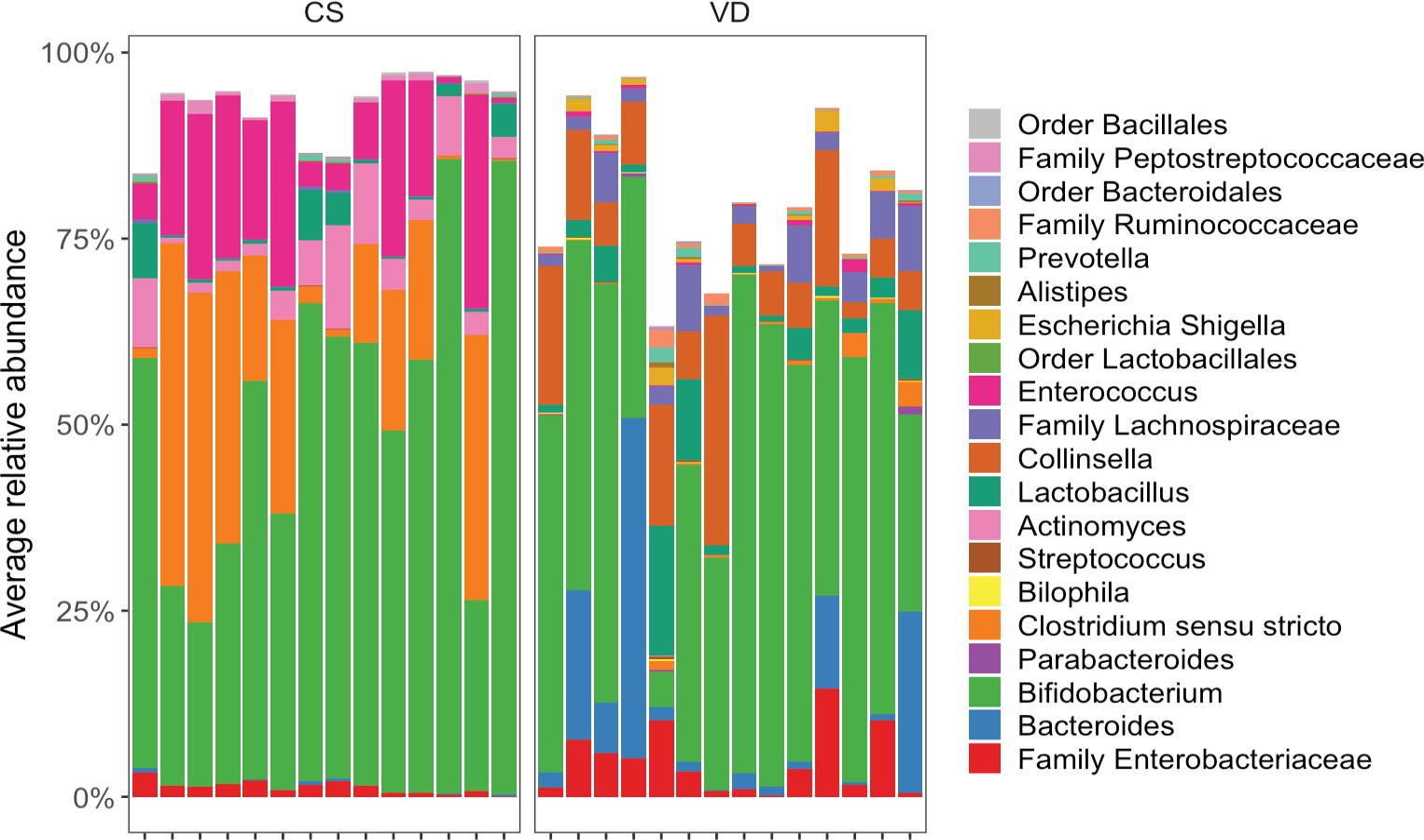
C

CS-GM VD-GM

D

|  |  |  | **Percentile** | | | | | | | | | |
| --- | --- | --- | --- | --- | --- | --- | --- | --- | --- | --- | --- | --- |
|  | **Abundance (%)** | | **0.0** | **25.0** | **50.0** | **75.0** | **100.0** | **0.0** | **25.0** | **50.0** | **75.0** | **100.0** |
| **Taxonomy** | **CS-GM** | **VD-GM** | **CS-GM** | **CS-GM** | **CS-GM** | **CS-GM** | **CS-GM** | **VD-GM** | **VD-GM** | **VD-GM** | **VD-GM** | **VD-GM** |
| Actinobacteria; Actinobacteria; Actinomycetales; Actinomycetaceae; Actinomyces | 4,98 | 0,00 | 632.0 | 1214.25 | 2859.0 | 6134.75 | 16143.0 | 1.0 | 1.0 | 1.0 | 1.0 | 65.0 |
| Actinobacteria; Actinobacteria; Bifidobacteriales; Bifidobacteriaceae; Bifidobacterium | 0,02 | 5,79 | 1.0 | 1.0 | 1.0 | 25.75 | 63.0 | 1.0 | 1879.25 | 2888.5 | 5810.25 | 34873.0 |
| Actinobacteria; Actinobacteria; Bifidobacteriales; Bifidobacteriaceae; Bifidobacterium; adolescentis | 0,08 | 20,84 | 1.0 | 56.0 | 80.5 | 86.5 | 129.0 | 1.0 | 8143.25 | 17008.5 | 24418.75 | 39852.0 |
| Actinobacteria; Actinobacteria; Bifidobacteriales; Bifidobacteriaceae; Bifidobacterium; breve | 3,60 | 1,97 | 579.0 | 1284.0 | 3650.0 | 4149.75 | 6482.0 | 223.0 | 787.75 | 1304.0 | 1987.25 | 5288.0 |
| Actinobacteria; Actinobacteria; Bifidobacteriales; Bifidobacteriaceae; Bifidobacterium; longum | 46,24 | 14,91 | 12183.0 | 19997.0 | 33062.0 | 52024.0 | 80724.0 | 2212.0 | 3583.25 | 6771.0 | 16319.5 | 44274.0 |
| Actinobacteria; Coriobacteriia; Coriobacteriales; Coriobacteriaceae; Collinsella | 0,00 | 2,29 | 1.0 | 1.0 | 1.0 | 1.0 | 1.0 | 162.0 | 395.5 | 1352.5 | 2764.75 | 5247.0 |
| Actinobacteria; Coriobacteriia; Coriobacteriales; Coriobacteriaceae; Collinsella; aerofaciens | 0,00 | 6,33 | 1.0 | 1.0 | 1.0 | 1.0 | 1.0 | 521.0 | 1909.75 | 3440.0 | 8427.0 | 17765.0 |
| Actinobacteria; Coriobacteriia; Coriobacteriales; Coriobacteriaceae; Collinsella; stercoris | 0,00 | 1,71 | 1.0 | 1.0 | 1.0 | 1.0 | 1.0 | 73.0 | 198.25 | 860.0 | 1747.75 | 5078.0 |
| Bacteroidetes; Bacteroidia; Bacteroidales; Bacteroidaceae; Bacteroides | 0,07 | 5,50 | 1.0 | 1.0 | 1.0 | 108.5 | 298.0 | 102.0 | 378.75 | 792.0 | 5072.5 | 31145.0 |
| Firmicutes; Bacilli; Lactobacillales; Enterococcaceae; Enterococcus | 12,75 | 0,39 | 587.0 | 4091.75 | 9293.0 | 12198.0 | 23574.0 | 1.0 | 97.75 | 170.0 | 469.25 | 1146.0 |
| Firmicutes; Clostridia; Clostridiales; Clostridiaceae; Clostridium; butyricum | 13,75 | 0,00 | 80.0 | 905.5 | 8747.0 | 12453.75 | 34119.0 | 1.0 | 1.0 | 1.0 | 1.0 | 1.0 |
| Firmicutes; Clostridia; Clostridiales; Lachnospiraceae | 0,19 | 4,03 | 1.0 | 44.75 | 93.5 | 250.5 | 628.0 | 525.0 | 1742.5 | 2431.5 | 5504.0 | 9560.0 |
| Firmicutes; Clostridia; Clostridiales; Lachnospiraceae; Ruminococcus | 0,00 | 1,30 | 1.0 | 1.0 | 1.0 | 1.0 | 1.0 | 1.0 | 1.0 | 1.0 | 1.0 | 1742.0 |
| Firmicutes; Erysipelotrichi; Erysipelotrichales; Erysipelotrichaceae; Eubacterium; biforme | 0,01 | 9,34 | 1.0 | 1.0 | 1.0 | 1.0 | 51.0 | 1.0 | 1798.25 | 5427.0 | 13228.5 | 20830.0 |
| Proteobacteria; Gammaproteobacteria; Enterobacteriales; Enterobacteriaceae | 1,01 | 4,02 | 73.0 | 329.75 | 627.5 | 955.25 | 2833.0 | 136.0 | 839.75 | 2257.0 | 6309.5 | 9351.0 |

Figure S2: 16sRNA gene tag encoded amplicon sequencing of feces from mice transplanted with human gut microbiota from cesarean section (CS-GM) delivered donors compared to vaginal delivered (VD-GM) donors. Principal coordinate analysis plot based on (A) Bray-curtis and (B) Jaccard distance matrix of 16sRNA gene tag encoded amplicon sequencing of feces obtained from cesarean section gut microbiota (CS-GM) and vaginally delivered gut microbiota (VD-GM) associated mice at 5 weeks of age. C) Average relative abundance of bacterial taxa in the two groups. D) ANCOM analysis results showing significant different bacterial taxa with a relative abundance > 1% in at least one of the two groups.
