# Supplementary material for: Cesarean section induced dysbiosis promotes type 2 immunity but not oxazolone-induced dermatitis in mice
Source: Gut Microbes. 2023 Oct 27;15(2):2271151. doi: 10.1080/19490976.2023.2271151 (PMC10730161; doi:10.1080/19490976.2023.2271151)
Supplement: Supplemental Material [file KGMI_A_2271151_SM1070.zip › KGMI-SUPPLEMENTAL MATERIAL/Supplementary material and methods_GM_R1clean.docx]

**Supplementary Materials and Methods**

***Ethics***

All studies were approved by the Danish Animal Experimentation Inspectorate, Ministry of Food, Fisheries and Agriculture of Denmark (license ID 2017-15-0201-01262) and performed in concordance with the Danish Act on Animal Experimentation (LBK nr 474 of 15/5/2014) and the directive 2010/63/EU about protection of animals in scientific research. Health monitoring was done in accordance with FELASA guidelines.^1^

***The human COPSAC_2010_ cohort***

The Copenhagen Prospective Studies on Asthma in Childhood 2010 (COPSAC_2010_) cohort is an ongoing population-based mother-child cohort of 700 children and their parents, recruited during week 24 of pregnancy and followed prospectively with deep clinical phenotyping during the first 6 years of life as well as acute care visits for respiratory or skin events. The research physicians collected all information during these clinical visits and were responsible for all diagnoses and treatment.^2^ The study was approved by the Local Ethics Committee (H-B-2008-093), and the Danish Data Protection Agency (2015-41-3696). Both parents gave verbal and written informed consent before enrolment.

Atopic dermatitis (AD) diagnosis was made prospectively based on the criteria of Hanifin and Rajka,^3^ requiring the presence of 3 of 4 major criteria and at least 3 of 23 minor criteria. Remission of eczema was defined by 12 months without relapse upon cessation of topical corticosteroid treatment. For analyses, we used the diagnosis to age 6 years.

***Human fecal microbiota transplants***

Human donor fecal samples were obtained at the research clinic or at home and was mixed on arrival with 1 ml of 10% (v/v) glycerol broth and stored at -80 degrees. Donors were selected according to the following inclusion criteria for VD donors: born by VD, breastfed, low CS microbial score (see Stokholm *et al*.^4^ for details on this score) at one month and one year, and for the CS donors: born by CS, breastfed, high CS microbial score at one month and one year. 15 potential donor samples (5 CS, 10 VD) met the inclusion criteria and 4 CS and 4 VD donors were selected according to differences in beta diversity.

The donor samples in each category were pooled together in an anaerobic chamber to obtain enough material, diluted 1:10 in sterile saline with 10% glycerol, and inoculated into two groups of germ-free mice resulting in a CS GM transplanted group (CS-GM) and a VD GM transplanted group (VD-GM). Inoculation was performed by administrating 0.1 ml inoculum to germ-free pregnant dams and 0.05 ml to pups at day 0, 7 and 21 by dripping the inoculum into their mouth and onto the mammae of the dam. A power analysis was performed before the experiments to estimate the needed group sizes and pups from a minimum of three litters per group were used to avoid litter and cage effects.

***Animal housing conditions***

Barrier-bred and germ-free BALB/c mice were housed in our AAALAC accredited barrier protected and germ-free facility (Faculty of Health and Medical Sciences, University of Copenhagen, Frederiksberg, Denmark) in open cages with free access to Altromin 1324 diet (Brogaarden, Lynge, Denmark) and tap water. Germ-free animals were housed in HEPA-ventilated isolators (PFI systems, Milton Keynes, UK) with free access to irradiated Altromin 1314 diet (Brogaarden, irradiated with 2*25 Gy) and sterile water.

***High throughput sequencing of the gut microbiota***

Feces sampled from barrier-bred mice (8 weeks) and human GM transplanted mice (5 weeks) were sampled and stored at -80°C until further processing. DNA from fecal samples was extracted using Bead-Beat Micro AX Gravity Kit (A&A Biotechnology, Gdynia, Poland) according to manufacturer’s instructions. The DNA purity and concentration were determined by NanoDrop 1000 Spectrophotometer (Thermo Fisher Scientific, MA, USA) and Varioskan Flash (Thermo Fisher Scientific, MA, USA), respectively.

To determine gut microbiome composition, near full-length 16S rRNA gene was amplified with multiple forward and reverse primers containing unique molecular identifier (UMI) sequence (Table S1). Two-step PCR was performed for the amplification and barcoding. First PCR conditions were as follows: 95°C for 5 min, 2 cycles of 95°C for 20 s, 48°C for 30 s, 65°C for 10 s, 72°C for 45 s, and a final extension at 72°C for 4 min. A second PCR step was done to barcode first PCR products with the following conditions: 95°C for 2 min followed by 33 cycles of 95°C for 20 s, 55°C for 20 s, 72°C for 40 s, and a final extension at 72°C for 4 min. PCR products were cleaned up using SpeedBeadsTM magnetic carboxylate (obtained from Sigma Aldrich) after each PCR step. The size of barcoded PCR products was checked on 1.5% agarose gel.

Nanopore sequencing libraries were constructed according to the ligation sequencing kit SQK-LSK109 protocol (Oxford Nanopore Technologies, Oxford, UK) and run on the Oxford Nanopore GridION x 5 sequencing platform (Oxford Nanopore Technologies, Oxford, UK) using two R9.4.1 flowcells. Sequenced data were collected using Nanopore sequencing software GridION version 21.02.5 (https://nanoporetech.com). ONT’s Guppy version 4.5.2 (https://nanoporetech.com) was used for base calling and demultiplexing. Next, filtering and trimming of demultiplexed sequences (min=1300bp, max=1600bp, q score ≥10) were performed by Nanofilt version 2.7.1.^5^ Taxonomy assignment was done by parallel_assign_taxonomy_uclust.py script of Quantitative Insights into Microbial Ecology (Qiime) 1 version 1.8.0.^6^ Greengenes database version 13.8.^7^ was used as a reference database. The reads classifications did not contain UMI correction because of the low coverage of UMI clusters.

Stool samples from the human cohort were collected 1 month after birth, sent to the laboratory and frozen at -80°C. DNA was extracted using MoBio PowerSoil kits on an epMotion 5075, amplified using a two-step PCR reaction with 515F and 806R primers flanking the V4 region of 16S rRNA gene, and sequenced using the v2 kit (PE250bp reads) on the MiSeq platform (Illumina Inc., San Diego CA). Bioinformatics pipelines and calculation of the CS microbial scores have previously been published.^4^

***Flow cytometry***

Single cell suspension from the auricular lymph node (ALN) and the spleen were stained with the following flourochrome-conjugated anti-mouse antibodies: FITC-conjugated anti-CD4, APC-conjugated anti-TCRγδ, PerCP-cyanine5.5-conjugated anti-TCRαβ and PE-conjugated anti-CD8α in one panel and FITC-conjugated anti-CD4, APC-conjugated anti-Rorγt, PerCP-cyanine5.5-conjugated anti-FoxP3 and PE-conjugated anti-GATA3 in the second panel (all purchased from eBioscience, San Diego, CA) and analyzed on an Accuri C6 flow cytometer (Accuri Cytometers, Ann Arbor, MI).

***Gene expression analysis***

Mouse ear tissue from 10 VD and 10 CS delivered mice was homogenized in 1 mL Qiazol lysis buffer using a Tissuelyzer II (Qiagen, Hilden, Germany), and RNA was purified using the Qiagen RNeasy Lipid Mini Kit (Qiagen, Hilden, Germany) where chloroform was substituted with 1-bromo-3-chloropropane (Sigma-Aldrich, Darmstadt, Germany), and the optional DNase treatment step was added to the protocol (Qiagen, Hilden, Germany). RNA integrity was assessed on 1,4 % agarose gel and 2 µg total RNA per sample was sent for RNA sequencing at Novogene (Beijing, China) where RNA libraries were prepared for sequencing using standard Illumina protocols and sequenced on an Illumina Novaseq 6000 platform. Illumina CASAVA software was used for basecalling and adaptors were trimmed with Fastp, filtered for low quality sequence and mapped to Mus Musculus (GRCm38/mm10) genome with HISAT2 v 2.0.5 and a gene count file made using FeatureCounts v 1.5.0.

***Statistics***

Ear tissue RNAseq count data was analyzed in R (v. 4.0.5) using the DESeq2 package with standard settings (v. 1.30.1),^8^ and the volcano plot was generated using EnhancedVolcano package (v. 1.8.0). An adjusted p value < 0.05 and a fold change ± 1.5 was considered a significant difference for all gene expression data.

Differences in GM alpha and beta diversity analysis were performed using Qiime 2 version 2019.4.0. The feature table were subsampled to 12,000 and 35,000 reads/sample respectively for the conventional and the human GM transplanted mice. The subsampling value was adjusted based on the sequencing depth (85% of the most indigent samples within the dataset). Principal coordinate analysis (PCoA) was conducted on Bray-Curtis and Jaccard dissimilarity UniFrac metrics, a PERMANOVA test with FDR correction was performed to detect pairwise group differences. Differences in relative abundance at single OTU level between control and treatment groups was analyzed using Analysis of Composition of Microbes (ANCOM). The relative abundances of overlapping genera between human donor and mice samples were analyzed according to delivery group using Wilcoxon rank-sum tests in the statistical software package R version 4.1.2, with the package phyloseq,^9^ to handle the microbiome data.

**References**

1. Mahler Convenor M, Berard M, Feinstein R, Gallagher A, Illgen-Wilcke B, Pritchett-Corning K, Raspa M. FELASA recommendations for the health monitoring of mouse, rat, hamster, guinea pig and rabbit colonies in breeding and experimental units. Lab Anim. 2014; 48:178-192.

2. Bisgaard H, Vissing NH, Carson CG, Bischoff AL, Følsgaard NV, Kreiner-Møller E, Chawes BL, Stokholm J, Pedersen L, Bjarnadóttir E, et al. Deep phenotyping of the unselected COPSAC2010 birth cohort study. Clin Exp Allergy. 2013; 43:1384-1394.

3. Hanifin J, Rajka G. Diagnostic features of atopic dermatitis. Acta Derm Venereol. 1980; 92:44-47.

4. Stokholm J, Thorsen J, Blaser MJ, Rasmussen MA, Hjelmsø M, Shah S, Christensen ED, Chawes BL, Bønnelykke K, Brix S, et al. Delivery mode and gut microbial changes correlate with an increased risk of childhood asthma. Science Translational Medicine. 2020; 12:eaax9929.

5. De Coster W, D’Hert S, Schultz DT, Cruts M, Van Broeckhoven C. NanoPack: visualizing and processing long-read sequencing data. Bioinformatics. 2018; 34:2666-2669.

6. Caporaso JG, Kuczynski J, Stombaugh J, Bittinger K, Bushman FD, Costello EK, Fierer N, Pena AG, Goodrich JK, Gordon JI, et al. QIIME allows analysis of high-throughput community sequencing data. Nat Methods. 2010; 7:335-356.

7. McDonald D, Price MN, Goodrich J, Nawrocki EP, DeSantis TZ, Probst A, Andersen GL, Knight R, Hugenholtz P. An improved Greengenes taxonomy with explicit ranks for ecological and evolutionary analyses of bacteria and archaea. Isme j. 2012; 6:610-618.

8. Love MI, Huber W, Anders S. Moderated estimation of fold change and dispersion for RNA-seq data with DESeq2. Genome Biol. 2014; 15:550

9. McMurdie PJ, Holmes S. phyloseq: An R Package for Reproducible Interactive Analysis and Graphics of Microbiome Census Data. PLoS One. 2013; 8:e61217.
