# Supplementary material for: Cesarean section induced dysbiosis promotes type 2 immunity but not oxazolone-induced dermatitis in mice
Source: Gut Microbes. 2023 Oct 27;15(2):2271151. doi: 10.1080/19490976.2023.2271151 (PMC10730161; doi:10.1080/19490976.2023.2271151)
Supplement: Supplemental Material [file KGMI_A_2271151_SM1070.zip › KGMI-SUPPLEMENTAL MATERIAL/Supplementary_table_1_Ear_VD_CS_geneCounts.docx]

| **Supplementary Table 1. Gene counts from RNA seq of the oxazolone-treated ear from each mouse born vaginally or by cesarean section are given as indicated in the first row**  ,"gene_id","CS_OXA_2","CS_OXA_3","CS_OXA_4","CS_OXA_5","CS_OXA_8","CS_OXA_9","CS_OXA11","CS_OXA12","CS_OXA13","CS_OXA14","VD_OXA_2","VD_OXA_3","VD_OXA_4","VD_OXA_5","VD_OXA_8","VD_OXA_9","VD_OXA10","VD_OXA12","VD_OXA13","VD_OXA20" | ENSMUSG00000065401,"ENSMUSG00000065401",0,0,0,0,0,0,0,0,0,0,0,0,0,0,0,0,0,0,0,0  ENSMUSG00000114470,"ENSMUSG00000114470",10,10,10,3,10,20,12,13,8,8,15,5,12,11,3,18,10,16,9,3 ENSMUSG00000052525,"ENSMUSG00000052525",14,8,11,18,9,8,18,7,3,8,5,5,9,5,2,15,9,10,5,5 | ENSMUSG00000102362,"ENSMUSG00000102362",0,0,0,0,0,0,0,0,0,0,0,0,0,0,0,0,0,0,0,0  ENSMUSG00000102191,"ENSMUSG00000102191",0,2,0,0,0,0,0,0,0,0,0,0,0,0,0,0,0,0,0,0 ENSMUSG00000103193,"ENSMUSG00000103193",0,0,0,0,0,0,0,0,0,0,0,0,0,0,0,0,0,0,0,0 |
| --- | --- | --- |
| ENSMUSG00000064351,"ENSMUSG00000064351",124046,119106,133873,123308,164280,117273,127291,108176,124627,131248,137766,106516,131938,111384,116252,122058,113283,154602,104767,134285 | ENSMUSG00000039492,"ENSMUSG00000039492",1,1,5,1,2,1,3,5,5,1,0,4,4,1,0,2,5,4,2,3 | ENSMUSG00000105092,"ENSMUSG00000105092",0,0,0,0,0,0,0,0,0,0,0,0,0,0,0,0,0,0,0,0 |
| ENSMUSG00000029368,"ENSMUSG00000029368",21,20,41,12,28,42,42,64,77,18,27,30,43,33,52,18,25,23,81,104 | ENSMUSG00000101258,"ENSMUSG00000101258",4,0,1,3,4,4,0,0,0,0,2,3,4,2,2,2,1,7,1,0 | ENSMUSG00000106293,"ENSMUSG00000106293",0,0,0,0,1,0,0,0,0,0,0,0,1,0,0,0,0,0,1,0 |
| ENSMUSG00000037071,"ENSMUSG00000037071",10507,9994,8694,13377,11043,11658,14819,7494,12395,11687,9875,8590,9255,11328,7906,7886,8957,11516,8643,9434 | ENSMUSG00000037014,"ENSMUSG00000037014",2,1,5,3,0,4,4,3,5,3,3,4,5,6,2,2,1,9,1,5 | ENSMUSG00000102629,"ENSMUSG00000102629",0,0,0,0,0,0,0,0,0,0,0,0,0,0,0,2,0,0,0,0 |
| ENSMUSG00000027559,"ENSMUSG00000027559",1206,1723,1656,1652,2463,1453,1828,1475,1633,2132,1399,1132,2142,1627,1721,1392,1965,2154,1868,1788 | ENSMUSG00000117192,"ENSMUSG00000117192",3,9,10,15,8,9,13,15,17,16,8,3,12,7,5,2,4,5,3,5 | ENSMUSG00000105012,"ENSMUSG00000105012",0,0,0,0,0,0,0,0,0,0,0,0,0,0,0,0,0,0,0,0 |
| ENSMUSG00000064370,"ENSMUSG00000064370",29386,44569,48870,43039,57186,41874,49387,35705,46367,50283,50301,39988,44718,40899,40601,41496,40410,52244,34686,46209 | ENSMUSG00000050875,"ENSMUSG00000050875",0,3,6,3,4,2,4,2,4,2,9,3,5,1,2,5,0,0,3,6 | ENSMUSG00000104265,"ENSMUSG00000104265",0,0,0,0,0,0,0,0,0,0,0,0,0,0,0,0,0,0,0,0 |
| ENSMUSG00000037742,"ENSMUSG00000037742",62215,81558,104866,104539,95635,92375,93037,73457,82440,86336,98252,77605,86521,88520,80258,97534,76087,101784,76935,84271 | ENSMUSG00000114375,"ENSMUSG00000114375",3,20,55,28,36,48,23,37,24,25,52,17,35,28,23,36,40,47,27,21 | ENSMUSG00000078691,"ENSMUSG00000078691",0,0,0,0,0,0,0,0,0,0,0,0,0,0,0,0,0,0,0,0 |
| ENSMUSG00000026879,"ENSMUSG00000026879",11419,13674,16002,19670,16028,14277,15786,13367,14501,15447,17489,14344,17580,15862,15031,14169,14690,16403,13791,16023 | ENSMUSG00000067121,"ENSMUSG00000067121",6,11,13,10,13,11,10,8,7,13,10,8,12,11,8,7,7,16,0,11 | ENSMUSG00000093093,"ENSMUSG00000093093",0,0,0,0,0,0,0,0,0,0,0,0,0,0,0,0,0,0,0,2 |
| ENSMUSG00000002985,"ENSMUSG00000002985",11023,25451,41237,30156,26911,28182,28571,26483,27280,28330,33000,20027,33295,29905,27009,28783,27427,34801,36620,29311 | ENSMUSG00000035745,"ENSMUSG00000035745",13,13,12,8,10,27,11,13,12,11,14,11,9,16,17,25,6,13,17,13 | ENSMUSG00000105819,"ENSMUSG00000105819",0,0,1,0,0,1,0,0,0,0,1,0,0,0,0,1,0,0,0,0 |
| ENSMUSG00000064341,"ENSMUSG00000064341",29731,41323,45697,38911,52122,36727,39401,29166,37767,40585,42091,34955,40127,33137,36352,36339,34623,49427,29701,44039 | ENSMUSG00000091866,"ENSMUSG00000091866",5,8,13,13,7,9,2,6,13,12,9,6,13,16,12,14,6,12,8,30 | ENSMUSG00000106174,"ENSMUSG00000106174",0,0,0,0,0,0,1,0,0,0,1,1,0,0,0,1,0,0,0,0 |
| ENSMUSG00000028255,"ENSMUSG00000028255",55,125,98,154,126,79,75,42,93,75,134,101,66,96,64,99,67,62,95,34 | ENSMUSG00000092482,"ENSMUSG00000092482",6,13,8,6,13,3,14,13,10,13,10,6,8,16,10,13,4,17,11,18 | ENSMUSG00000091175,"ENSMUSG00000091175",0,0,0,0,0,0,0,0,0,0,0,0,0,0,0,0,0,0,0,0 |
| ENSMUSG00000064367,"ENSMUSG00000064367",33715,28958,29071,29300,37812,24980,28398,22006,25550,27373,30028,23903,27884,23144,26206,24851,25328,32663,20662,26986 | ENSMUSG00000109772,"ENSMUSG00000109772",0,0,0,0,0,0,0,0,0,0,0,0,0,0,0,0,0,0,0,0 | ENSMUSG00000077085,"ENSMUSG00000077085",0,0,0,0,0,0,0,0,0,0,0,0,0,0,0,3,0,0,0,0 |
| ENSMUSG00000062515,"ENSMUSG00000062515",829,1054,1423,1192,1536,1545,992,899,1008,1607,1137,976,1431,1300,1074,1136,1222,1532,1213,1333 | ENSMUSG00000052616,"ENSMUSG00000052616",3,11,0,10,5,1,11,3,3,7,7,7,4,3,6,6,7,6,1,4 | ENSMUSG00000060256,"ENSMUSG00000060256",0,0,0,0,0,0,0,0,0,0,0,0,0,0,0,0,0,0,0,0 |
| ENSMUSG00000025515,"ENSMUSG00000025515",9,5,9,12,8,15,19,15,7,7,22,22,25,10,17,14,20,25,16,11 | ENSMUSG00000047370,"ENSMUSG00000047370",12,6,15,10,9,8,9,9,7,8,8,9,25,10,14,13,12,10,5,20 | ENSMUSG00000094328,"ENSMUSG00000094328",0,0,0,0,0,0,0,0,0,0,0,0,0,0,0,0,0,0,0,0 |
| ENSMUSG00000047730,"ENSMUSG00000047730",5414,6866,6987,13320,10442,2375,9178,4188,6556,5743,9149,9171,4880,7059,7161,5498,5301,3851,4742,3291 | ENSMUSG00000020591,"ENSMUSG00000020591",1,2,4,4,3,0,1,3,5,1,1,3,1,3,2,4,3,2,6,4 | ENSMUSG00000087720,"ENSMUSG00000087720",0,0,0,0,0,0,0,0,0,0,0,0,0,0,0,0,0,0,0,0 |
| ENSMUSG00000071356,"ENSMUSG00000071356",0,3,1,5,0,0,0,0,0,4,1,0,0,0,0,1,1,2,0,0 | ENSMUSG00000079808,"ENSMUSG00000079808",6,20,23,16,18,19,17,35,29,29,22,10,21,33,30,26,22,46,27,32 | ENSMUSG00000097539,"ENSMUSG00000097539",0,0,0,1,0,0,0,0,2,0,0,0,0,0,0,0,0,0,0,0 |
| ENSMUSG00000029580,"ENSMUSG00000029580",56697,59031,68835,64935,62203,64038,68143,59068,67736,68077,62230,48146,63948,72239,59074,68670,57945,71879,50998,74476 | ENSMUSG00000086714,"ENSMUSG00000086714",8,5,8,6,5,2,3,5,1,6,8,11,7,8,1,5,4,8,4,3 | ENSMUSG00000106486,"ENSMUSG00000106486",0,0,0,0,0,0,0,0,0,0,0,0,0,0,0,0,0,0,0,0 |
| ENSMUSG00000069919,"ENSMUSG00000069919",409,399,902,536,1538,572,819,285,208,326,502,578,655,377,494,603,871,662,341,118 | ENSMUSG00000076499,"ENSMUSG00000076499",7,1,9,3,5,4,7,3,9,2,9,17,8,8,4,7,1,10,1,1 | ENSMUSG00000106316,"ENSMUSG00000106316",0,0,0,0,0,0,0,0,0,0,0,0,0,0,0,0,0,0,0,0 |
| ENSMUSG00000064345,"ENSMUSG00000064345",19805,15867,16202,14868,20122,14131,17075,13175,16217,18045,16406,14579,16063,13995,14570,13521,14733,17970,13137,18092 | ENSMUSG00000114014,"ENSMUSG00000114014",9,14,15,19,23,15,15,8,13,17,16,15,9,30,14,9,8,32,14,9 | ENSMUSG00000106453,"ENSMUSG00000106453",0,0,0,0,0,0,0,0,0,0,0,0,0,1,0,0,0,0,0,0 |
| ENSMUSG00000052305,"ENSMUSG00000052305",664,596,1356,836,2299,917,1056,427,487,493,833,1034,1073,552,770,877,1284,993,599,224 | ENSMUSG00000024593,"ENSMUSG00000024593",5,12,14,15,23,7,10,8,15,18,14,9,10,9,11,11,9,10,11,10 | ENSMUSG00000081506,"ENSMUSG00000081506",0,0,0,0,0,0,0,0,0,0,0,0,0,0,0,0,0,0,0,0 |
| ENSMUSG00000024164,"ENSMUSG00000024164",5401,7659,10251,9815,9566,7337,8591,10488,8871,11171,10099,6591,14237,11653,10956,9998,10305,13078,9480,11501 | ENSMUSG00000049598,"ENSMUSG00000049598",0,14,3,8,1,174,4,3,2,0,5,3,3,8,5,24,5,7,0,14 | ENSMUSG00000106510,"ENSMUSG00000106510",0,0,0,0,0,0,0,0,1,0,0,0,0,0,0,3,0,0,0,0 |
| ENSMUSG00000034994,"ENSMUSG00000034994",25256,33229,41958,43298,37155,34980,41930,29569,35422,33272,37084,31332,34270,36076,31845,38942,31044,41950,35729,37346 | ENSMUSG00000082884,"ENSMUSG00000082884",3,3,3,3,0,1,3,1,3,3,4,4,7,8,0,10,5,4,3,7 | ENSMUSG00000104607,"ENSMUSG00000104607",0,0,0,1,0,0,0,0,0,0,0,0,1,0,0,0,0,0,0,0 |
| ENSMUSG00000064363,"ENSMUSG00000064363",13678,16637,17719,15433,22867,15435,18993,13707,17399,18049,17860,14739,17193,14844,15330,14633,15424,19038,15669,17631 | ENSMUSG00000013353,"ENSMUSG00000013353",34,40,45,35,48,70,55,44,39,71,39,25,48,85,37,78,37,58,15,46 | ENSMUSG00000098042,"ENSMUSG00000098042",0,0,0,0,0,0,0,0,0,0,0,0,0,0,0,0,0,0,0,0 |
| ENSMUSG00000025479,"ENSMUSG00000025479",4,7,1,7,10,1,4,12,10,6,1,0,5,0,5,1,10,2,12,12 | ENSMUSG00000112120,"ENSMUSG00000112120",25,37,37,56,64,45,41,65,36,37,58,63,51,42,44,56,58,47,49,49 | ENSMUSG00000104902,"ENSMUSG00000104902",0,0,0,0,0,0,0,0,0,0,0,0,0,0,0,0,0,0,0,0 |
| ENSMUSG00000049382,"ENSMUSG00000049382",14,15,24,31,16,20,19,12,26,29,16,15,9,15,10,12,8,10,11,10 | ENSMUSG00000087721,"ENSMUSG00000087721",39,23,33,23,25,23,16,11,15,33,28,15,17,24,19,19,23,27,20,6 | ENSMUSG00000106094,"ENSMUSG00000106094",0,0,0,0,0,0,0,0,0,0,0,0,0,0,0,0,0,0,0,0 |
| ENSMUSG00000045545,"ENSMUSG00000045545",91967,145249,229610,182345,170971,150171,147294,144283,154904,177240,186086,161092,190337,161071,167344,228487,147920,249533,155325,209254 | ENSMUSG00000085311,"ENSMUSG00000085311",25,28,24,34,29,14,27,22,30,32,15,19,15,20,22,27,31,34,19,12 | ENSMUSG00000105939,"ENSMUSG00000105939",0,0,2,0,0,0,0,0,0,0,0,0,0,0,0,0,0,0,1,0 |
| ENSMUSG00000020609,"ENSMUSG00000020609",5,1,0,0,0,0,1,2,7,0,0,0,0,0,0,0,0,0,4,9 | ENSMUSG00000097766,"ENSMUSG00000097766",13,15,17,22,3,21,16,10,16,23,11,12,14,19,9,22,15,8,7,10 | ENSMUSG00000065447,"ENSMUSG00000065447",0,0,0,0,0,0,0,0,0,0,0,0,0,0,0,0,0,2,0,0 |
| ENSMUSG00000026417,"ENSMUSG00000026417",1,6,1,9,3,1,5,4,1,6,7,1,2,6,1,2,3,1,2,4 | ENSMUSG00000114784,"ENSMUSG00000114784",26,32,26,28,27,16,14,15,14,24,25,15,23,23,21,32,20,31,18,12 | ENSMUSG00000104556,"ENSMUSG00000104556",0,0,0,0,0,0,0,0,0,0,0,0,0,0,0,0,0,0,0,0 |
| ENSMUSG00000024661,"ENSMUSG00000024661",23299,32003,42294,28740,38021,41039,31750,37321,36754,46993,31300,23226,46601,41165,34097,46189,36242,48485,27502,45850 | ENSMUSG00000090223,"ENSMUSG00000090223",0,0,0,0,0,0,0,0,0,0,1,0,0,0,0,0,1,0,0,0 | ENSMUSG00000104617,"ENSMUSG00000104617",0,0,0,1,0,0,0,0,0,0,0,0,0,0,0,0,0,0,0,0 |
| ENSMUSG00000015568,"ENSMUSG00000015568",865,770,1240,829,1241,1007,796,1045,900,884,1093,755,1597,814,907,805,937,1203,893,988 | ENSMUSG00000052133,"ENSMUSG00000052133",8,7,13,8,8,6,13,10,9,5,15,5,15,9,7,8,15,13,6,5 | ENSMUSG00000104610,"ENSMUSG00000104610",0,0,0,0,0,0,1,0,0,0,0,0,0,0,0,0,0,0,0,0 |
| ENSMUSG00000064373,"ENSMUSG00000064373",13468,12439,23845,16847,17317,15421,10915,10501,11847,15748,16028,10005,11849,13042,11072,14370,12123,13841,9730,10268 | ENSMUSG00000116707,"ENSMUSG00000116707",11,10,12,17,16,15,14,12,10,5,15,12,0,6,11,13,12,11,12,3 | ENSMUSG00000106462,"ENSMUSG00000106462",0,0,0,0,0,0,0,0,0,0,0,0,0,0,0,0,0,0,0,0 |
| ENSMUSG00000025393,"ENSMUSG00000025393",12191,14009,18842,18272,16841,14729,16322,13578,14780,16379,18464,14099,16117,16500,15708,17357,14569,19494,17081,16569 | ENSMUSG00000101133,"ENSMUSG00000101133",3,4,10,6,11,5,4,8,4,3,3,2,5,6,7,5,1,8,1,3 | ENSMUSG00000105317,"ENSMUSG00000105317",0,1,0,0,0,0,0,1,0,0,0,0,0,0,0,1,0,0,0,0 |
| ENSMUSG00000022868,"ENSMUSG00000022868",0,0,6,0,2,0,4,9,8,0,3,1,1,0,0,3,2,2,16,13 | ENSMUSG00000100514,"ENSMUSG00000100514",3,12,12,28,24,8,7,6,15,23,19,21,12,18,17,14,13,14,10,14 | ENSMUSG00000065270,"ENSMUSG00000065270",0,0,0,1,0,0,0,0,0,0,0,0,0,0,0,0,0,0,0,0 |
| ENSMUSG00000060126,"ENSMUSG00000060126",31059,32659,36724,36561,33754,30716,31010,24029,31155,38671,34816,30100,29758,31778,29969,36833,31061,36267,27501,31311 | ENSMUSG00000060093,"ENSMUSG00000060093",0,15,3,1,4,6,6,1,0,4,7,2,7,2,6,6,4,5,2,6 | ENSMUSG00000083872,"ENSMUSG00000083872",0,0,0,0,3,0,0,0,0,0,1,0,0,0,0,0,0,0,0,0 |
| ENSMUSG00000074272,"ENSMUSG00000074272",280,243,338,270,294,360,287,263,218,269,297,176,347,294,250,283,279,425,230,299 | ENSMUSG00000020401,"ENSMUSG00000020401",0,3,3,4,6,5,5,7,4,10,5,5,10,10,10,8,4,5,13,9 | ENSMUSG00000081592,"ENSMUSG00000081592",0,0,0,0,0,0,0,0,0,0,0,0,0,0,0,0,0,0,0,0 |
| ENSMUSG00000032083,"ENSMUSG00000032083",9,16,22,1,14,18,21,40,23,19,18,6,27,15,12,17,15,27,23,47 | ENSMUSG00000025380,"ENSMUSG00000025380",30,50,43,43,54,27,29,53,55,40,25,39,30,55,28,47,26,52,37,34 | ENSMUSG00000084119,"ENSMUSG00000084119",0,0,0,0,0,0,0,0,2,0,0,0,0,0,2,0,0,0,0,0 |
| ENSMUSG00000047517,"ENSMUSG00000047517",0,3,0,1,2,2,2,0,1,5,0,1,2,0,2,0,2,0,4,1 | ENSMUSG00000033615,"ENSMUSG00000033615",0,5,11,7,3,1,4,2,7,2,3,3,1,2,1,1,3,5,2,1 | ENSMUSG00000085824,"ENSMUSG00000085824",0,0,0,0,0,0,0,0,0,0,0,0,0,0,0,0,0,0,0,0 |
| ENSMUSG00000039062,"ENSMUSG00000039062",1039,1808,2489,2404,2424,2169,1605,1996,2078,1957,2386,1815,2513,2149,1775,2348,2022,2633,1700,2002 | ENSMUSG00000025489,"ENSMUSG00000025489",10,19,29,16,61,37,28,25,26,26,31,25,27,44,21,42,23,32,21,32 | ENSMUSG00000083956,"ENSMUSG00000083956",0,0,0,0,0,0,0,0,0,0,0,0,0,0,0,0,0,0,0,0 |
| ENSMUSG00000021939,"ENSMUSG00000021939",32155,28673,47564,40120,35364,34155,29777,36008,31915,37422,41359,25087,44140,35796,34855,39237,33686,42543,31507,38099 | ENSMUSG00000089776,"ENSMUSG00000089776",26,44,43,69,60,43,62,19,47,54,36,32,25,57,47,33,31,36,20,30 | ENSMUSG00000080057,"ENSMUSG00000080057",0,0,0,0,0,0,0,0,0,0,0,0,0,0,0,0,0,0,0,0 |
| ENSMUSG00000027513,"ENSMUSG00000027513",27,35,42,11,24,10,33,91,39,89,34,28,86,25,56,19,74,60,45,91 | ENSMUSG00000115856,"ENSMUSG00000115856",0,3,0,2,0,0,1,0,0,0,1,0,4,0,0,0,1,0,0,2 | ENSMUSG00000082628,"ENSMUSG00000082628",0,0,0,0,0,3,0,0,1,0,0,0,0,0,0,0,0,0,0,0 |
| ENSMUSG00000025153,"ENSMUSG00000025153",2328,2983,3168,4295,3465,3255,3848,2690,3546,2775,3721,3694,3340,3346,2899,3006,2826,3392,3396,3240 | ENSMUSG00000063245,"ENSMUSG00000063245",30,40,44,22,36,33,25,17,19,34,35,18,31,24,38,23,33,38,24,22 | ENSMUSG00000081483,"ENSMUSG00000081483",0,0,0,0,0,0,0,0,0,0,0,0,0,0,0,0,0,0,0,0 |
| ENSMUSG00000030017,"ENSMUSG00000030017",150,181,369,290,242,193,207,170,240,250,235,201,270,238,215,199,147,244,248,189 | ENSMUSG00000005503,"ENSMUSG00000005503",0,0,0,0,0,0,0,0,0,0,0,0,0,0,0,0,0,0,0,0 | ENSMUSG00000082242,"ENSMUSG00000082242",0,0,0,0,0,0,0,0,0,0,0,0,0,0,0,0,0,0,0,0 |
| ENSMUSG00000013643,"ENSMUSG00000013643",8,7,6,5,3,7,7,3,7,7,10,3,1,4,3,8,9,7,4,0 | ENSMUSG00000030125,"ENSMUSG00000030125",1,4,6,3,1,0,3,5,1,12,1,4,3,2,5,4,4,4,7,4 | ENSMUSG00000083153,"ENSMUSG00000083153",0,0,0,0,0,0,0,0,0,0,0,0,0,0,0,0,0,0,0,0 |
| ENSMUSG00000092274,"ENSMUSG00000092274",18579,30033,39449,45335,38965,27030,38305,32217,28952,28665,46326,31083,43726,36814,34736,37275,25432,40930,46773,30673 | ENSMUSG00000002190,"ENSMUSG00000002190",10,7,8,6,9,6,9,5,4,7,6,2,6,5,9,6,7,8,3,7 | ENSMUSG00000083338,"ENSMUSG00000083338",0,0,0,0,0,0,0,0,0,0,0,0,0,0,0,0,0,0,0,0 |
| ENSMUSG00000033161,"ENSMUSG00000033161",10565,12940,15538,17819,14144,13488,14088,10400,12750,12615,16502,13962,12303,13678,12459,13549,11227,14804,12126,11735 | ENSMUSG00000027831,"ENSMUSG00000027831",3,4,8,8,12,15,7,5,8,8,7,4,22,5,14,3,18,21,11,12 | ENSMUSG00000081862,"ENSMUSG00000081862",0,0,0,0,0,0,0,0,0,0,0,0,0,0,0,0,0,0,0,0 |
| ENSMUSG00000060802,"ENSMUSG00000060802",21225,26187,30509,35736,34344,27139,27574,33602,31010,33254,35322,25422,37323,29720,30864,40521,31686,44384,34441,36342 | ENSMUSG00000110368,"ENSMUSG00000110368",46,41,52,57,58,51,30,37,43,40,48,53,28,43,40,65,48,49,23,37 | ENSMUSG00000070908,"ENSMUSG00000070908",0,0,0,0,0,0,0,0,0,0,0,0,0,0,0,0,0,0,0,0 |
| ENSMUSG00000004207,"ENSMUSG00000004207",14512,18234,27035,22331,21991,22916,19158,22596,20345,21815,24553,14105,27094,22805,19909,23886,21289,29850,20612,24459 | ENSMUSG00000043498,"ENSMUSG00000043498",4,1,7,3,6,18,2,8,8,3,4,6,5,14,5,6,6,11,2,5 | ENSMUSG00000045364,"ENSMUSG00000045364",0,0,0,1,1,0,0,0,0,0,0,0,0,0,0,0,0,0,0,0 |
| ENSMUSG00000020719,"ENSMUSG00000020719",18949,20986,23153,23724,23857,22743,22677,17729,21877,23868,22714,18704,20077,22851,19333,22259,19693,24520,17610,20396 | ENSMUSG00000039058,"ENSMUSG00000039058",4,11,4,11,4,11,11,9,3,5,6,5,9,7,7,15,0,10,2,5 | ENSMUSG00000085209,"ENSMUSG00000085209",0,0,0,0,0,0,0,0,0,0,0,0,0,0,0,0,0,0,0,0 |
| ENSMUSG00000073411,"ENSMUSG00000073411",25018,27195,34434,39278,35054,30300,30393,33235,32444,33306,35890,27923,41266,35780,31529,42954,32154,45413,29341,38965 | ENSMUSG00000085914,"ENSMUSG00000085914",2,1,1,4,6,2,3,4,6,1,4,6,6,2,5,7,7,9,12,4 | ENSMUSG00000087332,"ENSMUSG00000087332",0,0,0,0,0,0,0,0,0,0,0,0,0,0,0,0,0,0,0,0 |
| ENSMUSG00000030359,"ENSMUSG00000030359",0,4,0,0,0,0,0,1,6,0,0,0,2,2,0,1,3,1,10,5 | ENSMUSG00000095847,"ENSMUSG00000095847",5,12,10,8,11,7,12,6,12,8,11,7,14,11,13,11,10,7,3,9 | ENSMUSG00000073789,"ENSMUSG00000073789",0,0,0,0,0,0,0,0,0,0,0,0,0,0,1,0,0,0,0,0 |
| ENSMUSG00000023041,"ENSMUSG00000023041",97210,88958,110265,125283,110732,77572,104268,89209,98773,93290,116067,110138,109331,95986,115347,130959,90661,133333,99876,110309 | ENSMUSG00000097823,"ENSMUSG00000097823",2,7,5,10,13,2,9,3,7,2,11,8,9,3,2,5,2,6,9,3 | ENSMUSG00000035201,"ENSMUSG00000035201",0,0,0,0,0,0,0,0,0,0,1,0,0,0,0,0,0,0,0,0 |
| ENSMUSG00000062825,"ENSMUSG00000062825",45781,41253,43916,47056,46097,41708,53212,35579,41078,44593,34754,33159,33225,46377,39509,45037,38763,46071,35711,40339 | ENSMUSG00000028351,"ENSMUSG00000028351",18,16,32,17,36,41,15,43,38,10,31,8,44,31,42,37,47,27,16,31 | ENSMUSG00000037028,"ENSMUSG00000037028",4,0,0,0,0,0,0,0,0,0,0,0,0,0,0,0,0,0,0,0 |
| ENSMUSG00000026043,"ENSMUSG00000026043",38753,39136,66523,49912,45209,84979,51450,53993,42423,45623,51015,35512,71561,45785,50589,51019,44207,68138,39579,56997 | ENSMUSG00000021747,"ENSMUSG00000021747",0,4,3,1,4,2,1,1,3,1,3,2,4,8,2,0,5,6,1,2 | ENSMUSG00000085041,"ENSMUSG00000085041",0,0,0,0,0,0,0,0,0,1,0,0,0,0,0,1,0,0,0,0 |
| ENSMUSG00000030278,"ENSMUSG00000030278",29,78,99,77,117,87,100,124,106,183,97,97,150,143,132,93,117,116,156,131 | ENSMUSG00000087452,"ENSMUSG00000087452",1,1,2,4,1,0,0,1,2,0,1,0,1,2,0,3,0,1,0,0 | ENSMUSG00000081823,"ENSMUSG00000081823",0,0,0,0,0,0,0,0,0,0,0,0,0,0,0,0,0,0,0,0 |
| ENSMUSG00000049775,"ENSMUSG00000049775",14362,13329,18973,14033,16180,16738,13451,14585,12834,15255,15444,10129,17727,14319,13364,16402,13924,18729,13921,16295 | ENSMUSG00000013338,"ENSMUSG00000013338",1,2,3,1,2,1,0,4,2,1,0,5,5,2,4,5,0,4,3,3 | ENSMUSG00000088902,"ENSMUSG00000088902",0,0,0,0,0,0,0,0,0,0,0,0,0,0,0,0,0,0,0,0 |
| ENSMUSG00000025428,"ENSMUSG00000025428",10613,11391,14355,13881,13055,11675,12168,9930,11055,12774,14399,11089,12702,12250,11833,13513,11577,14884,11824,12673 | ENSMUSG00000106757,"ENSMUSG00000106757",17,7,14,12,8,14,15,12,8,12,16,17,10,4,4,8,25,11,13,12 | ENSMUSG00000082207,"ENSMUSG00000082207",0,0,0,0,0,0,0,0,0,0,0,0,0,0,0,0,0,0,0,0 |
| ENSMUSG00000035540,"ENSMUSG00000035540",0,0,2,2,1,0,5,6,10,2,1,1,1,2,0,0,1,5,12,8 | ENSMUSG00000109599,"ENSMUSG00000109599",0,2,3,1,2,5,3,5,0,7,3,1,12,3,0,3,2,4,4,1 | ENSMUSG00000095676,"ENSMUSG00000095676",0,2,0,0,0,0,0,0,0,0,0,0,0,0,0,0,0,0,0,0 |
| ENSMUSG00000058207,"ENSMUSG00000058207",1,5,1,14,4,4,6,15,10,4,7,9,2,7,8,1,0,2,19,11 | ENSMUSG00000104724,"ENSMUSG00000104724",12,8,3,2,2,13,18,10,9,20,5,5,9,12,20,16,5,10,3,5 | ENSMUSG00000082143,"ENSMUSG00000082143",0,0,0,0,0,0,0,0,0,0,0,0,1,0,0,0,0,1,0,0 |
| ENSMUSG00000002944,"ENSMUSG00000002944",909,771,773,929,936,911,789,711,730,1158,858,640,956,905,707,778,992,1049,639,841 | ENSMUSG00000105929,"ENSMUSG00000105929",1,12,9,9,10,12,17,5,8,6,11,9,20,6,4,13,4,4,1,15 | ENSMUSG00000087464,"ENSMUSG00000087464",0,0,0,0,0,0,0,0,0,0,0,0,0,0,0,2,0,0,0,0 |
| ENSMUSG00000023944,"ENSMUSG00000023944",14434,18142,22330,23455,19667,19235,23933,17172,20372,19270,21843,17918,18114,19898,18767,19626,17339,22719,18413,21507 | ENSMUSG00000076463,"ENSMUSG00000076463",1,6,2,6,6,4,0,4,0,5,7,2,3,3,2,5,1,2,1,6 | ENSMUSG00000096876,"ENSMUSG00000096876",0,0,0,0,0,0,0,0,0,0,0,0,0,0,0,0,0,0,0,0 |
| ENSMUSG00000023010,"ENSMUSG00000023010",10158,10286,13096,13352,12323,11590,11947,11340,12067,12045,12121,9452,12496,12393,11020,14163,10815,15097,9800,13017 | ENSMUSG00000044086,"ENSMUSG00000044086",33,44,46,42,48,13,47,31,32,59,41,42,53,35,29,41,46,41,36,55 | ENSMUSG00000089381,"ENSMUSG00000089381",0,0,0,0,0,0,0,0,0,0,0,0,0,0,0,0,0,0,0,0 |
| ENSMUSG00000059908,"ENSMUSG00000059908",0,0,0,0,0,0,5,3,3,0,0,0,0,0,0,0,0,0,7,6 | ENSMUSG00000085057,"ENSMUSG00000085057",5,3,0,0,3,1,1,6,4,2,2,1,2,8,3,0,4,3,0,5 | ENSMUSG00000084196,"ENSMUSG00000084196",0,0,0,0,0,0,0,0,0,0,0,0,0,0,0,0,0,0,0,0 |
| ENSMUSG00000032399,"ENSMUSG00000032399",16660,18742,22747,22403,19669,19165,20179,14446,17304,20235,21413,18186,17722,18629,17586,21362,17264,21887,16172,18401 | ENSMUSG00000038259,"ENSMUSG00000038259",9,17,20,35,34,12,19,22,23,26,13,13,12,24,13,24,16,10,32,17 | ENSMUSG00000098422,"ENSMUSG00000098422",0,0,0,0,0,0,0,0,0,0,0,0,0,0,0,0,0,0,0,0 |
| ENSMUSG00000067274,"ENSMUSG00000067274",25199,18382,20515,20573,18262,16659,18529,12692,17720,18484,20118,17664,15732,17040,16140,20452,16128,19795,17926,15571 | ENSMUSG00000084230,"ENSMUSG00000084230",18,2,15,8,3,10,7,4,5,6,7,2,3,7,1,7,9,6,4,10 | ENSMUSG00000085804,"ENSMUSG00000085804",0,0,0,0,0,0,0,0,0,0,0,0,0,0,0,1,0,0,0,0 |
| ENSMUSG00000028603,"ENSMUSG00000028603",1823,1770,2250,2331,2211,2139,1965,1713,1833,2024,2331,1725,2245,1980,1752,2072,1683,2354,1982,2000 | ENSMUSG00000097292,"ENSMUSG00000097292",3,2,5,4,1,1,3,0,0,0,2,1,2,3,3,0,1,6,2,1 | ENSMUSG00000086856,"ENSMUSG00000086856",0,0,0,0,0,0,0,0,0,0,0,0,0,0,0,0,0,0,0,0 |
| ENSMUSG00000035557,"ENSMUSG00000035557",50993,73572,86986,116395,98857,60241,94627,90055,88496,74529,96099,95743,83635,95561,99370,114301,80721,102282,92538,112471 | ENSMUSG00000086358,"ENSMUSG00000086358",8,2,3,8,6,6,4,2,0,0,6,6,3,4,6,12,2,6,6,2 | ENSMUSG00000080597,"ENSMUSG00000080597",0,0,0,0,0,0,0,0,0,0,0,0,0,0,0,0,1,0,0,0 |
| ENSMUSG00000053797,"ENSMUSG00000053797",72369,72178,99683,107334,98605,72492,90744,74650,83759,80472,93694,87640,89036,83750,86739,107297,81705,121115,97261,97149 | ENSMUSG00000054034,"ENSMUSG00000054034",4,17,28,28,23,8,16,26,28,47,32,30,47,31,29,37,39,27,30,37 | ENSMUSG00000073747,"ENSMUSG00000073747",0,0,0,0,0,0,0,0,0,0,0,0,0,0,1,0,0,3,0,0 |
| ENSMUSG00000025509,"ENSMUSG00000025509",812,1178,1563,1490,1507,1182,1304,1334,1451,1759,1320,1155,1497,1578,1315,1550,1237,1737,1309,1418 | ENSMUSG00000049123,"ENSMUSG00000049123",5,6,8,7,5,4,2,4,2,13,15,3,5,9,4,3,7,5,5,10 | ENSMUSG00000093294,"ENSMUSG00000093294",0,0,0,0,0,0,0,0,0,0,0,0,0,0,0,0,0,0,0,0 |
| ENSMUSG00000030695,"ENSMUSG00000030695",8230,12707,17705,16902,18002,13658,15640,18486,14937,14224,16122,13950,21006,15477,16914,19555,14862,22032,19063,21677 | ENSMUSG00000062081,"ENSMUSG00000062081",4,12,14,20,13,7,10,5,12,16,11,17,6,13,10,19,17,15,4,18 | ENSMUSG00000094359,"ENSMUSG00000094359",0,0,0,0,0,0,0,0,0,0,0,0,0,3,0,0,0,0,0,0 |
| ENSMUSG00000066154,"ENSMUSG00000066154",1,1,0,0,0,0,12,13,6,0,0,7,0,0,0,0,0,0,11,22 | ENSMUSG00000112605,"ENSMUSG00000112605",6,5,12,16,10,12,4,7,6,7,13,13,22,12,8,16,8,9,1,10 | ENSMUSG00000085968,"ENSMUSG00000085968",0,0,0,0,0,0,0,0,0,1,0,0,0,0,0,0,0,0,0,0 |
| ENSMUSG00000049350,"ENSMUSG00000049350",0,2,0,0,0,0,0,2,1,0,2,0,0,0,4,0,0,1,0,2 | ENSMUSG00000001566,"ENSMUSG00000001566",0,0,0,0,0,0,0,0,0,0,0,0,0,1,1,1,0,0,0,0 | ENSMUSG00000086164,"ENSMUSG00000086164",0,0,0,0,0,0,0,0,0,0,0,0,0,0,0,0,1,0,0,0 |
| ENSMUSG00000063856,"ENSMUSG00000063856",6532,6954,9830,7566,7816,7452,7015,7240,7126,7379,8256,6523,9084,7020,7779,10285,6874,10893,7469,9282 | ENSMUSG00000106209,"ENSMUSG00000106209",11,29,27,34,25,23,24,15,15,25,18,19,23,31,23,33,18,28,19,19 | ENSMUSG00000084238,"ENSMUSG00000084238",0,2,0,0,0,0,2,0,0,0,0,0,0,0,0,0,0,0,0,0 |
| ENSMUSG00000020911,"ENSMUSG00000020911",1,3,1,6,3,8,2,1,5,3,5,3,7,4,7,7,8,1,5,5 | ENSMUSG00000111777,"ENSMUSG00000111777",18,20,20,17,15,8,25,10,16,14,15,12,18,16,15,14,12,22,10,25 | ENSMUSG00000087903,"ENSMUSG00000087903",0,0,0,0,0,0,0,0,0,0,0,0,0,0,0,0,0,0,0,0 |
| ENSMUSG00000061780,"ENSMUSG00000061780",116,183,179,222,228,160,149,180,193,311,193,133,217,256,248,100,190,214,245,262 | ENSMUSG00000019301,"ENSMUSG00000019301",2,8,6,4,15,16,13,5,7,13,11,4,10,17,2,9,8,11,10,17 | ENSMUSG00000083403,"ENSMUSG00000083403",0,2,0,0,2,0,0,0,0,0,0,0,0,0,0,0,0,0,0,0 |
| ENSMUSG00000022108,"ENSMUSG00000022108",10454,13074,20583,18640,16035,15120,12424,13473,14236,14325,17243,12674,17096,14290,13479,17752,13982,19641,14360,15065 | ENSMUSG00000097673,"ENSMUSG00000097673",0,0,1,0,0,1,0,0,1,0,0,0,0,1,0,4,0,0,0,0 | ENSMUSG00000081227,"ENSMUSG00000081227",0,0,0,0,0,0,0,0,0,0,0,0,0,0,0,2,0,0,0,0 |
| ENSMUSG00000053964,"ENSMUSG00000053964",75,98,132,155,119,102,111,102,114,113,161,140,163,133,123,137,98,179,126,101 | ENSMUSG00000087036,"ENSMUSG00000087036",2,2,4,6,1,1,1,2,1,0,2,5,8,2,0,0,2,1,2,2 | ENSMUSG00000078511,"ENSMUSG00000078511",0,0,0,0,4,0,0,0,0,0,0,0,0,0,0,0,0,0,0,0 |
| ENSMUSG00000032518,"ENSMUSG00000032518",20855,19532,26321,23062,21410,21209,20833,16602,18663,21401,23586,18371,20858,20547,18817,23750,18524,24892,18410,20591 | ENSMUSG00000103081,"ENSMUSG00000103081",23,20,32,19,10,25,9,9,23,23,19,11,24,11,24,9,25,24,12,12 | ENSMUSG00000046262,"ENSMUSG00000046262",0,0,0,0,0,0,0,0,0,0,0,0,0,0,0,0,0,0,0,0 |
| ENSMUSG00000005610,"ENSMUSG00000005610",25114,19688,20889,23133,22028,19181,21016,14466,19270,21045,21677,18438,16251,18923,17435,20249,17650,21138,14149,16254 | ENSMUSG00000034336,"ENSMUSG00000034336",4,16,7,15,12,7,21,4,26,26,17,15,9,12,11,7,8,5,12,18 | ENSMUSG00000083173,"ENSMUSG00000083173",0,0,0,0,0,0,0,0,0,0,0,0,0,1,0,0,1,0,0,0 |
| ENSMUSG00000061808,"ENSMUSG00000061808",0,0,1,0,0,0,8,11,6,1,0,4,0,1,0,1,0,0,10,9 | ENSMUSG00000107230,"ENSMUSG00000107230",3,4,2,6,10,5,5,9,4,11,7,7,6,8,4,5,7,10,5,4 | ENSMUSG00000106054,"ENSMUSG00000106054",0,0,0,0,0,0,0,0,0,0,0,0,0,0,0,0,0,0,0,0 |
| ENSMUSG00000056035,"ENSMUSG00000056035",0,0,0,0,0,0,2,5,3,0,0,0,0,0,0,0,0,0,4,4 | ENSMUSG00000053550,"ENSMUSG00000053550",0,2,2,8,0,2,8,8,3,0,0,6,0,0,0,0,2,0,0,3 | ENSMUSG00000105536,"ENSMUSG00000105536",0,0,0,1,0,0,0,0,0,0,0,0,0,0,1,0,0,0,0,0 |
| ENSMUSG00000057666,"ENSMUSG00000057666",14711,22270,31487,25616,31625,25532,23780,28100,23404,24472,25161,21782,31310,25861,26421,32653,24480,35855,26634,31406 | ENSMUSG00000064672,"ENSMUSG00000064672",9,3,4,4,12,1,7,7,5,4,2,8,9,15,6,9,4,8,4,7 | ENSMUSG00000104672,"ENSMUSG00000104672",0,0,0,0,0,0,0,0,0,0,0,0,0,0,0,0,0,0,0,0 |
| ENSMUSG00000026193,"ENSMUSG00000026193",15090,19350,32198,22274,27812,29594,22411,21157,20614,29046,22489,15201,27592,27619,24425,20430,25183,20099,15109,22512 | ENSMUSG00000097723,"ENSMUSG00000097723",19,12,21,11,16,15,8,11,5,11,8,4,15,12,7,23,9,20,9,10 | ENSMUSG00000105310,"ENSMUSG00000105310",0,0,0,0,0,0,0,0,0,0,2,0,0,0,0,0,0,0,0,0 |
| ENSMUSG00000026864,"ENSMUSG00000026864",17384,15746,22964,23849,21026,20507,21487,18558,17903,19365,20476,14673,18988,21237,20111,21155,17588,21627,15809,19776 | ENSMUSG00000102691,"ENSMUSG00000102691",12,3,3,12,9,7,5,11,3,8,7,4,4,6,4,6,8,4,9,10 | ENSMUSG00000096269,"ENSMUSG00000096269",0,0,0,0,0,0,0,1,0,0,0,0,0,0,0,0,0,0,0,0 |
| ENSMUSG00000063229,"ENSMUSG00000063229",13232,13919,21386,19161,20289,17658,14692,18828,15132,15806,18240,15730,21354,17368,17929,22300,15909,25228,19068,21372 | ENSMUSG00000066196,"ENSMUSG00000066196",2,4,1,3,2,1,3,1,3,3,1,1,0,3,2,7,1,8,1,2 | ENSMUSG00000105254,"ENSMUSG00000105254",0,0,0,0,0,0,0,0,0,0,0,0,0,0,0,0,0,0,0,0 |
| ENSMUSG00000028001,"ENSMUSG00000028001",95,95,178,87,98,155,115,107,142,182,131,67,129,158,110,131,137,158,114,160 | ENSMUSG00000097879,"ENSMUSG00000097879",8,3,19,5,12,16,5,9,2,8,8,9,8,7,7,6,10,7,4,12 | ENSMUSG00000105052,"ENSMUSG00000105052",0,0,0,0,0,0,0,0,0,0,0,0,0,0,0,0,0,0,0,0 |
| ENSMUSG00000076617,"ENSMUSG00000076617",211,141,219,175,164,199,163,245,176,156,226,177,362,160,209,272,227,386,246,215 | ENSMUSG00000107102,"ENSMUSG00000107102",21,16,6,6,16,11,12,5,10,21,24,6,10,7,10,11,4,19,4,8 | ENSMUSG00000090671,"ENSMUSG00000090671",0,0,0,0,0,0,0,0,0,0,0,0,0,0,0,0,0,0,0,0 |
| ENSMUSG00000069833,"ENSMUSG00000069833",22721,26595,32313,36984,32875,27269,26551,23059,26402,32453,31020,25185,26162,32049,28226,32138,28497,34565,17119,25447 | ENSMUSG00000076589,"ENSMUSG00000076589",0,0,0,0,0,0,0,0,0,0,0,0,0,0,0,0,0,0,0,0 | ENSMUSG00000105946,"ENSMUSG00000105946",0,0,0,0,0,0,0,0,1,0,0,0,0,0,1,0,0,0,0,0 |
| ENSMUSG00000018593,"ENSMUSG00000018593",11216,12725,21007,16003,15365,25250,15972,16632,14568,15712,17022,11209,22995,15216,15387,16518,15589,20093,15430,19709 | ENSMUSG00000107745,"ENSMUSG00000107745",24,33,30,30,53,42,71,36,32,48,36,43,27,43,54,51,54,48,40,34 | ENSMUSG00000088514,"ENSMUSG00000088514",0,0,0,0,0,0,0,0,0,0,0,0,0,0,0,0,0,0,0,0 |
| ENSMUSG00000032231,"ENSMUSG00000032231",16519,25519,31226,32440,29894,26807,28521,22074,24336,30101,28481,25276,25299,29525,26838,30685,24872,32556,22703,27819 | ENSMUSG00000109165,"ENSMUSG00000109165",2,4,3,12,9,3,3,2,1,5,17,8,4,4,6,3,5,7,5,0 | ENSMUSG00000081200,"ENSMUSG00000081200",1,0,2,0,0,0,0,0,0,0,0,0,0,0,0,0,0,0,0,0 |
| ENSMUSG00000025991,"ENSMUSG00000025991",0,4,6,5,2,6,7,2,4,11,4,0,0,4,2,4,2,7,5,1 | ENSMUSG00000109097,"ENSMUSG00000109097",0,0,0,0,0,0,0,0,0,0,3,1,0,0,0,0,1,0,0,1 | ENSMUSG00000115557,"ENSMUSG00000115557",0,0,0,0,0,0,0,0,0,0,0,0,0,0,0,0,0,0,0,0 |
| ENSMUSG00000015656,"ENSMUSG00000015656",13558,14047,16553,16292,15108,14935,16094,11612,13834,15059,15436,11417,13420,14448,13301,15321,12923,14824,12808,13184 | ENSMUSG00000099270,"ENSMUSG00000099270",0,0,0,0,0,0,0,0,0,0,0,0,0,0,0,0,0,0,0,0 | ENSMUSG00000107070,"ENSMUSG00000107070",2,0,0,0,0,0,0,0,0,0,0,0,0,1,0,0,0,0,0,0 |
| ENSMUSG00000025130,"ENSMUSG00000025130",12803,13720,19929,18251,19985,17933,16081,18816,15923,15770,17988,14605,21286,18081,17902,20538,15992,21428,15314,20056 | ENSMUSG00000114638,"ENSMUSG00000114638",7,5,12,18,8,17,6,3,6,7,16,12,7,9,10,5,7,20,3,14 | ENSMUSG00000106214,"ENSMUSG00000106214",0,0,0,0,0,0,0,0,0,0,0,0,0,0,0,0,0,0,0,1 |
| ENSMUSG00000040249,"ENSMUSG00000040249",7816,11934,18126,15521,16721,17099,12813,14757,13715,16418,16223,9669,20722,16153,14929,17889,16060,22030,12011,16126 | ENSMUSG00000096577,"ENSMUSG00000096577",0,0,0,0,0,0,0,0,0,0,0,0,0,0,1,0,0,0,0,0 | ENSMUSG00000105332,"ENSMUSG00000105332",0,0,0,0,0,0,0,0,0,0,0,0,0,0,0,0,0,0,0,0 |
| ENSMUSG00000001175,"ENSMUSG00000001175",12097,10110,13758,12395,12339,11605,11044,10412,10550,12055,12335,9179,12310,11612,10974,13196,11250,14100,9707,11797 | ENSMUSG00000020037,"ENSMUSG00000020037",0,0,0,0,0,0,0,1,0,1,2,0,0,0,4,0,0,1,0,0 | ENSMUSG00000105637,"ENSMUSG00000105637",0,0,0,0,0,0,0,0,0,0,3,0,0,0,0,0,0,0,0,0 |
| ENSMUSG00000032294,"ENSMUSG00000032294",20685,32426,48821,41903,44369,41087,36371,42588,35472,40404,41303,31671,51547,45254,42491,51503,39192,57992,40029,48479 | ENSMUSG00000035916,"ENSMUSG00000035916",0,0,3,1,1,1,1,3,0,0,0,0,3,1,0,1,5,0,4,0 | ENSMUSG00000105050,"ENSMUSG00000105050",0,0,0,0,0,0,0,4,0,0,0,0,0,0,0,0,0,0,0,0 |
| ENSMUSG00000024610,"ENSMUSG00000024610",4955,8650,9262,10632,11307,11061,9398,10813,10124,11474,11501,8916,13528,11186,8962,13269,9900,17647,11469,11403 | ENSMUSG00000114554,"ENSMUSG00000114554",19,14,28,16,42,26,22,28,25,35,23,12,24,23,17,24,8,24,26,38 | ENSMUSG00000104709,"ENSMUSG00000104709",0,0,0,0,0,0,1,0,0,0,0,0,0,1,0,1,0,0,0,0 |
| ENSMUSG00000022824,"ENSMUSG00000022824",8,9,12,15,17,13,13,16,6,11,13,9,2,10,14,23,9,24,5,8 | ENSMUSG00000015879,"ENSMUSG00000015879",4,8,3,10,4,8,5,6,11,8,3,4,7,5,6,7,4,7,14,6 | ENSMUSG00000104961,"ENSMUSG00000104961",0,0,0,0,0,0,0,0,0,0,0,0,0,0,0,0,0,0,0,0 |
| ENSMUSG00000031328,"ENSMUSG00000031328",10373,13625,17042,16064,15326,16735,17066,13433,13754,15468,15006,11149,14564,17151,13933,16079,14686,18724,12172,15005 | ENSMUSG00000076488,"ENSMUSG00000076488",2,3,6,5,6,6,5,2,7,8,9,0,7,6,3,3,7,4,10,5 | ENSMUSG00000106785,"ENSMUSG00000106785",0,0,0,0,0,0,0,0,0,0,0,0,0,0,0,0,0,0,0,0 |
| ENSMUSG00000076609,"ENSMUSG00000076609",18,24,15,16,15,33,10,34,17,22,21,158,139,18,35,32,95,26,53,39 | ENSMUSG00000045009,"ENSMUSG00000045009",7,11,22,18,7,16,11,15,16,6,17,4,15,7,17,11,15,23,15,14 | ENSMUSG00000107206,"ENSMUSG00000107206",0,0,0,0,0,0,1,0,0,0,0,0,0,1,0,0,0,0,0,0 |
| ENSMUSG00000003814,"ENSMUSG00000003814",10363,14565,21016,20928,20055,16175,18422,18910,17410,16943,20844,15506,19898,19400,19598,20541,17196,21230,18434,21338 | ENSMUSG00000055216,"ENSMUSG00000055216",6,3,12,11,5,7,2,4,5,7,12,7,10,13,8,12,15,4,6,5 | ENSMUSG00000106136,"ENSMUSG00000106136",0,0,0,0,0,0,0,0,0,0,0,0,0,0,0,0,0,0,0,0 |
| ENSMUSG00000038393,"ENSMUSG00000038393",4774,5682,7295,6348,6127,6261,5524,6523,6631,7883,6226,4704,8153,7078,5519,6703,6454,7386,5136,7430 | ENSMUSG00000092626,"ENSMUSG00000092626",4,1,5,3,13,4,13,6,7,2,5,10,9,4,9,6,11,6,9,8 | ENSMUSG00000044058,"ENSMUSG00000044058",0,0,0,0,0,0,0,0,0,0,0,0,0,0,0,0,0,0,0,0 |
| ENSMUSG00000027187,"ENSMUSG00000027187",403,469,683,580,601,569,605,616,556,515,746,511,714,574,497,548,556,721,590,636 | ENSMUSG00000104116,"ENSMUSG00000104116",1,4,3,8,2,7,3,5,3,6,1,3,3,0,0,2,2,2,2,1 | ENSMUSG00000106860,"ENSMUSG00000106860",0,0,0,0,0,0,1,0,0,0,0,0,0,0,0,0,0,0,0,0 |
| ENSMUSG00000054889,"ENSMUSG00000054889",33253,40189,48705,61961,49609,37065,49721,36494,45243,40635,52404,50820,43778,43443,41954,51166,39658,53105,35169,44723 | ENSMUSG00000026904,"ENSMUSG00000026904",1,0,1,1,1,1,2,0,2,0,0,4,1,0,2,2,0,1,3,1 | ENSMUSG00000106576,"ENSMUSG00000106576",0,0,0,0,0,0,0,0,3,0,0,0,0,0,0,0,0,0,0,0 |
| ENSMUSG00000002831,"ENSMUSG00000002831",289,600,612,791,804,626,559,518,699,815,640,556,653,704,627,672,577,737,633,555 | ENSMUSG00000090667,"ENSMUSG00000090667",0,0,0,0,2,0,0,1,0,0,0,1,4,2,0,1,4,0,0,2 | ENSMUSG00000106507,"ENSMUSG00000106507",0,0,0,0,1,1,0,0,1,0,0,0,0,0,0,0,0,0,1,0 |
| ENSMUSG00000007892,"ENSMUSG00000007892",13216,17633,23003,20835,18332,17882,19155,14631,16682,19883,20777,17528,18718,18631,17508,21775,16188,23014,18892,19057 | ENSMUSG00000067702,"ENSMUSG00000067702",2,3,2,2,0,2,4,5,3,3,6,1,5,1,4,1,1,4,3,5 | ENSMUSG00000084500,"ENSMUSG00000084500",0,0,0,0,0,0,0,0,0,0,0,0,0,0,0,0,1,0,0,0 |
| ENSMUSG00000033831,"ENSMUSG00000033831",0,0,0,0,0,2,0,11,3,0,0,1,0,0,1,0,0,0,6,4 | ENSMUSG00000095913,"ENSMUSG00000095913",8,0,2,11,8,4,8,6,7,2,3,10,7,0,2,8,5,5,0,5 | ENSMUSG00000077175,"ENSMUSG00000077175",0,0,0,0,0,0,0,0,0,0,0,0,0,0,0,0,0,0,0,0 |
| ENSMUSG00000045394,"ENSMUSG00000045394",2355,3299,3674,4410,3782,3073,3737,2579,3356,2984,3393,3282,2406,3720,3131,3477,2735,3694,3125,3097 | ENSMUSG00000036449,"ENSMUSG00000036449",0,0,0,0,0,0,0,0,0,0,0,0,0,0,0,0,0,0,0,0 | ENSMUSG00000096044,"ENSMUSG00000096044",0,0,0,0,0,0,0,0,0,0,0,0,0,0,0,0,0,0,0,0 |
| ENSMUSG00000026473,"ENSMUSG00000026473",1840,2775,3899,3561,3289,3132,2788,3351,3212,3838,3466,2316,4001,3873,3428,3497,2983,3969,2841,3838 | ENSMUSG00000018862,"ENSMUSG00000018862",42,34,59,75,48,21,36,37,47,24,91,50,69,34,31,47,28,56,38,46 | ENSMUSG00000073497,"ENSMUSG00000073497",0,0,0,0,0,2,0,0,0,0,0,0,0,0,0,0,0,0,0,0 |
| ENSMUSG00000064339,"ENSMUSG00000064339",9957,8665,8027,8223,8789,7212,7443,5927,7056,8711,6664,5686,7155,6670,7092,8031,7577,7450,4650,8542 | ENSMUSG00000113780,"ENSMUSG00000113780",5,5,7,8,6,6,6,10,8,5,9,4,6,0,4,5,9,2,7,7 | ENSMUSG00000106610,"ENSMUSG00000106610",0,0,0,0,0,0,0,0,0,0,0,0,0,0,0,0,0,0,0,0 |
| ENSMUSG00000026728,"ENSMUSG00000026728",23342,20876,25878,29167,24970,26018,24591,22384,22946,29209,23319,15669,25006,28224,21990,24454,23933,27418,18272,24608 | ENSMUSG00000117226,"ENSMUSG00000117226",0,0,0,0,0,0,1,0,0,0,0,0,0,0,0,0,0,0,0,0 | ENSMUSG00000104808,"ENSMUSG00000104808",1,0,0,0,0,1,0,0,0,0,0,0,0,0,0,0,0,0,0,0 |
| ENSMUSG00000017707,"ENSMUSG00000017707",7483,7187,10055,8510,8620,8202,6962,7447,7686,8830,8932,6053,9534,8021,7369,8853,7682,10488,6607,7825 | ENSMUSG00000060989,"ENSMUSG00000060989",6,24,23,17,16,8,22,14,10,16,10,13,16,32,13,21,23,16,5,10 | ENSMUSG00000097237,"ENSMUSG00000097237",0,0,0,0,0,1,0,0,3,0,0,0,0,0,0,0,0,0,0,0 |
| ENSMUSG00000022443,"ENSMUSG00000022443",7934,15972,19384,19995,18178,17369,18812,15788,16445,17484,16482,13619,17015,19861,16583,20829,17383,21280,14213,17619 | ENSMUSG00000066755,"ENSMUSG00000066755",56,37,35,38,30,47,30,68,26,46,28,12,36,40,58,31,43,54,25,44 | ENSMUSG00000105508,"ENSMUSG00000105508",0,0,0,0,0,0,0,0,0,0,0,0,0,0,0,0,0,0,0,0 |
| ENSMUSG00000008682,"ENSMUSG00000008682",19969,14389,17803,17140,16131,15425,15802,12470,13921,16538,17160,14111,14756,15247,14278,17019,13734,17806,13403,15313 | ENSMUSG00000026567,"ENSMUSG00000026567",0,1,0,3,1,0,2,2,1,1,2,2,0,1,0,2,5,1,3,3 | ENSMUSG00000097018,"ENSMUSG00000097018",0,0,0,0,0,0,0,0,0,0,0,0,0,0,0,0,0,0,0,0 |
| ENSMUSG00000095079,"ENSMUSG00000095079",6,20,6,4,5,1,5,23,4,5,7,2,57,16,6,19,119,18,17,24 | ENSMUSG00000081400,"ENSMUSG00000081400",16,16,8,13,5,10,14,11,8,13,13,10,7,8,8,10,7,13,7,12 | ENSMUSG00000088429,"ENSMUSG00000088429",0,0,0,0,0,0,0,0,0,0,0,0,0,0,0,0,0,0,0,0 |
| ENSMUSG00000064368,"ENSMUSG00000064368",13716,7252,7778,7467,9683,6226,6996,5537,6506,7165,7921,6213,7090,5689,6225,6199,6472,8252,4892,7075 | ENSMUSG00000087017,"ENSMUSG00000087017",5,3,5,5,4,0,1,0,1,4,3,7,2,2,2,3,0,8,3,0 | ENSMUSG00000105188,"ENSMUSG00000105188",0,0,0,0,0,0,0,0,0,0,0,0,0,0,0,0,0,0,0,0 |
| ENSMUSG00000050708,"ENSMUSG00000050708",6497,8649,13891,8318,11463,11923,9683,11671,9275,10711,10221,6108,14592,10102,10481,11351,10346,13286,11146,12810 | ENSMUSG00000113303,"ENSMUSG00000113303",24,28,28,50,29,11,39,27,26,18,55,38,36,30,36,38,20,31,28,23 | ENSMUSG00000105591,"ENSMUSG00000105591",0,0,0,0,0,0,0,0,0,0,0,0,0,0,0,0,0,0,0,0 |
| ENSMUSG00000020315,"ENSMUSG00000020315",3834,4408,5597,5466,5102,5294,4724,4325,4488,5430,5181,3714,5545,5124,4582,5149,4687,6047,3400,4510 | ENSMUSG00000103358,"ENSMUSG00000103358",8,4,11,5,6,7,4,9,2,3,3,5,13,9,8,5,8,5,2,3 | ENSMUSG00000090688,"ENSMUSG00000090688",0,0,0,0,0,2,0,0,0,0,0,0,0,0,0,0,0,0,0,0 |
| ENSMUSG00000030744,"ENSMUSG00000030744",13035,13476,18484,16096,14417,14140,14197,11101,12627,15433,15965,13020,14692,14313,13309,15971,12473,17226,12727,14892 | ENSMUSG00000114278,"ENSMUSG00000114278",2,1,4,3,3,0,1,1,2,1,2,1,3,1,5,0,1,1,1,0 | ENSMUSG00000077254,"ENSMUSG00000077254",0,0,0,0,0,0,0,0,0,0,0,0,0,0,0,0,0,0,0,0 |
| ENSMUSG00000031996,"ENSMUSG00000031996",6843,7831,10618,9096,11138,8339,9346,8008,8634,8660,8749,7545,8312,9431,8702,9919,8286,11101,7251,9579 | ENSMUSG00000078640,"ENSMUSG00000078640",2,1,0,2,0,0,1,3,0,0,0,1,0,2,0,0,2,1,1,1 | ENSMUSG00000096238,"ENSMUSG00000096238",0,0,0,0,0,0,0,0,0,0,0,0,0,0,0,0,0,0,0,0 |
| ENSMUSG00000055322,"ENSMUSG00000055322",2183,3216,4697,4569,4098,3393,3070,3090,3452,3873,4616,3328,4267,3813,3409,3914,3588,4213,2710,3292 | ENSMUSG00000026246,"ENSMUSG00000026246",0,0,0,0,0,2,0,0,0,0,0,0,0,0,0,0,0,0,0,0 | ENSMUSG00000085919,"ENSMUSG00000085919",0,0,1,0,2,0,0,0,0,0,0,0,0,0,0,0,0,0,0,0 |
| ENSMUSG00000092341,"ENSMUSG00000092341",8830,10812,14483,11342,12374,13177,9760,10270,10408,16503,16723,10526,14747,12147,12993,13715,12593,17081,8212,12444 | ENSMUSG00000005883,"ENSMUSG00000005883",7,1,1,2,1,1,1,4,0,1,4,0,5,0,1,3,2,3,4,0 | ENSMUSG00000104538,"ENSMUSG00000104538",0,0,0,0,0,0,0,0,0,0,0,0,0,0,0,0,0,0,0,0 |
| ENSMUSG00000029455,"ENSMUSG00000029455",395,1175,1749,1538,1404,1502,1291,1213,1277,1510,1489,987,1655,1439,1316,1394,1335,1719,1410,1490 | ENSMUSG00000090457,"ENSMUSG00000090457",0,0,0,0,0,0,0,0,0,0,0,0,0,0,0,0,0,0,0,0 | ENSMUSG00000105735,"ENSMUSG00000105735",0,0,0,0,0,0,0,0,0,0,0,0,0,0,0,0,0,0,0,0 |
| ENSMUSG00000020372,"ENSMUSG00000020372",14161,14856,17891,17325,15936,15526,15927,11769,14366,16151,16638,14409,14313,14935,13752,17308,13807,17820,14709,14589 | ENSMUSG00000053214,"ENSMUSG00000053214",0,0,0,0,1,1,1,0,1,0,2,0,0,0,0,0,0,0,1,0 | ENSMUSG00000042002,"ENSMUSG00000042002",0,0,1,0,0,0,0,0,0,0,0,0,0,0,0,0,0,0,0,0 |
| ENSMUSG00000056071,"ENSMUSG00000056071",25852,48278,52193,36486,47106,50637,51201,42726,44439,60419,39544,33945,49506,55420,45873,63700,44760,61776,44640,57172 | ENSMUSG00000030491,"ENSMUSG00000030491",0,0,0,2,0,0,0,0,0,0,1,2,0,0,0,0,4,2,1,0 | ENSMUSG00000106829,"ENSMUSG00000106829",0,0,0,0,0,0,0,0,0,0,0,0,0,0,0,0,0,0,0,0 |
| ENSMUSG00000054999,"ENSMUSG00000054999",6,12,8,9,11,5,12,7,10,6,10,18,13,9,22,10,14,7,24,16 | ENSMUSG00000090459,"ENSMUSG00000090459",22,21,17,7,11,19,13,11,10,10,12,12,15,8,15,16,9,7,8,5 | ENSMUSG00000093041,"ENSMUSG00000093041",0,0,1,0,0,0,0,0,0,0,0,0,0,0,0,0,0,0,0,0 |
| ENSMUSG00000022650,"ENSMUSG00000022650",0,6,1,0,0,1,0,0,0,0,0,0,0,2,0,0,0,1,1,2 | ENSMUSG00000018925,"ENSMUSG00000018925",0,8,4,7,1,8,5,2,2,8,5,3,6,8,11,6,4,9,13,7 | ENSMUSG00000105583,"ENSMUSG00000105583",0,0,0,0,0,0,0,0,0,0,0,0,0,0,0,0,0,0,0,0 |
| ENSMUSG00000061527,"ENSMUSG00000061527",30269,58996,78203,90168,66518,56189,64476,55014,57570,59424,80126,71961,68257,60058,59642,77966,49976,79861,77274,68537 | ENSMUSG00000076137,"ENSMUSG00000076137",2,10,21,15,7,9,8,5,8,15,9,15,9,16,3,11,8,13,3,12 | ENSMUSG00000106271,"ENSMUSG00000106271",0,0,0,0,0,0,0,0,0,0,0,0,0,0,0,0,0,0,0,0 |
| ENSMUSG00000106106,"ENSMUSG00000106106",4292,9367,13991,13687,11983,13536,10444,7192,7462,13067,27876,8339,11714,11780,8767,11552,10016,15061,5202,8978 | ENSMUSG00000082431,"ENSMUSG00000082431",3,4,5,8,3,4,5,11,8,6,8,4,2,1,5,5,11,6,1,4 | ENSMUSG00000105673,"ENSMUSG00000105673",0,0,0,0,0,0,0,0,0,0,0,0,0,0,0,0,0,0,0,0 |
| ENSMUSG00000022283,"ENSMUSG00000022283",12976,15072,16312,17032,16531,15985,16605,10759,14762,16123,15091,13171,11403,14582,13586,16352,13790,16345,9219,12523 | ENSMUSG00000105395,"ENSMUSG00000105395",3,9,10,13,18,11,8,12,5,7,12,8,15,13,13,6,10,8,3,10 | ENSMUSG00000106235,"ENSMUSG00000106235",0,0,0,0,0,0,0,0,0,0,0,0,0,0,0,0,0,0,0,0 |
| ENSMUSG00000028307,"ENSMUSG00000028307",0,2,0,0,0,0,1,2,5,0,0,0,0,0,0,0,0,0,0,9 | ENSMUSG00000025892,"ENSMUSG00000025892",0,4,3,2,3,1,2,1,0,3,2,2,1,1,1,2,0,4,0,2 | ENSMUSG00000104727,"ENSMUSG00000104727",1,0,0,1,0,0,0,0,0,0,0,0,0,0,0,0,0,0,0,0 |
| ENSMUSG00000043716,"ENSMUSG00000043716",13514,13562,15693,15228,14862,14206,14023,10659,12718,14479,14995,12370,12645,12698,12253,14932,12230,15393,10524,13937 | ENSMUSG00000086481,"ENSMUSG00000086481",9,9,7,8,2,5,2,2,3,4,3,5,14,4,2,13,7,1,8,10 | ENSMUSG00000106522,"ENSMUSG00000106522",0,0,0,0,0,0,0,0,0,0,0,0,0,0,0,1,0,0,0,0 |
| ENSMUSG00000004980,"ENSMUSG00000004980",10895,10310,13056,13308,12177,11373,11023,10171,10286,11649,13231,9532,11513,11979,11276,12102,10582,13520,9757,11583 | ENSMUSG00000109459,"ENSMUSG00000109459",1,6,2,1,0,4,11,1,1,1,9,4,5,4,0,1,0,8,4,0 | ENSMUSG00000106570,"ENSMUSG00000106570",0,0,0,0,0,0,0,0,0,0,0,0,0,0,0,0,0,0,0,0 |
| ENSMUSG00000074445,"ENSMUSG00000074445",35378,47518,56768,61693,60493,47125,40106,44357,44577,45556,56535,53784,47013,45577,50734,65586,45413,65937,68212,49767 | ENSMUSG00000073154,"ENSMUSG00000073154",0,0,0,0,0,0,0,0,0,1,0,0,0,2,0,0,0,0,0,0 | ENSMUSG00000106356,"ENSMUSG00000106356",0,0,0,0,0,0,0,0,0,0,0,0,0,0,0,0,0,0,0,1 |
| ENSMUSG00000058354,"ENSMUSG00000058354",67827,61423,61124,61497,70980,49248,66246,49789,62887,61262,50277,63377,66099,61970,53701,80092,63455,78307,51270,83936 | ENSMUSG00000114689,"ENSMUSG00000114689",17,19,31,15,30,26,27,13,33,34,18,13,34,25,17,36,45,32,10,23 | ENSMUSG00000106384,"ENSMUSG00000106384",0,0,0,0,0,0,0,0,0,0,0,0,0,0,0,0,0,0,0,0 |
| ENSMUSG00000028081,"ENSMUSG00000028081",10689,11447,14152,13685,12258,12076,12424,8992,10983,13054,13744,11307,10826,11893,10662,12807,10126,13938,10267,11303 | ENSMUSG00000099950,"ENSMUSG00000099950",0,0,0,0,1,0,0,0,1,0,0,0,0,0,0,0,0,0,0,0 | ENSMUSG00000106323,"ENSMUSG00000106323",0,0,0,0,0,0,0,0,0,0,0,0,0,1,0,0,0,0,0,0 |
| ENSMUSG00000031320,"ENSMUSG00000031320",14603,14269,16507,16497,15528,14796,14555,10911,13416,15672,16417,13464,12787,13591,12765,15637,12821,16693,12221,14026 | ENSMUSG00000041735,"ENSMUSG00000041735",15,18,16,39,24,28,69,14,31,26,18,34,22,31,15,20,23,44,31,22 | ENSMUSG00000087244,"ENSMUSG00000087244",0,0,0,0,0,1,0,0,0,0,0,0,0,0,0,0,0,0,0,0 |
| ENSMUSG00000039218,"ENSMUSG00000039218",7220,9772,11295,11524,11767,10760,10855,8993,10836,10821,12460,9238,10899,11167,9821,11674,10074,12384,8599,9433 | ENSMUSG00000094525,"ENSMUSG00000094525",0,3,7,5,8,7,2,6,5,4,8,6,11,1,2,4,4,0,5,1 | ENSMUSG00000029711,"ENSMUSG00000029711",0,0,0,0,0,0,0,0,0,0,0,0,0,0,0,0,0,0,0,0 |

| ENSMUSG00000071866,"ENSMUSG00000071866",15470,12182,15524,13761,13877,13539,13463,10749,12291,14010,14766,12043,12942,13021,11908,14313,10960,14421,12729,12619 | ENSMUSG00000076471,"ENSMUSG00000076471",15,8,4,2,7,12,2,5,1,6,11,9,7,3,5,12,3,22,2,11 | ENSMUSG00000079165,"ENSMUSG00000079165",0,0,0,0,0,0,0,0,0,0,1,0,0,0,0,0,0,2,0,0 |
| --- | --- | --- |
| ENSMUSG00000018796,"ENSMUSG00000018796",2120,2411,2597,3638,3127,2221,2856,2389,2876,2470,3247,3068,2614,2750,2693,3168,2533,3167,2614,2580 | ENSMUSG00000114934,"ENSMUSG00000114934",2,1,0,8,14,6,4,1,5,4,7,6,3,4,7,4,1,4,7,6 | ENSMUSG00000103298,"ENSMUSG00000103298",0,0,0,0,0,0,0,0,0,0,0,0,0,0,0,0,0,0,0,0 |
| ENSMUSG00000020777,"ENSMUSG00000020777",1627,2071,2682,2917,2512,2103,2533,2187,2262,2402,2801,2120,2451,2568,2330,2622,2110,2589,2260,2336 | ENSMUSG00000117037,"ENSMUSG00000117037",8,3,2,3,6,7,7,3,10,10,5,11,5,1,6,7,4,6,3,8 | ENSMUSG00000029546,"ENSMUSG00000029546",0,0,0,0,0,0,0,0,0,0,0,0,0,0,0,0,0,0,0,0 |
| ENSMUSG00000054808,"ENSMUSG00000054808",5489,7817,10131,10786,9286,8425,9616,8104,8712,8513,9560,8024,9076,9296,8796,10254,8047,11054,9693,9676 | ENSMUSG00000085196,"ENSMUSG00000085196",3,3,6,3,5,7,6,5,4,11,5,2,10,2,8,8,4,10,2,2 | ENSMUSG00000105335,"ENSMUSG00000105335",0,0,0,0,1,0,1,1,0,0,0,0,0,0,0,0,0,0,0,0 |
| ENSMUSG00000000740,"ENSMUSG00000000740",6568,13779,18209,16530,14899,14486,14896,11357,12395,14816,16936,13629,14551,14740,13726,16682,12866,17767,13887,14807 | ENSMUSG00000110221,"ENSMUSG00000110221",0,0,7,3,5,1,3,0,1,1,1,3,0,1,1,1,0,0,2,4 | ENSMUSG00000105024,"ENSMUSG00000105024",0,0,0,0,0,0,0,0,0,0,0,0,0,0,0,2,0,1,0,0 |
| ENSMUSG00000022878,"ENSMUSG00000022878",76,90,114,122,169,189,95,95,84,144,72,74,120,99,115,92,113,117,95,112 | ENSMUSG00000042529,"ENSMUSG00000042529",20,27,35,28,33,14,23,17,20,34,24,25,43,28,24,39,20,37,11,34 | ENSMUSG00000079091,"ENSMUSG00000079091",0,0,0,0,0,0,0,0,0,0,0,0,0,0,0,0,0,0,0,0 |
| ENSMUSG00000031722,"ENSMUSG00000031722",2873,3141,4307,3488,4140,5259,3025,3276,2886,4928,3030,2159,4728,4588,3688,4251,3531,4639,3262,4551 | ENSMUSG00000096940,"ENSMUSG00000096940",7,11,13,5,7,6,4,8,5,5,14,4,10,6,3,6,6,9,5,9 | ENSMUSG00000106738,"ENSMUSG00000106738",0,0,0,1,0,0,0,0,0,0,0,0,0,0,0,0,0,0,0,1 |
| ENSMUSG00000005681,"ENSMUSG00000005681",7,29,9,12,13,36,26,33,31,29,27,18,33,26,19,26,15,18,19,39 | ENSMUSG00000068466,"ENSMUSG00000068466",7,7,16,17,11,8,12,12,18,7,15,8,13,13,11,9,11,14,13,13 | ENSMUSG00000056586,"ENSMUSG00000056586",0,0,0,0,0,0,0,0,0,0,0,0,0,0,0,0,0,1,0,0 |
| ENSMUSG00000061983,"ENSMUSG00000061983",15794,15420,17498,16929,15346,14752,15756,11505,14343,16322,17067,14146,13942,14558,13173,17148,13068,17496,13344,15307 | ENSMUSG00000076486,"ENSMUSG00000076486",1,0,5,12,3,7,3,3,9,3,13,4,5,10,11,12,3,10,2,2 | ENSMUSG00000107241,"ENSMUSG00000107241",0,0,0,0,0,0,0,0,0,0,0,0,0,0,0,0,0,0,0,0 |
| ENSMUSG00000093674,"ENSMUSG00000093674",6702,14051,19704,17064,13472,14568,13989,12405,13955,17617,18660,14273,15967,16676,14004,19106,14028,20682,13212,17322 | ENSMUSG00000097974,"ENSMUSG00000097974",1,7,1,6,13,6,4,1,6,8,6,7,10,4,9,6,4,8,6,8 | ENSMUSG00000077250,"ENSMUSG00000077250",0,0,0,0,0,0,0,0,0,0,0,0,0,0,0,0,0,0,0,0 |
| ENSMUSG00000006932,"ENSMUSG00000006932",10739,10132,13422,12833,12915,12586,11590,10220,10441,12661,12811,9407,12868,12376,11230,12930,10962,14019,10040,11699 | ENSMUSG00000111547,"ENSMUSG00000111547",11,7,8,4,7,2,2,6,4,0,4,7,1,3,7,3,1,3,4,3 | ENSMUSG00000106855,"ENSMUSG00000106855",0,0,0,0,0,0,0,0,0,0,0,0,0,0,0,0,0,0,0,0 |
| ENSMUSG00000056201,"ENSMUSG00000056201",9395,14384,21164,18818,16475,16705,15587,15232,14634,17273,18244,13121,18261,18280,16485,18933,16057,21048,16190,18288 | ENSMUSG00000116620,"ENSMUSG00000116620",0,7,10,5,9,8,8,7,8,9,9,21,12,14,7,14,7,7,3,11 | ENSMUSG00000098187,"ENSMUSG00000098187",0,1,0,0,0,0,0,0,0,0,0,0,0,0,0,1,0,0,0,0 |
| ENSMUSG00000037390,"ENSMUSG00000037390",0,0,0,0,0,0,1,0,0,2,4,0,0,0,0,1,1,0,0,0 | ENSMUSG00000114936,"ENSMUSG00000114936",38,50,33,61,39,29,58,32,66,44,45,47,50,37,58,49,50,71,25,32 | ENSMUSG00000106803,"ENSMUSG00000106803",1,0,0,0,0,0,1,0,0,0,0,0,0,0,0,0,0,0,0,0 |
| ENSMUSG00000016319,"ENSMUSG00000016319",6430,8079,10566,9966,9163,8682,8706,7215,8174,9050,10444,8021,8298,9210,8341,9536,8040,10499,8614,8546 | ENSMUSG00000027168,"ENSMUSG00000027168",1,0,6,1,0,2,4,1,2,1,4,1,0,2,1,0,3,2,5,1 | ENSMUSG00000106836,"ENSMUSG00000106836",0,0,0,0,1,0,0,0,0,0,0,0,3,0,0,0,0,0,0,0 |
| ENSMUSG00000045983,"ENSMUSG00000045983",6813,10678,13777,14498,12283,11444,13139,9620,11371,11695,13441,10907,11013,12434,10994,13015,10884,14612,10572,11421 | ENSMUSG00000031104,"ENSMUSG00000031104",2,3,6,11,9,4,1,4,4,3,2,3,16,2,3,4,1,11,10,11 | ENSMUSG00000077339,"ENSMUSG00000077339",0,0,0,0,0,0,0,0,0,0,0,0,0,0,0,0,0,0,0,0 |
| ENSMUSG00000060036,"ENSMUSG00000060036",11939,9250,11420,11403,9958,10129,9973,7784,9024,9920,11163,8566,9411,9573,9115,11109,8680,11352,7783,9784 | ENSMUSG00000018634,"ENSMUSG00000018634",2,0,0,3,1,1,0,0,0,1,2,3,0,0,0,1,0,0,0,0 | ENSMUSG00000102221,"ENSMUSG00000102221",0,0,0,0,0,0,0,0,0,0,0,0,0,0,0,0,0,0,0,0 |
| ENSMUSG00000034088,"ENSMUSG00000034088",7273,9553,12602,12246,12309,11648,11264,9379,10041,10647,12114,9221,11258,11039,10609,12223,9927,12802,8615,10588 | ENSMUSG00000087266,"ENSMUSG00000087266",0,3,2,2,1,7,5,3,4,6,5,2,4,0,0,5,3,4,1,9 | ENSMUSG00000097255,"ENSMUSG00000097255",0,0,1,0,0,0,0,0,0,0,0,1,0,0,0,1,0,0,0,0 |
| ENSMUSG00000047126,"ENSMUSG00000047126",21457,16080,20606,18929,20959,19379,16052,15152,16318,19068,18627,14070,17583,17753,17256,18977,17850,19484,11819,16264 | ENSMUSG00000054136,"ENSMUSG00000054136",2,13,47,35,52,39,19,52,32,13,18,46,60,32,36,98,26,72,61,54 | ENSMUSG00000108275,"ENSMUSG00000108275",0,0,0,0,0,0,0,0,0,0,0,0,0,0,0,0,0,0,0,0 |
| ENSMUSG00000029614,"ENSMUSG00000029614",10444,12640,15417,15026,14046,13460,13480,10245,11636,13166,14092,11442,12363,12476,12087,14245,11482,15264,11573,12729 | ENSMUSG00000071202,"ENSMUSG00000071202",2,7,11,5,2,7,0,5,2,4,8,7,4,7,8,16,3,5,4,3 | ENSMUSG00000094528,"ENSMUSG00000094528",0,0,0,0,0,0,0,0,0,0,0,0,0,0,0,0,1,0,0,0 |
| ENSMUSG00000033860,"ENSMUSG00000033860",41,24,62,43,21,19,14,27,37,28,20,12,62,24,37,50,53,32,36,55 | ENSMUSG00000104367,"ENSMUSG00000104367",3,8,9,11,8,20,15,10,5,14,9,6,9,22,8,7,19,16,3,15 | ENSMUSG00000080759,"ENSMUSG00000080759",0,0,0,0,0,0,0,0,0,0,0,0,0,0,0,0,0,0,0,0 |
| ENSMUSG00000026712,"ENSMUSG00000026712",43770,41562,65078,51645,56582,58695,41997,38562,41921,55433,48383,31224,47783,54403,45135,44152,49003,40848,29310,38837 | ENSMUSG00000038528,"ENSMUSG00000038528",5,3,5,7,10,8,2,1,2,6,9,6,7,8,8,3,15,4,9,4 | ENSMUSG00000107449,"ENSMUSG00000107449",0,0,0,0,0,0,0,0,0,0,0,0,0,0,0,0,0,4,0,0 |
| ENSMUSG00000026175,"ENSMUSG00000026175",4,1,1,5,2,3,0,1,2,2,5,0,2,4,0,2,2,2,3,5 | ENSMUSG00000110706,"ENSMUSG00000110706",5,9,12,7,4,4,8,3,4,6,7,3,7,10,5,4,4,4,5,1 | ENSMUSG00000107596,"ENSMUSG00000107596",0,0,0,1,0,0,0,0,0,0,0,0,0,0,0,0,0,0,0,0 |
| ENSMUSG00000032216,"ENSMUSG00000032216",4394,4002,5460,4914,4869,5138,4633,4763,4509,4183,5789,3661,5956,4141,4567,4513,4294,5095,4268,4974 | ENSMUSG00000044081,"ENSMUSG00000044081",0,1,1,6,3,2,3,1,3,3,4,1,0,4,1,0,1,0,2,0 | ENSMUSG00000108000,"ENSMUSG00000108000",0,0,0,1,0,0,1,0,0,0,0,0,0,2,0,0,0,0,0,0 |
| ENSMUSG00000030546,"ENSMUSG00000030546",51,105,93,58,117,110,98,103,80,120,86,103,170,110,114,55,109,119,121,131 | ENSMUSG00000098259,"ENSMUSG00000098259",2,11,5,8,8,8,6,8,6,12,7,7,8,11,8,2,5,5,9,11 | ENSMUSG00000106124,"ENSMUSG00000106124",0,0,0,0,0,0,0,0,0,0,0,0,0,0,0,0,0,0,0,0 |
| ENSMUSG00000000303,"ENSMUSG00000000303",13422,17218,17198,20920,20291,15887,20527,13575,18715,17515,16444,17928,13131,18772,16198,20050,16543,21000,13553,17070 | ENSMUSG00000083240,"ENSMUSG00000083240",3,5,4,6,0,3,7,2,7,3,3,5,5,4,4,8,7,3,0,5 | ENSMUSG00000105989,"ENSMUSG00000105989",0,0,0,0,0,0,0,0,0,0,0,0,0,0,0,0,0,0,0,0 |
| ENSMUSG00000003970,"ENSMUSG00000003970",8938,12391,16300,14854,12994,12980,13709,10069,11665,12678,14110,11960,12738,13262,12156,14582,11366,15808,14039,13885 | ENSMUSG00000086233,"ENSMUSG00000086233",9,0,7,5,4,7,8,3,2,3,5,5,3,0,5,5,1,12,5,8 | ENSMUSG00000105110,"ENSMUSG00000105110",0,0,0,0,0,0,0,0,0,0,0,0,0,0,0,0,0,0,0,0 |
| ENSMUSG00000056054,"ENSMUSG00000056054",45715,33678,35056,27127,33209,35762,39259,33336,32164,44879,28902,24348,34288,38360,32388,38562,33947,51157,26673,39478 | ENSMUSG00000101678,"ENSMUSG00000101678",8,8,9,4,6,10,10,11,11,7,5,10,10,6,5,7,5,6,1,9 | ENSMUSG00000106428,"ENSMUSG00000106428",0,0,0,0,0,0,0,0,0,0,0,0,0,0,0,0,0,0,0,0 |
| ENSMUSG00000027248,"ENSMUSG00000027248",8093,8641,12804,12933,12304,11056,11027,10857,10753,10652,13276,9538,12065,11194,11559,11796,10460,13026,9893,12472 | ENSMUSG00000115098,"ENSMUSG00000115098",14,18,17,18,13,10,23,13,18,17,16,13,7,20,23,13,19,13,10,13 | ENSMUSG00000108088,"ENSMUSG00000108088",0,0,0,0,0,0,0,0,0,0,0,0,0,0,0,0,0,0,0,0 |
| ENSMUSG00000031447,"ENSMUSG00000031447",9114,8074,11937,10149,10049,9669,8565,7857,8724,9563,9980,7259,9699,8920,8275,9651,8581,10723,8484,8791 | ENSMUSG00000097159,"ENSMUSG00000097159",12,5,8,11,14,11,6,7,5,3,11,6,9,7,2,11,8,5,3,5 | ENSMUSG00000101404,"ENSMUSG00000101404",0,0,0,0,0,0,0,0,0,0,0,0,0,0,0,0,0,0,0,0 |
| ENSMUSG00000033685,"ENSMUSG00000033685",5784,7580,10191,8265,9114,8815,7056,8379,7967,10589,8973,6358,9294,9660,7938,10499,8632,11966,6993,8781 | ENSMUSG00000007908,"ENSMUSG00000007908",11,8,11,7,5,7,3,6,4,7,9,6,14,5,8,5,9,7,12,10 | ENSMUSG00000068232,"ENSMUSG00000068232",0,0,0,0,0,0,0,2,0,0,0,0,0,0,0,0,0,0,0,0 |
| ENSMUSG00000035775,"ENSMUSG00000035775",0,4,8,8,9,6,4,2,7,3,9,2,5,2,5,1,4,6,6,4 | ENSMUSG00000085664,"ENSMUSG00000085664",4,9,8,6,14,14,12,11,10,13,11,7,10,7,18,13,19,11,6,6 | ENSMUSG00000108082,"ENSMUSG00000108082",0,0,2,0,0,0,0,0,0,1,0,0,0,0,0,0,0,0,0,1 |
| ENSMUSG00000007655,"ENSMUSG00000007655",1808,1783,2320,2189,2006,2374,1934,1615,1783,2318,2219,1448,2161,1703,1719,1886,1906,2290,1545,2051 | ENSMUSG00000113841,"ENSMUSG00000113841",10,5,7,7,13,6,10,10,7,13,13,9,12,8,5,9,3,9,2,2 | ENSMUSG00000107713,"ENSMUSG00000107713",0,0,0,0,0,0,0,0,0,0,0,0,0,0,1,0,0,0,0,0 |
| ENSMUSG00000069515,"ENSMUSG00000069515",24,16,17,25,23,21,19,11,16,25,16,27,20,29,14,17,18,40,25,24 | ENSMUSG00000085204,"ENSMUSG00000085204",12,4,11,7,2,6,9,4,4,5,8,6,9,2,1,4,2,6,4,12 | ENSMUSG00000107204,"ENSMUSG00000107204",0,0,0,0,0,0,0,0,0,0,0,0,0,0,0,0,0,0,0,2 |
| ENSMUSG00000055065,"ENSMUSG00000055065",6297,7250,9556,9084,9172,8900,8651,7558,7864,8324,9821,6515,9300,8941,8229,9095,8283,10704,7502,7983 | ENSMUSG00000115200,"ENSMUSG00000115200",0,0,0,0,0,0,0,0,0,0,0,0,0,0,0,0,0,1,0,0 | ENSMUSG00000084157,"ENSMUSG00000084157",0,0,0,0,0,0,0,0,0,0,0,0,0,0,0,0,0,0,0,0 |
| ENSMUSG00000027875,"ENSMUSG00000027875",7,63,82,47,58,49,35,93,52,53,92,54,66,49,47,39,51,39,49,49 | ENSMUSG00000104324,"ENSMUSG00000104324",22,17,10,20,14,12,6,6,5,11,18,11,10,7,13,12,7,11,8,5 | ENSMUSG00000108272,"ENSMUSG00000108272",0,0,0,0,0,2,2,0,0,0,0,0,0,0,0,0,0,0,0,0 |
| ENSMUSG00000045128,"ENSMUSG00000045128",10885,9542,12471,11353,11240,10795,10834,8691,9703,11268,11282,9491,10403,10025,9125,10996,9073,12909,11085,10369 | ENSMUSG00000028840,"ENSMUSG00000028840",8,10,20,28,14,20,21,6,12,24,14,22,6,21,11,16,15,7,9,10 | ENSMUSG00000053251,"ENSMUSG00000053251",0,0,0,0,0,0,0,0,0,1,0,0,0,0,0,0,0,0,0,0 |
| ENSMUSG00000016559,"ENSMUSG00000016559",12775,16543,17302,20663,17287,15787,16978,14774,18617,18448,16791,15542,15459,18525,15255,19042,15534,19722,14383,17722 | ENSMUSG00000100642,"ENSMUSG00000100642",6,7,9,6,10,4,8,9,4,5,7,5,8,6,3,12,13,11,3,4 | ENSMUSG00000107903,"ENSMUSG00000107903",0,0,0,0,0,0,0,0,0,0,0,0,0,0,0,0,0,0,0,0 |
| ENSMUSG00000027533,"ENSMUSG00000027533",24429,34929,49544,49390,49743,42454,43391,42102,40921,41552,50597,42178,46490,41954,45147,53922,36495,56091,53713,48752 | ENSMUSG00000043621,"ENSMUSG00000043621",0,6,7,4,1,2,4,3,0,3,4,3,0,2,2,4,1,3,1,3 | ENSMUSG00000107933,"ENSMUSG00000107933",0,0,0,0,0,0,0,0,0,0,0,0,0,0,0,0,0,0,0,0 |
| ENSMUSG00000008540,"ENSMUSG00000008540",1062,1239,1371,1376,1350,1443,1450,1246,1306,1467,1190,968,1262,1287,1048,1411,1142,1693,1076,1304 | ENSMUSG00000106832,"ENSMUSG00000106832",35,43,32,37,59,44,26,50,20,59,43,20,46,59,43,41,46,44,19,32 | ENSMUSG00000076217,"ENSMUSG00000076217",0,0,0,0,0,0,0,0,0,0,0,0,0,0,0,1,0,0,0,0 |
| ENSMUSG00000052397,"ENSMUSG00000052397",5713,9253,10088,11592,9617,7625,10694,7050,9625,11642,9076,8251,7233,11031,9070,10675,8347,11133,7790,9173 | ENSMUSG00000078931,"ENSMUSG00000078931",17,10,11,9,6,7,11,6,3,6,5,8,9,7,8,6,5,10,8,10 | ENSMUSG00000108183,"ENSMUSG00000108183",0,0,0,0,0,0,0,0,0,0,0,0,0,0,0,0,0,0,0,0 |
| ENSMUSG00000069516,"ENSMUSG00000069516",12850,14653,16257,13887,12818,12570,11583,10723,9995,15826,13243,10295,14283,13767,11688,13627,12859,18120,13737,13179 | ENSMUSG00000041078,"ENSMUSG00000041078",4,0,6,5,7,12,6,7,1,2,1,0,11,3,11,8,0,5,5,3 | ENSMUSG00000107389,"ENSMUSG00000107389",0,0,0,0,0,0,0,0,0,0,0,0,0,0,0,0,0,0,0,0 |
| ENSMUSG00000033022,"ENSMUSG00000033022",96,94,188,90,114,108,112,136,103,155,119,65,228,143,123,124,119,157,101,118 | ENSMUSG00000110774,"ENSMUSG00000110774",15,22,23,19,20,14,19,19,19,27,18,6,27,31,19,22,20,33,4,12 | ENSMUSG00000107379,"ENSMUSG00000107379",0,0,0,0,0,0,1,0,0,0,0,0,0,0,1,0,0,0,0,1 |
| ENSMUSG00000020048,"ENSMUSG00000020048",6359,6291,9658,9447,9031,8097,8570,8837,7293,7449,9490,6574,9169,8444,8902,8293,7669,9409,7059,8901 | ENSMUSG00000100053,"ENSMUSG00000100053",6,11,4,4,7,11,5,12,6,7,10,4,15,11,4,4,8,6,3,16 | ENSMUSG00000107832,"ENSMUSG00000107832",0,0,0,0,0,0,0,0,0,0,0,1,0,0,0,0,0,0,0,0 |
| ENSMUSG00000047139,"ENSMUSG00000047139",7668,8346,10412,9566,10875,8692,8544,8875,9417,10577,9847,9585,8647,9230,9532,11516,8375,11149,8696,10624 | ENSMUSG00000103973,"ENSMUSG00000103973",7,8,10,6,16,12,4,13,4,14,10,8,13,12,7,12,6,16,10,3 | ENSMUSG00000108106,"ENSMUSG00000108106",0,0,0,0,0,0,0,0,0,0,0,0,0,0,0,0,0,0,0,0 |
| ENSMUSG00000018293,"ENSMUSG00000018293",4758,10395,15964,12751,12399,12705,11651,11488,10637,12863,13594,9350,14574,13492,12557,14022,12106,16056,12260,14075 | ENSMUSG00000101845,"ENSMUSG00000101845",13,23,15,15,12,7,15,15,18,21,18,24,12,8,10,7,16,12,7,14 | ENSMUSG00000108179,"ENSMUSG00000108179",0,0,0,1,0,0,0,0,0,0,1,0,1,0,0,0,1,0,0,0 |
| ENSMUSG00000030895,"ENSMUSG00000030895",13,19,55,27,37,72,14,20,25,22,27,19,32,26,10,16,34,24,20,28 | ENSMUSG00000110830,"ENSMUSG00000110830",6,6,8,2,2,6,7,4,8,5,3,1,4,3,1,6,2,1,2,5 | ENSMUSG00000107569,"ENSMUSG00000107569",0,0,0,0,0,0,0,2,0,2,0,0,0,0,0,0,0,0,0,0 |
| ENSMUSG00000061904,"ENSMUSG00000061904",8362,7982,9449,8827,9094,8687,9055,7152,8586,9096,9352,7290,7798,8434,7799,9155,7642,9681,7380,8712 | ENSMUSG00000074213,"ENSMUSG00000074213",5,11,9,9,20,24,4,34,19,11,7,10,12,20,12,24,12,20,13,15 | ENSMUSG00000107870,"ENSMUSG00000107870",0,0,0,0,0,0,0,0,0,0,0,0,0,0,0,0,0,0,0,0 |
| ENSMUSG00000026531,"ENSMUSG00000026531",0,0,0,0,0,0,0,0,0,0,0,0,0,0,0,0,0,0,0,0 | ENSMUSG00000092300,"ENSMUSG00000092300",9,5,6,11,9,9,2,2,12,8,10,12,8,6,12,7,8,15,11,6 | ENSMUSG00000107693,"ENSMUSG00000107693",1,0,0,0,0,0,0,0,0,0,0,0,0,1,0,0,0,0,0,0 |
| ENSMUSG00000059796,"ENSMUSG00000059796",11689,15178,17757,19504,16797,16192,17454,13253,15579,16865,18341,15106,14410,16930,15574,16356,14625,18319,13865,14869 | ENSMUSG00000064919,"ENSMUSG00000064919",8,23,7,9,11,9,12,18,13,14,10,13,5,11,8,13,1,8,11,6 | ENSMUSG00000070995,"ENSMUSG00000070995",0,0,0,0,0,0,0,0,0,0,0,1,0,0,0,0,0,0,0,0 |
| ENSMUSG00000001918,"ENSMUSG00000001918",1045,1141,1401,1407,1327,1469,1351,978,1202,1331,1244,1023,1132,1368,1234,1173,1213,1621,1189,1233 | ENSMUSG00000084347,"ENSMUSG00000084347",5,6,7,10,14,7,6,12,11,11,8,9,11,13,11,15,7,11,8,6 | ENSMUSG00000108113,"ENSMUSG00000108113",0,0,0,0,0,0,0,0,0,0,0,0,0,0,0,0,0,0,0,0 |
| ENSMUSG00000056973,"ENSMUSG00000056973",92,94,51,92,113,92,108,55,69,95,75,66,75,87,56,47,62,76,83,82 | ENSMUSG00000035295,"ENSMUSG00000035295",2,3,5,2,2,1,1,3,3,1,6,5,2,3,4,9,2,4,5,5 | ENSMUSG00000109416,"ENSMUSG00000109416",0,0,0,0,0,0,0,0,0,0,2,0,0,0,0,0,0,0,0,0 |
| ENSMUSG00000025809,"ENSMUSG00000025809",12680,12340,15241,13373,15583,14060,13762,10708,12755,15081,12659,10633,11786,13554,12672,14696,12854,15411,8275,12754 | ENSMUSG00000031553,"ENSMUSG00000031553",3,9,7,7,4,5,4,4,10,6,3,2,10,2,3,9,12,5,7,7 | ENSMUSG00000090968,"ENSMUSG00000090968",0,0,0,0,0,0,0,0,0,0,0,0,0,0,0,1,0,0,0,0 |
| ENSMUSG00000058558,"ENSMUSG00000058558",8286,8753,10671,10576,9557,9110,9395,7151,8356,9596,10728,8482,8464,8698,8603,9675,8257,10808,7991,9053 | ENSMUSG00000102374,"ENSMUSG00000102374",5,8,4,9,11,8,8,16,6,15,13,8,8,8,4,15,13,10,7,7 | ENSMUSG00000092523,"ENSMUSG00000092523",0,0,0,0,0,0,0,0,0,0,0,0,0,0,0,0,0,0,0,0 |
| ENSMUSG00000022961,"ENSMUSG00000022961",10928,9829,10865,11203,11683,11059,10271,8868,10119,11491,11686,8916,10479,10911,9927,11284,10345,12421,7688,9560 | ENSMUSG00000104117,"ENSMUSG00000104117",0,1,1,0,2,6,0,3,1,3,10,4,2,1,1,3,1,5,1,2 | ENSMUSG00000090714,"ENSMUSG00000090714",0,0,0,0,0,0,0,0,0,0,0,0,0,0,0,0,0,0,0,0 |
| ENSMUSG00000025290,"ENSMUSG00000025290",5184,10225,11942,11539,10665,9945,10073,8073,9508,11045,11482,9726,9847,9408,9577,11578,9381,11861,10102,10418 | ENSMUSG00000108030,"ENSMUSG00000108030",1,1,0,0,4,6,1,3,1,5,2,2,1,0,2,3,1,4,3,2 | ENSMUSG00000100566,"ENSMUSG00000100566",0,0,0,0,0,0,0,0,0,0,0,0,0,0,0,0,0,0,0,0 |
| ENSMUSG00000022565,"ENSMUSG00000022565",7837,11525,14069,14745,13476,13003,13812,9902,11987,14727,13585,10519,12563,14852,11557,14519,12659,15853,8958,11473 | ENSMUSG00000056411,"ENSMUSG00000056411",13,5,8,13,7,8,10,13,6,6,15,10,5,13,11,2,8,11,8,5 | ENSMUSG00000098520,"ENSMUSG00000098520",0,0,0,0,0,0,0,0,0,0,0,0,0,0,0,0,0,0,0,0 |
| ENSMUSG00000028691,"ENSMUSG00000028691",5365,4968,6055,5650,5632,5635,5379,4089,4882,5729,5757,4735,4876,5174,4702,5358,4592,5937,4276,5048 | ENSMUSG00000086140,"ENSMUSG00000086140",0,0,0,0,0,0,0,0,0,0,0,0,0,0,0,0,0,0,0,0 | ENSMUSG00000108331,"ENSMUSG00000108331",0,0,0,1,0,2,0,0,1,0,0,0,0,0,0,0,0,0,0,0 |
| ENSMUSG00000004044,"ENSMUSG00000004044",1420,2021,3150,2219,2175,3291,2156,2213,1989,2616,2678,1683,3289,2321,2392,2397,2245,3318,1984,2903 | ENSMUSG00000081453,"ENSMUSG00000081453",2,11,7,2,5,10,6,3,10,10,10,13,2,4,6,11,2,11,6,3 | ENSMUSG00000108835,"ENSMUSG00000108835",0,0,0,0,0,0,0,0,0,0,0,0,0,0,2,0,0,0,0,0 |
| ENSMUSG00000019929,"ENSMUSG00000019929",19076,13243,19428,17446,17138,18075,15309,13702,14086,15346,16557,11941,17562,13793,13352,14944,13710,18832,12145,14866 | ENSMUSG00000036131,"ENSMUSG00000036131",12,10,16,17,18,14,12,25,13,11,14,8,27,17,10,29,16,17,11,24 | ENSMUSG00000004542,"ENSMUSG00000004542",0,0,0,0,0,0,0,0,0,0,0,0,0,0,0,0,0,0,0,0 |
| ENSMUSG00000038900,"ENSMUSG00000038900",10085,10426,12992,11844,11137,10605,10836,8334,9781,11084,11748,9741,10215,10484,9526,12057,9305,12753,10030,10428 | ENSMUSG00000106858,"ENSMUSG00000106858",19,23,29,12,30,23,17,19,24,32,13,20,14,28,20,20,19,19,14,15 | ENSMUSG00000108567,"ENSMUSG00000108567",0,0,0,0,0,0,0,0,0,0,0,1,0,1,0,0,0,0,0,0 |
| ENSMUSG00000056429,"ENSMUSG00000056429",3711,3664,4558,4043,4212,4746,4071,3544,3738,4687,3966,2769,4130,4210,3849,4347,3768,4694,2635,3627 | ENSMUSG00000050967,"ENSMUSG00000050967",4,11,11,9,14,7,5,26,18,4,10,7,22,5,10,13,4,37,11,7 | ENSMUSG00000092514,"ENSMUSG00000092514",0,0,0,0,0,0,0,0,0,0,0,0,0,0,0,0,0,0,0,0 |
| ENSMUSG00000062328,"ENSMUSG00000062328",9796,8651,10479,9708,9305,8921,8704,6972,8279,9438,9345,8252,8774,8567,8259,10529,8207,10578,8391,8786 | ENSMUSG00000102856,"ENSMUSG00000102856",14,8,29,12,15,27,14,6,12,32,23,10,14,15,22,21,14,21,1,9 | ENSMUSG00000099417,"ENSMUSG00000099417",1,0,0,0,0,0,0,0,0,0,0,0,1,0,0,0,0,0,0,0 |
| ENSMUSG00000017493,"ENSMUSG00000017493",3281,4581,10687,4510,6345,8027,5283,5678,4666,5337,6604,3434,9824,4796,5353,6638,6098,8347,4653,6548 | ENSMUSG00000028750,"ENSMUSG00000028750",0,0,0,2,0,3,0,0,0,0,0,1,0,1,1,0,0,0,0,1 | ENSMUSG00000109102,"ENSMUSG00000109102",0,0,0,0,0,0,0,0,0,0,0,0,0,0,1,0,0,0,0,0 |
| ENSMUSG00000047675,"ENSMUSG00000047675",11654,10302,13357,11579,11394,11169,11048,8532,9818,11361,12293,10108,10349,10723,10282,11924,9490,12971,10773,10718 | ENSMUSG00000067642,"ENSMUSG00000067642",7,2,3,4,0,2,3,3,2,1,1,10,9,2,4,12,2,5,2,1 | ENSMUSG00000083381,"ENSMUSG00000083381",0,0,0,0,0,0,0,0,0,0,0,0,0,0,0,0,0,0,0,0 |
| ENSMUSG00000078812,"ENSMUSG00000078812",11069,16090,20234,20933,18551,17003,18667,14845,16647,17721,19181,16380,15724,19018,16668,20707,15593,21205,16137,17788 | ENSMUSG00000071636,"ENSMUSG00000071636",2,1,2,1,0,0,0,0,0,1,0,1,0,1,0,0,0,0,0,1 | ENSMUSG00000099765,"ENSMUSG00000099765",0,0,0,0,1,0,0,0,0,0,0,0,0,0,0,0,0,0,0,2 |
| ENSMUSG00000006333,"ENSMUSG00000006333",5716,10251,15076,12932,11601,11618,11475,9363,10103,11686,12297,9647,11838,11802,10959,13456,10330,15361,11698,11663 | ENSMUSG00000107509,"ENSMUSG00000107509",25,5,10,16,20,11,9,7,9,15,18,11,8,17,9,7,16,8,11,7 | ENSMUSG00000102464,"ENSMUSG00000102464",0,0,0,0,0,0,0,0,0,0,0,0,0,0,0,0,0,0,0,0 |
| ENSMUSG00000060002,"ENSMUSG00000060002",393,321,395,374,370,310,309,337,319,370,333,265,437,275,280,338,381,485,297,373 | ENSMUSG00000033027,"ENSMUSG00000033027",14,9,16,7,14,7,8,14,10,11,19,18,22,5,6,19,15,16,5,16 | ENSMUSG00000048312,"ENSMUSG00000048312",0,0,0,0,0,0,0,0,0,0,0,0,0,0,0,0,0,1,0,0 |
| ENSMUSG00000037266,"ENSMUSG00000037266",5871,4738,7221,6395,6921,5925,5015,5400,4940,5445,7866,4905,8058,5671,5840,7215,5829,7868,5464,5588 | ENSMUSG00000078787,"ENSMUSG00000078787",8,5,12,5,3,8,8,3,4,10,5,6,5,14,6,4,1,12,5,20 | ENSMUSG00000108754,"ENSMUSG00000108754",0,0,0,0,0,0,0,0,0,0,0,0,0,0,0,0,0,1,1,0 |
| ENSMUSG00000022892,"ENSMUSG00000022892",6479,8861,11646,10331,10473,11258,9053,9074,9116,10443,9670,6698,10801,10643,9209,11646,9674,13165,9509,10343 | ENSMUSG00000072624,"ENSMUSG00000072624",0,0,0,2,0,1,0,0,0,0,0,0,0,0,0,0,0,0,0,0 | ENSMUSG00000092301,"ENSMUSG00000092301",0,0,0,0,0,0,0,0,0,0,0,0,0,0,0,0,0,0,0,0 |
| ENSMUSG00000027556,"ENSMUSG00000027556",0,0,0,0,0,1,0,0,0,0,0,0,0,0,0,0,0,0,0,0 | ENSMUSG00000087303,"ENSMUSG00000087303",1,3,2,0,3,0,0,2,0,1,0,0,0,1,0,1,0,0,1,1 | ENSMUSG00000064194,"ENSMUSG00000064194",0,0,0,0,0,0,0,0,0,0,0,0,0,0,0,0,0,0,0,0 |
| ENSMUSG00000073418,"ENSMUSG00000073418",1539,1952,2466,2659,2152,1993,2384,2710,2321,2416,2709,1584,3548,2398,2484,2636,2574,3699,2978,3062 | ENSMUSG00000105872,"ENSMUSG00000105872",0,0,0,0,0,0,0,0,0,0,0,0,0,0,0,0,0,0,0,0 | ENSMUSG00000060177,"ENSMUSG00000060177",0,0,0,0,0,0,0,0,0,0,0,0,0,0,0,0,0,0,0,0 |
| ENSMUSG00000028639,"ENSMUSG00000028639",2947,7594,11289,8720,9429,8724,7917,7520,7669,9374,9438,7145,9371,8149,8736,9194,8183,10405,7188,8840 | ENSMUSG00000094622,"ENSMUSG00000094622",3,4,3,2,6,4,2,6,3,8,1,5,5,4,2,1,6,0,3,3 | ENSMUSG00000108774,"ENSMUSG00000108774",0,0,0,0,0,0,0,0,0,0,0,0,0,0,0,0,0,0,0,0 |
| ENSMUSG00000023175,"ENSMUSG00000023175",3785,4628,6744,5657,6046,5081,4711,5391,4596,4912,5845,4391,6549,5302,5444,6576,5030,7096,5702,6242 | ENSMUSG00000114399,"ENSMUSG00000114399",6,0,3,2,5,3,0,1,3,8,4,3,1,3,1,4,3,2,5,0 | ENSMUSG00000109926,"ENSMUSG00000109926",0,0,0,0,0,0,0,0,0,0,0,0,0,0,0,0,0,0,0,0 |
| ENSMUSG00000063524,"ENSMUSG00000063524",11020,12787,19067,14994,17716,15135,13026,16203,13533,13686,14802,13042,18632,14927,14973,20158,13956,21807,14723,18792 | ENSMUSG00000099875,"ENSMUSG00000099875",28,26,38,26,34,28,24,11,22,30,29,30,26,23,16,27,21,35,4,29 | ENSMUSG00000099560,"ENSMUSG00000099560",0,0,0,0,0,0,0,0,0,0,0,0,0,0,0,0,0,0,0,0 |
| ENSMUSG00000000787,"ENSMUSG00000000787",15433,13951,14944,15177,16028,15325,14325,10447,13936,15972,14823,12657,11708,13928,12302,14278,12569,15159,8883,11605 | ENSMUSG00000084479,"ENSMUSG00000084479",0,0,0,0,0,0,0,0,0,0,0,0,0,0,0,0,0,0,0,0 | ENSMUSG00000109164,"ENSMUSG00000109164",0,0,0,0,0,0,0,0,0,0,0,0,0,0,0,0,0,0,0,0 |
| ENSMUSG00000028495,"ENSMUSG00000028495",13581,9110,10773,10915,9281,9311,8761,7124,8358,9641,10727,8578,8543,9404,8609,10639,8493,10965,7450,9452 | ENSMUSG00000048065,"ENSMUSG00000048065",3,4,3,17,10,10,12,11,6,12,17,4,12,16,14,12,12,18,10,1 | ENSMUSG00000109393,"ENSMUSG00000109393",0,0,0,0,0,0,0,0,0,0,0,0,0,0,0,0,0,0,0,0 |
| ENSMUSG00000015932,"ENSMUSG00000015932",13101,10713,11862,13899,12445,10238,12161,8086,10727,12255,12469,11554,9559,10870,10913,12312,9958,12279,8106,10090 | ENSMUSG00000065734,"ENSMUSG00000065734",7,13,10,28,13,10,17,6,10,17,15,25,14,19,12,16,18,13,10,29 | ENSMUSG00000108421,"ENSMUSG00000108421",0,0,0,0,0,0,0,0,0,0,0,0,0,0,0,0,0,0,0,0 |
| ENSMUSG00000024892,"ENSMUSG00000024892",598,760,969,1310,989,714,921,776,875,690,886,948,699,912,866,963,732,1039,930,826 | ENSMUSG00000114452,"ENSMUSG00000114452",4,5,4,6,2,3,5,5,2,2,6,1,4,1,0,4,2,5,2,2 | ENSMUSG00000109152,"ENSMUSG00000109152",0,3,0,1,0,0,0,0,0,0,0,0,0,0,0,0,0,0,0,0 |
| ENSMUSG00000071178,"ENSMUSG00000071178",21,17,44,31,35,30,42,33,26,29,36,19,35,31,26,48,21,56,43,36 | ENSMUSG00000047591,"ENSMUSG00000047591",7,12,29,22,23,20,17,25,29,39,19,25,40,22,26,21,21,25,24,30 | ENSMUSG00000109161,"ENSMUSG00000109161",0,0,0,0,0,0,0,0,0,0,0,0,0,0,0,0,0,0,0,0 |
| ENSMUSG00000090841,"ENSMUSG00000090841",1682,5661,8588,6873,6078,5913,6458,5731,5579,6798,7387,5359,7632,7024,6969,7942,6206,8504,7253,7421 | ENSMUSG00000114104,"ENSMUSG00000114104",16,11,15,8,10,13,13,28,10,10,9,5,18,14,15,6,4,10,4,27 | ENSMUSG00000070511,"ENSMUSG00000070511",0,0,0,0,0,0,0,0,0,0,0,0,0,0,0,0,0,0,0,0 |
| ENSMUSG00000021546,"ENSMUSG00000021546",9108,10746,12448,13130,12905,11408,11436,9278,10880,11775,12164,9963,10355,11428,10312,11852,10341,13141,8995,10676 | ENSMUSG00000063328,"ENSMUSG00000063328",3,3,6,11,5,7,11,6,11,15,15,19,7,14,8,13,11,10,7,11 | ENSMUSG00000098035,"ENSMUSG00000098035",0,0,0,0,0,0,0,0,1,0,0,0,0,0,0,0,0,0,0,1 |
| ENSMUSG00000039630,"ENSMUSG00000039630",6462,8022,9767,9883,9041,8799,8720,6900,7874,9419,10307,7634,8464,8537,8384,9242,8091,10013,7240,8186 | ENSMUSG00000038204,"ENSMUSG00000038204",23,11,34,37,24,3,23,35,19,38,33,19,42,18,20,33,32,34,49,43 | ENSMUSG00000108803,"ENSMUSG00000108803",0,0,0,0,0,0,0,0,0,0,0,0,0,0,0,0,0,0,0,0 |
| ENSMUSG00000020368,"ENSMUSG00000020368",6966,6512,8691,8780,8050,7288,7708,6881,7504,7445,9373,7009,8070,7655,7590,7663,7106,8667,5768,7571 | ENSMUSG00000107296,"ENSMUSG00000107296",0,2,0,1,0,0,0,1,0,0,0,0,0,0,0,0,0,2,0,0 | ENSMUSG00000069196,"ENSMUSG00000069196",0,0,0,0,0,0,0,0,0,0,0,0,0,0,0,0,0,0,0,0 |
| ENSMUSG00000028465,"ENSMUSG00000028465",6251,7999,10343,9000,9606,10871,9203,8356,8524,9451,8880,5882,9484,9853,8136,10348,9326,11264,7089,9084 | ENSMUSG00000063063,"ENSMUSG00000063063",1,0,0,1,0,1,0,1,1,0,0,0,2,0,2,1,0,0,1,2 | ENSMUSG00000066378,"ENSMUSG00000066378",0,0,0,0,0,0,0,0,0,0,0,0,0,0,0,0,0,0,0,0 |
| ENSMUSG00000026238,"ENSMUSG00000026238",5076,8520,12115,11780,9674,9837,9066,8159,8894,9337,11456,8690,10362,9847,9194,10331,8506,12564,11925,10638 | ENSMUSG00000030732,"ENSMUSG00000030732",2,7,6,9,6,1,4,6,4,10,6,7,13,7,15,13,7,11,19,18 | ENSMUSG00000108861,"ENSMUSG00000108861",0,0,0,0,0,0,0,0,0,0,0,0,0,0,0,0,0,0,0,0 |
| ENSMUSG00000012848,"ENSMUSG00000012848",11372,9401,11464,11607,9931,9326,10045,7419,8735,10246,11149,9165,9250,9592,9221,11028,9011,11296,9964,9585 | ENSMUSG00000102414,"ENSMUSG00000102414",32,38,57,71,58,53,62,41,38,19,56,40,35,25,53,53,44,83,19,25 | ENSMUSG00000039608,"ENSMUSG00000039608",0,0,0,0,0,0,0,0,0,0,0,0,0,0,0,0,0,0,0,0 |
| ENSMUSG00000003123,"ENSMUSG00000003123",542,835,955,959,992,861,1075,805,895,926,918,798,996,987,1028,1059,890,1167,877,979 | ENSMUSG00000078907,"ENSMUSG00000078907",2,3,3,4,2,4,10,2,4,4,5,4,7,2,3,3,9,4,3,3 | ENSMUSG00000108953,"ENSMUSG00000108953",0,0,0,0,0,0,0,0,0,0,0,0,0,0,0,0,0,0,0,0 |
| ENSMUSG00000021794,"ENSMUSG00000021794",2680,2887,3683,3288,3340,3254,2897,2323,2707,3461,3378,2525,3040,2778,2649,3008,2855,3496,2252,2769 | ENSMUSG00000046160,"ENSMUSG00000046160",0,3,3,5,0,0,1,1,0,1,6,3,1,0,1,2,2,1,1,0 | ENSMUSG00000087980,"ENSMUSG00000087980",0,0,1,1,0,0,0,0,0,0,0,0,0,0,0,0,0,0,0,0 |
| ENSMUSG00000028649,"ENSMUSG00000028649",11704,11656,13416,13806,14730,12588,13811,9807,12144,13488,12315,11721,10280,13208,11992,14120,12413,15398,8097,10886 | ENSMUSG00000086109,"ENSMUSG00000086109",8,19,19,23,17,14,17,27,13,24,29,12,24,15,19,25,17,19,21,22 | ENSMUSG00000053360,"ENSMUSG00000053360",0,0,0,0,0,0,0,0,0,1,0,0,0,0,0,0,0,0,0,0 |
| ENSMUSG00000055737,"ENSMUSG00000055737",637,736,752,974,839,741,706,543,650,817,774,638,674,646,637,617,599,730,508,581 | ENSMUSG00000087611,"ENSMUSG00000087611",0,2,1,3,0,1,1,3,5,3,9,2,0,1,1,6,1,5,0,4 | ENSMUSG00000073966,"ENSMUSG00000073966",0,0,0,0,0,0,0,0,0,0,0,0,0,0,0,0,0,0,0,0 |
| ENSMUSG00000061477,"ENSMUSG00000061477",7251,7525,9395,8886,8591,8229,8297,6469,7379,8789,8904,7251,7677,7797,7166,8721,7172,9230,6788,7467 | ENSMUSG00000069540,"ENSMUSG00000069540",6,1,4,1,5,3,6,0,7,7,3,8,5,5,1,2,1,5,0,7 | ENSMUSG00000058976,"ENSMUSG00000058976",0,0,0,0,0,0,0,0,0,0,0,0,0,0,0,0,0,0,0,0 |
| ENSMUSG00000028024,"ENSMUSG00000028024",53,65,72,52,48,26,52,46,47,57,60,35,73,52,49,56,34,66,37,39 | ENSMUSG00000086839,"ENSMUSG00000086839",3,3,7,0,7,4,9,3,4,3,6,4,8,7,4,0,9,10,1,3 | ENSMUSG00000058662,"ENSMUSG00000058662",0,0,0,0,0,0,0,0,0,0,0,0,0,0,0,0,0,0,0,0 |
| ENSMUSG00000024990,"ENSMUSG00000024990",68,135,119,120,132,112,143,137,115,177,118,94,161,124,105,98,137,194,151,154 | ENSMUSG00000048834,"ENSMUSG00000048834",38,33,43,23,35,44,40,34,58,24,69,17,61,40,26,50,48,61,20,29 | ENSMUSG00000073937,"ENSMUSG00000073937",0,0,0,0,0,0,0,0,0,0,0,0,0,0,0,0,0,0,0,0 |
| ENSMUSG00000026701,"ENSMUSG00000026701",1901,1877,2383,2574,2187,1979,2015,1852,1950,2023,2324,1997,2118,2024,1818,2358,1764,2510,1763,2096 | ENSMUSG00000085766,"ENSMUSG00000085766",6,12,11,2,7,9,12,11,11,15,13,5,10,8,10,14,12,15,21,14 | ENSMUSG00000044899,"ENSMUSG00000044899",0,2,0,0,0,0,0,0,0,0,0,0,0,0,0,0,0,0,0,0 |
| ENSMUSG00000029661,"ENSMUSG00000029661",13670,14268,25128,15281,16413,37771,17264,20669,16444,17174,18163,12283,28146,15905,18411,18987,18569,25649,14133,21506 | ENSMUSG00000002104,"ENSMUSG00000002104",11,20,38,16,26,13,18,20,16,29,35,9,37,19,24,27,24,33,26,37 | ENSMUSG00000109676,"ENSMUSG00000109676",0,0,0,0,0,0,2,0,0,0,1,0,0,0,0,1,0,0,0,0 |
| ENSMUSG00000034427,"ENSMUSG00000034427",8,18,28,20,12,9,19,10,14,4,12,17,12,16,13,10,11,7,11,23 | ENSMUSG00000036231,"ENSMUSG00000036231",0,1,0,1,0,0,2,1,0,0,0,1,0,0,0,1,0,0,0,0 | ENSMUSG00000059087,"ENSMUSG00000059087",0,0,0,0,0,0,0,0,0,1,0,0,0,0,0,0,0,0,0,0 |
| ENSMUSG00000018326,"ENSMUSG00000018326",4148,4122,5009,5101,4898,4738,4492,3597,4185,4711,4803,3733,4268,4371,4111,4751,4143,5024,3376,4152 | ENSMUSG00000073295,"ENSMUSG00000073295",10,20,15,14,16,12,12,7,12,17,24,13,6,15,9,14,24,19,13,29 | ENSMUSG00000066197,"ENSMUSG00000066197",0,0,0,0,0,0,0,0,0,0,0,0,0,0,0,0,0,0,0,0 |
| ENSMUSG00000001156,"ENSMUSG00000001156",14130,13191,14317,13028,15212,14714,14018,15320,14495,17997,13806,9938,17818,17927,14968,18202,14625,17908,8738,16552 | ENSMUSG00000079450,"ENSMUSG00000079450",0,0,0,0,0,0,0,0,0,0,0,0,0,0,0,1,0,0,0,0 | ENSMUSG00000108911,"ENSMUSG00000108911",0,0,0,0,0,0,0,0,0,0,0,0,0,0,0,0,0,0,0,0 |
| ENSMUSG00000031502,"ENSMUSG00000031502",3077,5104,8220,6186,5652,9052,5982,5398,5761,7589,7089,3542,8543,6590,6279,7098,6205,7894,4150,5960 | ENSMUSG00000065191,"ENSMUSG00000065191",2,4,2,6,0,4,2,3,0,0,5,1,4,2,3,3,5,2,3,3 | ENSMUSG00000108708,"ENSMUSG00000108708",0,0,0,0,0,0,0,0,0,0,0,0,0,1,0,0,0,0,0,0 |
| ENSMUSG00000028234,"ENSMUSG00000028234",8660,10311,11670,12695,10132,9449,10513,7987,10196,11618,11989,10172,9590,10356,9252,12635,9086,12384,9259,10094 | ENSMUSG00000090192,"ENSMUSG00000090192",27,21,42,25,23,16,25,28,34,44,24,14,24,35,30,39,15,50,27,28 | ENSMUSG00000108309,"ENSMUSG00000108309",0,0,0,0,0,0,0,0,0,0,0,0,0,0,0,0,0,0,0,0 |
| ENSMUSG00000026576,"ENSMUSG00000026576",647,589,656,707,779,459,504,492,557,717,741,539,777,620,591,620,565,803,590,721 | ENSMUSG00000032292,"ENSMUSG00000032292",0,5,0,1,0,0,0,1,1,1,0,0,0,0,0,0,0,0,0,0 | ENSMUSG00000108583,"ENSMUSG00000108583",0,0,0,0,0,0,0,0,0,0,0,0,0,0,0,0,0,0,0,0 |
| ENSMUSG00000062647,"ENSMUSG00000062647",4744,9746,12974,12528,10620,10625,10775,8484,9547,10824,12148,9657,10456,11056,10308,12209,9426,12821,10627,10523 | ENSMUSG00000102614,"ENSMUSG00000102614",1,1,2,2,0,0,1,0,0,0,2,1,0,3,0,0,0,1,1,0 | ENSMUSG00000092905,"ENSMUSG00000092905",0,0,0,0,0,0,0,0,0,0,0,0,0,0,0,0,0,0,0,0 |
| ENSMUSG00000057738,"ENSMUSG00000057738",5465,6568,8544,9147,8346,7456,7949,6740,7192,7657,8486,6608,8187,7961,7564,8541,7436,9100,5942,7360 | ENSMUSG00000105199,"ENSMUSG00000105199",9,2,4,2,2,5,4,4,3,0,4,2,2,4,3,1,5,7,2,3 | ENSMUSG00000108663,"ENSMUSG00000108663",0,0,0,0,0,0,0,0,0,0,0,0,0,0,0,0,0,0,0,0 |
| ENSMUSG00000047215,"ENSMUSG00000047215",8101,9085,10662,10647,9734,8951,9328,6968,8848,10089,10269,8698,8678,9186,8275,10598,8075,10669,8498,9298 | ENSMUSG00000096795,"ENSMUSG00000096795",33,28,22,30,29,20,14,21,14,25,20,11,21,22,17,18,15,25,13,22 | ENSMUSG00000110354,"ENSMUSG00000110354",0,0,0,0,0,0,0,0,0,0,0,0,0,0,0,0,0,0,0,0 |
| ENSMUSG00000059070,"ENSMUSG00000059070",6551,8247,12293,10579,8399,8582,8545,7296,7800,9484,10573,8199,9569,9526,8723,10684,7888,11752,8924,9487 | ENSMUSG00000047155,"ENSMUSG00000047155",0,0,0,0,0,0,0,0,0,0,0,0,0,0,5,0,0,0,0,0 | ENSMUSG00000052508,"ENSMUSG00000052508",0,0,0,0,0,0,0,0,0,1,0,0,0,0,0,0,0,0,0,0 |
| ENSMUSG00000022587,"ENSMUSG00000022587",7964,6873,8586,12265,8639,6938,8290,10472,9473,7887,10188,8484,10261,8326,9553,11443,8728,13042,10246,11419 | ENSMUSG00000097215,"ENSMUSG00000097215",36,31,23,37,36,44,43,31,27,27,48,22,33,25,31,56,38,42,41,40 | ENSMUSG00000083812,"ENSMUSG00000083812",0,0,0,0,0,0,2,1,0,0,0,0,0,0,0,0,0,1,0,0 |
| ENSMUSG00000076441,"ENSMUSG00000076441",776,1277,1722,1323,1395,1597,1300,1416,1342,1199,1354,1045,1588,1385,1355,1803,1256,1933,1464,1881 | ENSMUSG00000112082,"ENSMUSG00000112082",10,6,3,5,2,9,5,8,9,6,6,1,8,9,6,12,5,6,3,5 | ENSMUSG00000110040,"ENSMUSG00000110040",0,0,0,0,0,0,1,0,0,0,0,0,0,0,0,0,0,0,1,1 |
| ENSMUSG00000000204,"ENSMUSG00000000204",30557,25599,31498,28982,32571,41599,33317,42288,34223,40706,33601,18717,50606,42088,39816,53572,36893,56400,23615,47676 | ENSMUSG00000087203,"ENSMUSG00000087203",5,3,3,3,0,4,3,3,4,10,5,2,4,3,1,6,6,3,8,2 | ENSMUSG00000112048,"ENSMUSG00000112048",0,0,0,0,0,0,0,0,0,0,0,0,0,0,0,0,0,0,0,0 |
| ENSMUSG00000024608,"ENSMUSG00000024608",11268,7948,10732,9262,8532,8282,7944,6573,7363,8826,8751,7011,8627,8294,7668,9255,7236,10049,8979,8380 | ENSMUSG00000103261,"ENSMUSG00000103261",58,0,3,3,3,159,6,58,157,49,11,0,40,71,2,5,1,3,3,0 | ENSMUSG00000112307,"ENSMUSG00000112307",0,0,0,0,0,0,0,0,0,0,0,0,0,0,0,0,0,0,0,0 |
| ENSMUSG00000048076,"ENSMUSG00000048076",6960,8689,11877,10846,10911,10206,10151,8985,9365,9974,10409,8348,10317,10782,9655,11125,9511,12377,9158,10415 | ENSMUSG00000068860,"ENSMUSG00000068860",6,24,21,18,15,16,20,13,20,15,25,20,16,11,13,23,16,20,13,21 | ENSMUSG00000111951,"ENSMUSG00000111951",0,0,0,0,0,0,0,0,0,0,0,0,0,0,0,1,0,0,0,0 |
| ENSMUSG00000023019,"ENSMUSG00000023019",40,127,189,150,187,128,138,141,126,165,193,112,214,173,151,147,149,126,199,145 | ENSMUSG00000082065,"ENSMUSG00000082065",0,0,0,0,0,0,0,0,0,0,0,0,0,0,0,0,0,0,0,0 | ENSMUSG00000111177,"ENSMUSG00000111177",0,0,0,0,0,0,0,0,0,0,0,0,2,0,0,0,0,2,0,0 |
| ENSMUSG00000038612,"ENSMUSG00000038612",8986,12082,14203,11547,13757,13590,11787,12893,13123,15349,12084,8940,14245,15059,12196,14859,12437,15353,8802,13911 | ENSMUSG00000086368,"ENSMUSG00000086368",3,9,9,11,12,6,9,7,5,6,20,10,15,13,6,11,7,10,10,10 | ENSMUSG00000112370,"ENSMUSG00000112370",0,0,0,0,0,0,0,0,0,0,0,0,0,0,0,0,0,0,0,0 |
| ENSMUSG00000017950,"ENSMUSG00000017950",0,0,0,0,1,0,0,2,2,1,0,0,0,0,1,0,0,0,0,5 | ENSMUSG00000037846,"ENSMUSG00000037846",12,1,13,6,9,7,5,4,5,2,11,8,2,3,6,9,8,6,8,5 | ENSMUSG00000111576,"ENSMUSG00000111576",0,0,0,0,0,0,0,0,0,0,0,0,0,0,0,0,0,0,0,0 |
| ENSMUSG00000005683,"ENSMUSG00000005683",3694,4409,4832,6012,5208,4250,5431,4003,4686,4539,5412,4610,4504,4854,4469,4920,4209,5184,4750,4950 | ENSMUSG00000056487,"ENSMUSG00000056487",0,0,0,0,0,0,0,0,0,0,0,0,0,0,0,0,0,0,0,0 | ENSMUSG00000110893,"ENSMUSG00000110893",0,0,0,0,0,0,0,0,0,0,0,0,0,0,0,0,0,0,0,0 |
| ENSMUSG00000039361,"ENSMUSG00000039361",13030,13305,14874,13226,15881,15263,12335,11829,12833,16795,13209,9787,13021,15183,12480,15340,13889,15023,9007,12438 | ENSMUSG00000088552,"ENSMUSG00000088552",32,31,13,26,44,33,45,34,26,26,31,23,36,36,34,30,35,46,36,37 | ENSMUSG00000098409,"ENSMUSG00000098409",0,0,0,0,0,0,0,2,0,0,0,0,2,0,0,0,0,0,0,0 |
| ENSMUSG00000033174,"ENSMUSG00000033174",1198,1645,1268,2523,1516,2204,2341,873,1616,1672,1668,1507,1119,1664,1055,1223,1123,1766,1537,1055 | ENSMUSG00000109379,"ENSMUSG00000109379",3,8,8,11,11,3,10,8,11,9,10,3,5,1,8,7,9,6,8,3 | ENSMUSG00000112295,"ENSMUSG00000112295",0,0,0,0,0,0,0,0,0,0,0,0,0,0,0,1,0,0,0,0 |
| ENSMUSG00000025362,"ENSMUSG00000025362",11234,9393,11084,10731,10159,9559,10648,7293,8832,10263,10701,9206,9105,9347,8336,10687,8230,10851,8255,9714 | ENSMUSG00000101279,"ENSMUSG00000101279",26,32,27,15,22,37,17,33,29,33,21,19,36,32,25,50,26,42,10,21 | ENSMUSG00000112349,"ENSMUSG00000112349",0,0,0,0,0,0,0,0,0,0,0,0,0,0,0,0,0,0,0,0 |
| ENSMUSG00000019818,"ENSMUSG00000019818",13725,11260,13263,13697,14195,13644,11776,10454,12665,14552,11590,9600,10714,13210,10935,13371,12715,13187,7444,11252 | ENSMUSG00000085151,"ENSMUSG00000085151",0,2,0,1,0,1,0,2,1,2,1,0,0,0,5,1,1,2,1,1 | ENSMUSG00000112049,"ENSMUSG00000112049",0,0,0,0,0,0,0,0,0,0,0,0,0,0,0,0,0,0,0,0 |
| ENSMUSG00000018707,"ENSMUSG00000018707",5094,6408,7651,9777,8054,6634,8346,6476,7449,6072,7328,5911,6754,7045,6332,7936,6756,8034,6113,6983 | ENSMUSG00000060070,"ENSMUSG00000060070",0,0,0,0,0,0,0,0,0,0,0,0,0,0,0,0,0,0,0,0 | ENSMUSG00000111880,"ENSMUSG00000111880",0,0,0,0,0,0,0,0,0,0,0,0,0,0,0,0,0,0,0,0 |
| ENSMUSG00000020321,"ENSMUSG00000020321",3001,2555,3012,2872,2778,2390,2710,2225,2436,2850,3317,2292,2926,2472,2509,2727,2366,3174,2546,2567 | ENSMUSG00000090105,"ENSMUSG00000090105",0,1,3,1,5,1,0,0,4,4,4,4,1,4,6,0,5,3,0,3 | ENSMUSG00000091731,"ENSMUSG00000091731",0,0,0,0,0,0,0,0,0,0,0,0,0,0,0,0,0,0,0,0 |
| ENSMUSG00000019505,"ENSMUSG00000019505",3131,9661,13756,12528,9827,9966,9415,9795,10058,11896,12443,9450,12569,11876,11147,13507,10913,14202,7847,12817 | ENSMUSG00000074569,"ENSMUSG00000074569",15,11,15,17,10,18,10,21,8,24,18,12,4,17,14,16,18,13,10,10 | ENSMUSG00000080526,"ENSMUSG00000080526",0,0,0,0,0,0,0,0,0,0,0,0,0,0,0,0,0,0,0,0 |
| ENSMUSG00000025982,"ENSMUSG00000025982",9385,7983,8940,8449,9625,8911,7973,7108,8109,9300,8860,6741,8605,8701,7629,9445,7847,9649,5729,7744 | ENSMUSG00000086695,"ENSMUSG00000086695",9,14,16,7,12,4,10,14,4,25,13,2,9,28,15,24,4,13,14,19 | ENSMUSG00000085662,"ENSMUSG00000085662",0,0,0,0,0,0,0,0,0,0,0,0,0,0,0,0,0,0,0,0 |
| ENSMUSG00000029467,"ENSMUSG00000029467",11803,11551,13145,14315,14134,11487,14457,10228,12360,12986,12455,10894,11125,12618,12469,13377,11644,15303,11091,12176 | ENSMUSG00000086890,"ENSMUSG00000086890",1,7,7,10,3,4,10,5,13,7,9,12,8,5,2,10,3,5,10,6 | ENSMUSG00000112653,"ENSMUSG00000112653",0,0,0,0,0,2,0,0,0,0,0,0,0,0,0,0,0,0,1,0 |
| ENSMUSG00000086503,"ENSMUSG00000086503",11644,9364,10551,9333,10667,9937,8292,8335,8679,10338,12127,8805,10493,9009,10015,9858,9609,10724,7306,8162 | ENSMUSG00000095338,"ENSMUSG00000095338",0,0,0,0,0,0,0,0,0,0,0,0,0,0,0,0,0,0,0,0 | ENSMUSG00000112623,"ENSMUSG00000112623",0,0,0,0,0,1,0,0,0,0,0,0,0,0,0,0,0,1,0,0 |
| ENSMUSG00000034957,"ENSMUSG00000034957",3008,4350,4965,5043,4507,4112,5188,3517,4497,4863,5104,4295,4951,4584,4380,4475,4170,5716,3837,4577 | ENSMUSG00000071303,"ENSMUSG00000071303",6,9,5,4,9,7,7,2,13,10,7,18,8,16,9,9,11,9,0,10 | ENSMUSG00000107822,"ENSMUSG00000107822",0,0,0,0,0,0,1,0,0,0,0,0,0,0,0,0,0,2,0,0 |
| ENSMUSG00000019326,"ENSMUSG00000019326",79,74,105,78,117,125,87,82,74,96,116,60,154,87,139,69,84,107,104,131 | ENSMUSG00000094027,"ENSMUSG00000094027",13,14,17,13,15,17,12,14,23,16,11,6,17,8,13,7,11,27,14,11 | ENSMUSG00000112732,"ENSMUSG00000112732",0,0,0,0,0,0,0,0,0,0,0,0,0,0,0,0,0,1,0,0 |
| ENSMUSG00000023456,"ENSMUSG00000023456",3445,5231,9678,8218,9405,6971,5123,9904,6051,6066,7993,7044,11817,7686,8952,11165,7494,12403,9158,10493 | ENSMUSG00000100007,"ENSMUSG00000100007",21,21,26,15,14,30,14,14,17,22,23,23,20,16,17,19,18,26,13,34 | ENSMUSG00000060205,"ENSMUSG00000060205",0,0,0,0,0,0,0,0,0,0,0,0,0,0,0,0,0,0,0,0 |
| ENSMUSG00000029992,"ENSMUSG00000029992",2454,1785,2164,2532,2753,2026,2232,2131,2223,2112,2145,1892,2198,2283,2314,2670,2101,2745,1716,2285 | ENSMUSG00000042388,"ENSMUSG00000042388",3,6,8,7,7,11,9,8,6,0,12,3,5,10,5,13,8,14,11,8 | ENSMUSG00000111524,"ENSMUSG00000111524",0,0,0,0,0,0,0,0,0,0,0,0,0,0,0,1,1,0,0,0 |
| ENSMUSG00000019851,"ENSMUSG00000019851",28939,26695,31516,37040,30744,24969,29697,21878,28654,28067,35548,32035,27330,26651,25665,31998,24285,34184,24133,28572 | ENSMUSG00000115003,"ENSMUSG00000115003",7,11,5,5,7,13,4,4,3,4,2,6,3,4,2,6,9,6,7,5 | ENSMUSG00000112893,"ENSMUSG00000112893",0,0,0,0,0,0,0,0,0,0,0,0,0,0,0,0,0,0,0,0 |
| ENSMUSG00000028656,"ENSMUSG00000028656",8392,9985,12226,11459,12559,12285,11332,10188,10275,12695,11273,8991,11650,12448,11016,12823,11086,13586,9107,11488 | ENSMUSG00000112920,"ENSMUSG00000112920",0,0,0,0,0,0,0,0,3,0,1,0,0,0,0,0,0,0,0,0 | ENSMUSG00000090308,"ENSMUSG00000090308",0,0,0,0,0,0,0,0,0,0,0,0,0,0,0,0,0,0,0,0 |
| ENSMUSG00000030536,"ENSMUSG00000030536",12956,12838,15458,14724,15317,14772,14241,12218,12657,15908,13356,10823,13837,15707,12828,15488,14065,15844,9138,13151 | ENSMUSG00000084790,"ENSMUSG00000084790",5,4,12,2,0,4,11,11,5,9,5,2,5,11,4,5,6,6,6,9 | ENSMUSG00000111349,"ENSMUSG00000111349",0,0,0,0,0,0,0,0,0,1,0,0,0,0,0,0,0,0,0,0 |
| ENSMUSG00000038274,"ENSMUSG00000038274",8129,7300,10831,9085,7932,7744,7260,7212,7291,8576,9164,7537,9219,8694,7865,10548,7460,10791,7891,9111 | ENSMUSG00000045876,"ENSMUSG00000045876",7,5,13,7,13,8,10,17,11,11,20,7,17,12,8,9,12,18,9,9 | ENSMUSG00000112484,"ENSMUSG00000112484",0,0,0,0,0,0,0,0,0,0,0,3,0,0,0,0,0,0,0,0 |
| ENSMUSG00000034918,"ENSMUSG00000034918",0,3,6,0,3,4,8,2,0,7,2,2,1,3,7,1,3,4,4,2 | ENSMUSG00000087739,"ENSMUSG00000087739",4,10,8,1,0,5,4,4,5,6,3,4,5,7,3,3,10,6,8,3 | ENSMUSG00000112896,"ENSMUSG00000112896",0,0,0,0,1,0,0,0,0,0,0,0,0,0,0,0,1,0,0,0 |
| ENSMUSG00000028692,"ENSMUSG00000028692",5217,6341,9286,8594,7839,7269,6555,6274,6782,7355,8395,6216,8267,7409,7311,8438,6593,9309,7197,7597 | ENSMUSG00000108975,"ENSMUSG00000108975",0,1,0,0,0,0,0,0,0,0,0,0,0,0,0,0,0,0,0,0 | ENSMUSG00000084545,"ENSMUSG00000084545",0,0,0,0,0,0,0,0,0,0,0,0,0,0,0,0,0,0,0,0 |
| ENSMUSG00000024576,"ENSMUSG00000024576",14403,12126,13509,15375,14767,11976,13595,10055,13229,13638,13878,12729,10908,12870,12330,14507,11869,15066,9471,11760 | ENSMUSG00000112441,"ENSMUSG00000112441",3,0,3,4,2,1,3,5,3,0,2,1,5,2,2,3,4,3,4,4 | ENSMUSG00000112523,"ENSMUSG00000112523",0,0,0,0,0,0,0,0,0,1,0,0,0,1,0,0,0,0,0,0 |
| ENSMUSG00000031207,"ENSMUSG00000031207",14389,16520,19352,19939,21134,19884,19696,16989,17754,19867,18205,14448,18137,20482,17558,20964,17796,22249,13052,18483 | ENSMUSG00000039155,"ENSMUSG00000039155",0,2,1,0,2,1,1,5,0,2,4,1,2,1,0,0,3,6,3,0 | ENSMUSG00000112016,"ENSMUSG00000112016",0,0,0,0,0,0,0,0,0,0,0,0,0,0,0,0,0,0,0,0 |
| ENSMUSG00000037815,"ENSMUSG00000037815",6847,8445,9682,10862,10132,8283,10170,7179,8932,9721,8984,8274,7552,9995,9117,10638,8488,10720,6364,8977 | ENSMUSG00000030523,"ENSMUSG00000030523",19,8,20,23,21,14,16,10,14,18,12,24,19,11,20,10,17,12,9,22 | ENSMUSG00000112399,"ENSMUSG00000112399",0,0,0,0,0,0,0,0,0,0,0,0,0,0,0,0,0,0,0,0 |
| ENSMUSG00000031089,"ENSMUSG00000031089",23600,17003,17365,22301,25533,14669,20255,15410,17368,20292,17767,19562,13602,18977,21752,23110,19464,22625,13266,18978 | ENSMUSG00000103272,"ENSMUSG00000103272",9,3,8,2,11,9,4,4,4,4,2,11,13,5,3,2,3,9,3,3 | ENSMUSG00000112155,"ENSMUSG00000112155",0,0,0,0,0,0,0,0,0,0,0,0,0,0,0,0,0,0,0,0 |
| ENSMUSG00000021577,"ENSMUSG00000021577",2036,2511,3588,3610,3137,2832,2922,2585,2797,3061,3402,2595,3223,3125,3049,3049,2819,3333,2971,3010 | ENSMUSG00000108105,"ENSMUSG00000108105",1,3,0,1,1,1,2,2,3,0,1,1,4,8,0,5,0,0,0,2 | ENSMUSG00000084764,"ENSMUSG00000084764",0,0,0,0,0,0,0,0,0,1,0,0,0,0,0,0,0,0,0,0 |

| ENSMUSG00000001924,"ENSMUSG00000001924",7675,9081,11620,13091,11539,10423,12181,9806,10784,10356,10744,9020,10473,11722,10545,12105,9887,12788,9588,11053 | ENSMUSG00000110545,"ENSMUSG00000110545",9,16,28,18,18,20,5,11,17,10,14,17,26,8,20,12,18,21,6,12 | ENSMUSG00000112846,"ENSMUSG00000112846",0,0,0,0,0,0,0,0,0,0,0,0,0,0,0,0,0,0,0,0 |
| --- | --- | --- |
| ENSMUSG00000027422,"ENSMUSG00000027422",2895,5938,7885,7668,7186,7083,6289,6170,5857,6592,7010,5256,7313,7704,6670,7855,6660,8705,5599,7049 | ENSMUSG00000104184,"ENSMUSG00000104184",18,17,13,16,21,17,15,33,15,10,22,10,18,17,23,18,23,14,4,33 | ENSMUSG00000112036,"ENSMUSG00000112036",1,0,0,0,0,0,0,0,0,0,0,0,1,0,0,1,0,0,0,0 |
| ENSMUSG00000079017,"ENSMUSG00000079017",1086,1164,1706,1914,1405,1260,1062,2228,1451,1676,2127,1042,3055,1822,2093,2465,2038,2894,2018,2479 | ENSMUSG00000085207,"ENSMUSG00000085207",2,8,10,7,10,5,5,10,2,18,11,3,15,10,1,6,13,17,11,7 | ENSMUSG00000092162,"ENSMUSG00000092162",1,0,0,0,0,0,0,0,0,0,0,0,0,0,1,0,0,0,0,0 |
| ENSMUSG00000021270,"ENSMUSG00000021270",6167,4832,5935,5831,5725,5576,5584,3959,4558,5438,5270,4417,4615,4875,4772,5030,4614,5906,3545,4672 | ENSMUSG00000088901,"ENSMUSG00000088901",0,3,7,3,11,5,8,4,6,7,6,6,10,2,8,8,6,8,4,10 | ENSMUSG00000112712,"ENSMUSG00000112712",0,0,0,0,0,0,0,0,0,0,0,0,0,0,0,0,0,0,0,0 |
| ENSMUSG00000037798,"ENSMUSG00000037798",0,0,0,1,1,0,0,3,2,0,0,0,0,0,0,2,0,0,8,5 | ENSMUSG00000062464,"ENSMUSG00000062464",29,20,24,24,14,11,23,5,10,21,30,26,14,29,22,14,15,0,7,6 | ENSMUSG00000093816,"ENSMUSG00000093816",0,0,0,0,0,0,0,0,0,0,0,0,0,0,0,0,0,0,0,0 |
| ENSMUSG00000026385,"ENSMUSG00000026385",1390,2430,3312,3584,2622,2239,2353,2170,2541,2707,3566,2613,2717,2911,2586,3050,2153,3181,2924,2789 | ENSMUSG00000104818,"ENSMUSG00000104818",2,25,18,28,18,10,12,19,19,18,27,6,8,26,16,23,17,27,7,18 | ENSMUSG00000098978,"ENSMUSG00000098978",0,0,0,0,0,0,0,0,0,1,0,3,0,0,0,0,0,0,0,0 |
| ENSMUSG00000021134,"ENSMUSG00000021134",5973,7899,9832,10371,9163,8781,8146,6871,8329,8958,10111,7939,7880,9357,7922,9530,7451,10780,7286,8019 | ENSMUSG00000089722,"ENSMUSG00000089722",41,64,2,3,2,1,1,81,52,85,3,0,8,1,1,5,1,3,3,1 | ENSMUSG00000109436,"ENSMUSG00000109436",0,0,0,0,0,0,0,0,0,0,0,0,0,0,0,0,0,0,0,0 |
| ENSMUSG00000020077,"ENSMUSG00000020077",41492,28881,35594,25214,33930,32877,27261,31313,31089,42725,26066,19125,37868,39421,29669,39112,33323,36002,22101,35270 | ENSMUSG00000115457,"ENSMUSG00000115457",4,9,17,10,9,3,6,3,2,12,18,9,10,10,6,20,13,11,5,16 | ENSMUSG00000109064,"ENSMUSG00000109064",0,0,0,0,0,0,0,0,0,0,0,0,0,0,0,0,0,0,0,0 |
| ENSMUSG00000057400,"ENSMUSG00000057400",0,0,0,0,0,0,3,1,5,0,0,0,0,0,0,0,0,0,2,2 | ENSMUSG00000083596,"ENSMUSG00000083596",4,23,13,15,17,12,21,13,13,26,16,17,13,17,14,10,12,17,6,19 | ENSMUSG00000108918,"ENSMUSG00000108918",0,0,0,0,0,0,0,0,0,0,0,0,0,0,0,0,0,0,0,0 |
| ENSMUSG00000028382,"ENSMUSG00000028382",18677,12679,14209,14836,15923,13829,13031,10781,13670,15521,12691,11530,11316,13061,11694,14819,12677,14623,8454,12200 | ENSMUSG00000022658,"ENSMUSG00000022658",1,1,2,2,0,8,3,0,0,0,0,0,1,0,0,0,0,1,0,4 | ENSMUSG00000109067,"ENSMUSG00000109067",0,0,0,0,0,0,0,0,0,0,0,0,0,0,0,0,0,0,0,0 |
| ENSMUSG00000059481,"ENSMUSG00000059481",1,1,1,0,0,0,0,2,0,0,0,0,0,0,4,1,1,1,3,3 | ENSMUSG00000097405,"ENSMUSG00000097405",14,15,13,14,25,15,21,15,20,12,16,15,8,13,18,16,9,14,13,7 | ENSMUSG00000089234,"ENSMUSG00000089234",0,0,0,0,0,0,0,0,0,0,0,0,0,0,0,0,0,0,0,0 |
| ENSMUSG00000006699,"ENSMUSG00000006699",8863,7551,9848,9077,9599,9103,8049,7428,8126,9229,9377,6895,8786,8488,7907,8741,8375,10044,6081,8425 | ENSMUSG00000024647,"ENSMUSG00000024647",2,0,2,0,0,3,1,0,1,1,3,1,5,3,1,2,0,0,1,0 | ENSMUSG00000109582,"ENSMUSG00000109582",0,0,0,0,0,0,0,0,0,0,0,0,0,0,0,0,0,0,0,0 |
| ENSMUSG00000064337,"ENSMUSG00000064337",4417,4403,4776,4852,5088,4043,4770,3554,4352,4390,4390,3437,4409,3863,4036,4226,4148,4526,3427,4425 | ENSMUSG00000026147,"ENSMUSG00000026147",32,34,51,49,29,32,46,65,45,27,44,23,44,49,47,33,50,38,51,56 | ENSMUSG00000084945,"ENSMUSG00000084945",0,0,0,3,0,0,0,0,0,0,1,0,0,0,0,0,0,0,0,0 |
| ENSMUSG00000006574,"ENSMUSG00000006574",1,1,6,0,6,16,4,7,1,9,3,2,5,12,8,9,8,8,0,8 | ENSMUSG00000069804,"ENSMUSG00000069804",8,5,8,10,3,7,9,4,4,3,5,9,6,3,11,10,2,6,10,3 | ENSMUSG00000110132,"ENSMUSG00000110132",0,0,2,0,0,0,0,0,0,0,0,0,0,0,0,0,0,2,0,0 |
| ENSMUSG00000030747,"ENSMUSG00000030747",2038,2717,3092,3833,3079,2312,3240,2736,2906,2813,3645,3227,3074,3182,3109,3317,2631,3492,2833,3277 | ENSMUSG00000027871,"ENSMUSG00000027871",0,0,0,0,0,0,0,0,0,0,0,0,0,0,0,0,0,0,0,0 | ENSMUSG00000110530,"ENSMUSG00000110530",0,0,0,0,0,0,0,0,0,0,0,0,0,0,0,0,0,0,0,0 |
| ENSMUSG00000030208,"ENSMUSG00000030208",18411,19949,22980,23695,22902,21937,22632,14681,20426,26553,20423,16982,16042,22983,17929,20296,20665,22589,13015,18167 | ENSMUSG00000116249,"ENSMUSG00000116249",1,7,9,5,13,7,10,3,5,1,7,4,9,1,2,2,3,11,1,1 | ENSMUSG00000061847,"ENSMUSG00000061847",0,0,0,0,0,0,0,0,0,0,0,0,0,0,0,0,0,0,0,0 |
| ENSMUSG00000030934,"ENSMUSG00000030934",2091,1921,2646,2528,2345,2306,2333,1911,1982,2269,2517,1780,2581,2270,2095,2122,2045,2468,1868,2168 | ENSMUSG00000100627,"ENSMUSG00000100627",0,2,0,0,0,0,2,0,2,0,0,4,1,0,3,0,0,0,0,0 | ENSMUSG00000095507,"ENSMUSG00000095507",0,0,0,0,0,0,0,0,0,0,0,0,0,0,0,0,0,0,0,0 |
| ENSMUSG00000027562,"ENSMUSG00000027562",562,747,1386,742,857,935,695,663,781,917,777,526,888,799,698,820,764,715,610,790 | ENSMUSG00000108536,"ENSMUSG00000108536",5,1,17,18,4,6,4,7,6,6,8,9,8,8,6,3,4,4,9,5 | ENSMUSG00000058568,"ENSMUSG00000058568",0,0,0,1,0,0,0,0,0,0,0,0,0,0,0,0,0,0,0,0 |
| ENSMUSG00000025203,"ENSMUSG00000025203",2582,3079,4971,4276,5820,4302,2787,4627,4180,2969,4859,3883,7588,3968,3802,4855,3792,5353,5086,5368 | ENSMUSG00000111058,"ENSMUSG00000111058",7,0,4,2,1,3,0,4,1,2,6,3,5,1,3,0,0,2,1,1 | ENSMUSG00000086289,"ENSMUSG00000086289",0,0,0,0,0,0,2,0,0,0,0,0,0,0,0,0,0,0,0,0 |
| ENSMUSG00000025497,"ENSMUSG00000025497",0,0,0,2,4,2,0,0,1,2,3,0,3,4,1,1,0,2,0,0 | ENSMUSG00000073077,"ENSMUSG00000073077",2,0,0,0,4,1,3,1,1,0,0,0,1,7,1,1,0,0,0,3 | ENSMUSG00000110172,"ENSMUSG00000110172",0,0,0,0,0,0,0,0,0,0,0,0,0,0,0,0,0,0,0,0 |
| ENSMUSG00000046352,"ENSMUSG00000046352",29272,28192,31472,35266,41995,23640,33181,31705,32772,28340,28131,31601,26357,31763,34710,43817,30415,39963,28095,34374 | ENSMUSG00000038570,"ENSMUSG00000038570",11,1,10,13,12,3,8,6,5,13,7,10,4,6,7,7,3,6,8,11 | ENSMUSG00000109996,"ENSMUSG00000109996",0,0,0,0,0,0,0,0,0,0,0,0,0,0,0,0,0,0,0,0 |
| ENSMUSG00000016534,"ENSMUSG00000016534",12987,8923,10599,10766,10890,10078,9033,8068,8936,10416,10275,7973,9290,9345,8347,10286,8904,11653,6838,8748 | ENSMUSG00000092261,"ENSMUSG00000092261",12,13,12,15,28,23,16,7,11,21,17,14,6,22,15,13,4,18,5,10 | ENSMUSG00000109614,"ENSMUSG00000109614",0,0,0,0,0,0,0,0,0,0,0,0,0,0,0,0,0,0,0,0 |
| ENSMUSG00000021922,"ENSMUSG00000021922",8,4,1,7,5,4,5,7,10,1,4,5,2,6,0,8,4,9,7,6 | ENSMUSG00000105643,"ENSMUSG00000105643",8,5,11,4,11,15,4,7,12,8,2,7,9,9,8,10,5,12,3,6 | ENSMUSG00000110130,"ENSMUSG00000110130",0,0,0,0,0,0,0,0,0,0,0,0,0,0,0,0,0,0,0,0 |
| ENSMUSG00000037805,"ENSMUSG00000037805",6521,8714,10425,9991,9049,9014,9093,6790,8400,9300,9984,8333,8393,8635,7993,10232,7781,10282,7811,8898 | ENSMUSG00000046413,"ENSMUSG00000046413",22,30,50,40,46,36,43,31,42,37,53,33,31,36,38,41,42,48,36,35 | ENSMUSG00000110314,"ENSMUSG00000110314",0,0,0,0,0,2,0,0,0,0,0,0,0,0,0,0,0,0,0,0 |
| ENSMUSG00000090733,"ENSMUSG00000090733",12097,7978,8979,8574,8035,7249,8052,5879,7479,8800,8210,7308,7046,7844,6816,8284,6595,9578,6996,8128 | ENSMUSG00000115914,"ENSMUSG00000115914",0,0,0,0,0,0,0,0,0,0,0,0,0,0,0,0,0,0,0,0 | ENSMUSG00000110335,"ENSMUSG00000110335",0,0,0,0,0,0,0,0,0,0,0,0,0,0,0,0,0,0,1,0 |
| ENSMUSG00000029064,"ENSMUSG00000029064",7569,6857,9149,9038,8684,7686,7687,6497,6920,7973,7780,6238,7446,8113,7617,8674,7512,9261,6914,7882 | ENSMUSG00000025081,"ENSMUSG00000025081",0,1,1,0,1,0,1,2,0,1,3,0,1,1,0,1,2,1,0,0 | ENSMUSG00000109977,"ENSMUSG00000109977",0,0,0,0,0,0,0,0,0,0,0,0,0,0,0,0,0,0,0,0 |
| ENSMUSG00000024066,"ENSMUSG00000024066",9734,12353,14435,20818,14425,12340,13772,13246,14014,13960,15273,12733,14104,16128,14217,16947,13520,18499,12208,14508 | ENSMUSG00000108571,"ENSMUSG00000108571",3,6,5,8,10,7,2,10,0,9,7,10,9,11,8,4,3,14,7,9 | ENSMUSG00000110168,"ENSMUSG00000110168",0,0,0,0,0,0,0,0,0,0,0,0,0,0,0,0,0,0,0,0 |
| ENSMUSG00000037563,"ENSMUSG00000037563",6403,8616,11474,10651,8591,8273,8634,7524,7998,10298,10831,9016,9314,10157,9076,10740,8319,11524,8736,9074 | ENSMUSG00000086410,"ENSMUSG00000086410",12,4,18,7,14,22,12,12,11,28,15,10,16,16,11,11,17,15,2,7 | ENSMUSG00000109912,"ENSMUSG00000109912",0,0,0,0,0,0,0,0,0,0,0,0,0,0,0,0,1,0,0,0 |
| ENSMUSG00000059291,"ENSMUSG00000059291",3818,7659,11432,9749,8156,8137,8461,6906,7259,9246,10338,7904,9036,9263,8149,9759,7735,11049,7946,9121 | ENSMUSG00000082922,"ENSMUSG00000082922",1,7,11,5,4,6,5,4,6,6,8,10,6,6,3,5,1,6,5,7 | ENSMUSG00000110281,"ENSMUSG00000110281",0,0,0,0,0,0,0,0,0,0,0,0,0,0,0,0,0,0,0,0 |
| ENSMUSG00000020460,"ENSMUSG00000020460",10820,7981,10248,9070,8972,8366,8617,6892,7773,9301,9215,7637,7542,8071,7685,9183,7629,10319,7324,8350 | ENSMUSG00000047108,"ENSMUSG00000047108",7,2,1,0,2,5,1,7,2,2,3,1,2,0,7,4,3,7,3,4 | ENSMUSG00000109966,"ENSMUSG00000109966",0,0,0,0,0,0,0,0,0,0,0,0,0,0,0,0,0,0,0,0 |
| ENSMUSG00000076258,"ENSMUSG00000076258",303,4919,8201,10336,6587,5726,8532,4961,5457,5666,19376,4326,5614,6731,4729,7153,5007,7162,3441,5944 | ENSMUSG00000116836,"ENSMUSG00000116836",6,3,8,12,1,0,8,2,3,5,19,10,3,8,4,9,5,5,7,3 | ENSMUSG00000110308,"ENSMUSG00000110308",0,0,0,0,0,0,0,0,0,0,0,0,0,0,0,0,0,0,0,0 |
| ENSMUSG00000027639,"ENSMUSG00000027639",17796,19340,22368,29774,23731,22027,20743,21714,22285,22385,24578,19774,23373,24117,20784,28638,20891,33314,17101,23065 | ENSMUSG00000060029,"ENSMUSG00000060029",1,3,15,7,4,10,7,3,9,6,7,15,6,12,5,15,10,15,3,6 | ENSMUSG00000109718,"ENSMUSG00000109718",0,0,0,0,0,0,0,0,0,0,0,0,0,0,0,0,0,0,0,0 |
| ENSMUSG00000031818,"ENSMUSG00000031818",2912,3670,5144,4595,4228,3863,4101,3392,4109,4164,4571,3684,4295,4275,3897,4635,3567,5056,4362,4399 | ENSMUSG00000062818,"ENSMUSG00000062818",0,0,0,0,0,0,0,0,0,0,0,0,0,0,0,0,0,0,0,0 | ENSMUSG00000110283,"ENSMUSG00000110283",0,0,0,0,0,0,0,0,0,0,0,0,0,0,0,0,0,0,0,0 |
| ENSMUSG00000046364,"ENSMUSG00000046364",4114,8458,10179,9770,8539,8525,8858,6849,7977,9724,9521,8092,7955,8655,7587,9931,7601,10283,8370,8801 | ENSMUSG00000085502,"ENSMUSG00000085502",0,0,0,0,1,0,1,0,0,2,0,0,1,0,0,1,0,1,0,2 | ENSMUSG00000106683,"ENSMUSG00000106683",0,0,0,0,0,0,0,0,0,1,0,0,0,0,0,0,0,0,0,0 |
| ENSMUSG00000028367,"ENSMUSG00000028367",6235,7333,13037,8524,9198,9901,7776,10698,9538,9989,9169,6835,10026,9313,9460,13952,8859,15342,10424,13567 | ENSMUSG00000115151,"ENSMUSG00000115151",0,0,2,2,0,0,4,0,1,0,4,0,1,0,2,0,0,2,0,0 | ENSMUSG00000110515,"ENSMUSG00000110515",0,0,0,0,0,0,0,0,0,0,0,0,0,0,0,0,0,0,0,0 |
| ENSMUSG00000020788,"ENSMUSG00000020788",331,480,697,457,555,626,462,571,382,478,635,373,751,529,527,540,440,835,535,530 | ENSMUSG00000097390,"ENSMUSG00000097390",16,13,9,3,11,13,5,5,3,15,5,5,16,11,3,13,4,12,6,10 | ENSMUSG00000110294,"ENSMUSG00000110294",0,0,0,0,1,0,0,0,0,0,0,0,0,1,0,0,0,2,0,0 |
| ENSMUSG00000026341,"ENSMUSG00000026341",11784,10418,12009,11979,12142,11686,11062,9135,10623,12324,11281,9329,10213,11233,10372,12588,10782,13863,8042,10925 | ENSMUSG00000048473,"ENSMUSG00000048473",63,38,49,9,36,78,48,23,35,54,28,16,40,82,33,37,23,36,19,52 | ENSMUSG00000109828,"ENSMUSG00000109828",0,0,0,0,0,0,0,0,0,0,0,0,0,0,0,2,0,0,0,0 |
| ENSMUSG00000001670,"ENSMUSG00000001670",0,0,1,0,0,1,1,2,3,0,0,2,5,0,0,1,0,0,2,4 | ENSMUSG00000115439,"ENSMUSG00000115439",43,31,41,38,55,24,30,32,25,21,40,24,25,28,35,24,20,29,24,23 | ENSMUSG00000110302,"ENSMUSG00000110302",0,0,0,0,0,0,0,0,0,0,0,0,0,0,0,0,0,0,0,0 |
| ENSMUSG00000049517,"ENSMUSG00000049517",4808,8551,11763,10518,8350,8377,7721,6971,7774,9622,10853,8741,9242,9757,8893,11199,8486,12061,7756,9633 | ENSMUSG00000095868,"ENSMUSG00000095868",5,7,8,25,21,9,12,13,11,11,9,6,9,22,14,6,12,15,7,16 | ENSMUSG00000110023,"ENSMUSG00000110023",0,0,0,0,0,0,0,0,0,0,0,0,0,0,0,0,0,1,0,0 |
| ENSMUSG00000028217,"ENSMUSG00000028217",4,9,6,4,12,8,8,2,9,8,9,7,14,7,6,8,7,13,9,15 | ENSMUSG00000073145,"ENSMUSG00000073145",2,6,6,1,2,12,2,13,4,2,10,3,4,11,7,2,1,3,1,3 | ENSMUSG00000110701,"ENSMUSG00000110701",0,1,0,0,0,0,0,0,1,0,0,0,1,0,0,0,0,0,0,0 |
| ENSMUSG00000022477,"ENSMUSG00000022477",1917,3221,4156,4139,3936,2982,3801,3288,3337,3571,4089,3171,4043,3753,3658,4113,3411,4488,3975,3794 | ENSMUSG00000077714,"ENSMUSG00000077714",19,8,22,28,21,37,13,21,24,13,30,12,19,19,17,14,11,21,12,18 | ENSMUSG00000109699,"ENSMUSG00000109699",0,0,0,0,0,0,0,0,1,0,0,0,0,0,0,0,0,0,0,0 |
| ENSMUSG00000017404,"ENSMUSG00000017404",4691,9052,11342,10345,9920,9173,9472,7159,8396,9488,10108,8080,9140,9128,8705,10841,8348,10710,9800,9532 | ENSMUSG00000038201,"ENSMUSG00000038201",20,32,39,34,36,10,28,33,35,65,34,41,55,30,51,42,49,62,42,61 | ENSMUSG00000110709,"ENSMUSG00000110709",0,0,0,0,0,0,0,0,2,0,0,0,0,0,0,0,0,0,0,0 |
| ENSMUSG00000037242,"ENSMUSG00000037242",14045,12582,14082,18321,15773,13667,14249,12181,14383,15509,12319,11262,11469,15511,12606,16617,13533,17907,9228,13519 | ENSMUSG00000066510,"ENSMUSG00000066510",14,2,15,4,14,14,15,9,9,10,24,6,14,14,10,17,10,5,8,5 | ENSMUSG00000110662,"ENSMUSG00000110662",0,0,0,0,0,0,0,0,0,0,0,0,0,0,0,0,0,0,0,0 |
| ENSMUSG00000074364,"ENSMUSG00000074364",1559,1729,2422,2417,2104,2268,2268,1937,2025,2116,2349,1638,2440,2148,2038,2200,2079,2467,2028,2352 | ENSMUSG00000084806,"ENSMUSG00000084806",2,0,2,1,1,3,4,3,4,3,0,2,6,1,1,0,1,1,2,0 | ENSMUSG00000103830,"ENSMUSG00000103830",0,0,0,0,0,0,0,0,0,0,0,0,0,0,0,0,0,0,0,0 |
| ENSMUSG00000022285,"ENSMUSG00000022285",11464,10046,11205,12610,11624,10393,11374,8589,10413,11130,10905,9473,8615,10861,9228,11668,9582,11647,7620,10029 | ENSMUSG00000085614,"ENSMUSG00000085614",5,13,13,6,9,8,2,8,4,5,5,12,16,11,4,9,5,8,6,6 | ENSMUSG00000110492,"ENSMUSG00000110492",0,0,0,0,0,0,0,0,0,0,0,0,0,0,0,0,0,0,0,0 |
| ENSMUSG00000022884,"ENSMUSG00000022884",4374,3445,3904,4162,3921,3396,3874,3037,3566,3764,4374,3262,3222,3431,3298,3400,3294,3934,2976,3170 | ENSMUSG00000087500,"ENSMUSG00000087500",0,0,0,1,0,0,0,2,0,0,0,0,0,0,0,0,0,0,0,0 | ENSMUSG00000079402,"ENSMUSG00000079402",0,0,0,0,0,0,0,0,0,0,0,0,0,0,0,0,0,0,0,0 |
| ENSMUSG00000021218,"ENSMUSG00000021218",7775,6615,8608,7593,8549,7789,6474,5866,6487,7764,7617,5654,7164,6709,6418,7714,7042,8394,5272,6929 | ENSMUSG00000097699,"ENSMUSG00000097699",2,1,9,6,4,5,1,5,1,1,10,8,2,1,6,9,8,9,3,6 | ENSMUSG00000097127,"ENSMUSG00000097127",0,0,0,0,0,0,0,0,0,0,0,0,0,0,0,0,0,0,0,0 |
| ENSMUSG00000068587,"ENSMUSG00000068587",177,180,181,155,176,347,241,234,188,259,193,63,241,291,193,194,209,395,88,181 | ENSMUSG00000087669,"ENSMUSG00000087669",0,2,8,0,1,4,1,6,1,2,3,3,2,5,1,1,2,4,4,1 | ENSMUSG00000094460,"ENSMUSG00000094460",0,0,0,0,0,0,0,0,0,0,0,0,0,0,0,0,0,0,0,0 |
| ENSMUSG00000007891,"ENSMUSG00000007891",4846,8534,11296,9695,9377,10105,10041,10333,9771,8812,10373,7399,12504,9357,8821,11096,8265,13120,11075,12478 | ENSMUSG00000069305,"ENSMUSG00000069305",6,4,3,5,5,6,8,1,0,1,2,0,7,6,2,3,4,3,5,3 | ENSMUSG00000096828,"ENSMUSG00000096828",0,0,0,0,0,0,0,0,0,0,0,0,0,0,0,0,0,0,0,0 |
| ENSMUSG00000021477,"ENSMUSG00000021477",11332,15747,22932,19990,18147,14543,14734,16277,16626,18177,19160,12034,20736,18488,15697,19226,15958,19969,14942,19837 | ENSMUSG00000024530,"ENSMUSG00000024530",28,22,34,20,26,29,20,31,13,21,23,13,25,21,17,13,20,25,14,20 | ENSMUSG00000090627,"ENSMUSG00000090627",0,0,0,0,0,0,0,0,0,0,0,0,0,0,0,0,0,0,0,0 |
| ENSMUSG00000024659,"ENSMUSG00000024659",24094,18985,22091,17908,21091,20396,21081,13444,19254,27190,16070,15758,14353,22153,17481,22467,17559,24499,11893,19745 | ENSMUSG00000106927,"ENSMUSG00000106927",4,7,1,8,9,8,7,5,5,4,6,6,6,7,6,6,7,9,5,11 | ENSMUSG00000114611,"ENSMUSG00000114611",0,0,0,0,0,0,0,0,0,0,0,0,0,0,0,0,0,0,0,0 |
| ENSMUSG00000050359,"ENSMUSG00000050359",25872,29977,29910,41720,35476,25388,40802,28855,38368,32342,30608,35052,27708,32377,34768,43099,32577,42351,31064,39827 | ENSMUSG00000085803,"ENSMUSG00000085803",0,0,0,0,0,0,0,1,0,0,0,0,0,0,3,0,1,0,0,2 | ENSMUSG00000091676,"ENSMUSG00000091676",0,0,0,0,0,0,0,0,0,0,0,0,0,0,0,0,0,0,0,0 |
| ENSMUSG00000054452,"ENSMUSG00000054452",870,2126,3340,2488,2469,2443,2171,2279,1974,2366,2823,1958,3100,2352,2382,2799,2302,3241,2827,2797 | ENSMUSG00000000632,"ENSMUSG00000000632",0,0,1,0,0,0,0,1,0,0,0,0,0,0,0,0,0,1,0,0 | ENSMUSG00000096579,"ENSMUSG00000096579",0,0,0,0,0,0,0,0,0,0,0,0,0,0,0,0,0,0,0,0 |
| ENSMUSG00000030168,"ENSMUSG00000030168",2209,3005,3116,3968,3505,2985,3254,2588,3012,3257,3805,3332,3348,3394,3136,3597,3024,3883,2774,2904 | ENSMUSG00000111933,"ENSMUSG00000111933",13,32,35,20,34,31,37,14,42,43,30,19,39,44,36,40,26,38,25,30 | ENSMUSG00000049460,"ENSMUSG00000049460",0,0,0,0,0,0,0,0,0,0,0,0,0,0,0,0,0,0,0,0 |
| ENSMUSG00000023150,"ENSMUSG00000023150",4667,4087,4456,5079,5141,3881,4177,3069,4004,4214,4929,4371,3781,4372,3883,4019,3738,4414,3057,3232 | ENSMUSG00000085442,"ENSMUSG00000085442",3,8,5,10,4,10,7,4,15,11,10,8,5,8,6,10,11,7,7,11 | ENSMUSG00000089833,"ENSMUSG00000089833",0,0,0,0,0,0,0,0,0,0,0,0,0,0,0,0,0,0,0,0 |
| ENSMUSG00000008845,"ENSMUSG00000008845",23134,24693,47277,36286,35494,35037,24090,22095,24704,32889,31716,19094,28210,33463,27627,25562,26021,23942,18560,21466 | ENSMUSG00000115980,"ENSMUSG00000115980",0,5,11,10,6,4,2,1,8,3,1,1,9,2,3,8,9,2,13,5 | ENSMUSG00000072707,"ENSMUSG00000072707",0,0,0,0,0,0,0,0,0,0,0,0,0,0,0,0,1,0,0,0 |
| ENSMUSG00000026234,"ENSMUSG00000026234",9702,12566,14285,15980,13131,12624,12844,8600,11317,13871,14915,12366,9692,12287,11095,13216,11083,13684,9265,10973 | ENSMUSG00000090439,"ENSMUSG00000090439",17,27,46,22,37,29,31,55,33,33,36,27,51,31,35,36,41,43,23,46 | ENSMUSG00000084722,"ENSMUSG00000084722",0,0,0,0,0,0,0,0,0,0,0,0,0,0,0,0,0,0,0,0 |
| ENSMUSG00000022564,"ENSMUSG00000022564",5014,8271,10658,8100,9695,10187,8869,10854,8951,10638,9050,6574,12593,11122,9295,12088,9376,12862,8146,12549 | ENSMUSG00000110690,"ENSMUSG00000110690",0,0,3,4,0,1,1,0,1,0,2,1,5,0,1,3,2,3,1,1 | ENSMUSG00000114877,"ENSMUSG00000114877",0,0,0,0,0,0,0,0,0,0,0,0,1,0,0,0,0,0,0,0 |
| ENSMUSG00000078578,"ENSMUSG00000078578",9552,8960,10307,10847,10844,9859,10117,7631,9748,10983,9934,8380,8792,9529,8624,10356,9101,11319,7025,9424 | ENSMUSG00000081249,"ENSMUSG00000081249",10,11,14,13,16,15,11,13,9,12,9,15,5,12,18,10,16,28,12,5 | ENSMUSG00000059827,"ENSMUSG00000059827",0,0,0,0,0,0,0,0,0,0,0,0,0,0,0,0,0,0,0,0 |
| ENSMUSG00000024740,"ENSMUSG00000024740",4243,5281,7415,6789,6363,6616,6493,5318,5915,5780,6973,5382,6388,6107,5885,6451,5574,7013,5389,6199 | ENSMUSG00000104279,"ENSMUSG00000104279",0,6,9,3,14,5,6,7,9,6,13,4,5,3,11,4,2,9,2,7 | ENSMUSG00000109849,"ENSMUSG00000109849",0,0,0,0,0,0,0,0,0,0,0,0,0,0,0,0,0,0,0,0 |
| ENSMUSG00000071415,"ENSMUSG00000071415",4247,7806,10323,9923,7948,7918,7805,6406,7360,8589,10150,8213,7935,8902,7433,8834,7722,9709,7798,8503 | ENSMUSG00000082676,"ENSMUSG00000082676",42,21,19,35,34,18,17,19,22,4,18,14,6,27,19,16,23,18,5,9 | ENSMUSG00000114774,"ENSMUSG00000114774",0,0,0,0,0,1,0,0,0,1,0,2,0,0,0,0,0,0,0,0 |
| ENSMUSG00000048578,"ENSMUSG00000048578",2051,2167,3474,3014,2937,3029,2763,2810,2365,2540,3382,2190,3741,2738,2893,2818,2551,3377,2759,3004 | ENSMUSG00000021919,"ENSMUSG00000021919",0,2,0,1,2,0,0,0,2,0,0,0,0,0,0,0,0,0,0,2 | ENSMUSG00000115311,"ENSMUSG00000115311",0,0,0,0,0,0,0,1,0,0,0,0,0,0,0,0,0,0,0,0 |
| ENSMUSG00000025261,"ENSMUSG00000025261",6198,6597,7288,8170,7785,6762,7429,5470,6639,6697,7400,6325,6195,6876,6123,7231,6381,8012,5340,5925 | ENSMUSG00000113816,"ENSMUSG00000113816",6,3,14,5,7,10,7,7,6,9,18,6,15,4,6,10,4,6,2,15 | ENSMUSG00000096405,"ENSMUSG00000096405",0,0,0,0,0,0,0,0,0,0,0,0,0,0,0,0,0,0,0,0 |
| ENSMUSG00000017344,"ENSMUSG00000017344",13,20,31,27,29,23,29,48,23,24,26,19,46,30,31,25,39,35,25,33 | ENSMUSG00000116238,"ENSMUSG00000116238",0,2,3,0,2,0,3,4,0,1,4,0,0,2,0,2,0,1,1,0 | ENSMUSG00000114933,"ENSMUSG00000114933",0,0,0,0,0,0,0,0,0,0,0,0,0,0,0,0,0,0,0,0 |
| ENSMUSG00000020190,"ENSMUSG00000020190",1477,2541,3680,2637,2896,2885,2408,2835,2633,2946,3217,2185,4010,2920,2820,3388,2892,3649,2796,3251 | ENSMUSG00000049173,"ENSMUSG00000049173",21,33,68,38,39,14,43,36,47,42,49,22,72,47,53,36,53,78,46,40 | ENSMUSG00000091216,"ENSMUSG00000091216",1,0,2,1,0,0,0,0,0,0,0,0,0,0,0,0,0,0,0,0 |
| ENSMUSG00000024359,"ENSMUSG00000024359",4421,5743,6475,6998,6062,5430,6216,4304,5525,5841,6954,5259,5254,5493,5393,6109,5109,6283,4869,5380 | ENSMUSG00000103089,"ENSMUSG00000103089",2,15,30,2,9,37,23,17,18,38,18,9,30,26,22,19,19,28,10,24 | ENSMUSG00000091718,"ENSMUSG00000091718",0,0,0,0,0,0,0,0,0,0,0,0,0,0,0,0,0,0,0,0 |
| ENSMUSG00000001552,"ENSMUSG00000001552",12554,17062,19778,24295,20724,15307,20482,14918,19474,17702,19722,20075,16919,18465,17382,22311,16494,23014,16114,18372 | ENSMUSG00000097852,"ENSMUSG00000097852",0,0,1,4,3,0,2,1,0,4,3,0,1,3,2,1,0,0,0,3 | ENSMUSG00000072598,"ENSMUSG00000072598",1,0,0,1,0,0,0,0,1,0,0,0,0,0,0,0,0,1,0,0 |
| ENSMUSG00000025016,"ENSMUSG00000025016",5987,5158,6800,6108,6245,6123,5436,4380,5147,5970,6262,4761,5557,5594,5199,5467,5189,6400,4033,4802 | ENSMUSG00000116284,"ENSMUSG00000116284",0,0,0,0,0,0,0,0,0,0,0,2,0,1,0,0,0,0,0,0 | ENSMUSG00000079265,"ENSMUSG00000079265",0,0,0,0,0,0,0,0,0,0,0,0,0,1,0,0,0,0,0,1 |
| ENSMUSG00000025794,"ENSMUSG00000025794",7770,8151,10669,10156,9061,8367,8669,6489,7530,8530,10046,8156,8510,8981,8225,9983,7734,10586,8393,8701 | ENSMUSG00000085783,"ENSMUSG00000085783",3,7,8,11,7,14,12,5,7,8,9,9,4,13,8,5,7,12,1,8 | ENSMUSG00000085866,"ENSMUSG00000085866",0,0,0,0,0,0,0,0,0,0,0,0,0,0,0,0,0,0,0,0 |
| ENSMUSG00000020029,"ENSMUSG00000020029",2892,2864,3430,2966,3384,3483,3211,2714,3240,4041,3110,2239,3407,3533,2723,3890,3050,3991,2067,3360 | ENSMUSG00000103574,"ENSMUSG00000103574",0,2,2,7,4,2,7,7,2,0,9,5,6,3,6,6,7,3,4,3 | ENSMUSG00000085380,"ENSMUSG00000085380",0,0,0,0,0,0,0,0,1,0,0,0,0,0,0,0,0,0,0,0 |
| ENSMUSG00000050335,"ENSMUSG00000050335",7280,11733,15198,15353,13791,11743,12180,11060,11529,14883,13552,10155,14125,14207,12128,14155,13766,15821,11820,13688 | ENSMUSG00000086494,"ENSMUSG00000086494",0,0,2,0,0,1,0,0,1,0,0,0,0,0,0,0,0,0,0,1 | ENSMUSG00000115617,"ENSMUSG00000115617",0,0,0,0,0,0,0,0,0,0,0,0,0,0,0,0,0,0,0,0 |
| ENSMUSG00000032562,"ENSMUSG00000032562",5717,6823,10250,7578,9128,9315,6948,7542,6768,9001,8580,5632,10267,8197,7858,8918,8028,10816,6729,8592 | ENSMUSG00000039977,"ENSMUSG00000039977",0,1,1,2,7,2,1,3,0,0,3,3,2,0,2,2,0,0,0,1 | ENSMUSG00000115310,"ENSMUSG00000115310",0,0,0,0,0,0,0,0,0,0,0,0,0,1,0,1,0,0,0,0 |
| ENSMUSG00000069922,"ENSMUSG00000069922",0,0,0,0,0,0,0,4,3,0,0,0,0,0,0,0,0,0,3,0 | ENSMUSG00000058728,"ENSMUSG00000058728",3,7,5,2,5,5,5,7,6,8,16,3,5,8,8,8,6,11,9,9 | ENSMUSG00000115323,"ENSMUSG00000115323",0,0,0,0,0,0,0,0,0,0,0,0,0,0,0,0,0,0,0,0 |
| ENSMUSG00000019987,"ENSMUSG00000019987",12629,13031,18102,15410,16900,11555,10586,20489,14775,19037,17609,10246,23076,20562,18063,19086,21914,17823,13088,19899 | ENSMUSG00000105105,"ENSMUSG00000105105",1,3,1,2,3,0,0,2,3,1,3,2,2,1,1,0,0,2,0,0 | ENSMUSG00000091296,"ENSMUSG00000091296",0,0,0,0,0,0,0,0,0,0,0,0,0,0,0,0,0,0,0,0 |
| ENSMUSG00000027447,"ENSMUSG00000027447",6240,8406,10973,10647,9540,9016,8768,8368,9128,10243,10565,7298,10788,9684,8462,10739,9209,10594,9665,9423 | ENSMUSG00000030887,"ENSMUSG00000030887",1,3,7,8,6,8,5,7,4,2,8,5,1,3,2,6,4,10,10,2 | ENSMUSG00000090740,"ENSMUSG00000090740",0,0,0,0,0,0,0,0,0,0,0,0,0,0,0,0,0,0,0,0 |
| ENSMUSG00000030739,"ENSMUSG00000030739",431,701,718,1288,778,552,955,486,785,715,893,880,607,859,686,763,629,752,654,678 | ENSMUSG00000116605,"ENSMUSG00000116605",29,22,42,25,34,50,28,21,10,24,20,15,22,21,19,29,20,36,22,23 | ENSMUSG00000104598,"ENSMUSG00000104598",0,0,0,0,0,0,1,0,0,0,0,0,0,0,0,0,0,0,0,0 |
| ENSMUSG00000074227,"ENSMUSG00000074227",1408,1594,2115,2175,1875,1538,1837,1635,1643,1599,2168,1837,1945,1922,1967,2321,1730,2451,2075,2013 | ENSMUSG00000039720,"ENSMUSG00000039720",0,0,0,0,0,0,0,0,0,0,0,0,0,0,0,0,0,0,0,0 | ENSMUSG00000104673,"ENSMUSG00000104673",0,0,0,0,0,3,0,0,0,0,0,0,0,0,0,0,0,0,0,0 |
| ENSMUSG00000047281,"ENSMUSG00000047281",16353,27498,31819,37940,30516,25118,34085,22365,31128,28966,28304,28630,21878,30468,27172,34165,23971,34032,29669,29147 | ENSMUSG00000112550,"ENSMUSG00000112550",4,8,12,12,11,15,10,6,6,11,6,7,14,8,10,11,11,15,10,14 | ENSMUSG00000105823,"ENSMUSG00000105823",0,0,0,0,0,0,0,0,0,0,0,1,0,0,0,0,0,0,0,0 |
| ENSMUSG00000030364,"ENSMUSG00000030364",0,0,1,1,0,0,1,0,0,0,0,0,0,0,1,0,0,0,0,0 | ENSMUSG00000022303,"ENSMUSG00000022303",4,7,7,14,7,6,7,7,5,3,10,11,10,9,13,8,15,14,11,20 | ENSMUSG00000092232,"ENSMUSG00000092232",0,1,0,0,0,0,0,0,0,0,0,0,0,0,0,0,0,0,0,0 |
| ENSMUSG00000053522,"ENSMUSG00000053522",17120,29783,37884,41485,30931,27267,27980,24655,27598,32767,38366,32746,32845,31946,31634,40449,26860,42472,41696,34156 | ENSMUSG00000108053,"ENSMUSG00000108053",7,6,8,15,10,14,3,10,4,6,3,9,11,1,7,12,4,8,4,9 | ENSMUSG00000100162,"ENSMUSG00000100162",0,0,0,1,0,0,0,0,0,0,1,0,0,0,0,0,0,0,0,0 |
| ENSMUSG00000059005,"ENSMUSG00000059005",4766,8039,10416,11422,9254,8835,8670,7759,7927,9295,11213,9476,9150,9482,9765,9490,7979,12422,9447,8576 | ENSMUSG00000107944,"ENSMUSG00000107944",1,8,11,8,5,8,8,4,1,5,13,10,11,2,3,11,4,6,16,6 | ENSMUSG00000115232,"ENSMUSG00000115232",0,0,0,0,0,0,0,0,0,0,0,1,0,0,0,0,0,0,0,0 |
| ENSMUSG00000018830,"ENSMUSG00000018830",107,240,239,298,250,260,305,287,217,249,322,147,316,265,312,240,259,333,373,308 | ENSMUSG00000058934,"ENSMUSG00000058934",7,12,10,7,8,8,5,3,7,2,3,1,5,3,8,3,2,15,10,7 | ENSMUSG00000115757,"ENSMUSG00000115757",0,0,0,0,0,0,0,0,0,0,0,0,0,0,0,0,0,0,0,0 |
| ENSMUSG00000025132,"ENSMUSG00000025132",4471,7207,10033,8938,9101,8988,8195,7166,7871,8940,8751,6731,8493,8923,7774,8940,7900,10069,8116,8327 | ENSMUSG00000101037,"ENSMUSG00000101037",0,3,6,3,4,4,0,0,5,5,5,2,5,1,6,4,0,2,3,2 | ENSMUSG00000094250,"ENSMUSG00000094250",0,0,0,0,0,0,0,0,0,0,0,0,0,0,0,0,0,0,0,0 |
| ENSMUSG00000028452,"ENSMUSG00000028452",6315,6599,8697,8696,8326,7533,8087,6656,7003,7131,8667,6517,7950,7580,7669,8261,6923,9381,6704,7837 | ENSMUSG00000046005,"ENSMUSG00000046005",0,0,0,0,0,0,0,0,1,0,1,0,0,0,0,0,0,0,0,0 | ENSMUSG00000072068,"ENSMUSG00000072068",0,0,0,0,0,0,0,0,0,0,0,0,0,0,0,0,0,0,0,0 |
| ENSMUSG00000023247,"ENSMUSG00000023247",52,38,16,51,27,36,50,17,29,30,32,70,16,24,22,39,36,25,10,11 | ENSMUSG00000098447,"ENSMUSG00000098447",4,3,6,2,12,7,4,8,1,3,3,4,9,5,2,4,2,5,5,6 | ENSMUSG00000114948,"ENSMUSG00000114948",0,0,0,0,0,1,0,0,1,1,0,1,0,0,0,0,0,0,0,0 |
| ENSMUSG00000035242,"ENSMUSG00000035242",3693,4232,6434,5309,5501,5591,5379,4938,4820,5523,5668,4118,6173,5297,4702,5868,4740,6822,4490,5880 | ENSMUSG00000085563,"ENSMUSG00000085563",9,12,12,9,18,21,18,9,10,11,12,15,3,16,13,20,7,20,19,6 | ENSMUSG00000114920,"ENSMUSG00000114920",0,0,0,0,0,0,0,0,0,0,0,0,0,0,0,0,0,0,0,0 |
| ENSMUSG00000041028,"ENSMUSG00000041028",4567,3562,4410,4898,4078,3617,3995,3362,3792,4199,4447,3642,3960,4062,3786,4128,3724,4737,3472,3818 | ENSMUSG00000086670,"ENSMUSG00000086670",0,0,1,0,0,0,2,1,0,0,0,1,0,1,6,1,0,8,0,1 | ENSMUSG00000064442,"ENSMUSG00000064442",0,0,0,0,0,0,0,0,0,0,0,0,0,0,0,0,0,0,0,0 |
| ENSMUSG00000021282,"ENSMUSG00000021282",11087,9224,8967,10751,9653,8994,9694,6418,9303,10348,9438,8123,6987,9289,7960,9339,8489,9298,5512,7463 | ENSMUSG00000094595,"ENSMUSG00000094595",9,3,11,6,2,2,7,4,6,6,5,3,5,3,9,8,1,1,6,6 | ENSMUSG00000114829,"ENSMUSG00000114829",0,0,0,0,0,0,0,0,0,0,0,0,0,0,0,0,0,2,0,0 |
| ENSMUSG00000029772,"ENSMUSG00000029772",1357,1255,1696,1649,1567,1613,1490,1147,1252,1520,1555,1149,1273,1383,1411,1480,1481,1499,1084,1313 | ENSMUSG00000095668,"ENSMUSG00000095668",1,7,6,3,7,3,2,3,2,1,12,4,16,4,12,7,9,10,10,5 | ENSMUSG00000115139,"ENSMUSG00000115139",0,0,0,0,0,0,0,0,0,0,0,0,0,0,0,0,0,0,0,0 |
| ENSMUSG00000009927,"ENSMUSG00000009927",4533,7865,9658,9244,8949,8392,8577,6198,8021,9105,9009,7939,7698,8349,7352,9068,7293,9592,7036,7777 | ENSMUSG00000031297,"ENSMUSG00000031297",19,10,16,7,7,10,9,13,10,13,17,11,20,13,12,6,8,9,7,15 | ENSMUSG00000115470,"ENSMUSG00000115470",0,0,0,0,0,0,0,0,0,0,0,0,1,0,0,0,0,0,0,0 |
| ENSMUSG00000036752,"ENSMUSG00000036752",4722,6090,6938,7428,6768,5618,7430,4842,6258,6273,6079,5766,5222,6568,5963,6986,5620,7328,6066,6543 | ENSMUSG00000052769,"ENSMUSG00000052769",7,10,4,4,8,3,13,7,5,16,2,4,5,2,3,4,13,7,2,6 | ENSMUSG00000115427,"ENSMUSG00000115427",0,0,0,0,0,0,0,0,0,0,0,0,0,0,0,0,0,0,0,0 |
| ENSMUSG00000010277,"ENSMUSG00000010277",5020,5145,6583,7289,6915,5805,5751,5158,5593,5410,6662,4995,6017,5647,5691,6703,5450,7426,5104,5753 | ENSMUSG00000097150,"ENSMUSG00000097150",7,7,3,1,8,6,3,2,1,11,14,3,0,5,10,6,0,6,10,5 | ENSMUSG00000115271,"ENSMUSG00000115271",0,0,0,0,0,0,0,0,0,0,0,0,0,0,0,0,0,0,0,0 |
| ENSMUSG00000025278,"ENSMUSG00000025278",4918,6597,7864,8897,8272,5998,8875,6466,7634,6878,6237,6318,5264,8053,7118,8778,7014,8906,6214,7359 | ENSMUSG00000021081,"ENSMUSG00000021081",0,0,0,0,0,0,0,0,0,0,0,0,0,0,0,0,0,0,0,0 | ENSMUSG00000115306,"ENSMUSG00000115306",0,0,0,0,0,0,2,0,0,0,0,0,0,0,0,0,0,0,0,0 |
| ENSMUSG00000020456,"ENSMUSG00000020456",2372,2621,3209,3571,3253,2666,3548,2778,3051,2987,3526,2498,3060,3089,2916,3151,2783,3275,3124,2870 | ENSMUSG00000026940,"ENSMUSG00000026940",1,4,6,8,4,6,5,7,9,8,5,0,4,2,11,1,0,1,3,1 | ENSMUSG00000115471,"ENSMUSG00000115471",0,0,0,0,0,0,0,0,0,0,0,0,0,0,0,0,0,0,0,0 |
| ENSMUSG00000025745,"ENSMUSG00000025745",2155,3193,4008,3770,3426,3179,3594,3160,3125,3680,3809,2923,3607,3492,3320,3830,3232,4125,3476,3599 | ENSMUSG00000078897,"ENSMUSG00000078897",3,6,2,3,6,0,1,2,7,2,4,3,2,2,6,7,2,4,6,11 | ENSMUSG00000115435,"ENSMUSG00000115435",0,0,0,0,0,0,0,0,0,0,0,0,0,0,0,0,0,0,0,0 |
| ENSMUSG00000009185,"ENSMUSG00000009185",21406,27273,46258,32923,32898,28124,29519,30227,30958,34211,37242,21890,35805,30845,30505,37069,31509,35675,31876,32605 | ENSMUSG00000027971,"ENSMUSG00000027971",0,0,0,0,0,0,0,0,0,0,0,0,0,0,0,2,0,0,0,0 | ENSMUSG00000115320,"ENSMUSG00000115320",0,0,0,0,0,0,0,0,0,0,0,0,0,0,0,0,0,0,0,0 |
| ENSMUSG00000024029,"ENSMUSG00000024029",0,1,0,0,0,0,0,0,0,1,0,1,2,0,0,0,0,0,0,0 | ENSMUSG00000106717,"ENSMUSG00000106717",13,18,7,7,10,1,10,5,7,5,8,18,4,7,3,15,11,19,4,12 | ENSMUSG00000115515,"ENSMUSG00000115515",0,0,0,0,0,0,0,0,0,0,0,0,0,0,0,0,0,0,0,0 |
| ENSMUSG00000025503,"ENSMUSG00000025503",2075,2293,3081,2416,2580,2655,2650,2489,2309,2725,2709,1938,3083,2728,2677,2973,2491,3321,2197,2929 | ENSMUSG00000067338,"ENSMUSG00000067338",2,0,0,0,0,0,0,0,0,0,0,0,0,0,0,0,0,0,0,0 | ENSMUSG00000090284,"ENSMUSG00000090284",0,0,0,0,0,0,0,0,0,0,0,0,0,1,0,0,0,0,0,0 |
| ENSMUSG00000022037,"ENSMUSG00000022037",937,1297,1464,2212,1462,1158,1624,1263,1660,1191,1679,1460,1453,1516,1210,1454,1092,1492,1334,1693 | ENSMUSG00000085009,"ENSMUSG00000085009",0,2,5,2,2,3,4,0,2,2,5,5,4,4,4,2,3,3,0,0 | ENSMUSG00000110425,"ENSMUSG00000110425",0,0,0,0,0,0,0,0,0,0,0,0,0,0,0,0,0,0,0,0 |
| ENSMUSG00000063457,"ENSMUSG00000063457",2105,6293,8458,7484,6331,6348,6375,5643,5930,6694,7686,6290,7057,6756,6263,8077,6107,8197,7411,7080 | ENSMUSG00000108618,"ENSMUSG00000108618",8,5,6,2,2,5,4,2,0,1,0,3,7,5,3,5,5,7,1,4 | ENSMUSG00000111648,"ENSMUSG00000111648",0,0,1,0,0,0,1,0,0,0,0,0,0,0,0,1,1,0,0,0 |
| ENSMUSG00000036606,"ENSMUSG00000036606",7576,8538,10265,10966,10181,8741,10645,8514,9125,9083,9689,8179,9748,9547,9743,10677,9004,11954,8748,9703 | ENSMUSG00000090709,"ENSMUSG00000090709",8,20,31,4,21,23,15,13,21,23,10,8,16,15,23,15,11,24,11,11 | ENSMUSG00000111503,"ENSMUSG00000111503",0,0,0,0,0,0,0,0,0,0,0,0,0,0,0,0,0,0,0,0 |
| ENSMUSG00000028788,"ENSMUSG00000028788",7324,5587,6087,6565,6671,5420,6027,4474,5596,6301,6101,5466,5113,5529,5303,6105,5598,6636,4220,5760 | ENSMUSG00000111840,"ENSMUSG00000111840",9,3,5,11,7,1,2,2,2,0,10,5,9,2,5,5,9,2,4,3 | ENSMUSG00000111612,"ENSMUSG00000111612",0,0,0,0,0,0,0,0,2,0,0,0,0,0,0,0,0,0,1,0 |
| ENSMUSG00000031924,"ENSMUSG00000031924",2313,2187,2787,2927,2819,2447,2432,2033,2357,2297,2747,2203,2396,2379,2437,2710,2305,3068,2123,2312 | ENSMUSG00000053263,"ENSMUSG00000053263",1,5,8,4,8,1,3,6,0,5,4,6,6,4,4,4,4,2,3,3 | ENSMUSG00000077384,"ENSMUSG00000077384",0,0,0,0,1,0,0,0,0,0,0,0,0,2,0,0,0,0,0,0 |
| ENSMUSG00000074768,"ENSMUSG00000074768",1,9,6,6,6,8,7,5,3,7,12,8,4,6,0,6,4,2,16,3 | ENSMUSG00000000202,"ENSMUSG00000000202",33,38,49,70,51,20,47,25,44,31,28,44,14,32,28,34,21,23,41,15 | ENSMUSG00000110882,"ENSMUSG00000110882",0,0,0,2,0,0,0,0,0,0,0,0,0,1,1,0,0,0,0,0 |
| ENSMUSG00000024712,"ENSMUSG00000024712",915,894,1037,989,1118,951,821,671,913,1023,1043,744,821,892,746,882,893,931,625,786 | ENSMUSG00000114598,"ENSMUSG00000114598",7,16,21,16,24,17,16,8,24,30,13,12,14,22,9,22,14,20,10,20 | ENSMUSG00000110786,"ENSMUSG00000110786",0,0,0,0,0,0,0,0,0,2,0,0,0,0,0,0,0,0,0,0 |
| ENSMUSG00000026553,"ENSMUSG00000026553",5263,5137,6531,7052,6768,5938,6256,5234,5777,5946,6556,4979,6026,6057,5748,6297,5601,6915,4817,6002 | ENSMUSG00000084989,"ENSMUSG00000084989",17,17,30,26,23,16,27,24,19,27,34,9,40,25,15,16,26,30,13,41 | ENSMUSG00000111161,"ENSMUSG00000111161",0,0,0,0,0,0,0,0,0,0,0,0,0,0,0,0,0,0,0,0 |
| ENSMUSG00000020152,"ENSMUSG00000020152",12780,9122,11357,11248,11722,10573,9369,8542,9111,11022,10307,7999,9792,9977,9313,11479,9886,12160,6696,9501 | ENSMUSG00000103115,"ENSMUSG00000103115",7,5,10,6,5,13,2,2,3,12,15,10,13,9,3,3,3,8,9,3 | ENSMUSG00000051414,"ENSMUSG00000051414",0,0,0,0,0,0,0,0,0,0,0,0,0,0,0,0,0,0,0,0 |
| ENSMUSG00000026715,"ENSMUSG00000026715",3,8,1,9,5,3,3,5,17,5,19,8,5,4,12,7,2,2,5,15 | ENSMUSG00000038276,"ENSMUSG00000038276",3,6,11,10,9,5,9,7,9,8,15,6,8,3,5,5,7,12,3,8 | ENSMUSG00000086016,"ENSMUSG00000086016",0,0,0,0,0,0,1,0,0,0,0,0,0,0,0,0,0,0,0,0 |
| ENSMUSG00000005103,"ENSMUSG00000005103",8611,8672,10274,10546,9820,9682,10277,7738,8887,9898,9348,7679,8245,10284,8677,10027,8659,10518,6757,8647 | ENSMUSG00000106103,"ENSMUSG00000106103",3,1,1,2,6,2,4,1,2,2,6,10,2,6,1,2,0,1,0,2 | ENSMUSG00000089909,"ENSMUSG00000089909",0,0,0,0,0,0,0,0,0,0,0,0,0,0,0,0,0,0,0,0 |
| ENSMUSG00000035493,"ENSMUSG00000035493",13884,19993,25087,21858,23555,24385,20300,20500,21710,24181,22894,15114,27782,24610,20810,24374,23764,26215,18608,23880 | ENSMUSG00000112203,"ENSMUSG00000112203",6,0,4,5,5,5,2,9,2,2,8,5,6,2,2,3,3,4,6,5 | ENSMUSG00000087531,"ENSMUSG00000087531",0,0,0,0,0,0,0,2,0,0,0,0,0,0,0,0,0,0,0,0 |
| ENSMUSG00000028356,"ENSMUSG00000028356",7,8,6,6,10,5,9,17,4,8,6,4,13,5,11,10,5,7,3,11 | ENSMUSG00000110433,"ENSMUSG00000110433",13,14,23,19,20,16,21,18,15,22,21,11,11,22,11,9,9,20,15,10 | ENSMUSG00000111413,"ENSMUSG00000111413",0,0,0,0,0,0,1,0,0,1,0,1,0,0,0,0,0,0,0,1 |
| ENSMUSG00000028763,"ENSMUSG00000028763",2159,3064,4652,3929,3888,5143,3861,3779,3402,3926,4209,2488,4711,3821,3667,4104,4006,5270,3127,3765 | ENSMUSG00000111605,"ENSMUSG00000111605",13,11,9,12,10,14,16,13,6,14,16,19,14,12,14,13,5,26,8,15 | ENSMUSG00000111153,"ENSMUSG00000111153",0,0,0,0,0,0,0,0,0,0,0,0,0,0,0,0,3,0,0,0 |
| ENSMUSG00000030057,"ENSMUSG00000030057",8868,7102,7790,8715,7333,6618,7325,5369,6569,7521,8580,6812,6466,6896,6359,7107,6131,7695,5542,6360 | ENSMUSG00000025789,"ENSMUSG00000025789",4,8,8,4,6,11,1,7,10,4,10,4,13,1,6,3,6,2,15,5 | ENSMUSG00000111645,"ENSMUSG00000111645",0,0,0,0,0,0,0,0,0,0,0,0,0,0,0,0,0,0,0,0 |
| ENSMUSG00000020849,"ENSMUSG00000020849",6917,6386,7570,7752,7587,7058,7468,5924,6563,7330,7602,6133,6390,6976,6356,7081,6572,8030,5901,6983 | ENSMUSG00000112569,"ENSMUSG00000112569",0,0,0,0,0,0,0,0,0,0,0,0,0,0,0,0,0,0,0,0 | ENSMUSG00000111467,"ENSMUSG00000111467",0,0,0,0,0,0,0,0,0,0,0,1,0,0,1,0,0,0,2,0 |
| ENSMUSG00000001506,"ENSMUSG00000001506",9159,11635,21800,11498,12267,34772,15203,19508,13843,14372,16062,9310,27590,13012,17145,16204,16988,22380,10509,19008 | ENSMUSG00000090071,"ENSMUSG00000090071",0,0,0,3,0,1,0,0,4,0,0,1,0,0,0,2,0,0,0,0 | ENSMUSG00000111797,"ENSMUSG00000111797",0,0,0,0,0,0,0,0,0,0,0,0,0,0,0,0,0,0,0,0 |
| ENSMUSG00000020585,"ENSMUSG00000020585",6920,5525,7352,7539,7084,6381,6149,5963,6258,6595,7281,5657,7036,6493,6066,7149,5882,8347,5450,6833 | ENSMUSG00000029219,"ENSMUSG00000029219",0,0,0,0,4,1,2,2,0,0,0,1,2,0,1,0,1,1,1,2 | ENSMUSG00000097946,"ENSMUSG00000097946",0,0,0,0,1,0,0,0,0,0,0,0,0,0,0,0,0,1,0,0 |
| ENSMUSG00000015837,"ENSMUSG00000015837",3077,4156,5712,4777,4705,4473,4640,4346,4536,5065,4560,3664,5360,4802,4286,5294,4322,5945,3982,5126 | ENSMUSG00000061578,"ENSMUSG00000061578",1,4,4,6,5,2,7,3,4,6,9,2,8,0,3,7,11,11,5,7 | ENSMUSG00000057444,"ENSMUSG00000057444",0,0,0,0,0,0,0,0,0,0,3,0,0,0,0,0,0,1,0,0 |
| ENSMUSG00000001525,"ENSMUSG00000001525",5575,7064,9937,8087,8528,9203,8966,7935,7975,7842,8895,6362,8938,8086,7729,8663,7132,9849,8220,9731 | ENSMUSG00000093458,"ENSMUSG00000093458",1,3,1,7,1,0,6,0,5,4,5,1,8,3,2,5,0,1,1,0 | ENSMUSG00000043331,"ENSMUSG00000043331",0,0,0,0,0,0,0,0,0,0,0,0,0,0,0,0,0,0,0,0 |
| ENSMUSG00000021190,"ENSMUSG00000021190",12847,17780,29378,23966,23067,21475,18159,22303,20683,24041,23728,14658,27211,23846,21300,23803,22137,24497,20045,23480 | ENSMUSG00000042800,"ENSMUSG00000042800",0,3,2,10,8,6,5,9,5,2,6,2,6,6,3,10,14,3,0,2 | ENSMUSG00000088028,"ENSMUSG00000088028",0,0,0,0,1,0,0,0,0,0,0,0,0,0,0,0,0,0,1,0 |
| ENSMUSG00000025002,"ENSMUSG00000025002",0,0,0,0,0,0,0,0,0,0,0,0,0,0,0,0,0,0,0,0 | ENSMUSG00000026809,"ENSMUSG00000026809",6,11,16,4,15,10,10,8,13,6,9,5,22,4,13,4,12,17,7,14 | ENSMUSG00000080358,"ENSMUSG00000080358",0,0,0,0,0,0,0,0,0,0,0,0,0,0,0,0,0,0,0,0 |
| ENSMUSG00000004535,"ENSMUSG00000004535",6213,5408,6304,6429,6717,6326,5350,4993,5583,7087,5998,4931,5600,6196,5383,7082,5591,7197,3998,5690 | ENSMUSG00000085532,"ENSMUSG00000085532",0,0,0,0,0,0,1,0,2,1,0,2,1,1,1,1,2,0,0,2 | ENSMUSG00000110924,"ENSMUSG00000110924",0,0,0,0,1,0,1,0,0,0,0,0,0,0,0,0,0,0,0,0 |
| ENSMUSG00000057841,"ENSMUSG00000057841",3856,7687,10662,10003,8103,7971,7840,6243,7144,8590,9357,7767,8238,8767,8081,10224,7435,10455,7202,7973 | ENSMUSG00000023935,"ENSMUSG00000023935",6,5,9,3,10,7,12,1,3,9,10,19,10,2,3,9,3,15,7,7 | ENSMUSG00000110789,"ENSMUSG00000110789",0,0,0,0,0,0,0,0,0,0,0,0,0,0,0,0,0,0,1,0 |
| ENSMUSG00000020821,"ENSMUSG00000020821",3018,5348,7606,7064,6695,5823,5905,4607,5242,6551,6690,5321,6760,6300,5885,6867,5848,8364,5353,5997 | ENSMUSG00000037683,"ENSMUSG00000037683",5,7,9,5,11,9,4,1,8,5,7,2,13,2,11,5,6,17,6,4 | ENSMUSG00000110826,"ENSMUSG00000110826",0,0,0,0,0,0,0,0,0,0,0,0,0,0,0,0,0,0,0,4 |
| ENSMUSG00000025401,"ENSMUSG00000025401",1,2,2,2,1,7,1,6,2,3,8,1,9,3,8,1,0,2,2,5 | ENSMUSG00000087518,"ENSMUSG00000087518",2,0,0,1,0,2,0,1,0,1,1,0,3,1,0,0,0,0,0,0 | ENSMUSG00000062874,"ENSMUSG00000062874",0,0,0,0,0,0,0,0,0,0,0,0,0,0,0,0,0,0,0,0 |
| ENSMUSG00000029622,"ENSMUSG00000029622",5099,7957,11998,10327,10503,9899,8568,8589,8255,9662,10396,7185,11300,10536,9505,10912,9346,11955,9299,9950 | ENSMUSG00000078902,"ENSMUSG00000078902",3,13,13,13,16,8,8,5,6,9,16,17,10,5,6,9,14,18,8,8 | ENSMUSG00000084652,"ENSMUSG00000084652",0,0,0,0,0,0,0,0,0,0,0,0,0,0,0,1,0,0,0,0 |
| ENSMUSG00000001847,"ENSMUSG00000001847",7373,8445,9820,9986,10629,8843,9376,7235,8716,9303,8821,7828,8151,8912,8399,10170,8444,10817,6983,8763 | ENSMUSG00000105512,"ENSMUSG00000105512",2,5,5,3,10,4,5,6,7,5,7,7,17,5,6,7,7,10,6,7 | ENSMUSG00000101357,"ENSMUSG00000101357",0,0,0,0,0,0,0,0,0,0,0,0,0,0,0,0,0,0,0,0 |
| ENSMUSG00000067149,"ENSMUSG00000067149",5,6,10,3,8,5,3,13,1,3,5,30,35,11,1,10,44,9,13,9 | ENSMUSG00000034968,"ENSMUSG00000034968",5,1,4,1,7,2,1,8,7,4,5,1,3,2,2,6,6,9,9,9 | ENSMUSG00000101419,"ENSMUSG00000101419",0,0,0,0,0,0,0,0,0,0,0,0,0,0,0,0,0,0,0,0 |
| ENSMUSG00000032366,"ENSMUSG00000032366",1803,2685,3081,3161,3158,2209,2947,2461,2852,3651,2831,2375,3786,2985,2851,3079,2992,3643,3034,3286 | ENSMUSG00000064158,"ENSMUSG00000064158",2,6,7,7,5,8,5,3,5,5,3,7,6,22,5,13,2,14,4,8 | ENSMUSG00000084714,"ENSMUSG00000084714",0,0,0,0,0,0,0,0,0,0,0,0,0,0,0,0,0,0,0,0 |
| ENSMUSG00000040952,"ENSMUSG00000040952",5489,6233,8332,8185,6217,6235,6300,5430,5791,7000,7955,6512,6962,7274,6583,7653,5775,8687,6693,6790 | ENSMUSG00000097503,"ENSMUSG00000097503",4,12,9,5,4,6,10,6,9,6,6,10,14,10,7,4,6,10,9,7 | ENSMUSG00000053820,"ENSMUSG00000053820",0,0,0,0,0,0,1,0,0,0,0,0,0,3,0,0,0,0,0,0 |
| ENSMUSG00000074195,"ENSMUSG00000074195",0,0,0,0,0,0,0,0,0,0,0,0,0,0,0,0,0,0,0,2 | ENSMUSG00000104671,"ENSMUSG00000104671",20,14,11,22,14,9,8,7,12,9,18,7,8,8,12,12,8,17,12,12 | ENSMUSG00000111744,"ENSMUSG00000111744",0,0,0,0,0,0,0,0,0,0,0,0,0,0,0,0,0,0,0,0 |
| ENSMUSG00000030748,"ENSMUSG00000030748",16221,21741,29839,28290,27772,29851,25365,21557,22351,29215,24455,17541,22100,30530,24287,27363,24661,26934,21110,23621 | ENSMUSG00000105565,"ENSMUSG00000105565",5,16,20,22,14,27,18,11,13,19,14,15,16,18,10,8,8,23,13,18 | ENSMUSG00000092184,"ENSMUSG00000092184",0,0,0,0,0,0,0,0,0,0,2,0,0,0,0,0,0,0,0,0 |
| ENSMUSG00000034160,"ENSMUSG00000034160",4567,3411,4494,3797,4408,4234,3598,3940,3686,3957,4836,3130,5030,3829,4028,4360,3931,4859,3111,3884 | ENSMUSG00000031576,"ENSMUSG00000031576",2,2,5,0,2,0,1,2,0,2,3,2,3,1,1,1,0,2,0,6 | ENSMUSG00000111270,"ENSMUSG00000111270",0,4,0,0,0,0,0,0,0,0,0,0,0,0,0,0,0,0,0,0 |
| ENSMUSG00000027184,"ENSMUSG00000027184",3923,4276,5300,5394,4835,4887,4867,4066,4438,4661,5377,3910,4662,4515,4219,4771,4361,5055,3653,4307 | ENSMUSG00000089820,"ENSMUSG00000089820",7,16,17,12,13,19,13,10,16,14,15,16,15,26,12,14,22,8,10,13 | ENSMUSG00000111341,"ENSMUSG00000111341",0,0,1,0,0,0,0,0,0,0,0,0,0,0,1,0,0,0,0,0 |
| ENSMUSG00000031004,"ENSMUSG00000031004",1450,1681,2770,1724,1959,2435,1624,1751,1639,1844,2050,1456,2358,1820,1600,2288,1689,2740,1563,2187 | ENSMUSG00000111658,"ENSMUSG00000111658",0,0,0,0,0,0,0,0,0,1,0,1,1,1,2,0,5,3,2,0 | ENSMUSG00000110984,"ENSMUSG00000110984",0,0,0,0,0,0,0,0,0,0,0,0,0,0,0,0,0,0,0,0 |
| ENSMUSG00000021215,"ENSMUSG00000021215",4815,4799,4815,6233,5836,4086,5871,3777,5266,5301,5018,5221,3404,5077,5126,5460,4479,5224,3539,4718 | ENSMUSG00000087377,"ENSMUSG00000087377",2,3,3,4,6,6,12,1,6,4,7,1,6,1,1,2,3,3,0,4 | ENSMUSG00000085559,"ENSMUSG00000085559",0,0,0,0,0,0,0,0,0,0,0,0,0,0,0,0,0,0,0,0 |
| ENSMUSG00000038014,"ENSMUSG00000038014",4686,4230,5600,5328,5341,5105,4752,4297,4418,4681,5534,4178,5099,4547,4449,4922,4598,5804,3964,4803 | ENSMUSG00000086866,"ENSMUSG00000086866",16,8,24,18,13,15,13,18,14,28,16,17,34,27,15,22,25,23,20,23 | ENSMUSG00000088688,"ENSMUSG00000088688",0,0,0,0,0,0,0,0,0,0,0,0,0,0,0,0,0,0,0,0 |
| ENSMUSG00000021709,"ENSMUSG00000021709",4957,4267,4502,4768,4928,4398,4165,3367,4246,5107,4769,3820,4192,4450,3767,4609,4249,4711,2466,3724 | ENSMUSG00000067399,"ENSMUSG00000067399",6,3,7,4,4,5,4,4,8,12,6,6,4,3,7,2,7,9,5,5 | ENSMUSG00000062511,"ENSMUSG00000062511",0,0,0,0,0,0,0,1,0,0,0,0,0,0,0,0,0,0,0,0 |
| ENSMUSG00000030122,"ENSMUSG00000030122",1067,3377,4926,3883,4372,4338,3646,3668,3455,3679,3709,2757,5022,4269,3571,4214,3780,5235,3904,4540 | ENSMUSG00000105981,"ENSMUSG00000105981",6,12,10,13,11,8,17,12,6,14,9,6,12,4,9,13,7,11,5,9 | ENSMUSG00000084442,"ENSMUSG00000084442",0,0,0,0,0,0,0,0,0,0,0,0,0,0,0,0,0,0,0,0 |
| ENSMUSG00000006304,"ENSMUSG00000006304",6205,8380,10597,10322,10169,9812,9256,8348,8765,10300,9672,7936,9418,10003,8603,10543,9116,11565,7317,9435 | ENSMUSG00000074206,"ENSMUSG00000074206",25,37,36,73,26,16,29,12,32,27,54,73,34,36,54,37,22,22,20,22 | ENSMUSG00000082157,"ENSMUSG00000082157",0,0,0,0,0,0,0,0,0,0,0,0,0,0,0,0,0,0,0,0 |

| ENSMUSG00000032038,"ENSMUSG00000032038",1367,1798,2151,2115,2046,1980,2011,1535,1695,2134,1844,1481,1532,2199,1683,2029,1724,2034,1415,1707 | ENSMUSG00000109973,"ENSMUSG00000109973",19,28,38,47,36,22,44,30,23,47,50,42,49,39,41,52,44,42,20,46 | ENSMUSG00000099015,"ENSMUSG00000099015",0,0,0,0,0,0,0,0,0,0,0,0,0,0,0,0,0,0,0,0 |
| --- | --- | --- |
| ENSMUSG00000026413,"ENSMUSG00000026413",21040,24667,30210,38612,33118,23526,29679,26399,29957,25875,34119,33321,28357,28281,29479,34192,25249,36227,27904,29804 | ENSMUSG00000025383,"ENSMUSG00000025383",26,17,33,12,37,38,29,26,48,42,23,16,45,42,25,57,49,54,49,35 | ENSMUSG00000084905,"ENSMUSG00000084905",0,0,0,0,1,0,0,0,0,0,1,0,0,0,0,0,0,0,0,0 |
| ENSMUSG00000021998,"ENSMUSG00000021998",11591,13008,17398,13311,16404,17617,13902,13809,12433,17620,12850,9359,16065,17039,13190,16530,15066,16890,10207,14686 | ENSMUSG00000102533,"ENSMUSG00000102533",4,0,2,1,3,5,3,4,1,0,2,0,2,2,1,5,4,2,6,3 | ENSMUSG00000085510,"ENSMUSG00000085510",0,0,0,0,0,0,0,0,0,0,0,0,0,0,0,0,0,0,0,0 |
| ENSMUSG00000020917,"ENSMUSG00000020917",2830,3644,4466,4874,4511,4010,4417,3607,4263,3965,4938,3907,4639,4449,3840,4417,3874,5091,3949,4447 | ENSMUSG00000097523,"ENSMUSG00000097523",3,9,11,11,5,6,8,9,9,6,12,3,3,13,8,7,7,5,9,15 | ENSMUSG00000088135,"ENSMUSG00000088135",0,0,0,0,0,0,0,0,0,0,0,0,0,0,0,0,0,0,0,0 |
| ENSMUSG00000021756,"ENSMUSG00000021756",13678,13132,16328,18942,15656,16326,14952,12623,13524,16941,17226,12203,14361,16807,14461,14660,15128,15446,10997,12923 | ENSMUSG00000076472,"ENSMUSG00000076472",0,0,0,1,0,1,3,0,0,1,0,2,0,4,4,0,0,4,0,2 | ENSMUSG00000088125,"ENSMUSG00000088125",0,0,0,0,0,0,0,0,0,0,0,0,0,0,0,0,0,0,0,0 |
| ENSMUSG00000041697,"ENSMUSG00000041697",2025,2818,4220,3428,3222,3072,3306,2754,2791,3174,3536,2765,3559,3413,3340,3633,2912,4224,3390,3544 | ENSMUSG00000110010,"ENSMUSG00000110010",3,4,3,9,5,4,3,4,8,5,4,0,0,15,4,4,1,9,3,4 | ENSMUSG00000082309,"ENSMUSG00000082309",0,0,0,1,0,0,0,0,1,0,0,0,0,0,0,0,0,0,0,0 |
| ENSMUSG00000027808,"ENSMUSG00000027808",6464,5061,6455,5943,6670,6296,5692,4590,5258,5553,6271,4650,5229,5551,5137,5925,5047,6418,4000,5248 | ENSMUSG00000078630,"ENSMUSG00000078630",5,8,13,8,10,6,7,11,5,14,18,7,11,8,11,15,12,7,4,9 | ENSMUSG00000087388,"ENSMUSG00000087388",0,0,0,0,0,0,0,0,0,0,0,0,0,0,0,0,0,0,0,0 |
| ENSMUSG00000020733,"ENSMUSG00000020733",1483,2598,3263,2861,3038,2822,3162,2798,2769,3087,2762,2257,3483,3439,2944,3561,2670,3796,2590,3474 | ENSMUSG00000108084,"ENSMUSG00000108084",0,0,1,5,1,1,1,0,0,2,2,2,2,2,5,1,1,1,0,3 | ENSMUSG00000087306,"ENSMUSG00000087306",1,0,0,0,0,0,0,0,0,0,0,0,0,0,0,0,0,0,0,0 |
| ENSMUSG00000026626,"ENSMUSG00000026626",1769,2162,2822,2518,2533,2310,2282,2065,2182,2474,2545,2003,2586,2317,2250,2708,2322,2945,1849,2436 | ENSMUSG00000043648,"ENSMUSG00000043648",1,0,0,2,3,2,2,1,1,6,0,1,3,0,0,0,2,3,1,1 | ENSMUSG00000082614,"ENSMUSG00000082614",0,0,0,0,0,0,0,0,0,0,0,0,0,0,0,0,0,0,0,0 |
| ENSMUSG00000058908,"ENSMUSG00000058908",1,3,2,1,5,1,5,1,6,4,5,2,1,4,2,1,0,3,1,2 | ENSMUSG00000117028,"ENSMUSG00000117028",11,9,9,3,19,18,12,19,15,18,7,16,18,13,11,14,16,23,13,17 | ENSMUSG00000065417,"ENSMUSG00000065417",0,0,0,0,0,0,0,0,0,0,0,0,1,0,0,0,0,0,0,0 |
| ENSMUSG00000018217,"ENSMUSG00000018217",1335,1563,2026,2181,1818,1974,1596,1405,1575,1965,1768,1320,1921,1730,1568,1987,1653,2132,1360,1759 | ENSMUSG00000064032,"ENSMUSG00000064032",1,8,12,18,13,3,15,7,9,9,12,5,5,2,4,14,13,5,6,6 | ENSMUSG00000084302,"ENSMUSG00000084302",0,0,0,0,0,0,0,0,0,1,1,0,0,0,0,0,0,0,0,0 |
| ENSMUSG00000028364,"ENSMUSG00000028364",14463,24004,37789,26054,26002,44840,30994,23585,24779,30210,29598,15045,29033,28468,26335,30560,26782,37208,18219,23907 | ENSMUSG00000106224,"ENSMUSG00000106224",3,11,10,7,11,9,5,13,10,13,8,14,12,15,5,14,8,10,1,7 | ENSMUSG00000082380,"ENSMUSG00000082380",0,0,0,0,0,0,0,0,0,0,0,0,0,0,0,0,0,0,0,0 |
| ENSMUSG00000045962,"ENSMUSG00000045962",6735,7788,9424,9233,9007,9172,7885,7277,8434,11637,9259,6782,8921,10055,7864,10430,8701,10098,5827,7626 | ENSMUSG00000022805,"ENSMUSG00000022805",1,1,2,7,12,3,5,5,5,5,7,2,7,1,6,4,5,6,5,0 | ENSMUSG00000050818,"ENSMUSG00000050818",0,0,0,0,0,0,0,0,0,0,0,0,0,0,0,0,0,0,0,0 |
| ENSMUSG00000041959,"ENSMUSG00000041959",7515,6832,8360,8966,7771,6729,7809,5250,6774,9355,7456,6607,6286,8402,7025,8164,6696,8185,5174,7049 | ENSMUSG00000092550,"ENSMUSG00000092550",10,20,17,15,22,19,23,18,24,22,20,20,21,27,17,23,14,24,13,16 | ENSMUSG00000095136,"ENSMUSG00000095136",0,0,0,0,0,0,0,0,0,0,0,0,0,0,0,0,0,0,0,0 |
| ENSMUSG00000036371,"ENSMUSG00000036371",3664,7290,9483,8949,7678,7606,7889,5859,7171,8019,9462,7532,7570,7504,7243,8462,6627,8812,5981,7626 | ENSMUSG00000105315,"ENSMUSG00000105315",0,0,0,0,0,0,0,0,0,0,0,0,0,0,0,0,0,0,0,0 | ENSMUSG00000082405,"ENSMUSG00000082405",0,0,0,0,0,0,0,0,0,0,0,0,0,0,0,1,0,0,0,0 |
| ENSMUSG00000035530,"ENSMUSG00000035530",7822,6594,7453,7805,7036,6551,7068,5609,7193,8035,7572,6036,5805,7635,5989,7587,5980,7656,5294,6544 | ENSMUSG00000013936,"ENSMUSG00000013936",10,40,19,31,56,12,34,31,27,67,23,39,72,32,69,26,58,31,52,69 | ENSMUSG00000082027,"ENSMUSG00000082027",0,0,0,0,0,0,0,0,0,0,0,0,0,0,0,0,0,0,0,0 |
| ENSMUSG00000068747,"ENSMUSG00000068747",922,1086,1269,1564,1338,1091,1165,1015,1180,1092,1490,1217,1306,1208,1169,1382,1006,1498,1240,1137 | ENSMUSG00000079906,"ENSMUSG00000079906",14,15,19,22,21,14,22,12,18,19,23,18,21,18,7,16,15,24,17,19 | ENSMUSG00000087779,"ENSMUSG00000087779",0,0,0,0,0,0,0,0,0,0,0,0,0,0,0,0,0,0,0,0 |
| ENSMUSG00000073702,"ENSMUSG00000073702",2965,6044,8158,7356,6948,6295,6972,5676,5941,7402,7737,6226,6914,7000,6446,7444,6001,8812,6794,6767 | ENSMUSG00000096718,"ENSMUSG00000096718",5,13,7,6,9,1,3,4,5,2,12,5,4,5,1,2,4,9,9,4 | ENSMUSG00000093278,"ENSMUSG00000093278",0,0,0,0,0,0,0,0,0,0,0,0,0,0,0,0,0,0,0,0 |
| ENSMUSG00000061787,"ENSMUSG00000061787",3884,6429,8356,7503,6567,6275,6401,5254,5937,7268,7457,6138,6614,7047,6353,7727,5935,8555,5916,6966 | ENSMUSG00000097128,"ENSMUSG00000097128",7,7,11,13,8,7,6,11,11,8,10,13,12,7,3,8,9,18,4,5 | ENSMUSG00000077706,"ENSMUSG00000077706",0,0,0,0,0,0,0,0,0,0,0,0,0,0,0,0,0,0,0,0 |
| ENSMUSG00000025980,"ENSMUSG00000025980",2699,3443,3699,4020,3453,3558,3868,2648,3268,3420,4311,3092,2929,3309,3153,3554,2834,3657,3047,3090 | ENSMUSG00000084981,"ENSMUSG00000084981",0,0,0,0,0,0,0,0,0,0,2,0,0,0,0,0,0,0,0,0 | ENSMUSG00000084856,"ENSMUSG00000084856",0,0,0,0,0,0,0,0,0,0,0,0,0,0,0,0,0,0,0,0 |
| ENSMUSG00000038615,"ENSMUSG00000038615",2636,4896,6038,7478,6181,4682,5635,4900,5221,5383,6127,5021,5926,5671,5612,5973,5502,6961,5539,5368 | ENSMUSG00000045327,"ENSMUSG00000045327",13,15,9,3,11,14,14,10,9,7,10,9,8,15,6,24,14,9,11,19 | ENSMUSG00000103376,"ENSMUSG00000103376",0,0,0,0,0,0,0,0,0,0,0,0,0,0,0,0,0,2,0,0 |
| ENSMUSG00000028967,"ENSMUSG00000028967",4916,5885,5969,6318,6552,5436,5770,5103,5833,5991,4460,3735,4804,6936,4992,5646,5345,5068,3412,4968 | ENSMUSG00000066721,"ENSMUSG00000066721",4,6,14,13,10,16,11,17,8,5,14,26,19,20,16,9,12,26,10,11 | ENSMUSG00000078668,"ENSMUSG00000078668",0,0,0,0,1,3,0,0,0,0,0,0,0,0,0,0,0,0,0,0 |
| ENSMUSG00000023914,"ENSMUSG00000023914",0,0,2,0,1,1,0,0,0,0,0,0,0,0,0,1,0,0,2,0 | ENSMUSG00000047642,"ENSMUSG00000047642",0,0,0,0,0,1,0,0,1,1,1,0,0,0,0,0,1,0,1,0 | ENSMUSG00000092762,"ENSMUSG00000092762",0,0,0,0,0,0,0,0,0,0,0,1,0,0,0,0,0,0,0,0 |
| ENSMUSG00000053617,"ENSMUSG00000053617",3764,4540,5379,5675,5536,4755,5334,3954,4730,5064,4472,4165,4504,4967,4235,5802,4630,6320,3830,4525 | ENSMUSG00000085776,"ENSMUSG00000085776",4,5,3,1,4,0,4,5,2,0,1,2,0,2,2,3,3,1,3,7 | ENSMUSG00000084152,"ENSMUSG00000084152",0,0,0,0,0,0,0,0,0,0,0,0,0,0,0,0,0,0,0,0 |
| ENSMUSG00000003617,"ENSMUSG00000003617",515,507,658,629,582,537,465,529,486,659,714,390,795,537,549,706,550,858,408,639 | ENSMUSG00000075605,"ENSMUSG00000075605",35,50,38,118,49,31,53,17,58,48,67,83,24,51,25,15,15,30,28,15 | ENSMUSG00000086617,"ENSMUSG00000086617",0,0,0,0,0,0,0,0,0,0,0,0,0,0,0,0,0,0,0,0 |
| ENSMUSG00000026664,"ENSMUSG00000026664",442,583,812,748,772,705,607,638,707,654,869,669,792,697,739,713,721,767,732,764 | ENSMUSG00000099343,"ENSMUSG00000099343",0,0,0,0,0,0,0,0,0,0,0,0,0,0,0,0,0,0,0,0 | ENSMUSG00000084120,"ENSMUSG00000084120",0,0,0,0,0,0,0,0,0,0,0,0,0,0,0,0,0,0,0,0 |
| ENSMUSG00000008683,"ENSMUSG00000008683",9356,5862,7126,6629,6381,5999,6018,4740,5685,6093,6374,5321,5682,5568,5424,6624,5195,6925,5433,6232 | ENSMUSG00000094344,"ENSMUSG00000094344",3,10,6,12,9,11,8,6,9,11,17,13,6,10,6,9,9,11,3,9 | ENSMUSG00000086089,"ENSMUSG00000086089",1,0,1,0,0,0,0,1,0,0,0,0,0,0,0,0,0,0,0,0 |
| ENSMUSG00000020850,"ENSMUSG00000020850",3441,4300,5210,5616,5335,4682,5395,4159,4896,4590,5051,4182,4605,5088,4122,4879,4418,5442,4330,4908 | ENSMUSG00000108049,"ENSMUSG00000108049",8,9,11,1,3,8,7,3,14,3,5,3,11,8,1,11,9,10,0,12 | ENSMUSG00000085864,"ENSMUSG00000085864",0,0,0,0,0,0,0,0,0,0,0,0,0,0,0,0,0,0,0,0 |
| ENSMUSG00000051391,"ENSMUSG00000051391",3321,3600,4608,4647,4012,4298,4308,3663,3979,4582,4651,3336,4547,4236,3911,4421,3738,4833,3636,4228 | ENSMUSG00000092335,"ENSMUSG00000092335",4,2,3,1,4,2,0,2,4,1,1,1,4,5,0,1,3,1,3,0 | ENSMUSG00000087139,"ENSMUSG00000087139",0,0,0,0,0,0,0,0,0,0,0,0,0,0,0,0,0,0,0,0 |
| ENSMUSG00000020571,"ENSMUSG00000020571",3838,5064,8177,8076,7277,6557,6923,7096,6180,6294,8344,5630,8007,7571,7785,6973,6430,7894,6270,7573 | ENSMUSG00000097518,"ENSMUSG00000097518",1,2,4,6,12,6,5,4,4,5,4,7,4,5,5,9,4,8,6,6 | ENSMUSG00000081548,"ENSMUSG00000081548",0,0,0,0,0,0,0,0,0,0,0,0,0,0,0,0,0,0,0,0 |
| ENSMUSG00000003032,"ENSMUSG00000003032",4188,10034,11226,13015,12660,9229,10036,9750,12187,10414,10739,10470,10151,10931,10093,13001,9537,14551,8710,11662 | ENSMUSG00000043753,"ENSMUSG00000043753",0,1,5,0,4,2,2,1,0,3,2,1,2,1,4,2,3,0,1,0 | ENSMUSG00000113740,"ENSMUSG00000113740",0,0,0,0,0,0,0,0,0,0,0,0,0,0,0,0,0,0,0,0 |
| ENSMUSG00000030432,"ENSMUSG00000030432",1045,6512,8541,8041,6873,6472,6918,5821,6539,6959,8095,6220,7146,7263,6559,8501,6263,8549,6728,7527 | ENSMUSG00000040367,"ENSMUSG00000040367",0,3,2,5,1,6,2,3,3,0,8,4,1,0,3,0,0,3,2,9 | ENSMUSG00000113763,"ENSMUSG00000113763",0,0,0,0,0,0,0,0,0,0,0,0,0,0,0,0,0,0,0,0 |
| ENSMUSG00000000049,"ENSMUSG00000000049",1,0,1,2,0,0,3,2,1,0,1,0,0,1,4,0,3,0,1,2 | ENSMUSG00000085363,"ENSMUSG00000085363",6,3,8,0,4,9,1,4,6,7,2,3,4,5,2,4,5,2,2,3 | ENSMUSG00000113810,"ENSMUSG00000113810",0,0,0,0,0,0,0,0,0,0,0,0,0,0,0,0,0,0,0,0 |
| ENSMUSG00000020581,"ENSMUSG00000020581",0,0,1,0,0,0,0,2,1,0,0,0,0,0,0,0,0,0,0,0 | ENSMUSG00000054753,"ENSMUSG00000054753",44,37,34,36,38,62,45,46,38,46,29,20,24,46,28,64,64,41,35,58 | ENSMUSG00000113371,"ENSMUSG00000113371",0,0,0,0,0,0,0,0,0,0,0,0,0,0,0,0,0,0,0,0 |
| ENSMUSG00000031503,"ENSMUSG00000031503",2227,3280,5817,4088,3800,6257,4442,3941,3563,5004,4779,2181,5627,4711,4103,4953,4120,5656,3084,4293 | ENSMUSG00000107763,"ENSMUSG00000107763",4,6,2,10,2,8,4,6,4,3,2,2,8,3,6,1,1,6,1,2 | ENSMUSG00000114152,"ENSMUSG00000114152",0,0,0,0,0,0,0,2,0,0,0,0,0,0,0,0,0,0,0,0 |
| ENSMUSG00000001472,"ENSMUSG00000001472",3556,4758,6514,6206,5936,5217,5436,5227,5169,5295,6135,4724,6230,5763,5596,6495,5154,7002,5166,5786 | ENSMUSG00000079794,"ENSMUSG00000079794",0,0,0,0,0,1,2,0,0,3,2,0,2,1,2,0,0,1,0,2 | ENSMUSG00000087898,"ENSMUSG00000087898",0,0,0,0,0,1,0,0,0,0,0,0,0,1,0,0,0,0,1,0 |
| ENSMUSG00000021910,"ENSMUSG00000021910",3165,3918,5529,5016,5225,5205,4407,4741,4699,4454,5612,3891,6158,4702,4620,5100,4482,5913,4292,4949 | ENSMUSG00000097850,"ENSMUSG00000097850",9,48,61,68,44,35,19,26,36,35,75,42,47,33,35,46,27,59,39,19 | ENSMUSG00000064653,"ENSMUSG00000064653",0,0,0,0,0,0,0,0,0,0,0,0,0,0,0,0,0,0,0,0 |
| ENSMUSG00000040564,"ENSMUSG00000040564",182,652,679,914,593,421,445,465,630,611,769,630,657,720,580,824,509,760,753,612 | ENSMUSG00000039419,"ENSMUSG00000039419",1,3,0,1,4,0,1,3,2,2,2,1,2,1,0,4,2,2,2,6 | ENSMUSG00000113191,"ENSMUSG00000113191",0,0,0,0,0,0,0,0,0,0,0,0,0,2,0,0,0,0,0,0 |
| ENSMUSG00000027620,"ENSMUSG00000027620",6084,5397,5628,5924,5799,5429,5176,4329,5223,6325,5685,4910,5143,5432,4928,5702,5128,5991,3790,5183 | ENSMUSG00000110157,"ENSMUSG00000110157",0,0,0,0,0,0,1,0,0,0,0,0,0,0,0,0,0,0,2,0 | ENSMUSG00000113251,"ENSMUSG00000113251",0,0,0,0,0,0,0,0,0,0,0,0,0,0,0,0,0,0,0,0 |
| ENSMUSG00000019370,"ENSMUSG00000019370",1050,1682,2785,2022,2113,2205,1818,2056,1664,1977,2372,1421,2841,2075,1989,2158,1916,2602,2085,2241 | ENSMUSG00000110469,"ENSMUSG00000110469",0,0,50,19,30,0,17,32,0,22,21,0,0,25,33,14,22,1,2,28 | ENSMUSG00000060404,"ENSMUSG00000060404",0,0,0,0,0,0,0,0,0,0,0,0,0,0,0,0,0,0,0,0 |
| ENSMUSG00000025544,"ENSMUSG00000025544",3109,2558,3189,3199,3300,2987,2813,2395,2744,3076,3297,2438,2939,2729,2782,3019,2791,3183,2190,2716 | ENSMUSG00000097021,"ENSMUSG00000097021",9,5,4,11,7,7,11,10,6,7,3,6,15,8,4,11,5,13,5,5 | ENSMUSG00000081576,"ENSMUSG00000081576",0,0,0,0,0,0,0,0,0,0,0,0,0,0,0,0,0,0,0,0 |
| ENSMUSG00000021676,"ENSMUSG00000021676",626,650,933,1076,777,730,612,732,702,906,900,657,940,928,833,918,796,943,506,749 | ENSMUSG00000098269,"ENSMUSG00000098269",18,5,4,15,10,6,7,3,2,8,12,11,5,7,6,8,10,8,12,2 | ENSMUSG00000099611,"ENSMUSG00000099611",0,0,0,0,0,0,0,0,0,0,0,0,0,0,0,0,0,0,0,0 |
| ENSMUSG00000035686,"ENSMUSG00000035686",2,8,11,7,13,2,5,10,14,18,12,17,33,8,13,7,8,5,20,23 | ENSMUSG00000040495,"ENSMUSG00000040495",12,3,2,4,5,1,3,5,4,5,2,5,2,4,6,9,4,4,4,0 | ENSMUSG00000083167,"ENSMUSG00000083167",0,0,0,0,0,0,1,0,0,0,0,0,0,0,0,0,0,0,0,0 |
| ENSMUSG00000036478,"ENSMUSG00000036478",7004,9634,9269,8587,10834,10405,9548,8284,10557,11781,7978,7151,9670,10526,7489,12187,9172,11394,6318,10033 | ENSMUSG00000053574,"ENSMUSG00000053574",2,6,2,5,2,8,7,2,0,1,9,5,0,3,3,6,1,9,2,5 | ENSMUSG00000069295,"ENSMUSG00000069295",0,0,0,0,0,0,0,0,0,0,0,0,0,0,0,0,0,0,0,0 |
| ENSMUSG00000012405,"ENSMUSG00000012405",3979,6001,7885,7111,6356,6294,6234,5133,5709,6524,7215,5686,6599,6622,6133,6985,5765,7966,5962,6334 | ENSMUSG00000097838,"ENSMUSG00000097838",2,3,3,2,2,4,3,1,5,8,4,0,7,3,7,4,5,6,4,3 | ENSMUSG00000083297,"ENSMUSG00000083297",0,0,0,2,1,0,0,0,0,0,0,0,1,0,0,0,0,0,0,0 |
| ENSMUSG00000024393,"ENSMUSG00000024393",2341,4976,6513,5848,5629,6129,5443,4515,4918,5540,6336,4438,5957,5696,5236,6010,5303,6793,4225,4871 | ENSMUSG00000092277,"ENSMUSG00000092277",1,3,0,5,7,3,4,1,5,3,1,2,5,6,2,6,1,4,4,3 | ENSMUSG00000097202,"ENSMUSG00000097202",0,0,0,0,1,0,1,0,2,0,0,0,0,0,0,0,0,0,0,0 |
| ENSMUSG00000028249,"ENSMUSG00000028249",10148,8400,10205,8790,10135,10502,8994,8365,8338,11161,8637,6168,9793,9884,8101,10208,9100,10929,5744,9543 | ENSMUSG00000102278,"ENSMUSG00000102278",5,4,7,7,3,4,3,3,5,4,7,4,7,6,1,13,6,2,5,9 | ENSMUSG00000077601,"ENSMUSG00000077601",1,0,0,0,0,0,0,1,0,1,0,0,0,0,0,0,0,0,0,1 |
| ENSMUSG00000014077,"ENSMUSG00000014077",2005,1984,2673,2187,2370,2288,2110,1996,2021,2345,2309,1688,2674,2212,2139,2422,2139,2842,1997,2328 | ENSMUSG00000027965,"ENSMUSG00000027965",7,0,0,2,0,2,4,0,4,2,0,0,5,0,0,1,8,0,1,3 | ENSMUSG00000114270,"ENSMUSG00000114270",0,1,0,0,0,0,0,0,0,0,0,0,0,0,0,0,0,0,0,0 |
| ENSMUSG00000070493,"ENSMUSG00000070493",3197,3259,4553,3980,3789,3466,3516,2894,3131,3880,3642,3042,3687,3581,3546,4116,3288,4245,3749,3843 | ENSMUSG00000107374,"ENSMUSG00000107374",2,2,8,9,9,6,9,2,4,1,5,6,10,8,3,6,0,2,7,2 | ENSMUSG00000114336,"ENSMUSG00000114336",0,0,0,0,0,0,0,0,0,0,0,0,0,0,0,0,2,0,0,0 |
| ENSMUSG00000023057,"ENSMUSG00000023057",0,0,1,0,0,0,0,0,3,0,3,0,0,1,0,0,0,0,0,0 | ENSMUSG00000053046,"ENSMUSG00000053046",2,0,1,1,6,2,0,0,5,8,5,2,4,5,2,8,1,3,3,0 | ENSMUSG00000114319,"ENSMUSG00000114319",0,0,0,0,0,0,0,0,0,2,1,1,0,0,0,0,0,0,0,0 |
| ENSMUSG00000071644,"ENSMUSG00000071644",4648,6277,7966,7688,6917,6949,7308,5455,6122,6686,7915,6366,6584,6607,6535,7341,5982,7803,5662,6768 | ENSMUSG00000097868,"ENSMUSG00000097868",8,6,29,11,14,5,10,10,7,15,17,10,11,20,11,35,12,9,11,10 | ENSMUSG00000114841,"ENSMUSG00000114841",0,0,0,0,0,0,0,0,0,0,0,0,0,0,0,0,0,0,0,0 |
| ENSMUSG00000051223,"ENSMUSG00000051223",11567,8217,9376,10097,9396,8214,8701,6151,8154,9663,9651,7914,7324,8246,7483,8331,7776,8754,5747,7285 | ENSMUSG00000109807,"ENSMUSG00000109807",8,8,4,3,10,8,3,0,5,3,3,1,3,3,4,1,8,5,5,4 | ENSMUSG00000103479,"ENSMUSG00000103479",0,0,0,0,0,0,0,0,0,0,0,0,0,0,0,0,0,0,0,0 |
| ENSMUSG00000006373,"ENSMUSG00000006373",821,948,1178,1153,1124,1078,1098,871,1088,1081,1210,919,1171,952,858,1050,971,1264,968,1144 | ENSMUSG00000078937,"ENSMUSG00000078937",1,5,10,6,12,9,12,6,2,7,7,8,8,6,8,11,12,5,12,12 | ENSMUSG00000098124,"ENSMUSG00000098124",0,0,0,0,1,0,0,0,0,0,0,0,0,0,0,0,3,0,0,0 |
| ENSMUSG00000036427,"ENSMUSG00000036427",2882,4066,6238,5767,5812,4881,4994,5609,5042,4592,5920,4586,6413,5260,5388,6416,4791,7208,6044,6388 | ENSMUSG00000087443,"ENSMUSG00000087443",7,14,21,14,20,23,27,13,17,27,28,7,19,29,11,19,15,21,27,19 | ENSMUSG00000088189,"ENSMUSG00000088189",0,0,0,0,0,0,0,0,0,0,0,0,0,0,0,0,0,0,0,0 |
| ENSMUSG00000024991,"ENSMUSG00000024991",4467,6495,7462,8208,6824,6419,7310,5192,5934,6643,7795,6104,6259,6653,6154,6618,6186,7569,5450,5883 | ENSMUSG00000042763,"ENSMUSG00000042763",0,3,9,9,6,2,4,4,7,13,12,9,3,3,4,2,5,8,4,4 | ENSMUSG00000114881,"ENSMUSG00000114881",0,0,0,0,0,0,0,0,0,0,0,1,1,0,0,0,2,0,0,0 |
| ENSMUSG00000032097,"ENSMUSG00000032097",5103,5424,6596,5846,5999,6484,5511,5004,5584,6733,6363,4741,6368,6299,5414,6540,5565,7086,4005,5617 | ENSMUSG00000076485,"ENSMUSG00000076485",1,9,8,8,11,14,9,9,2,6,16,3,6,12,11,7,6,13,7,7 | ENSMUSG00000113963,"ENSMUSG00000113963",0,0,0,0,0,0,0,0,0,0,0,0,0,0,0,0,0,0,0,0 |
| ENSMUSG00000021957,"ENSMUSG00000021957",1305,1849,2410,2247,2041,2250,2199,1722,2025,1886,2382,1829,2147,1915,1783,1991,1725,2221,2264,2183 | ENSMUSG00000054031,"ENSMUSG00000054031",2,2,2,2,5,3,4,1,0,1,8,1,6,3,3,0,3,4,7,2 | ENSMUSG00000112976,"ENSMUSG00000112976",0,0,0,0,0,0,0,0,0,0,0,0,0,0,0,0,0,0,0,0 |
| ENSMUSG00000027828,"ENSMUSG00000027828",7446,5695,7267,7429,7701,6974,6945,5202,6094,6769,7187,5671,5888,6442,6038,6522,5992,7386,4610,6044 | ENSMUSG00000086049,"ENSMUSG00000086049",13,15,9,9,17,23,13,7,10,8,13,8,30,17,13,9,8,21,6,14 | ENSMUSG00000113090,"ENSMUSG00000113090",0,0,0,0,0,0,0,0,0,0,0,0,0,0,0,0,0,0,0,0 |
| ENSMUSG00000022875,"ENSMUSG00000022875",8,11,13,16,15,9,19,7,9,26,6,7,4,15,4,8,8,7,14,16 | ENSMUSG00000114422,"ENSMUSG00000114422",0,0,0,1,2,4,0,2,4,2,2,2,3,4,1,0,1,3,0,1 | ENSMUSG00000114854,"ENSMUSG00000114854",0,0,0,0,0,0,0,0,0,0,0,0,0,0,0,0,0,0,0,0 |
| ENSMUSG00000021242,"ENSMUSG00000021242",5117,7138,10722,10648,8853,7828,7460,9631,8322,9496,10381,6809,11729,9652,9421,12318,9724,13412,9148,10493 | ENSMUSG00000058183,"ENSMUSG00000058183",1,3,12,2,4,2,10,1,6,5,8,4,7,5,4,5,5,8,16,7 | ENSMUSG00000042243,"ENSMUSG00000042243",0,0,0,0,0,0,0,0,0,0,0,0,0,0,0,0,0,0,0,0 |
| ENSMUSG00000027249,"ENSMUSG00000027249",2,0,0,1,0,1,0,3,3,1,0,2,0,0,1,0,1,0,8,2 | ENSMUSG00000111837,"ENSMUSG00000111837",10,24,35,33,39,9,18,17,25,20,41,30,25,28,30,28,13,31,42,24 | ENSMUSG00000033834,"ENSMUSG00000033834",3,0,0,0,0,0,0,0,0,0,0,0,0,0,0,0,0,0,0,0 |
| ENSMUSG00000007850,"ENSMUSG00000007850",4684,4650,5716,5371,5532,5625,5230,4072,4819,5070,5931,4357,4932,4970,4907,5432,4559,6469,4361,4768 | ENSMUSG00000092268,"ENSMUSG00000092268",4,4,5,5,10,2,5,3,8,2,11,6,7,10,9,7,10,9,10,8 | ENSMUSG00000113014,"ENSMUSG00000113014",0,0,0,0,0,0,0,0,0,0,0,0,0,0,0,0,0,0,0,0 |
| ENSMUSG00000005397,"ENSMUSG00000005397",2826,3137,4079,3962,3856,4850,4231,3321,3384,3802,3682,2390,3450,3621,3055,3523,3219,4311,2700,3370 | ENSMUSG00000081996,"ENSMUSG00000081996",6,4,7,5,5,4,15,9,6,4,10,12,3,6,8,6,2,5,1,4 | ENSMUSG00000094096,"ENSMUSG00000094096",0,0,0,0,0,0,0,0,0,0,0,0,0,0,0,0,0,0,0,0 |
| ENSMUSG00000028980,"ENSMUSG00000028980",670,929,1289,1075,1186,1395,982,1116,1014,1138,1154,670,1380,1275,1210,1279,1084,1530,952,1162 | ENSMUSG00000103921,"ENSMUSG00000103921",1,1,0,0,1,1,0,0,0,3,0,1,0,0,0,0,0,0,0,1 | ENSMUSG00000113708,"ENSMUSG00000113708",0,0,0,0,0,0,0,0,0,0,0,0,0,0,1,0,0,0,0,0 |
| ENSMUSG00000038366,"ENSMUSG00000038366",2344,3462,4456,3402,4298,4623,3855,4126,3760,4515,4136,2795,5782,4517,3936,4728,4448,5372,3310,4855 | ENSMUSG00000087406,"ENSMUSG00000087406",0,2,7,2,2,3,2,1,2,3,0,2,8,0,4,2,0,0,1,1 | ENSMUSG00000113825,"ENSMUSG00000113825",0,0,0,0,0,0,0,0,0,0,0,0,0,0,0,0,0,0,0,0 |
| ENSMUSG00000034248,"ENSMUSG00000034248",798,860,889,825,894,907,945,858,876,964,983,633,972,842,771,1040,809,1144,770,933 | ENSMUSG00000099825,"ENSMUSG00000099825",0,1,8,9,3,2,2,1,0,1,3,2,6,0,4,4,1,4,4,4 | ENSMUSG00000114092,"ENSMUSG00000114092",0,0,0,0,0,0,0,0,0,0,0,0,0,0,0,0,0,0,0,0 |
| ENSMUSG00000068220,"ENSMUSG00000068220",2063,5579,9658,6793,7307,7486,6969,7312,6013,6623,8195,4590,10010,7024,7287,7340,7072,8074,6676,7873 | ENSMUSG00000097136,"ENSMUSG00000097136",8,9,9,12,11,6,3,8,5,4,21,2,7,6,6,6,6,7,4,5 | ENSMUSG00000097483,"ENSMUSG00000097483",0,0,0,0,0,0,0,0,0,0,0,0,0,0,0,0,0,0,0,0 |
| ENSMUSG00000023224,"ENSMUSG00000023224",2475,2946,3848,4213,3387,3639,3290,3955,3418,3583,4077,2904,5029,3588,3603,4496,3488,5754,3681,4382 | ENSMUSG00000051723,"ENSMUSG00000051723",0,0,2,1,0,0,0,2,0,1,1,0,1,3,4,2,1,6,0,0 | ENSMUSG00000114816,"ENSMUSG00000114816",0,0,0,0,0,0,0,0,0,0,0,0,0,0,0,0,0,0,0,0 |
| ENSMUSG00000058135,"ENSMUSG00000058135",275,500,526,617,597,358,522,386,487,414,579,476,460,487,412,451,435,439,445,412 | ENSMUSG00000086464,"ENSMUSG00000086464",11,6,6,3,9,9,7,7,4,4,10,6,4,14,8,6,9,9,5,4 | ENSMUSG00000103035,"ENSMUSG00000103035",0,0,0,0,0,0,0,0,0,0,0,1,0,0,0,0,0,0,0,0 |
| ENSMUSG00000029068,"ENSMUSG00000029068",4839,5065,6217,5965,6195,5789,5700,5152,5187,5916,6257,4633,6054,6122,5336,6332,5167,6899,4578,5628 | ENSMUSG00000085612,"ENSMUSG00000085612",3,9,8,8,9,16,17,12,3,8,7,7,4,9,9,2,9,7,3,14 | ENSMUSG00000114038,"ENSMUSG00000114038",0,0,0,0,0,0,0,0,1,0,0,0,0,0,0,0,0,0,0,0 |
| ENSMUSG00000025037,"ENSMUSG00000025037",909,1135,1227,1612,1121,940,1070,699,1026,1196,1395,1271,873,1098,1003,1165,963,1195,830,773 | ENSMUSG00000038496,"ENSMUSG00000038496",9,9,1,9,8,12,2,2,2,2,2,2,5,5,2,7,5,8,2,2 | ENSMUSG00000114778,"ENSMUSG00000114778",1,0,0,0,0,0,0,0,0,0,1,0,0,0,0,0,0,0,0,0 |
| ENSMUSG00000040731,"ENSMUSG00000040731",5368,6231,8382,8179,7921,6938,6853,6657,6458,7252,7695,6760,7231,7449,7261,9006,6578,9052,6442,7303 | ENSMUSG00000075279,"ENSMUSG00000075279",1,4,12,5,4,4,7,2,10,9,7,6,9,4,2,11,6,8,7,10 | ENSMUSG00000114479,"ENSMUSG00000114479",0,0,0,0,0,0,0,0,0,0,0,0,0,0,0,1,0,0,0,0 |
| ENSMUSG00000010064,"ENSMUSG00000010064",23,40,38,53,41,17,38,29,40,31,30,35,51,49,26,30,32,52,50,57 | ENSMUSG00000066116,"ENSMUSG00000066116",3,1,10,7,3,2,3,2,2,8,2,3,3,4,5,6,2,1,1,4 | ENSMUSG00000065866,"ENSMUSG00000065866",0,0,0,0,0,0,0,0,0,0,0,0,0,0,0,0,0,0,0,0 |
| ENSMUSG00000017774,"ENSMUSG00000017774",3124,4567,5708,5778,5698,5242,5078,4656,4667,5291,5194,4240,5263,5464,4984,6026,4887,6362,4398,5229 | ENSMUSG00000076497,"ENSMUSG00000076497",3,4,9,4,5,2,4,5,5,5,8,1,10,0,5,7,3,6,3,6 | ENSMUSG00000099115,"ENSMUSG00000099115",0,0,0,0,0,0,0,0,0,0,0,0,0,0,1,0,1,0,0,0 |
| ENSMUSG00000030088,"ENSMUSG00000030088",103,160,231,282,206,218,180,213,210,167,237,193,299,226,222,211,171,283,275,309 | ENSMUSG00000030178,"ENSMUSG00000030178",1,1,4,11,3,2,4,9,7,8,11,2,11,10,9,4,8,5,3,10 | ENSMUSG00000114521,"ENSMUSG00000114521",0,0,0,0,1,0,1,0,0,0,0,0,0,0,0,0,0,0,0,0 |
| ENSMUSG00000029063,"ENSMUSG00000029063",3456,4100,5324,4984,4489,4762,4792,3393,4086,4486,4741,3445,4311,4530,3862,4616,3973,5143,3749,4302 | ENSMUSG00000045587,"ENSMUSG00000045587",0,0,0,0,1,0,0,0,0,0,0,0,0,1,0,0,0,0,0,0 | ENSMUSG00000103545,"ENSMUSG00000103545",0,0,0,0,0,0,0,0,0,0,0,0,0,0,0,0,0,1,0,0 |
| ENSMUSG00000006360,"ENSMUSG00000006360",697,2298,3565,3470,2440,2380,2604,2055,2318,3579,2803,1979,2666,2906,2394,2735,2246,3215,2218,2859 | ENSMUSG00000002324,"ENSMUSG00000002324",3,2,1,5,5,3,0,2,3,3,5,0,2,2,2,9,1,2,1,2 | ENSMUSG00000114223,"ENSMUSG00000114223",0,0,0,0,0,0,0,0,0,0,0,0,0,0,0,0,0,0,0,0 |
| ENSMUSG00000067235,"ENSMUSG00000067235",73,187,301,304,202,203,215,227,205,256,331,244,302,270,267,70,247,416,235,251 | ENSMUSG00000085642,"ENSMUSG00000085642",22,19,15,16,21,20,9,17,20,20,11,15,12,23,8,14,18,19,14,19 | ENSMUSG00000077261,"ENSMUSG00000077261",0,0,0,0,0,0,0,0,0,0,0,0,0,0,0,0,0,0,0,0 |
| ENSMUSG00000028699,"ENSMUSG00000028699",5,1,1,5,5,4,6,4,0,2,4,0,1,4,2,4,1,3,0,2 | ENSMUSG00000082002,"ENSMUSG00000082002",0,1,3,3,0,3,3,0,0,1,1,0,0,4,0,2,3,0,0,3 | ENSMUSG00000067122,"ENSMUSG00000067122",0,0,0,0,0,0,0,0,0,0,0,0,0,0,0,0,0,0,0,0 |
| ENSMUSG00000026219,"ENSMUSG00000026219",9051,7873,8493,9486,9513,8038,8324,6681,7977,8773,8279,7316,7633,8391,7635,9368,7917,9573,5507,7302 | ENSMUSG00000034563,"ENSMUSG00000034563",3,2,10,9,11,10,9,6,4,13,9,8,4,6,15,11,8,10,5,15 | ENSMUSG00000086057,"ENSMUSG00000086057",0,0,0,0,0,0,0,0,0,0,0,0,0,0,0,0,0,0,0,0 |
| ENSMUSG00000053279,"ENSMUSG00000053279",71,66,108,82,72,51,59,75,74,104,90,69,112,104,70,70,71,92,90,94 | ENSMUSG00000107306,"ENSMUSG00000107306",16,19,18,14,18,17,18,12,19,20,15,8,32,16,23,25,9,15,13,24 | ENSMUSG00000051002,"ENSMUSG00000051002",0,0,0,0,0,0,0,0,0,0,0,0,0,0,0,0,0,0,0,0 |
| ENSMUSG00000079477,"ENSMUSG00000079477",7956,8342,10179,9907,9607,9559,9214,9060,8993,10602,9297,7143,10688,10483,9298,11358,9038,11788,7592,10013 | ENSMUSG00000102983,"ENSMUSG00000102983",0,2,1,0,1,0,0,0,0,0,0,0,0,0,0,0,0,1,0,0 | ENSMUSG00000088814,"ENSMUSG00000088814",0,0,0,0,0,2,0,1,0,0,0,0,0,0,0,1,0,0,0,0 |
| ENSMUSG00000058655,"ENSMUSG00000058655",3398,4415,5736,5827,4797,4553,5186,3918,4605,4735,5823,4527,5050,4796,4547,5021,4238,5409,4156,4570 | ENSMUSG00000064224,"ENSMUSG00000064224",21,38,38,69,49,42,59,25,65,20,62,54,29,46,36,41,22,33,52,38 | ENSMUSG00000114879,"ENSMUSG00000114879",0,0,0,0,0,0,0,0,0,0,0,1,0,0,0,0,0,0,0,0 |
| ENSMUSG00000027763,"ENSMUSG00000027763",4233,3775,4282,4141,4525,4202,3502,3267,3754,3939,4408,3160,3876,3593,3302,3926,3604,4387,2800,3333 | ENSMUSG00000114871,"ENSMUSG00000114871",6,20,36,35,19,31,20,32,10,14,31,24,57,14,23,38,24,45,24,27 | ENSMUSG00000114347,"ENSMUSG00000114347",0,0,0,0,0,0,0,0,0,0,0,0,0,0,0,0,0,0,0,0 |
| ENSMUSG00000066036,"ENSMUSG00000066036",4180,5020,5733,6544,6160,5596,6175,4777,5194,5563,5389,4175,5105,5878,5057,6510,5428,6865,4222,4969 | ENSMUSG00000092124,"ENSMUSG00000092124",5,5,7,5,8,1,2,1,4,3,4,2,2,5,2,5,6,3,6,4 | ENSMUSG00000114485,"ENSMUSG00000114485",0,0,0,0,0,0,0,0,0,0,0,0,0,0,0,0,0,0,0,0 |
| ENSMUSG00000046330,"ENSMUSG00000046330",4463,6216,8119,7727,6158,6226,6408,5335,6202,7154,7572,6174,6389,6867,5741,7592,5872,8099,5923,6910 | ENSMUSG00000108199,"ENSMUSG00000108199",9,16,14,10,10,10,8,7,9,16,14,8,15,13,10,7,16,13,9,10 | ENSMUSG00000101579,"ENSMUSG00000101579",0,0,0,0,0,0,0,0,0,0,0,0,0,0,0,0,0,0,0,1 |
| ENSMUSG00000024644,"ENSMUSG00000024644",4286,4488,6223,6533,5415,4945,5030,4804,4593,5657,5321,3852,5761,6165,5252,6369,5614,7237,4537,5583 | ENSMUSG00000061311,"ENSMUSG00000061311",5,7,6,8,0,7,3,3,4,9,6,2,4,5,4,7,1,6,5,2 | ENSMUSG00000088207,"ENSMUSG00000088207",0,0,0,0,0,0,0,0,1,0,0,0,0,0,0,0,0,0,0,0 |
| ENSMUSG00000024835,"ENSMUSG00000024835",2049,2661,4417,3951,3402,3467,3066,3468,2972,3266,3882,2648,4310,3612,3597,3879,3339,4502,3589,3992 | ENSMUSG00000086999,"ENSMUSG00000086999",4,1,2,0,3,1,0,0,2,1,0,0,0,3,0,1,0,2,0,2 | ENSMUSG00000111967,"ENSMUSG00000111967",0,0,0,0,0,0,0,0,0,0,0,2,0,0,0,0,0,0,0,0 |
| ENSMUSG00000062070,"ENSMUSG00000062070",5381,6461,10158,8670,9918,8537,6560,9687,7282,7457,8465,7135,10769,8325,8844,11287,7985,12896,9073,10601 | ENSMUSG00000063975,"ENSMUSG00000063975",36,28,67,39,41,38,19,36,38,39,39,39,49,29,32,54,25,57,48,68 | ENSMUSG00000113937,"ENSMUSG00000113937",0,0,0,0,0,0,0,0,0,0,0,0,0,0,0,0,0,0,0,0 |
| ENSMUSG00000023064,"ENSMUSG00000023064",31,93,115,118,131,154,91,108,74,134,121,111,188,117,137,131,110,147,136,158 | ENSMUSG00000097115,"ENSMUSG00000097115",0,0,3,1,2,1,1,0,0,0,0,0,1,1,0,2,1,0,0,1 | ENSMUSG00000064717,"ENSMUSG00000064717",0,0,0,0,0,0,0,0,0,0,0,0,0,0,0,0,0,0,0,0 |
| ENSMUSG00000007815,"ENSMUSG00000007815",5276,5180,6652,6382,6610,6063,5692,5153,5657,6402,6342,4905,5997,5813,5426,6167,5561,6708,4481,5880 | ENSMUSG00000044737,"ENSMUSG00000044737",4,10,46,27,25,42,26,64,52,14,12,14,47,26,34,76,13,72,47,56 | ENSMUSG00000113246,"ENSMUSG00000113246",0,0,0,0,0,0,0,0,0,0,0,0,0,0,0,0,0,0,0,0 |
| ENSMUSG00000038642,"ENSMUSG00000038642",6933,10886,15700,13953,13549,11452,9328,13075,11945,13675,13958,8240,16825,13551,12352,16412,13458,17471,13212,14952 | ENSMUSG00000089742,"ENSMUSG00000089742",10,11,10,20,9,12,11,4,13,21,17,6,15,13,11,11,10,23,22,15 | ENSMUSG00000113592,"ENSMUSG00000113592",0,0,0,0,0,0,0,0,0,0,0,0,0,0,0,0,0,0,0,0 |
| ENSMUSG00000031808,"ENSMUSG00000031808",477,501,801,670,626,580,587,674,629,624,819,454,794,607,541,648,590,731,548,620 | ENSMUSG00000094845,"ENSMUSG00000094845",21,37,34,23,24,35,23,26,31,23,28,18,29,37,26,54,12,31,32,24 | ENSMUSG00000116775,"ENSMUSG00000116775",0,0,0,0,0,0,0,0,0,0,0,0,0,0,0,0,0,0,0,0 |
| ENSMUSG00000026395,"ENSMUSG00000026395",5352,4724,5561,4123,5232,6319,4498,5094,4076,5933,5267,2843,6508,5506,4734,5750,4979,7435,3405,5134 | ENSMUSG00000090185,"ENSMUSG00000090185",4,5,6,1,7,3,5,3,7,1,7,4,0,2,3,4,10,0,3,7 | ENSMUSG00000114158,"ENSMUSG00000114158",0,0,0,0,0,0,0,0,0,0,0,0,0,0,0,0,0,0,0,0 |
| ENSMUSG00000041740,"ENSMUSG00000041740",2875,4054,4957,4769,4539,4114,4285,3416,4135,4650,4847,3849,4431,4475,4124,4820,4098,5222,3383,4101 | ENSMUSG00000034551,"ENSMUSG00000034551",13,13,6,13,11,7,11,12,7,16,4,7,17,11,9,9,10,9,7,8 | ENSMUSG00000113915,"ENSMUSG00000113915",0,0,0,0,0,0,0,0,0,0,0,0,0,0,0,0,0,0,0,0 |
| ENSMUSG00000031765,"ENSMUSG00000031765",13755,10945,11247,16196,13352,11252,10834,7478,12993,16810,9963,11614,6935,15319,10189,13818,10188,12773,6980,9818 | ENSMUSG00000064293,"ENSMUSG00000064293",0,0,1,1,3,0,2,1,0,0,1,0,5,1,0,1,0,1,0,1 | ENSMUSG00000113085,"ENSMUSG00000113085",0,0,0,0,0,1,0,0,0,0,0,0,0,0,0,0,0,0,0,0 |
| ENSMUSG00000053907,"ENSMUSG00000053907",7014,6224,6023,8610,7020,5708,6804,3884,5710,5936,6777,7286,3472,6193,5714,5797,4874,5792,4680,4191 | ENSMUSG00000099133,"ENSMUSG00000099133",0,0,0,0,1,0,0,0,0,0,0,0,0,0,0,0,0,0,0,0 | ENSMUSG00000113832,"ENSMUSG00000113832",0,0,0,0,0,1,0,0,0,0,0,0,0,0,0,0,0,0,0,0 |
| ENSMUSG00000019179,"ENSMUSG00000019179",2782,3503,4195,4121,4190,3531,4188,3348,3794,3782,4238,3247,3863,3987,3589,4211,3562,4286,3861,4175 | ENSMUSG00000102196,"ENSMUSG00000102196",25,20,26,17,32,51,37,30,15,57,16,28,53,45,30,27,26,33,15,28 | ENSMUSG00000114135,"ENSMUSG00000114135",0,0,1,0,0,0,0,0,0,0,0,0,0,0,0,0,0,0,0,0 |
| ENSMUSG00000008475,"ENSMUSG00000008475",8430,7218,8944,8598,8795,8256,7698,6193,7378,8661,8178,6478,7307,8448,7283,8496,7599,9217,5357,7455 | ENSMUSG00000103108,"ENSMUSG00000103108",0,11,19,9,16,10,9,4,13,15,10,14,14,21,16,14,4,17,8,7 | ENSMUSG00000113154,"ENSMUSG00000113154",0,0,0,0,0,0,0,0,0,0,0,0,0,0,0,0,0,0,0,0 |
| ENSMUSG00000020484,"ENSMUSG00000020484",5066,5116,6286,6167,6008,6540,5759,4861,5526,6699,5491,4409,5556,6759,5430,6399,4975,6305,4434,5379 | ENSMUSG00000090293,"ENSMUSG00000090293",24,27,30,29,32,38,22,18,25,60,36,18,33,39,24,39,20,36,20,18 | ENSMUSG00000113438,"ENSMUSG00000113438",0,0,0,0,0,0,0,0,0,0,0,0,0,0,0,0,0,0,0,0 |
| ENSMUSG00000037820,"ENSMUSG00000037820",7421,12107,11239,11695,11105,11440,11715,12020,12030,14633,10828,7377,12799,17029,11052,14253,13818,14786,9406,12449 | ENSMUSG00000044071,"ENSMUSG00000044071",1,1,1,0,1,2,1,2,0,5,0,0,3,6,2,3,4,1,0,1 | ENSMUSG00000112821,"ENSMUSG00000112821",0,0,0,2,0,0,0,0,1,0,0,0,0,0,0,0,0,1,0,0 |
| ENSMUSG00000000346,"ENSMUSG00000000346",5071,5565,6886,5791,6953,6228,5608,5055,5705,6697,6046,4793,5988,6035,4925,6499,5522,7026,4626,5805 | ENSMUSG00000110816,"ENSMUSG00000110816",9,8,9,10,6,8,5,5,8,6,7,4,12,5,6,11,6,6,3,6 | ENSMUSG00000112186,"ENSMUSG00000112186",0,0,0,0,0,0,0,0,0,0,0,0,0,0,0,0,0,1,0,0 |
| ENSMUSG00000074207,"ENSMUSG00000074207",74,110,143,172,136,60,42,64,93,112,177,120,168,128,97,88,77,105,98,116 | ENSMUSG00000113529,"ENSMUSG00000113529",7,9,1,7,4,2,3,2,0,1,7,1,5,4,3,0,4,5,6,0 | ENSMUSG00000113161,"ENSMUSG00000113161",0,0,0,0,0,0,0,0,0,0,0,0,0,0,0,0,0,0,0,0 |
| ENSMUSG00000028530,"ENSMUSG00000028530",5599,6132,7448,7156,7252,7030,6548,5676,6275,6919,6738,5308,6956,6948,6060,7509,6204,8033,4847,6312 | ENSMUSG00000005553,"ENSMUSG00000005553",3,5,4,8,5,11,4,4,1,10,6,5,8,10,3,5,4,10,11,5 | ENSMUSG00000113944,"ENSMUSG00000113944",0,0,0,0,0,0,0,0,1,0,0,0,1,0,0,0,0,0,0,0 |
| ENSMUSG00000061315,"ENSMUSG00000061315",4022,5689,6599,6785,5949,5577,5987,4497,5381,6347,6844,5628,5246,5830,5356,6201,4975,6580,5268,5819 | ENSMUSG00000010044,"ENSMUSG00000010044",3,7,13,11,6,9,4,8,13,11,12,3,10,0,6,11,12,4,3,11 | ENSMUSG00000114155,"ENSMUSG00000114155",0,0,0,0,0,0,0,0,0,1,0,0,0,0,0,0,0,0,0,0 |
| ENSMUSG00000034926,"ENSMUSG00000034926",2156,2415,2795,3675,2839,3520,3765,1839,2739,2585,3214,2767,2844,2865,2221,2441,2354,3799,2678,2517 | ENSMUSG00000114277,"ENSMUSG00000114277",13,21,40,22,37,19,37,23,14,18,22,13,21,23,13,22,23,31,8,25 | ENSMUSG00000066457,"ENSMUSG00000066457",0,1,0,0,0,0,0,0,0,0,0,0,0,0,0,0,0,0,0,0 |
| ENSMUSG00000018770,"ENSMUSG00000018770",1808,3497,4331,4045,3845,3662,3713,3294,3675,4020,4573,3511,3763,3774,3572,4120,3460,4690,3804,4052 | ENSMUSG00000037627,"ENSMUSG00000037627",0,0,1,2,3,0,1,3,0,4,3,1,1,0,0,0,7,9,4,1 | ENSMUSG00000079031,"ENSMUSG00000079031",0,3,0,0,0,0,0,0,0,0,0,0,0,0,0,0,0,0,0,0 |
| ENSMUSG00000038209,"ENSMUSG00000038209",14,21,28,18,28,21,24,15,21,25,21,14,25,34,14,19,21,33,13,32 | ENSMUSG00000105434,"ENSMUSG00000105434",16,14,9,5,21,15,13,14,20,14,23,25,5,12,11,18,10,22,8,25 | ENSMUSG00000093847,"ENSMUSG00000093847",0,1,0,0,0,0,0,0,1,0,0,0,0,0,0,0,0,0,0,0 |
| ENSMUSG00000044734,"ENSMUSG00000044734",5634,4944,5327,6718,6236,4776,5492,4048,4704,6089,4767,5306,3429,5342,4997,5901,4276,6264,3882,4783 | ENSMUSG00000059854,"ENSMUSG00000059854",1,0,0,1,0,0,0,0,0,0,2,0,3,1,1,0,1,0,1,2 | ENSMUSG00000113578,"ENSMUSG00000113578",0,0,0,0,0,0,0,0,0,0,1,0,0,0,0,0,0,0,0,0 |
| ENSMUSG00000041453,"ENSMUSG00000041453",2005,4776,5452,5114,4628,4298,4522,3418,4195,4850,5318,4312,4195,4397,4252,5258,4124,5435,4614,4523 | ENSMUSG00000113396,"ENSMUSG00000113396",8,8,24,14,20,7,14,19,15,14,10,11,20,17,9,16,16,19,21,9 | ENSMUSG00000113877,"ENSMUSG00000113877",0,0,0,0,0,0,0,0,0,0,0,0,0,0,0,0,0,0,1,0 |
| ENSMUSG00000019761,"ENSMUSG00000019761",25484,29373,27028,54578,28070,18545,24363,13213,22679,26367,40259,34157,30027,26401,19613,27444,20524,17231,17798,11263 | ENSMUSG00000099413,"ENSMUSG00000099413",6,10,4,11,4,5,15,3,4,5,11,2,9,14,11,12,6,13,7,5 | ENSMUSG00000090989,"ENSMUSG00000090989",0,0,0,0,0,0,0,0,0,0,0,0,0,0,0,0,0,0,0,0 |
| ENSMUSG00000004655,"ENSMUSG00000004655",2064,2405,3463,2677,3095,3336,2930,2843,2635,3139,3133,1829,3957,3204,2809,3329,3036,4140,2861,3266 | ENSMUSG00000083337,"ENSMUSG00000083337",9,4,2,4,5,8,2,4,6,5,5,6,7,10,8,7,3,15,4,9 | ENSMUSG00000086060,"ENSMUSG00000086060",0,0,0,0,0,0,0,0,0,0,0,0,0,0,0,0,0,0,0,0 |
| ENSMUSG00000033295,"ENSMUSG00000033295",3637,4833,6058,6671,6510,5245,5579,4840,5209,4543,6566,5958,5699,5127,5330,6775,4933,7437,4821,5458 | ENSMUSG00000024485,"ENSMUSG00000024485",21,34,33,21,18,20,25,22,26,15,24,17,13,17,16,25,21,37,18,18 | ENSMUSG00000076313,"ENSMUSG00000076313",0,0,0,0,1,0,0,0,0,0,0,0,1,0,0,0,0,0,0,0 |
| ENSMUSG00000027642,"ENSMUSG00000027642",2989,3461,4827,4787,4385,4380,4652,3832,4086,3755,4321,3462,4371,4253,4016,4303,3562,4750,3799,4463 | ENSMUSG00000047678,"ENSMUSG00000047678",7,7,1,11,7,9,2,4,7,4,7,7,12,5,3,1,6,4,11,5 | ENSMUSG00000080411,"ENSMUSG00000080411",0,0,1,0,0,0,0,0,0,0,0,0,0,0,0,0,0,0,0,0 |
| ENSMUSG00000017009,"ENSMUSG00000017009",5400,9228,10262,11724,10050,8995,10011,8342,9659,10553,8961,7556,7879,11575,9058,10663,9597,9282,7773,9065 | ENSMUSG00000034486,"ENSMUSG00000034486",0,0,2,0,6,9,3,2,0,0,0,1,0,0,0,1,0,1,2,4 | ENSMUSG00000077980,"ENSMUSG00000077980",0,0,0,0,0,0,0,0,0,0,0,0,0,1,0,0,0,0,0,3 |
| ENSMUSG00000022841,"ENSMUSG00000022841",7728,6435,10635,9257,9213,8733,8197,7433,7223,8281,9283,6452,9057,8617,8315,8562,7748,9299,8098,8226 | ENSMUSG00000085483,"ENSMUSG00000085483",0,0,0,0,0,0,1,0,1,0,0,0,1,0,0,0,3,0,1,1 | ENSMUSG00000077001,"ENSMUSG00000077001",0,1,0,0,0,0,0,0,0,0,0,0,0,0,0,0,0,0,0,0 |
| ENSMUSG00000035545,"ENSMUSG00000035545",2354,4317,5190,5397,4743,4373,4681,4041,4559,4450,5343,3966,4911,4998,4292,5118,4497,5649,4702,4327 | ENSMUSG00000087241,"ENSMUSG00000087241",0,0,0,0,0,0,0,0,0,0,0,0,0,0,0,0,0,0,0,0 | ENSMUSG00000091606,"ENSMUSG00000091606",0,0,0,0,0,0,0,0,0,0,0,0,0,0,0,0,0,0,0,0 |
| ENSMUSG00000068823,"ENSMUSG00000068823",4596,4309,5235,5315,5365,4881,4458,3841,4444,5155,5030,4006,4572,4613,4330,4883,4452,5497,3020,4415 | ENSMUSG00000109572,"ENSMUSG00000109572",0,0,0,1,3,0,0,3,0,0,3,2,0,2,1,0,2,4,1,1 | ENSMUSG00000102453,"ENSMUSG00000102453",0,0,0,0,0,0,0,0,0,0,0,0,0,0,0,0,0,0,0,0 |
| ENSMUSG00000035202,"ENSMUSG00000035202",3506,2773,4468,5900,3629,3129,4316,2522,3059,3316,10777,2557,3135,3838,2582,3766,2745,3796,1923,3330 | ENSMUSG00000092364,"ENSMUSG00000092364",1,3,2,3,3,0,2,1,0,0,0,1,1,0,5,0,2,1,1,1 | ENSMUSG00000106077,"ENSMUSG00000106077",0,0,0,0,0,0,0,0,2,0,0,0,0,0,0,1,0,0,0,0 |
| ENSMUSG00000024308,"ENSMUSG00000024308",6402,8785,11087,14709,11774,9652,11068,11113,11416,10192,12113,9964,11667,12002,10703,14267,10702,15802,8979,12593 | ENSMUSG00000110517,"ENSMUSG00000110517",17,11,17,24,17,15,16,14,10,11,16,18,10,11,12,16,19,14,11,8 | ENSMUSG00000105123,"ENSMUSG00000105123",0,0,0,0,0,0,0,0,0,0,0,0,0,0,0,0,0,0,0,0 |
| ENSMUSG00000019132,"ENSMUSG00000019132",5079,5101,6014,5598,6171,6307,5453,4996,5452,5893,6520,4465,6411,5210,5187,6044,5168,7377,4570,5368 | ENSMUSG00000115284,"ENSMUSG00000115284",7,7,2,8,11,14,11,13,12,11,9,12,9,16,11,8,14,6,4,10 | ENSMUSG00000115585,"ENSMUSG00000115585",0,0,0,0,0,0,0,0,0,0,0,0,0,0,0,0,0,0,0,0 |
| ENSMUSG00000020250,"ENSMUSG00000020250",10436,12056,10491,14564,13829,9444,15050,8424,11769,13744,11449,12373,8236,13751,12634,13569,11358,12745,7714,10520 | ENSMUSG00000065523,"ENSMUSG00000065523",0,0,1,0,0,0,1,0,0,0,0,0,1,0,0,0,0,0,0,0 | ENSMUSG00000095874,"ENSMUSG00000095874",0,0,0,0,0,0,0,0,0,0,0,0,0,0,0,0,0,0,0,0 |
| ENSMUSG00000054422,"ENSMUSG00000054422",0,0,0,0,0,0,2,3,0,0,0,1,0,0,0,0,0,0,7,5 | ENSMUSG00000103753,"ENSMUSG00000103753",15,11,8,13,11,12,12,10,5,12,11,5,15,12,9,15,13,5,8,17 | ENSMUSG00000114971,"ENSMUSG00000114971",0,0,0,0,0,0,0,0,0,0,0,0,0,0,0,0,0,0,0,0 |
| ENSMUSG00000022982,"ENSMUSG00000022982",360,736,1220,919,917,1017,941,818,833,930,967,716,1061,978,867,1020,900,1382,920,982 | ENSMUSG00000106478,"ENSMUSG00000106478",35,18,13,22,10,23,33,21,12,23,12,11,24,19,34,26,21,42,31,29 | ENSMUSG00000087836,"ENSMUSG00000087836",0,0,0,0,0,0,0,0,0,0,0,0,0,0,0,0,0,0,0,0 |
| ENSMUSG00000031375,"ENSMUSG00000031375",7836,7926,12878,9754,10959,15213,10888,11254,10012,10160,10619,6971,14246,9358,9775,10592,10148,13039,9763,11661 | ENSMUSG00000030321,"ENSMUSG00000030321",3,3,5,5,7,4,4,4,1,2,10,2,15,3,5,3,9,1,5,6 | ENSMUSG00000115337,"ENSMUSG00000115337",0,0,0,0,0,0,0,0,0,0,0,0,0,0,0,0,0,0,0,0 |
| ENSMUSG00000027984,"ENSMUSG00000027984",714,772,1024,932,831,707,741,734,756,718,1138,781,1042,791,671,891,739,1090,842,857 | ENSMUSG00000089753,"ENSMUSG00000089753",28,17,31,12,36,15,13,19,21,23,35,5,44,19,21,28,22,38,25,41 | ENSMUSG00000115589,"ENSMUSG00000115589",0,0,0,0,0,0,0,0,0,0,0,0,0,0,0,0,0,0,0,0 |
| ENSMUSG00000036438,"ENSMUSG00000036438",4281,5428,7109,6695,6428,5449,5912,5021,5704,6915,6839,5397,5577,6120,5610,5924,5483,7100,5183,5938 | ENSMUSG00000074375,"ENSMUSG00000074375",1,0,0,0,0,0,0,0,0,0,1,0,0,0,0,0,0,0,0,0 | ENSMUSG00000115621,"ENSMUSG00000115621",0,0,0,0,0,0,0,0,0,0,0,0,0,0,1,0,0,0,0,0 |
| ENSMUSG00000001225,"ENSMUSG00000001225",0,0,1,2,0,1,1,0,1,0,5,4,1,2,1,1,1,2,2,0 | ENSMUSG00000097697,"ENSMUSG00000097697",10,2,4,5,10,6,6,2,3,8,4,6,4,9,1,3,3,6,4,2 | ENSMUSG00000065852,"ENSMUSG00000065852",0,0,0,0,0,0,0,0,0,0,0,0,0,0,0,0,0,0,0,0 |
| ENSMUSG00000032081,"ENSMUSG00000032081",0,1,0,0,0,0,1,1,2,0,0,0,0,0,0,0,0,0,3,4 | ENSMUSG00000112847,"ENSMUSG00000112847",12,8,3,8,10,10,4,14,6,16,11,8,13,6,7,9,13,8,5,1 | ENSMUSG00000099751,"ENSMUSG00000099751",0,0,0,0,0,0,0,0,0,0,0,0,0,0,0,0,0,0,0,0 |
| ENSMUSG00000093930,"ENSMUSG00000093930",3401,3159,3232,5024,4171,3032,3205,2740,3457,3148,4328,4432,3135,3439,2946,3466,2852,3663,3139,2977 | ENSMUSG00000047935,"ENSMUSG00000047935",0,0,0,0,1,2,0,1,0,1,0,0,0,0,0,2,0,0,0,0 | ENSMUSG00000115563,"ENSMUSG00000115563",0,0,0,0,1,1,0,0,0,0,1,0,0,1,0,0,0,0,0,0 |
| ENSMUSG00000034320,"ENSMUSG00000034320",1388,1135,1522,1592,1624,1417,1203,1161,1235,1318,1730,1206,1450,1311,1272,1560,1292,1786,1117,1158 | ENSMUSG00000033491,"ENSMUSG00000033491",2,15,41,18,22,30,22,44,35,24,19,22,28,12,15,35,24,29,17,25 | ENSMUSG00000115068,"ENSMUSG00000115068",0,0,0,0,0,0,0,0,0,0,0,0,1,0,0,0,0,0,0,0 |
| ENSMUSG00000060962,"ENSMUSG00000060962",10394,23418,27332,37113,24206,17902,27939,18872,25400,22259,32690,28183,26866,27743,23709,27787,20864,31191,28275,23713 | ENSMUSG00000103703,"ENSMUSG00000103703",0,4,1,0,5,0,1,1,2,1,1,2,3,3,0,1,3,0,3,0 | ENSMUSG00000077511,"ENSMUSG00000077511",0,0,0,0,0,0,0,0,0,0,0,0,0,0,0,0,0,0,0,0 |
| ENSMUSG00000075706,"ENSMUSG00000075706",1036,1232,1882,1338,1563,1429,1296,1312,1250,1530,1567,1064,1863,1256,1278,1514,1283,1956,1561,1645 | ENSMUSG00000086075,"ENSMUSG00000086075",27,30,26,20,33,29,23,23,32,35,23,23,18,22,24,46,24,24,27,33 | ENSMUSG00000115169,"ENSMUSG00000115169",0,0,0,0,0,0,0,0,0,0,0,0,0,0,0,0,0,0,0,0 |
| ENSMUSG00000063870,"ENSMUSG00000063870",3910,5500,7035,6329,6568,6314,6120,5829,5914,6399,6850,5037,7107,6401,5944,6814,5849,7512,5273,6503 | ENSMUSG00000094262,"ENSMUSG00000094262",0,0,0,0,0,0,0,0,0,0,0,0,0,0,0,0,0,0,0,0 | ENSMUSG00000116092,"ENSMUSG00000116092",0,0,0,0,0,0,0,0,0,0,0,0,0,0,0,0,0,0,0,0 |
| ENSMUSG00000024313,"ENSMUSG00000024313",0,0,0,0,0,0,0,0,0,3,0,0,0,0,0,0,0,0,0,0 | ENSMUSG00000110781,"ENSMUSG00000110781",0,0,0,0,0,0,0,0,0,0,0,0,0,0,0,0,0,0,0,0 | ENSMUSG00000101983,"ENSMUSG00000101983",0,0,0,0,0,0,0,0,0,0,0,0,0,0,0,0,0,0,0,0 |
| ENSMUSG00000028494,"ENSMUSG00000028494",3387,3670,5108,4171,3310,3835,4126,4367,4217,5565,4250,2354,5110,4712,3656,4821,4328,5799,3236,4978 | ENSMUSG00000113472,"ENSMUSG00000113472",4,7,11,8,15,10,12,17,11,7,19,10,16,12,8,9,11,12,1,8 | ENSMUSG00000101140,"ENSMUSG00000101140",0,0,0,0,0,0,0,0,0,0,0,0,0,0,0,0,0,0,0,0 |
| ENSMUSG00000027366,"ENSMUSG00000027366",8232,5635,6990,8515,7889,6485,5908,5769,6177,6772,7137,5957,6197,6546,6598,7741,6608,7929,4538,6090 | ENSMUSG00000097163,"ENSMUSG00000097163",1,2,6,1,1,2,6,3,0,4,2,1,0,1,1,1,1,0,0,0 | ENSMUSG00000100462,"ENSMUSG00000100462",0,0,0,0,1,0,0,0,0,0,0,0,0,0,0,1,0,0,0,0 |
| ENSMUSG00000041577,"ENSMUSG00000041577",1009,1313,1780,1709,1596,1759,1521,1375,1218,1556,1581,1142,1786,1437,1459,1509,1450,2126,1310,1695 | ENSMUSG00000107814,"ENSMUSG00000107814",10,5,11,7,10,5,13,18,6,6,6,10,7,10,7,4,8,8,6,13 | ENSMUSG00000101289,"ENSMUSG00000101289",0,0,0,0,0,0,0,0,0,0,0,0,0,0,0,0,0,0,0,0 |

| ENSMUSG00000004040,"ENSMUSG00000004040",10892,12471,12986,15408,14127,12353,13957,10284,12973,13664,12755,11949,10517,14469,11771,14584,11807,15231,10088,11456 | ENSMUSG00000025808,"ENSMUSG00000025808",6,6,12,2,4,7,0,1,7,6,3,0,5,6,6,2,6,10,1,9 | ENSMUSG00000116272,"ENSMUSG00000116272",0,0,0,0,0,0,0,0,0,0,0,0,0,0,0,0,0,0,0,0 |
| --- | --- | --- |
| ENSMUSG00000030738,"ENSMUSG00000030738",6067,6393,7817,8178,7648,6788,7113,5292,6464,6678,7368,6272,5944,6747,6544,7881,6269,7933,5981,6661 | ENSMUSG00000085684,"ENSMUSG00000085684",8,13,16,12,24,17,11,13,12,33,14,7,11,20,14,26,10,23,10,19 | ENSMUSG00000089242,"ENSMUSG00000089242",0,0,0,0,0,0,0,0,0,0,0,0,0,0,0,0,0,0,0,0 |
| ENSMUSG00000018927,"ENSMUSG00000018927",9823,13510,19413,15290,15968,16404,11678,10821,11990,17668,13648,11181,12521,15722,12813,15088,12914,14641,10801,12260 | ENSMUSG00000085531,"ENSMUSG00000085531",7,22,13,21,20,14,4,3,6,2,19,8,14,9,11,16,11,24,13,16 | ENSMUSG00000116162,"ENSMUSG00000116162",0,0,0,0,0,0,0,0,0,0,0,0,0,0,0,0,0,0,0,0 |
| ENSMUSG00000025508,"ENSMUSG00000025508",4799,5368,7823,6826,6013,5834,5449,4774,5008,6169,6766,5448,6296,6153,6115,7222,5376,7949,6784,6126 | ENSMUSG00000085143,"ENSMUSG00000085143",3,1,7,6,8,4,3,2,5,7,5,2,2,7,7,2,2,6,5,8 | ENSMUSG00000092751,"ENSMUSG00000092751",0,0,0,0,0,0,0,0,0,0,0,0,0,0,0,0,0,0,0,0 |
| ENSMUSG00000027790,"ENSMUSG00000027790",0,0,0,0,0,0,0,0,0,0,0,0,0,0,0,0,0,0,0,0 | ENSMUSG00000104918,"ENSMUSG00000104918",4,5,10,9,9,8,8,8,8,8,8,4,6,8,12,3,3,12,8,9 | ENSMUSG00000064764,"ENSMUSG00000064764",0,0,0,0,0,0,0,0,0,0,0,0,0,0,0,0,0,0,0,0 |
| ENSMUSG00000063316,"ENSMUSG00000063316",5741,6060,7973,6983,6479,6552,6438,5374,5744,6720,7355,5716,6830,6647,6213,7163,5986,8351,6699,6646 | ENSMUSG00000096105,"ENSMUSG00000096105",0,0,0,0,0,0,0,0,0,0,0,0,0,0,0,0,0,0,0,0 | ENSMUSG00000116204,"ENSMUSG00000116204",0,0,0,0,0,0,0,0,0,0,1,2,0,0,0,0,0,0,0,0 |
| ENSMUSG00000014867,"ENSMUSG00000014867",4924,4875,6580,6460,6585,6117,5915,5115,5368,5559,5983,4709,5783,5759,5567,6273,5163,6765,5225,5804 | ENSMUSG00000079598,"ENSMUSG00000079598",16,19,20,26,22,20,13,10,22,19,22,22,25,15,20,14,21,28,25,20 | ENSMUSG00000116428,"ENSMUSG00000116428",0,0,0,0,0,0,0,0,1,0,0,0,0,0,0,0,0,1,0,0 |
| ENSMUSG00000031799,"ENSMUSG00000031799",8366,9063,10995,11761,11348,10398,10821,8810,9447,10502,10766,9265,9139,10355,9724,11310,9759,12179,7775,9704 | ENSMUSG00000022586,"ENSMUSG00000022586",17,18,27,19,25,26,28,36,15,18,25,19,43,20,27,61,31,38,23,34 | ENSMUSG00000047295,"ENSMUSG00000047295",0,0,0,0,0,0,0,0,0,0,0,0,0,0,2,0,0,0,0,0 |
| ENSMUSG00000014956,"ENSMUSG00000014956",7054,4239,4852,5713,5474,4428,4907,3740,4539,5188,5231,4735,4332,4395,4636,5071,4415,5395,3462,4447 | ENSMUSG00000117321,"ENSMUSG00000117321",13,9,3,9,15,10,7,4,7,5,10,8,3,7,4,2,10,6,6,6 | ENSMUSG00000116493,"ENSMUSG00000116493",0,0,0,0,0,0,0,0,0,0,1,0,0,0,0,0,0,0,0,0 |
| ENSMUSG00000023004,"ENSMUSG00000023004",3762,3835,5392,4634,5202,5128,4722,4468,4027,4300,4687,3629,5079,4225,4425,4534,3751,5799,5450,4913 | ENSMUSG00000106157,"ENSMUSG00000106157",11,6,5,5,9,11,6,9,14,4,2,4,8,0,4,3,4,10,2,3 | ENSMUSG00000116131,"ENSMUSG00000116131",0,0,0,0,0,0,0,0,0,0,0,0,0,0,0,0,0,0,0,0 |
| ENSMUSG00000030688,"ENSMUSG00000030688",371,624,749,743,775,678,785,596,628,617,804,707,696,687,720,803,653,926,704,731 | ENSMUSG00000109262,"ENSMUSG00000109262",9,4,5,5,4,4,8,4,3,4,4,9,6,15,12,8,10,7,4,1 | ENSMUSG00000096448,"ENSMUSG00000096448",0,0,0,0,0,0,0,0,0,0,1,0,0,0,0,0,1,1,0,1 |
| ENSMUSG00000025651,"ENSMUSG00000025651",2980,2909,3862,3428,3568,3234,3349,2766,3225,3322,3846,2940,3408,3141,3095,3507,2993,4000,3519,3731 | ENSMUSG00000080364,"ENSMUSG00000080364",5,11,13,18,12,3,18,9,13,7,11,14,6,12,7,13,12,12,15,6 | ENSMUSG00000077443,"ENSMUSG00000077443",0,0,0,0,0,0,0,0,0,0,0,0,0,0,0,0,0,0,0,0 |
| ENSMUSG00000060803,"ENSMUSG00000060803",329,440,656,511,552,531,507,556,497,504,574,350,642,469,562,581,473,604,620,660 | ENSMUSG00000028360,"ENSMUSG00000028360",10,8,15,16,10,15,9,13,6,13,11,7,15,18,20,25,11,15,7,7 | ENSMUSG00000050328,"ENSMUSG00000050328",0,0,0,0,0,1,1,0,0,0,0,0,2,0,0,0,0,0,0,0 |
| ENSMUSG00000018166,"ENSMUSG00000018166",2561,2736,3159,3634,3584,2630,3232,2751,3234,2751,3478,3242,3077,3184,3014,3902,2543,3889,3066,3139 | ENSMUSG00000105896,"ENSMUSG00000105896",1,0,3,3,3,3,1,2,0,2,4,0,1,1,1,1,0,1,3,0 | ENSMUSG00000116455,"ENSMUSG00000116455",0,0,0,0,0,2,0,0,0,0,0,0,0,0,0,0,0,0,0,0 |
| ENSMUSG00000011179,"ENSMUSG00000011179",14907,12905,14883,13281,15097,12966,15096,7336,12264,16767,8734,10302,6724,14496,11145,13852,11436,13109,7924,11495 | ENSMUSG00000048636,"ENSMUSG00000048636",14,21,13,22,20,19,21,7,15,20,15,8,16,29,18,22,11,15,4,14 | ENSMUSG00000116348,"ENSMUSG00000116348",0,0,0,0,0,0,0,0,0,0,0,0,0,0,0,1,0,0,0,0 |
| ENSMUSG00000035885,"ENSMUSG00000035885",1334,2222,3337,2884,2412,2460,2555,2196,2416,2737,3141,2250,2901,3005,2616,3133,2539,3576,3169,2872 | ENSMUSG00000092200,"ENSMUSG00000092200",0,2,0,1,0,0,0,1,1,0,0,0,2,0,0,0,0,1,0,0 | ENSMUSG00000101565,"ENSMUSG00000101565",0,0,0,0,2,0,0,0,0,0,0,0,0,0,0,0,0,0,0,0 |
| ENSMUSG00000057113,"ENSMUSG00000057113",5197,8098,9109,9238,8440,8380,8666,6126,7771,9114,9515,7616,6608,7745,7269,8071,7253,9167,6639,8036 | ENSMUSG00000104830,"ENSMUSG00000104830",0,1,3,1,8,1,1,1,1,4,5,4,2,2,4,4,1,4,3,0 | ENSMUSG00000082554,"ENSMUSG00000082554",0,0,0,0,0,0,0,0,0,0,0,0,0,0,0,0,0,0,0,0 |
| ENSMUSG00000049493,"ENSMUSG00000049493",15,6,2,13,14,5,4,4,11,6,5,7,8,11,8,13,10,14,8,1 | ENSMUSG00000070498,"ENSMUSG00000070498",11,8,5,11,8,10,3,2,6,12,4,11,2,8,3,3,7,8,1,3 | ENSMUSG00000075335,"ENSMUSG00000075335",0,0,0,0,0,0,0,0,0,1,2,0,0,0,0,0,0,1,0,0 |
| ENSMUSG00000025967,"ENSMUSG00000025967",4071,5331,6298,6147,5538,5553,5821,3948,5099,5524,5687,4846,5011,5019,4852,5887,4876,6289,5213,5212 | ENSMUSG00000000731,"ENSMUSG00000000731",0,4,4,0,3,4,0,0,5,2,5,0,1,3,4,3,1,5,11,3 | ENSMUSG00000116712,"ENSMUSG00000116712",0,0,0,0,0,0,0,0,0,0,0,0,0,0,0,0,0,0,0,0 |
| ENSMUSG00000025492,"ENSMUSG00000025492",11718,12598,16056,19578,15807,13094,13731,14216,14306,15318,17150,14061,16224,16191,15856,19816,15169,22424,15470,17527 | ENSMUSG00000087013,"ENSMUSG00000087013",11,3,1,4,10,6,1,0,4,0,4,3,6,2,5,3,6,10,7,1 | ENSMUSG00000065372,"ENSMUSG00000065372",0,0,1,0,0,0,0,0,0,0,0,0,0,0,0,0,0,0,0,0 |
| ENSMUSG00000001119,"ENSMUSG00000001119",4790,6206,10372,8861,7991,10762,8553,7520,7968,8410,8847,5676,9257,8395,7771,7101,7709,8007,6506,7760 | ENSMUSG00000098286,"ENSMUSG00000098286",7,5,9,9,14,7,12,12,11,10,3,5,7,15,12,8,12,9,2,9 | ENSMUSG00000116785,"ENSMUSG00000116785",0,0,0,0,0,0,0,0,0,0,0,0,0,0,0,2,0,0,0,0 |
| ENSMUSG00000006998,"ENSMUSG00000006998",6046,5638,7602,7354,6896,6343,6700,5611,6117,6259,7221,5774,6774,6571,6494,6977,5830,8212,5545,6841 | ENSMUSG00000091476,"ENSMUSG00000091476",2,1,14,9,8,13,5,2,3,2,18,9,13,12,1,12,3,15,5,6 | ENSMUSG00000077602,"ENSMUSG00000077602",0,0,1,0,0,0,0,0,0,0,0,0,0,0,0,0,0,0,0,0 |
| ENSMUSG00000018569,"ENSMUSG00000018569",56,63,64,56,102,75,79,43,81,83,42,47,42,95,84,98,62,79,50,100 | ENSMUSG00000071550,"ENSMUSG00000071550",0,2,0,3,1,4,1,3,2,1,2,1,1,2,0,0,1,7,2,3 | ENSMUSG00000091972,"ENSMUSG00000091972",0,0,0,0,1,0,0,0,0,1,0,0,0,0,0,0,0,0,0,0 |
| ENSMUSG00000025781,"ENSMUSG00000025781",2680,2663,3205,3055,3132,2760,2848,2340,2647,2831,3284,2560,2873,2728,2686,3064,2532,3371,2684,2963 | ENSMUSG00000042289,"ENSMUSG00000042289",1,11,15,8,6,10,6,6,4,9,5,5,8,3,2,10,3,5,11,4 | ENSMUSG00000095791,"ENSMUSG00000095791",0,0,0,0,0,0,0,0,0,0,0,0,0,0,0,0,0,0,1,0 |
| ENSMUSG00000020592,"ENSMUSG00000020592",9413,12273,13575,14591,13430,11499,14520,9378,12808,11952,12520,11920,10413,13496,11047,13783,10558,14936,10656,13271 | ENSMUSG00000110626,"ENSMUSG00000110626",18,22,33,30,40,35,31,27,31,75,35,32,37,41,21,52,22,46,28,38 | ENSMUSG00000115997,"ENSMUSG00000115997",0,0,0,0,0,0,2,0,0,0,0,0,0,0,0,0,0,0,0,0 |
| ENSMUSG00000020405,"ENSMUSG00000020405",4,28,28,27,19,12,13,12,12,14,24,23,15,31,24,19,10,28,13,13 | ENSMUSG00000113679,"ENSMUSG00000113679",5,7,6,2,6,8,5,16,5,10,5,2,15,7,2,11,3,10,4,2 | ENSMUSG00000077844,"ENSMUSG00000077844",0,0,0,0,0,0,0,0,0,0,0,0,0,0,0,0,0,0,0,0 |
| ENSMUSG00000046434,"ENSMUSG00000046434",3920,5903,6954,8012,6526,5785,6183,5019,5615,6302,7844,6269,5647,6412,5860,6167,5585,6690,5503,5504 | ENSMUSG00000076670,"ENSMUSG00000076670",0,0,0,0,0,0,0,0,0,0,0,0,0,0,0,0,0,0,0,0 | ENSMUSG00000116773,"ENSMUSG00000116773",1,0,0,0,0,0,0,0,0,0,0,0,0,2,0,0,1,0,0,0 |
| ENSMUSG00000000563,"ENSMUSG00000000563",2355,3339,3994,3959,3985,3578,3795,2878,3371,3920,3941,3225,3464,3454,3465,3680,3319,4238,3341,3685 | ENSMUSG00000112770,"ENSMUSG00000112770",9,7,9,7,4,4,5,6,5,7,10,7,11,6,7,5,6,6,11,2 | ENSMUSG00000065188,"ENSMUSG00000065188",0,0,1,0,0,0,0,0,0,0,0,0,0,1,0,0,0,1,0,0 |
| ENSMUSG00000079641,"ENSMUSG00000079641",8015,5591,6755,6146,5871,5847,6128,4777,5801,6901,6618,5422,5521,5560,5041,6591,5159,7543,3940,6266 | ENSMUSG00000094724,"ENSMUSG00000094724",10,6,7,18,11,14,6,6,4,9,6,14,14,7,7,13,5,13,8,6 | ENSMUSG00000095894,"ENSMUSG00000095894",0,1,1,0,0,0,0,0,0,0,0,0,1,1,0,0,0,0,0,0 |
| ENSMUSG00000036550,"ENSMUSG00000036550",3880,3828,4790,4705,4848,4611,4286,3974,4085,4560,4748,3829,4437,4298,3853,4564,3819,5032,3144,4039 | ENSMUSG00000087684,"ENSMUSG00000087684",18,21,44,34,30,33,40,29,27,24,33,24,32,31,41,39,25,40,29,35 | ENSMUSG00000065667,"ENSMUSG00000065667",0,0,0,0,0,0,0,0,0,0,0,0,0,0,0,0,0,0,0,0 |
| ENSMUSG00000036256,"ENSMUSG00000036256",1897,3374,5538,4366,3960,5118,4037,3614,3612,4192,4237,2567,5373,4095,3874,4413,3648,5165,3859,4470 | ENSMUSG00000104235,"ENSMUSG00000104235",1,4,10,1,4,4,0,3,3,3,5,3,5,1,1,3,5,11,3,2 | ENSMUSG00000099074,"ENSMUSG00000099074",0,0,0,1,0,0,0,0,1,0,0,0,0,0,0,0,0,0,0,0 |
| ENSMUSG00000026131,"ENSMUSG00000026131",3168,3909,4565,5103,4313,3881,4484,3362,3794,4210,5026,3822,4472,4114,3779,4329,3822,4911,2849,3537 | ENSMUSG00000097149,"ENSMUSG00000097149",8,3,23,4,12,2,9,3,10,17,10,9,5,10,3,4,3,6,7,3 | ENSMUSG00000096535,"ENSMUSG00000096535",0,0,0,0,0,0,0,0,0,0,0,0,0,0,0,0,0,0,0,0 |
| ENSMUSG00000022040,"ENSMUSG00000022040",58,59,93,62,78,47,80,74,76,70,80,49,61,73,53,63,63,86,72,89 | ENSMUSG00000111203,"ENSMUSG00000111203",3,5,3,4,2,2,3,6,2,4,4,1,2,9,2,7,6,9,3,0 | ENSMUSG00000116991,"ENSMUSG00000116991",0,0,0,0,0,0,0,0,0,0,0,0,0,0,0,0,0,2,0,0 |
| ENSMUSG00000038733,"ENSMUSG00000038733",5496,5103,6261,5942,6319,6033,4946,4626,5458,6341,6403,4751,6159,5546,5124,6470,5276,7163,4015,5172 | ENSMUSG00000076676,"ENSMUSG00000076676",0,0,0,0,0,0,0,0,0,0,0,0,0,0,0,0,0,0,0,0 | ENSMUSG00000116849,"ENSMUSG00000116849",0,0,0,0,0,0,0,0,0,0,0,0,0,0,0,0,0,0,0,0 |
| ENSMUSG00000018501,"ENSMUSG00000018501",2119,2671,3425,3317,3299,3102,2939,2587,2883,3151,3306,2472,3125,3081,2753,3110,2912,3325,2277,2726 | ENSMUSG00000096992,"ENSMUSG00000096992",36,39,33,33,14,26,45,23,40,37,40,33,33,23,27,26,13,36,19,23 | ENSMUSG00000116882,"ENSMUSG00000116882",0,0,0,0,0,0,0,0,0,0,0,0,0,0,0,0,0,0,0,0 |
| ENSMUSG00000029445,"ENSMUSG00000029445",0,0,0,0,0,0,2,1,1,0,0,0,3,0,0,0,1,1,3,4 | ENSMUSG00000086873,"ENSMUSG00000086873",5,10,4,16,8,6,6,2,8,7,13,9,2,7,8,13,1,8,14,3 | ENSMUSG00000105138,"ENSMUSG00000105138",2,0,0,0,0,0,0,0,0,0,0,0,0,0,0,0,0,0,0,0 |
| ENSMUSG00000046834,"ENSMUSG00000046834",19022,25372,34679,33063,23288,24897,18433,18054,20133,21267,31584,16541,38661,26334,13817,31654,18095,24722,24070,18073 | ENSMUSG00000065637,"ENSMUSG00000065637",7,11,14,9,8,11,14,7,13,8,8,12,15,16,9,25,7,8,3,21 | ENSMUSG00000116896,"ENSMUSG00000116896",0,0,0,0,0,0,0,0,0,0,0,0,0,0,0,0,0,0,0,0 |
| ENSMUSG00000019943,"ENSMUSG00000019943",3569,3707,4611,4192,4668,4174,3651,3369,3537,4884,4311,3217,4226,4309,3708,4300,3883,4747,2763,3860 | ENSMUSG00000020916,"ENSMUSG00000020916",0,0,1,0,0,26,0,0,1,0,0,0,0,0,0,1,0,4,0,2 | ENSMUSG00000116955,"ENSMUSG00000116955",0,0,0,0,1,0,0,0,0,0,0,0,0,0,0,1,0,0,1,0 |
| ENSMUSG00000028063,"ENSMUSG00000028063",5844,8174,10639,11125,8600,9081,9011,7084,8535,11924,9391,7083,8596,10282,8124,9397,8394,11156,8035,9385 | ENSMUSG00000114613,"ENSMUSG00000114613",0,0,0,0,0,0,0,0,0,0,0,0,0,0,0,0,0,0,0,0 | ENSMUSG00000099104,"ENSMUSG00000099104",0,0,0,0,0,0,0,0,1,0,0,0,1,0,0,0,0,0,0,0 |
| ENSMUSG00000027111,"ENSMUSG00000027111",9385,8528,9551,11097,9997,7858,10147,6400,8884,9050,9301,9456,6323,8623,8199,9461,7494,9667,5780,7795 | ENSMUSG00000065208,"ENSMUSG00000065208",22,4,10,23,14,10,9,9,4,8,14,7,3,3,11,15,11,6,3,4 | ENSMUSG00000117290,"ENSMUSG00000117290",0,0,0,1,0,0,0,0,0,0,2,0,0,0,0,0,0,0,0,0 |
| ENSMUSG00000032504,"ENSMUSG00000032504",5441,4903,5747,5873,5747,5465,5251,3874,4817,5515,5375,4599,4777,5220,4945,5759,4973,6203,3697,4757 | ENSMUSG00000026736,"ENSMUSG00000026736",2,4,16,9,4,8,10,12,7,13,9,7,9,17,7,8,7,14,2,11 | ENSMUSG00000117282,"ENSMUSG00000117282",0,0,2,0,0,0,0,0,0,1,0,0,0,0,0,0,0,0,0,0 |
| ENSMUSG00000017466,"ENSMUSG00000017466",4506,5682,8740,6997,7214,7343,5776,5482,5790,7168,7343,4715,7937,5940,5683,6432,6258,7683,5176,6386 | ENSMUSG00000068674,"ENSMUSG00000068674",7,9,9,15,8,8,7,8,10,9,12,6,8,11,11,11,9,8,5,12 | ENSMUSG00000116532,"ENSMUSG00000116532",0,0,0,0,0,0,0,0,0,0,0,0,0,0,0,0,0,0,0,0 |
| ENSMUSG00000070327,"ENSMUSG00000070327",11569,10099,11080,13889,11626,11458,11151,11039,11778,11336,11347,7901,11442,12133,11869,15341,12186,16336,7149,10721 | ENSMUSG00000103149,"ENSMUSG00000103149",2,0,1,6,1,0,1,3,6,3,4,3,1,2,4,2,0,4,1,1 | ENSMUSG00000090315,"ENSMUSG00000090315",0,0,0,0,0,0,0,0,0,0,0,0,0,0,0,0,0,0,0,0 |
| ENSMUSG00000031886,"ENSMUSG00000031886",12,18,37,15,13,15,19,13,16,17,15,10,6,13,22,9,29,17,15,11 | ENSMUSG00000105102,"ENSMUSG00000105102",55,52,70,44,42,26,52,19,41,42,47,36,16,36,47,42,23,31,31,29 | ENSMUSG00000067951,"ENSMUSG00000067951",0,0,1,0,0,0,0,0,0,0,0,2,1,0,0,0,0,0,0,0 |
| ENSMUSG00000003429,"ENSMUSG00000003429",2817,5087,6558,5888,5615,5215,5329,4388,4818,5338,6042,5233,5617,5705,5321,6185,4704,6733,5755,5681 | ENSMUSG00000100121,"ENSMUSG00000100121",13,22,10,32,29,16,15,14,15,15,14,15,12,24,17,18,29,27,7,16 | ENSMUSG00000096857,"ENSMUSG00000096857",0,0,0,0,0,0,0,0,0,0,0,0,0,0,0,0,0,0,0,0 |
| ENSMUSG00000001435,"ENSMUSG00000001435",736,1473,2536,1606,1567,2312,1679,1654,1585,1820,1978,1084,2023,1838,1655,2242,1741,2650,1612,2084 | ENSMUSG00000110360,"ENSMUSG00000110360",2,1,1,3,6,2,7,5,7,4,6,1,2,6,8,0,2,8,6,2 | ENSMUSG00000098826,"ENSMUSG00000098826",0,0,0,0,0,0,0,0,0,0,0,0,0,0,0,0,0,0,0,0 |
| ENSMUSG00000021771,"ENSMUSG00000021771",3499,3105,3664,3770,3497,3133,3402,2913,3360,3425,3829,3010,3230,3219,3113,3545,3090,3749,2893,3376 | ENSMUSG00000031789,"ENSMUSG00000031789",6,15,15,14,4,11,15,20,10,13,14,6,7,7,10,13,15,21,13,12 | ENSMUSG00000098445,"ENSMUSG00000098445",0,0,0,0,0,0,0,0,0,2,0,0,0,0,0,0,0,0,0,0 |
| ENSMUSG00000021876,"ENSMUSG00000021876",3691,3591,5607,4662,4697,4048,3363,3329,3509,4088,4678,3553,4391,3894,3614,4032,3443,4323,3267,3601 | ENSMUSG00000034706,"ENSMUSG00000034706",6,5,10,7,9,5,2,3,4,7,10,6,7,8,7,11,9,10,9,13 | ENSMUSG00000037334,"ENSMUSG00000037334",0,0,0,0,0,0,0,0,0,0,0,0,0,0,0,0,0,1,0,0 |
| ENSMUSG00000038991,"ENSMUSG00000038991",1912,2681,3626,3450,3323,3765,3131,2725,2748,2784,3423,2555,3334,2957,2916,3245,2796,3588,2531,2993 | ENSMUSG00000083097,"ENSMUSG00000083097",10,11,15,10,19,10,11,6,6,16,12,15,7,6,6,7,7,8,2,13 | ENSMUSG00000095603,"ENSMUSG00000095603",0,0,0,0,0,0,0,0,0,0,0,0,0,0,0,3,0,0,0,0 |
| ENSMUSG00000001440,"ENSMUSG00000001440",5104,5086,6724,6292,5987,6068,5641,4549,5003,5580,6697,5280,5402,5607,5053,5807,5193,6482,4688,5261 | ENSMUSG00000087030,"ENSMUSG00000087030",0,0,0,1,0,0,0,0,0,0,0,0,0,0,0,0,0,0,0,0 | ENSMUSG00000070209,"ENSMUSG00000070209",0,0,0,0,1,0,0,1,0,0,0,0,0,0,0,0,0,0,0,0 |
| ENSMUSG00000008730,"ENSMUSG00000008730",4953,4854,4817,4936,6063,4871,5279,3776,4897,6493,4785,4247,4214,5764,4770,5602,4888,5630,3298,4362 | ENSMUSG00000068324,"ENSMUSG00000068324",2,8,15,11,4,5,6,4,5,6,12,14,7,4,10,5,8,16,16,8 | ENSMUSG00000065743,"ENSMUSG00000065743",0,0,0,0,0,0,0,0,0,0,0,0,0,0,0,0,0,0,0,0 |
| ENSMUSG00000030795,"ENSMUSG00000030795",2999,5115,6090,6204,5645,5445,5110,4199,5065,5703,6838,5411,5257,5960,5474,6132,4927,6705,5711,5140 | ENSMUSG00000026065,"ENSMUSG00000026065",5,8,9,10,5,7,5,6,8,3,17,6,4,8,6,13,7,18,6,8 | ENSMUSG00000062472,"ENSMUSG00000062472",0,0,0,0,0,0,0,0,0,0,0,0,0,0,0,0,0,0,0,0 |
| ENSMUSG00000030655,"ENSMUSG00000030655",6204,5046,5677,5381,6082,5776,5226,4292,5015,5894,5162,4531,5076,5401,4751,5431,5125,6110,3519,4424 | ENSMUSG00000020052,"ENSMUSG00000020052",0,0,2,0,0,0,2,0,0,0,0,0,0,0,0,0,0,0,0,0 | ENSMUSG00000065027,"ENSMUSG00000065027",0,0,0,0,1,0,0,0,0,0,0,0,0,0,0,0,0,0,0,0 |
| ENSMUSG00000022665,"ENSMUSG00000022665",814,985,1274,1204,1221,1483,1084,1203,990,1062,1200,855,1676,1117,1149,999,1133,1465,1135,1425 | ENSMUSG00000052143,"ENSMUSG00000052143",5,13,17,42,30,7,17,20,12,15,15,17,18,10,16,17,30,32,11,19 | ENSMUSG00000117283,"ENSMUSG00000117283",0,0,0,0,0,0,0,0,0,0,0,0,0,0,0,0,0,0,0,0 |
| ENSMUSG00000020612,"ENSMUSG00000020612",5401,5439,6633,6153,6438,6187,5817,4913,5684,6660,5920,4534,5746,6122,5379,6229,5585,6938,4577,5832 | ENSMUSG00000087064,"ENSMUSG00000087064",18,11,6,8,8,3,7,8,6,14,16,10,10,10,5,11,13,13,11,11 | ENSMUSG00000097095,"ENSMUSG00000097095",0,0,0,0,0,0,0,0,0,0,0,0,0,0,0,0,0,0,0,0 |
| ENSMUSG00000034892,"ENSMUSG00000034892",859,5260,7252,6558,4464,4294,4298,4206,5378,6441,7331,5828,5909,6611,5615,7290,5419,7752,4381,6494 | ENSMUSG00000116815,"ENSMUSG00000116815",14,11,19,19,34,11,9,22,31,18,25,22,9,19,19,21,15,18,14,19 | ENSMUSG00000117298,"ENSMUSG00000117298",0,0,0,0,0,0,0,0,0,0,0,0,0,0,0,0,0,0,0,0 |
| ENSMUSG00000020741,"ENSMUSG00000020741",1557,2045,2165,2606,2143,2009,2360,1516,1963,2042,2693,2235,1815,2026,1984,2115,1805,2300,2006,1779 | ENSMUSG00000115759,"ENSMUSG00000115759",34,27,39,33,57,80,36,29,40,41,51,22,24,32,35,19,48,56,34,50 | ENSMUSG00000117276,"ENSMUSG00000117276",0,0,0,0,0,0,0,0,0,1,0,0,0,0,0,0,0,0,0,0 |
| ENSMUSG00000021033,"ENSMUSG00000021033",173,247,266,230,303,283,243,210,275,259,290,178,303,285,247,231,305,303,227,272 | ENSMUSG00000026344,"ENSMUSG00000026344",4,5,16,4,13,10,7,2,4,5,2,7,2,12,14,13,7,11,9,3 | ENSMUSG00000117075,"ENSMUSG00000117075",2,0,0,0,1,0,0,1,0,0,0,0,0,0,0,0,0,0,0,0 |
| ENSMUSG00000018567,"ENSMUSG00000018567",3008,4601,6692,5515,6029,5519,5194,5170,4965,6036,5984,4275,6305,5898,5343,5744,5245,6419,4193,6068 | ENSMUSG00000051081,"ENSMUSG00000051081",19,27,45,74,41,74,70,29,21,31,80,32,17,38,48,42,36,8,67,9 | ENSMUSG00000117047,"ENSMUSG00000117047",0,0,0,0,0,0,0,0,1,0,0,0,0,0,0,0,0,0,0,0 |
| ENSMUSG00000005087,"ENSMUSG00000005087",15390,18197,18607,20760,20987,17444,20278,13625,18509,20532,17004,16439,13388,20346,16156,20119,16843,20046,11259,15922 | ENSMUSG00000062846,"ENSMUSG00000062846",5,6,10,14,13,24,16,13,18,7,12,14,10,9,11,10,13,14,5,16 | ENSMUSG00000079333,"ENSMUSG00000079333",0,0,0,0,0,0,0,0,0,0,0,0,0,0,0,0,0,0,0,0 |
| ENSMUSG00000025355,"ENSMUSG00000025355",18246,18068,31261,29232,23902,18437,18702,13755,22254,33082,17734,11168,16388,31446,21649,19983,22422,13520,10685,13755 | ENSMUSG00000109291,"ENSMUSG00000109291",4,1,3,0,1,5,0,0,2,0,1,2,1,1,0,0,2,0,1,1 | ENSMUSG00000100634,"ENSMUSG00000100634",0,0,0,0,0,0,0,0,0,0,0,0,0,0,0,0,0,0,0,0 |
| ENSMUSG00000020149,"ENSMUSG00000020149",7908,5807,7460,7670,7579,6795,6669,5698,6947,7095,7241,5728,6131,6893,6288,7448,6234,7397,5749,6400 | ENSMUSG00000076470,"ENSMUSG00000076470",2,3,7,3,7,5,6,2,3,7,7,4,10,11,7,13,6,14,12,10 | ENSMUSG00000100472,"ENSMUSG00000100472",0,0,0,0,0,0,0,0,0,0,0,0,0,0,0,0,0,0,0,0 |
| ENSMUSG00000021484,"ENSMUSG00000021484",2939,3313,4492,4630,4205,3729,3855,3516,3696,3780,4286,3335,3893,3965,3818,4452,3627,4642,3557,4054 | ENSMUSG00000108447,"ENSMUSG00000108447",9,4,5,10,12,13,3,8,10,4,17,9,2,5,8,10,8,14,4,1 | ENSMUSG00000099463,"ENSMUSG00000099463",0,0,0,0,0,0,0,0,0,0,0,0,0,0,0,0,0,0,0,0 |
| ENSMUSG00000028618,"ENSMUSG00000028618",2722,2374,3043,2951,2858,2658,2430,2148,2364,2470,2778,2148,2653,2317,2261,2549,2155,2812,2164,2378 | ENSMUSG00000087429,"ENSMUSG00000087429",6,15,4,12,16,5,7,6,9,15,16,8,2,7,2,8,11,8,2,7 | ENSMUSG00000101491,"ENSMUSG00000101491",0,0,0,0,0,0,0,0,0,0,0,0,0,0,0,0,0,0,0,0 |
| ENSMUSG00000034708,"ENSMUSG00000034708",6296,6170,10893,8331,7984,8097,6221,8355,7465,8285,9263,5352,10654,7362,7603,9591,8065,11451,7283,8996 | ENSMUSG00000114004,"ENSMUSG00000114004",4,6,7,2,9,1,1,3,5,0,4,4,2,7,0,3,5,7,4,2 | ENSMUSG00000100973,"ENSMUSG00000100973",0,0,0,0,0,0,0,0,0,0,0,0,0,0,0,0,0,0,0,0 |
| ENSMUSG00000029322,"ENSMUSG00000029322",2294,2423,2586,3257,3027,2831,2562,3174,2956,2990,2693,1635,3413,3388,2761,4100,3215,5079,2506,3422 | ENSMUSG00000031766,"ENSMUSG00000031766",2,0,8,7,12,7,3,5,4,5,5,1,8,4,2,5,2,8,4,3 | ENSMUSG00000089054,"ENSMUSG00000089054",0,0,0,0,0,0,0,0,0,0,0,0,0,0,0,0,0,0,0,0 |
| ENSMUSG00000001123,"ENSMUSG00000001123",2925,2465,3538,5826,3839,3000,2937,3622,3356,3300,4116,3468,3622,3584,3745,4767,3454,4876,3021,3545 | ENSMUSG00000115918,"ENSMUSG00000115918",10,10,9,3,14,8,5,10,1,6,16,5,5,9,12,8,17,12,6,4 | ENSMUSG00000093274,"ENSMUSG00000093274",0,0,0,0,0,0,0,0,0,0,0,0,0,0,0,0,0,0,0,0 |
| ENSMUSG00000025647,"ENSMUSG00000025647",3479,4041,5886,6013,5033,4867,4752,4675,4719,5247,5447,3986,5903,5466,4571,5646,4955,6645,4681,5337 | ENSMUSG00000102562,"ENSMUSG00000102562",6,2,12,8,21,7,3,7,5,3,15,3,17,2,15,5,3,6,11,3 | ENSMUSG00000096934,"ENSMUSG00000096934",1,0,0,0,0,2,0,0,0,0,0,0,0,0,0,0,0,0,0,0 |
| ENSMUSG00000030707,"ENSMUSG00000030707",3695,4861,7168,4535,6069,6425,4893,5548,4346,5727,6033,3382,7926,6212,5407,6008,5648,7984,5441,6247 | ENSMUSG00000096056,"ENSMUSG00000096056",4,7,3,3,1,9,5,2,7,2,11,5,9,1,4,2,7,4,5,6 | ENSMUSG00000097507,"ENSMUSG00000097507",0,0,0,0,0,0,0,0,0,0,0,0,0,0,0,1,0,0,0,0 |
| ENSMUSG00000039202,"ENSMUSG00000039202",1644,1882,2391,2552,2507,2304,2214,1773,2185,2426,2275,1679,2147,2429,2045,2379,2217,2459,1488,2028 | ENSMUSG00000050511,"ENSMUSG00000050511",2,2,4,2,1,4,2,0,1,4,1,1,3,2,0,3,4,2,0,2 | ENSMUSG00000083766,"ENSMUSG00000083766",0,0,0,0,0,0,0,0,4,0,0,0,0,0,0,0,0,0,0,0 |
| ENSMUSG00000054364,"ENSMUSG00000054364",3397,3320,3781,2904,3798,3483,3350,3612,3534,4170,3151,2153,4626,3884,3343,3699,3444,4139,2893,3956 | ENSMUSG00000112105,"ENSMUSG00000112105",7,11,16,8,10,9,9,1,1,9,10,11,4,15,9,16,11,18,4,13 | ENSMUSG00000095482,"ENSMUSG00000095482",0,0,0,0,0,0,0,0,0,0,0,0,0,0,0,0,0,0,0,0 |
| ENSMUSG00000036707,"ENSMUSG00000036707",5599,4838,5331,5847,5699,4837,5134,3805,4961,5690,4946,4467,4519,4989,4457,5431,4773,5753,3629,4453 | ENSMUSG00000103015,"ENSMUSG00000103015",0,2,4,9,4,3,0,0,3,4,1,0,6,2,1,2,0,2,0,0 | ENSMUSG00000105951,"ENSMUSG00000105951",0,0,0,0,0,0,0,0,0,0,0,0,0,0,0,0,0,0,0,0 |
| ENSMUSG00000020402,"ENSMUSG00000020402",3875,3855,4599,5005,4631,3671,4385,3716,4052,4019,4818,4205,4289,4263,3931,4657,3681,5099,3911,4698 | ENSMUSG00000055494,"ENSMUSG00000055494",10,16,7,3,7,17,17,6,8,6,13,7,6,15,6,8,5,5,15,10 | ENSMUSG00000098721,"ENSMUSG00000098721",0,0,0,0,0,0,0,0,0,0,0,0,0,0,0,0,0,0,0,0 |
| ENSMUSG00000026880,"ENSMUSG00000026880",7980,8514,9183,11793,9797,7581,10149,6899,9045,8870,8401,8901,5829,9141,8431,9730,7814,9300,7619,7363 | ENSMUSG00000091881,"ENSMUSG00000091881",8,10,12,16,11,13,7,3,11,9,9,8,8,12,15,3,8,16,7,5 | ENSMUSG00000098305,"ENSMUSG00000098305",0,0,0,0,0,0,0,0,0,0,0,0,0,0,0,0,0,0,0,0 |
| ENSMUSG00000018846,"ENSMUSG00000018846",1874,1343,1403,1478,1590,1455,1285,1079,1353,1391,1541,1344,1261,1321,1254,1408,1391,1536,924,1193 | ENSMUSG00000105382,"ENSMUSG00000105382",2,1,6,3,12,10,2,7,7,5,6,3,8,9,6,6,9,7,2,6 | ENSMUSG00000077402,"ENSMUSG00000077402",0,0,0,0,0,0,0,0,0,0,0,0,0,0,0,0,0,0,0,0 |
| ENSMUSG00000015165,"ENSMUSG00000015165",3145,4801,6126,6044,5918,4753,4980,4560,4974,5231,6162,5107,5362,5109,5105,5570,4838,6326,4901,5263 | ENSMUSG00000040364,"ENSMUSG00000040364",2,5,8,4,7,5,2,6,17,8,8,4,4,10,8,10,7,9,3,12 | ENSMUSG00000098346,"ENSMUSG00000098346",0,0,0,0,0,0,0,0,0,0,1,0,0,0,0,0,0,0,0,0 |
| ENSMUSG00000020241,"ENSMUSG00000020241",4308,6112,9198,8804,7487,9883,7768,6549,7452,8173,8056,5337,8523,8091,7661,6510,7002,7240,5976,6890 | ENSMUSG00000117042,"ENSMUSG00000117042",2,1,3,1,1,3,0,1,0,2,1,0,0,2,1,0,2,0,0,2 | ENSMUSG00000044994,"ENSMUSG00000044994",0,0,0,0,2,0,0,0,0,0,0,0,0,0,0,0,0,0,0,0 |
| ENSMUSG00000026623,"ENSMUSG00000026623",2489,2389,2905,2604,3062,2938,2406,2335,2445,2883,2538,1983,2538,2677,2625,3001,2377,3600,1934,2565 | ENSMUSG00000117027,"ENSMUSG00000117027",8,8,15,13,13,11,14,7,10,8,21,16,12,21,10,7,12,12,6,3 | ENSMUSG00000067522,"ENSMUSG00000067522",0,0,0,0,0,0,0,0,0,0,0,0,0,0,0,0,0,0,0,0 |
| ENSMUSG00000112023,"ENSMUSG00000112023",23733,24041,22133,17179,25287,30292,21907,18372,21717,30882,16955,13669,19869,29550,17380,25591,19499,27276,12136,20762 | ENSMUSG00000097564,"ENSMUSG00000097564",1,8,6,5,2,5,6,5,4,4,8,6,6,2,6,6,3,7,8,2 | ENSMUSG00000109022,"ENSMUSG00000109022",0,0,0,0,0,0,0,0,0,0,0,0,0,0,0,0,0,0,0,0 |
| ENSMUSG00000029260,"ENSMUSG00000029260",0,0,0,0,0,0,0,3,0,0,0,0,0,0,0,0,0,0,0,0 | ENSMUSG00000106743,"ENSMUSG00000106743",14,17,22,26,12,20,5,15,12,9,13,22,11,11,7,14,20,13,9,11 | ENSMUSG00000109328,"ENSMUSG00000109328",0,0,0,0,0,0,0,0,0,0,0,0,0,0,0,0,0,0,0,0 |
| ENSMUSG00000027423,"ENSMUSG00000027423",12691,8956,13250,12060,12876,12253,9889,9871,9867,11873,12202,8152,11348,11144,10367,11246,11148,11722,8702,10555 | ENSMUSG00000093989,"ENSMUSG00000093989",1,7,19,13,4,12,12,9,9,9,11,6,9,9,7,6,7,7,15,9 | ENSMUSG00000037603,"ENSMUSG00000037603",0,0,0,0,0,0,0,0,0,0,0,0,0,0,0,0,0,0,0,0 |
| ENSMUSG00000024335,"ENSMUSG00000024335",3540,6494,7871,6753,6694,7014,7184,6208,6853,7543,7428,5757,6942,6900,6504,7972,6374,8750,5663,6924 | ENSMUSG00000091017,"ENSMUSG00000091017",15,22,29,8,20,42,41,77,34,37,30,8,40,22,24,53,29,67,37,77 | ENSMUSG00000088212,"ENSMUSG00000088212",0,0,0,0,2,0,0,0,0,0,0,0,0,0,0,0,0,0,0,0 |
| ENSMUSG00000063511,"ENSMUSG00000063511",1805,4232,5758,5647,4645,4398,4497,3965,4265,4563,5903,4375,5086,5101,4626,5348,4340,5894,5051,4844 | ENSMUSG00000105283,"ENSMUSG00000105283",22,17,24,15,25,23,12,19,26,25,18,14,20,11,19,24,20,37,11,12 | ENSMUSG00000096646,"ENSMUSG00000096646",0,0,0,0,0,0,0,0,0,0,0,0,0,0,0,0,0,0,0,0 |
| ENSMUSG00000071659,"ENSMUSG00000071659",2488,3692,4934,4816,4411,4023,4038,3407,3737,4159,5068,3914,4370,4366,4095,4511,3993,5196,3910,4037 | ENSMUSG00000106820,"ENSMUSG00000106820",14,13,24,15,21,15,10,10,11,9,10,11,17,15,8,18,4,13,15,27 | ENSMUSG00000095500,"ENSMUSG00000095500",0,0,0,0,0,0,0,0,0,0,0,0,0,0,0,0,0,0,0,0 |
| ENSMUSG00000062908,"ENSMUSG00000062908",632,786,1141,959,982,831,847,715,811,948,1017,779,1115,903,773,885,797,1012,823,843 | ENSMUSG00000070717,"ENSMUSG00000070717",1,5,2,9,5,3,3,1,10,3,8,4,2,6,4,8,3,6,2,9 | ENSMUSG00000096730,"ENSMUSG00000096730",0,0,1,0,0,0,0,0,0,0,0,1,0,0,2,0,0,0,0,0 |
| ENSMUSG00000035247,"ENSMUSG00000035247",5787,5928,6665,6636,6991,6119,6470,5264,5953,6943,6733,5462,6464,6630,6028,7059,6119,7383,4244,5832 | ENSMUSG00000097474,"ENSMUSG00000097474",2,6,4,4,0,2,7,1,7,7,2,1,0,4,1,3,3,5,6,11 | ENSMUSG00000102851,"ENSMUSG00000102851",0,0,0,0,0,0,0,0,0,0,0,0,0,0,0,0,0,0,0,0 |
| ENSMUSG00000024646,"ENSMUSG00000024646",723,828,1150,1098,991,836,939,773,855,1016,1116,913,1106,924,963,932,836,1161,883,980 | ENSMUSG00000098291,"ENSMUSG00000098291",8,4,5,2,3,3,5,12,2,16,4,1,9,5,11,3,4,7,1,7 | ENSMUSG00000103147,"ENSMUSG00000103147",0,0,0,1,0,0,0,0,0,0,0,0,0,0,0,0,0,0,0,0 |
| ENSMUSG00000052681,"ENSMUSG00000052681",8683,7570,9224,8058,9154,8892,7477,6745,7542,9159,7869,5897,7321,8268,7161,8239,7985,9186,5347,7520 | ENSMUSG00000062783,"ENSMUSG00000062783",8,4,10,7,10,4,2,15,4,9,3,2,19,8,9,20,9,6,8,5 | ENSMUSG00000102343,"ENSMUSG00000102343",0,0,0,0,0,0,0,0,0,0,0,0,0,0,0,0,0,0,0,0 |
| ENSMUSG00000020841,"ENSMUSG00000020841",5647,4776,5471,5096,5859,5019,5496,3844,5189,5038,5129,4558,4889,4846,4393,5140,4571,5816,3469,4581 | ENSMUSG00000107216,"ENSMUSG00000107216",8,4,8,8,6,7,5,4,2,3,9,4,16,9,6,7,13,9,4,4 | ENSMUSG00000103265,"ENSMUSG00000103265",0,0,0,0,0,0,0,0,0,0,0,0,0,0,0,0,0,0,0,0 |
| ENSMUSG00000028745,"ENSMUSG00000028745",3316,4525,6379,5455,5364,5498,4871,4838,4807,5738,5667,3990,5824,5501,5055,6284,4873,6543,4862,5627 | ENSMUSG00000063556,"ENSMUSG00000063556",1,10,8,16,10,10,5,6,13,14,6,11,4,8,8,11,8,11,6,7 | ENSMUSG00000100648,"ENSMUSG00000100648",0,0,0,0,0,0,0,0,0,0,0,0,1,0,0,0,0,0,0,0 |
| ENSMUSG00000017781,"ENSMUSG00000017781",5393,6678,9807,8587,8693,8982,7285,5964,6568,8438,7860,6007,7527,8171,7158,7943,7514,8529,6476,7146 | ENSMUSG00000111246,"ENSMUSG00000111246",0,4,6,9,9,11,7,2,9,9,4,11,4,14,7,13,5,16,4,2 | ENSMUSG00000101171,"ENSMUSG00000101171",0,0,0,0,0,0,0,0,0,0,0,0,0,0,0,0,0,0,0,0 |
| ENSMUSG00000024758,"ENSMUSG00000024758",2891,2282,3052,2659,2784,2740,2630,2447,2557,2525,2823,2025,2916,2466,2479,2656,2551,3052,2403,2826 | ENSMUSG00000092438,"ENSMUSG00000092438",6,9,8,10,3,7,9,2,5,4,5,8,9,5,4,6,4,10,4,8 | ENSMUSG00000104027,"ENSMUSG00000104027",0,0,0,0,0,0,0,0,0,0,0,0,0,0,0,0,0,0,0,0 |
| ENSMUSG00000020349,"ENSMUSG00000020349",6807,5563,6853,7119,6697,5943,6229,4799,5820,7003,6400,5649,5645,6345,5958,6706,5788,7263,5282,5851 | ENSMUSG00000028020,"ENSMUSG00000028020",4,1,6,6,1,1,6,3,7,1,10,1,12,6,2,9,5,7,5,6 | ENSMUSG00000102747,"ENSMUSG00000102747",0,0,0,0,0,0,0,0,0,0,0,0,0,0,0,0,0,0,0,0 |
| ENSMUSG00000045817,"ENSMUSG00000045817",4064,6826,8075,8582,7851,7893,6224,7476,8183,7673,7711,6105,8521,7785,7011,8053,7058,8668,5627,7577 | ENSMUSG00000112654,"ENSMUSG00000112654",11,5,11,23,10,9,1,7,11,10,13,7,3,14,5,10,17,17,3,7 | ENSMUSG00000117091,"ENSMUSG00000117091",0,0,0,0,0,0,0,0,0,0,0,0,0,0,0,0,0,0,0,0 |
| ENSMUSG00000029713,"ENSMUSG00000029713",2701,4783,6560,5576,5871,5545,5218,4912,4724,5647,5878,4249,5974,5602,5318,5955,5038,6642,4971,5433 | ENSMUSG00000032595,"ENSMUSG00000032595",5,5,10,3,5,6,8,5,4,5,6,6,13,8,7,9,7,7,9,2 | ENSMUSG00000103482,"ENSMUSG00000103482",0,0,0,0,0,0,0,0,0,0,0,0,0,0,0,0,0,0,0,0 |
| ENSMUSG00000000078,"ENSMUSG00000000078",7582,7704,6549,7738,7126,6551,7354,6722,9405,8835,5655,5351,5264,8777,6234,7097,7007,6451,3764,7128 | ENSMUSG00000025644,"ENSMUSG00000025644",3,15,15,18,8,6,7,9,7,12,17,9,10,12,13,11,19,21,16,9 | ENSMUSG00000084506,"ENSMUSG00000084506",0,0,0,0,0,0,0,0,0,0,0,0,0,0,0,0,0,0,0,0 |
| ENSMUSG00000037062,"ENSMUSG00000037062",4066,4399,5246,5324,5607,4851,4587,3714,4616,5316,4740,4009,4496,4755,4291,5095,4557,5488,3288,4372 | ENSMUSG00000036964,"ENSMUSG00000036964",0,9,6,0,1,3,1,1,0,4,3,1,3,0,5,1,2,8,2,5 | ENSMUSG00000045815,"ENSMUSG00000045815",0,0,0,0,0,0,0,0,0,0,0,0,0,0,0,0,0,0,0,0 |
| ENSMUSG00000071650,"ENSMUSG00000071650",2044,2353,3323,3370,3010,2872,3039,2614,2699,2611,3233,2341,2986,2858,2776,2922,2586,3317,2687,2938 | ENSMUSG00000113318,"ENSMUSG00000113318",6,15,2,2,6,4,4,2,7,5,3,11,6,4,2,3,6,3,3,7 | ENSMUSG00000101714,"ENSMUSG00000101714",0,0,0,0,0,0,0,0,0,0,0,0,0,0,0,0,0,0,0,0 |
| ENSMUSG00000060147,"ENSMUSG00000060147",3907,3724,4699,4605,4152,3480,3932,2814,3634,4425,4290,3701,3725,3689,3515,4246,3429,4507,3036,4033 | ENSMUSG00000090381,"ENSMUSG00000090381",4,4,10,10,5,6,9,5,6,6,7,4,7,7,6,13,3,22,5,13 | ENSMUSG00000109510,"ENSMUSG00000109510",0,1,0,0,0,0,0,0,0,0,0,0,0,0,0,0,0,0,0,0 |
| ENSMUSG00000019173,"ENSMUSG00000019173",5050,8316,10805,11323,10263,9437,9091,8624,9071,9658,10481,8531,10015,10633,9362,11530,9215,11582,10050,9227 | ENSMUSG00000077734,"ENSMUSG00000077734",9,8,11,11,19,5,20,2,7,3,13,11,16,8,7,17,6,14,10,9 | ENSMUSG00000099784,"ENSMUSG00000099784",0,0,0,0,0,0,0,0,0,0,0,0,0,0,0,0,0,0,0,0 |
| ENSMUSG00000052837,"ENSMUSG00000052837",5263,15557,17387,13157,17599,18363,18136,18349,19399,17417,12797,11291,17404,17565,15447,20128,15057,20337,13395,19954 | ENSMUSG00000091754,"ENSMUSG00000091754",13,10,13,18,4,11,11,11,20,7,11,6,7,8,11,15,9,13,12,10 | ENSMUSG00000100434,"ENSMUSG00000100434",0,0,0,0,0,0,1,0,1,0,0,0,0,0,0,0,0,0,0,0 |
| ENSMUSG00000027750,"ENSMUSG00000027750",11082,12458,17752,16946,16223,21750,14899,12520,14254,13570,16823,12037,14384,13798,12100,12704,13287,14302,9918,9865 | ENSMUSG00000101641,"ENSMUSG00000101641",0,0,2,1,0,0,2,1,0,1,5,1,3,0,0,1,0,1,0,1 | ENSMUSG00000100679,"ENSMUSG00000100679",0,0,0,2,0,0,0,0,0,0,0,0,0,0,0,0,0,0,0,0 |
| ENSMUSG00000029135,"ENSMUSG00000029135",8419,11681,11835,10849,12352,12222,12244,10656,11377,13750,10714,8612,11531,14467,11622,13683,11287,13001,8202,11332 | ENSMUSG00000106751,"ENSMUSG00000106751",18,11,15,11,12,9,7,7,10,17,12,19,5,20,11,12,12,3,9,11 | ENSMUSG00000092657,"ENSMUSG00000092657",0,0,1,0,0,0,0,0,0,0,0,0,0,0,0,0,0,0,2,0 |
| ENSMUSG00000023092,"ENSMUSG00000023092",5643,3632,3780,4128,4571,1360,4665,2893,3875,5453,3362,3215,4220,3626,3393,3169,4461,3649,3340,3548 | ENSMUSG00000019894,"ENSMUSG00000019894",7,3,4,0,0,3,0,0,5,3,4,4,0,0,2,7,2,1,2,1 | ENSMUSG00000082661,"ENSMUSG00000082661",0,0,0,0,0,0,0,0,0,0,0,0,0,0,0,0,0,0,0,0 |
| ENSMUSG00000022186,"ENSMUSG00000022186",2218,1833,2630,2551,2355,1984,1889,2013,1880,2188,2581,1688,2405,2024,2085,2243,1978,2455,1997,2088 | ENSMUSG00000085573,"ENSMUSG00000085573",3,11,1,18,3,3,10,1,9,13,5,3,5,12,4,10,6,6,3,2 | ENSMUSG00000086730,"ENSMUSG00000086730",0,0,0,0,0,0,0,0,0,0,0,0,0,0,0,0,0,0,1,0 |
| ENSMUSG00000040940,"ENSMUSG00000040940",2923,4253,5777,4761,4983,5104,4798,3972,4420,4947,4991,3864,4745,5093,4466,5322,4422,6166,3878,4703 | ENSMUSG00000056412,"ENSMUSG00000056412",14,10,4,3,10,8,9,5,8,10,4,5,11,5,8,10,4,7,4,8 | ENSMUSG00000067299,"ENSMUSG00000067299",0,0,0,0,0,0,0,0,0,0,0,0,0,0,0,0,0,0,0,0 |
| ENSMUSG00000029811,"ENSMUSG00000029811",0,1,1,0,0,1,1,2,1,0,1,0,0,2,1,0,0,1,0,2 | ENSMUSG00000090336,"ENSMUSG00000090336",0,0,0,2,0,0,0,0,0,0,0,0,0,0,0,0,0,0,0,0 | ENSMUSG00000085812,"ENSMUSG00000085812",0,0,0,0,0,0,0,0,0,0,0,0,0,0,0,0,1,0,0,0 |
| ENSMUSG00000037254,"ENSMUSG00000037254",13,19,19,27,20,12,10,20,14,28,15,7,17,20,20,18,12,19,17,7 | ENSMUSG00000086236,"ENSMUSG00000086236",9,6,3,8,11,12,4,4,3,12,11,7,12,1,6,11,9,12,6,7 | ENSMUSG00000100429,"ENSMUSG00000100429",0,0,1,0,0,0,0,0,0,0,0,0,0,0,0,0,2,0,0,0 |
| ENSMUSG00000040584,"ENSMUSG00000040584",67,84,99,63,105,86,69,128,84,98,132,69,161,100,95,116,96,148,109,110 | ENSMUSG00000039963,"ENSMUSG00000039963",0,6,1,5,1,1,2,5,4,2,5,5,3,4,1,1,2,6,2,9 | ENSMUSG00000100556,"ENSMUSG00000100556",0,0,0,0,0,0,0,0,0,0,0,0,0,0,0,0,0,0,0,0 |
| ENSMUSG00000026365,"ENSMUSG00000026365",2665,2115,2986,2511,2804,1738,1931,1996,2158,2395,2573,1580,2592,2318,2155,2215,2227,2650,1850,2012 | ENSMUSG00000021647,"ENSMUSG00000021647",0,0,1,0,0,0,0,0,0,0,0,0,0,0,0,1,1,0,0,0 | ENSMUSG00000093782,"ENSMUSG00000093782",0,0,0,0,0,2,0,0,0,0,0,0,0,1,0,0,0,0,0,0 |
| ENSMUSG00000036880,"ENSMUSG00000036880",843,825,1119,1006,949,876,904,806,794,948,1124,790,948,893,709,1027,769,1158,831,822 | ENSMUSG00000085636,"ENSMUSG00000085636",3,4,16,8,10,11,7,6,8,7,9,12,4,10,5,8,2,8,3,2 | ENSMUSG00000104396,"ENSMUSG00000104396",0,0,0,0,0,0,0,0,0,0,0,0,0,0,0,0,0,0,0,0 |
| ENSMUSG00000020091,"ENSMUSG00000020091",1793,1967,2676,2396,2348,2191,2064,1769,1990,2191,2803,2031,2508,2111,1920,2133,1944,2487,1987,1961 | ENSMUSG00000097899,"ENSMUSG00000097899",9,46,31,50,31,38,25,41,32,45,27,18,39,64,32,38,29,28,25,41 | ENSMUSG00000102481,"ENSMUSG00000102481",0,1,0,0,0,0,0,0,0,0,0,1,0,0,0,0,0,0,0,0 |
| ENSMUSG00000022797,"ENSMUSG00000022797",2111,1719,2124,2133,2313,1633,1844,1533,1817,2045,2505,2173,1628,1789,1714,1682,1794,1815,1548,1659 | ENSMUSG00000097573,"ENSMUSG00000097573",13,7,16,14,4,10,9,9,6,7,6,11,9,2,5,16,9,16,8,7 | ENSMUSG00000101328,"ENSMUSG00000101328",0,0,0,0,0,0,0,0,0,0,0,0,0,0,0,0,0,0,1,0 |
| ENSMUSG00000001025,"ENSMUSG00000001025",5868,7608,10446,7244,8079,8632,7542,5749,6627,8328,7910,6618,8396,7669,7298,9350,7362,10584,6815,9785 | ENSMUSG00000109205,"ENSMUSG00000109205",0,0,3,3,2,0,0,2,2,1,6,0,4,5,1,1,4,6,5,1 | ENSMUSG00000102956,"ENSMUSG00000102956",0,0,0,0,0,0,0,0,0,0,0,0,0,0,0,0,0,0,0,0 |
| ENSMUSG00000030654,"ENSMUSG00000030654",3315,2661,3833,4307,3395,3121,2859,2870,2788,3068,3511,2577,3405,3237,2938,3636,2866,4085,2973,3280 | ENSMUSG00000115025,"ENSMUSG00000115025",18,9,15,18,14,20,16,15,19,11,19,4,12,20,17,19,6,22,6,9 | ENSMUSG00000099553,"ENSMUSG00000099553",0,1,0,0,0,0,1,0,0,0,0,0,0,0,0,0,0,0,0,0 |
| ENSMUSG00000005514,"ENSMUSG00000005514",2350,3576,4589,3491,3994,4414,4106,3814,3561,4269,3738,2767,4564,4411,3973,4551,3803,5071,3043,4101 | ENSMUSG00000082064,"ENSMUSG00000082064",3,9,5,17,9,4,2,4,5,14,16,6,7,5,7,18,9,12,4,8 | ENSMUSG00000100850,"ENSMUSG00000100850",0,0,0,0,0,0,0,0,0,0,0,0,0,0,0,0,0,0,0,0 |
| ENSMUSG00000040659,"ENSMUSG00000040659",6721,8922,11541,10297,11111,10165,9873,8783,9258,11247,9303,7442,10427,11597,10008,11461,10532,12922,7952,10246 | ENSMUSG00000109237,"ENSMUSG00000109237",1,7,7,7,5,2,2,9,4,3,2,5,3,2,3,13,4,6,3,6 | ENSMUSG00000067064,"ENSMUSG00000067064",0,0,0,0,0,0,3,0,0,0,0,0,0,0,0,0,0,0,0,0 |
| ENSMUSG00000004789,"ENSMUSG00000004789",2303,2770,3409,3320,3228,3020,3310,2424,2801,3135,3129,2628,2713,3048,2795,3034,2745,3358,2701,2854 | ENSMUSG00000082180,"ENSMUSG00000082180",3,5,9,10,4,7,11,5,9,6,17,4,6,6,7,4,3,9,1,9 | ENSMUSG00000086161,"ENSMUSG00000086161",0,0,0,0,0,2,0,0,0,0,0,0,0,0,0,0,0,0,1,0 |
| ENSMUSG00000030824,"ENSMUSG00000030824",1513,2722,3875,3312,3409,3534,3074,2793,2796,3350,3436,2367,3676,3357,2977,3493,2770,3798,2788,3199 | ENSMUSG00000086794,"ENSMUSG00000086794",1,5,3,5,11,6,5,2,0,8,4,8,4,4,2,3,6,0,3,9 | ENSMUSG00000100041,"ENSMUSG00000100041",0,0,0,0,0,0,0,0,1,1,0,0,0,0,0,0,0,0,0,0 |
| ENSMUSG00000040385,"ENSMUSG00000040385",2330,3918,5820,5009,4985,4686,4774,4174,4245,4505,5137,3821,5186,4740,4708,5258,4275,6344,4913,5180 | ENSMUSG00000095889,"ENSMUSG00000095889",0,0,0,0,0,0,0,0,0,0,0,0,0,0,0,0,0,0,0,0 | ENSMUSG00000099870,"ENSMUSG00000099870",0,0,0,0,0,0,0,0,0,0,0,0,0,0,0,0,0,0,0,0 |
| ENSMUSG00000009291,"ENSMUSG00000009291",3285,3159,4016,3912,3931,3657,3347,2904,3405,3823,3332,2749,3315,3829,3055,3757,3291,3868,2693,3355 | ENSMUSG00000072381,"ENSMUSG00000072381",0,0,0,1,1,0,0,0,0,1,0,2,1,0,1,0,0,0,0,0 | ENSMUSG00000099860,"ENSMUSG00000099860",0,0,0,0,0,0,0,2,0,0,0,0,1,0,0,0,0,0,0,0 |
| ENSMUSG00000024789,"ENSMUSG00000024789",14773,13001,15127,13723,15405,15105,13113,9457,12590,16699,11646,9401,10585,15223,12020,13117,13737,12685,7495,10985 | ENSMUSG00000027977,"ENSMUSG00000027977",1,9,4,1,3,10,8,6,8,8,1,4,7,5,7,2,13,12,9,5 | ENSMUSG00000101179,"ENSMUSG00000101179",0,0,0,0,0,0,0,0,0,0,0,0,0,0,0,0,1,0,0,0 |
| ENSMUSG00000018340,"ENSMUSG00000018340",2005,2655,4589,4247,3757,3620,3236,3720,3245,3684,4286,2262,4791,4005,3974,3507,3725,4055,3357,3953 | ENSMUSG00000107350,"ENSMUSG00000107350",17,3,6,16,10,10,4,5,8,16,7,6,6,16,2,15,13,9,3,6 | ENSMUSG00000101275,"ENSMUSG00000101275",0,0,0,0,0,0,0,0,0,0,0,0,0,0,0,0,0,0,0,0 |
| ENSMUSG00000063015,"ENSMUSG00000063015",1962,1928,2447,1866,2361,2397,1938,1787,1878,2460,1900,1685,2235,2134,1970,2316,2142,2334,1422,2091 | ENSMUSG00000096398,"ENSMUSG00000096398",0,0,0,0,0,0,0,0,0,0,0,0,0,0,0,0,0,0,0,0 | ENSMUSG00000077846,"ENSMUSG00000077846",0,0,0,0,0,0,0,0,0,0,0,0,0,0,0,0,0,0,0,0 |
| ENSMUSG00000021486,"ENSMUSG00000021486",4443,4068,6391,5243,5899,5362,4837,5009,4534,4793,5123,4097,5712,4903,4882,5965,4762,6839,5046,5808 | ENSMUSG00000026226,"ENSMUSG00000026226",0,0,0,0,0,1,0,2,0,0,2,0,1,0,0,0,0,0,0,0 | ENSMUSG00000088164,"ENSMUSG00000088164",0,0,0,0,0,0,0,0,0,0,0,0,0,0,0,0,0,0,0,0 |
| ENSMUSG00000066406,"ENSMUSG00000066406",4087,4969,5657,5136,5944,6070,5390,4580,5107,6416,5402,3735,5871,6094,4930,5755,5172,6212,3533,5044 | ENSMUSG00000109559,"ENSMUSG00000109559",0,2,1,1,0,2,0,6,2,1,0,1,3,2,1,6,0,2,0,4 | ENSMUSG00000102822,"ENSMUSG00000102822",0,0,0,0,0,0,0,0,0,0,0,0,0,0,0,0,0,0,0,0 |
| ENSMUSG00000061353,"ENSMUSG00000061353",1498,2135,3294,1876,2358,3957,2345,2517,2361,2536,2999,1601,3349,2170,2133,2846,2403,4231,2202,3200 | ENSMUSG00000087095,"ENSMUSG00000087095",14,18,27,19,21,15,21,22,20,31,30,16,37,24,15,21,24,17,19,24 | ENSMUSG00000099808,"ENSMUSG00000099808",0,0,0,0,0,0,0,0,2,0,0,0,0,0,0,0,0,0,0,0 |
| ENSMUSG00000022836,"ENSMUSG00000022836",272,313,459,504,464,450,357,370,328,389,533,283,469,412,405,376,424,448,389,317 | ENSMUSG00000103114,"ENSMUSG00000103114",0,1,0,0,2,0,0,1,2,2,0,1,1,1,8,2,0,4,1,2 | ENSMUSG00000100595,"ENSMUSG00000100595",0,0,0,0,0,0,0,0,0,0,0,0,0,0,0,0,0,0,0,0 |
| ENSMUSG00000025473,"ENSMUSG00000025473",12287,19949,22380,19639,21238,21030,20800,20400,20265,25763,18215,14450,21949,28145,20596,24871,22258,23090,14752,22580 | ENSMUSG00000049176,"ENSMUSG00000049176",1,1,0,0,0,0,2,1,1,0,2,0,0,0,0,0,0,0,1,0 | ENSMUSG00000103840,"ENSMUSG00000103840",0,0,0,0,0,0,0,0,0,0,0,0,0,0,0,0,0,0,0,0 |
| ENSMUSG00000030530,"ENSMUSG00000030530",7470,14051,14184,14116,15856,13850,14469,9562,12985,16309,11112,11119,11140,15832,11752,15973,12847,15347,8331,11661 | ENSMUSG00000107254,"ENSMUSG00000107254",6,10,3,12,7,12,15,12,8,15,8,9,9,20,14,17,12,13,5,5 | ENSMUSG00000099933,"ENSMUSG00000099933",0,0,0,0,0,0,0,0,0,0,0,0,0,0,0,0,0,0,1,0 |
| ENSMUSG00000059898,"ENSMUSG00000059898",27919,22450,23003,30214,25707,18492,23879,15105,22587,21623,25555,26526,16480,21157,19999,22536,18841,21876,14500,17344 | ENSMUSG00000070424,"ENSMUSG00000070424",14,19,25,32,24,16,29,20,20,28,20,15,17,21,30,26,23,32,12,35 | ENSMUSG00000103333,"ENSMUSG00000103333",0,0,0,1,0,0,0,0,0,0,0,0,2,0,0,0,0,0,0,0 |
| ENSMUSG00000022462,"ENSMUSG00000022462",9162,6995,7523,6444,6707,7276,7513,5823,7218,7637,7597,5797,7032,6396,6596,7597,6120,7862,4754,6310 | ENSMUSG00000018844,"ENSMUSG00000018844",6,6,8,15,5,5,9,7,7,4,10,3,3,13,11,11,5,8,2,3 | ENSMUSG00000098379,"ENSMUSG00000098379",0,0,0,0,0,3,0,0,0,0,0,0,0,0,0,0,0,0,0,0 |
| ENSMUSG00000021238,"ENSMUSG00000021238",313,254,311,289,318,249,286,210,294,301,330,197,284,280,215,252,261,300,233,288 | ENSMUSG00000043036,"ENSMUSG00000043036",27,17,25,29,18,17,30,16,40,36,41,31,24,19,18,25,23,28,26,21 | ENSMUSG00000065573,"ENSMUSG00000065573",0,0,0,0,0,0,0,0,0,1,0,0,1,0,0,0,0,0,0,0 |
| ENSMUSG00000034634,"ENSMUSG00000034634",9257,18196,20494,21974,23976,15929,19758,14798,19303,22008,19064,18991,16525,18944,19856,25650,17516,25428,17723,20273 | ENSMUSG00000099098,"ENSMUSG00000099098",0,2,6,4,0,5,2,0,2,4,5,0,6,4,3,5,3,2,5,3 | ENSMUSG00000099303,"ENSMUSG00000099303",0,0,0,0,0,0,0,0,0,0,0,0,0,0,0,0,0,2,0,0 |
| ENSMUSG00000019877,"ENSMUSG00000019877",4668,3419,4418,4023,4432,3965,3672,3116,3555,4265,3866,2886,3411,3783,3267,3796,3739,4175,2602,3350 | ENSMUSG00000113571,"ENSMUSG00000113571",7,7,9,8,14,6,5,8,15,9,12,4,12,15,3,7,10,2,8,8 | ENSMUSG00000104202,"ENSMUSG00000104202",0,0,0,0,0,0,0,0,0,0,0,0,0,0,0,0,0,0,0,0 |
| ENSMUSG00000028435,"ENSMUSG00000028435",23700,23030,22542,33422,26526,20429,31172,13339,24533,18842,21107,27231,10839,23093,18341,24147,16636,24400,16157,15653 | ENSMUSG00000098037,"ENSMUSG00000098037",2,0,0,1,1,0,0,1,0,1,1,0,0,0,0,1,0,0,0,0 | ENSMUSG00000090533,"ENSMUSG00000090533",0,0,0,0,0,0,0,0,0,0,0,0,0,0,0,0,0,0,0,0 |
| ENSMUSG00000027506,"ENSMUSG00000027506",5270,5410,6260,5684,6339,6322,5923,4914,5478,6820,5482,4353,5731,6338,5330,6766,5405,7016,4080,5770 | ENSMUSG00000104346,"ENSMUSG00000104346",7,5,17,8,11,6,13,5,5,8,10,12,9,9,5,9,10,7,7,7 | ENSMUSG00000103942,"ENSMUSG00000103942",0,0,0,0,0,0,0,0,0,1,0,0,0,0,0,0,0,0,0,0 |

| ENSMUSG00000068874,"ENSMUSG00000068874",75,102,228,141,169,164,117,264,158,97,172,134,311,124,153,239,157,251,172,248 | ENSMUSG00000091507,"ENSMUSG00000091507",13,8,6,10,12,12,7,6,9,9,4,1,6,14,8,7,9,12,6,12 | ENSMUSG00000103449,"ENSMUSG00000103449",0,0,0,0,0,0,0,0,0,0,0,0,0,0,0,0,0,0,0,0 |
| --- | --- | --- |
| ENSMUSG00000037487,"ENSMUSG00000037487",6127,5558,5938,6574,6336,5485,6186,4359,5454,6223,5903,5105,5021,5705,5250,5802,5400,6349,4012,4812 | ENSMUSG00000106777,"ENSMUSG00000106777",11,12,13,26,23,24,31,15,20,18,31,9,14,22,20,28,12,21,25,15 | ENSMUSG00000104341,"ENSMUSG00000104341",0,0,0,0,0,0,1,0,0,0,0,0,0,0,0,0,0,0,0,0 |
| ENSMUSG00000053898,"ENSMUSG00000053898",626,891,1252,1133,1041,986,969,945,969,1045,1129,817,1264,985,944,1190,1016,1244,1042,1046 | ENSMUSG00000105868,"ENSMUSG00000105868",16,4,7,12,13,5,1,5,4,9,14,4,7,8,3,9,5,8,6,7 | ENSMUSG00000089158,"ENSMUSG00000089158",0,0,0,0,0,0,0,0,0,0,0,0,0,0,0,0,0,0,0,0 |
| ENSMUSG00000063696,"ENSMUSG00000063696",6740,10750,13510,14224,11000,10232,11388,8194,10068,10990,12880,10734,10339,11633,10316,12781,9799,12670,10174,9833 | ENSMUSG00000017588,"ENSMUSG00000017588",1,27,16,10,0,472,20,4,7,0,2,1,23,0,10,31,0,8,13,87 | ENSMUSG00000092633,"ENSMUSG00000092633",0,0,0,0,0,0,1,0,0,0,0,1,0,0,0,0,0,0,0,0 |
| ENSMUSG00000004098,"ENSMUSG00000004098",576,839,1638,943,912,1883,1173,1028,885,1172,1217,696,1577,1114,995,904,1034,1120,672,1030 | ENSMUSG00000020622,"ENSMUSG00000020622",3,1,1,2,1,0,3,0,3,2,1,2,3,3,6,1,1,2,0,0 | ENSMUSG00000086921,"ENSMUSG00000086921",0,0,0,0,0,0,0,0,0,0,0,0,0,0,0,0,0,0,0,0 |
| ENSMUSG00000005417,"ENSMUSG00000005417",3519,4415,5285,5907,5061,4597,4937,3897,4622,4714,5105,4508,4570,4794,4738,5398,4408,5834,4011,4709 | ENSMUSG00000026182,"ENSMUSG00000026182",2,0,0,0,0,0,0,0,0,0,0,0,0,0,0,0,0,0,0,0 | ENSMUSG00000082332,"ENSMUSG00000082332",0,0,1,0,0,0,0,0,0,0,0,0,0,0,2,0,0,0,0,0 |
| ENSMUSG00000028186,"ENSMUSG00000028186",1558,1794,1690,1714,2919,1574,2038,2226,1826,1988,1678,1838,1875,2096,2556,3101,2052,3246,2097,2730 | ENSMUSG00000053353,"ENSMUSG00000053353",0,6,2,4,2,1,1,3,5,0,2,3,0,2,1,2,0,9,6,2 | ENSMUSG00000104734,"ENSMUSG00000104734",0,0,0,0,2,0,0,0,0,0,0,0,0,0,0,0,0,0,0,0 |
| ENSMUSG00000033335,"ENSMUSG00000033335",2114,3982,5198,4848,5041,4893,4340,4271,4351,4767,4530,3633,5177,5100,4493,5158,4337,5767,4039,4727 | ENSMUSG00000103441,"ENSMUSG00000103441",0,2,0,2,0,2,1,0,0,4,0,2,0,3,0,0,0,1,0,0 | ENSMUSG00000081008,"ENSMUSG00000081008",0,0,0,0,0,0,0,0,0,0,0,0,0,0,0,0,0,0,0,0 |
| ENSMUSG00000012705,"ENSMUSG00000012705",32,42,36,25,40,30,37,30,47,71,41,20,68,43,42,43,45,57,42,32 | ENSMUSG00000085940,"ENSMUSG00000085940",0,0,0,0,0,0,0,0,0,0,0,0,0,0,0,0,0,0,0,0 | ENSMUSG00000086418,"ENSMUSG00000086418",0,0,0,0,0,0,0,0,0,0,0,0,0,0,0,0,0,0,0,0 |
| ENSMUSG00000031751,"ENSMUSG00000031751",2799,2645,3316,2978,3065,2934,2786,2089,2494,2997,2988,2302,2638,2787,2445,2798,2492,3176,1951,2396 | ENSMUSG00000076480,"ENSMUSG00000076480",5,5,6,6,6,2,7,3,8,5,7,8,13,6,3,7,4,5,9,7 | ENSMUSG00000082864,"ENSMUSG00000082864",0,0,0,0,0,0,0,0,0,0,0,0,0,0,0,0,0,0,0,0 |
| ENSMUSG00000028793,"ENSMUSG00000028793",8414,15174,17404,17446,16915,15817,16570,12892,16490,19451,12855,12394,13016,20077,15336,19106,16478,18887,11592,16252 | ENSMUSG00000020090,"ENSMUSG00000020090",0,0,2,1,3,0,2,1,0,0,0,0,0,0,1,1,0,0,2,3 | ENSMUSG00000080538,"ENSMUSG00000080538",0,0,0,0,0,0,0,0,0,0,0,0,0,0,0,0,0,0,0,0 |
| ENSMUSG00000067786,"ENSMUSG00000067786",118,197,227,198,237,201,227,138,216,209,156,167,175,206,202,187,212,235,183,206 | ENSMUSG00000097410,"ENSMUSG00000097410",12,19,19,17,14,17,18,13,12,17,19,20,4,10,7,9,20,6,6,7 | ENSMUSG00000086519,"ENSMUSG00000086519",0,0,0,0,0,0,0,0,0,0,0,0,0,0,0,0,0,0,0,0 |
| ENSMUSG00000052974,"ENSMUSG00000052974",89,117,123,177,141,207,116,95,139,143,116,131,125,142,139,104,104,149,149,193 | ENSMUSG00000093124,"ENSMUSG00000093124",12,5,9,9,14,11,9,7,16,11,15,7,9,8,7,8,13,18,2,7 | ENSMUSG00000105312,"ENSMUSG00000105312",0,0,0,0,0,0,0,0,0,0,0,0,0,0,0,0,0,0,0,0 |
| ENSMUSG00000032324,"ENSMUSG00000032324",6004,4149,5715,5278,5540,4274,5651,4047,5091,5117,5211,4362,4639,4560,5138,5393,4857,5708,4075,5031 | ENSMUSG00000084381,"ENSMUSG00000084381",10,11,13,15,6,12,9,11,13,12,6,15,12,10,9,10,20,5,8,3 | ENSMUSG00000065423,"ENSMUSG00000065423",0,0,0,0,0,0,0,0,0,0,0,0,0,0,0,0,0,0,0,0 |
| ENSMUSG00000029106,"ENSMUSG00000029106",1488,2142,3002,2748,2561,2616,2508,2558,2382,2584,2858,2101,3046,2651,2385,2638,2406,3148,2302,2789 | ENSMUSG00000092241,"ENSMUSG00000092241",0,8,10,5,4,5,5,5,5,10,3,9,1,4,4,9,5,5,8,10 | ENSMUSG00000088868,"ENSMUSG00000088868",0,0,0,0,0,0,3,0,0,0,0,0,0,0,0,0,0,0,0,0 |
| ENSMUSG00000070047,"ENSMUSG00000070047",1228,1635,2012,2110,1831,1831,2289,1697,2179,1676,2030,1688,1801,1745,1527,1806,1720,2086,1561,1892 | ENSMUSG00000097668,"ENSMUSG00000097668",0,0,0,3,0,1,0,0,0,0,2,0,0,2,0,0,1,1,0,1 | ENSMUSG00000086349,"ENSMUSG00000086349",0,2,0,1,0,0,0,0,0,0,0,0,0,0,0,0,0,0,0,0 |
| ENSMUSG00000009079,"ENSMUSG00000009079",2622,4487,5677,5988,5083,4713,4752,4116,4643,4955,5980,4435,5003,5323,4674,5407,4400,5844,4735,4826 | ENSMUSG00000067242,"ENSMUSG00000067242",6,10,6,10,15,8,5,8,5,12,6,3,5,10,3,11,5,7,3,7 | ENSMUSG00000086523,"ENSMUSG00000086523",0,0,0,0,1,0,0,0,0,0,0,0,0,0,0,0,1,0,0,0 |
| ENSMUSG00000020869,"ENSMUSG00000020869",4585,4407,5452,5648,5289,5051,5293,3962,4344,5077,5224,4111,4240,5107,4615,4986,4450,5483,4160,4494 | ENSMUSG00000085711,"ENSMUSG00000085711",0,1,6,1,1,3,6,2,3,0,4,1,2,1,2,4,1,2,1,2 | ENSMUSG00000083472,"ENSMUSG00000083472",0,0,0,0,0,0,0,0,0,0,0,0,0,0,0,0,0,0,0,0 |
| ENSMUSG00000019428,"ENSMUSG00000019428",2250,2786,4214,3862,3477,3168,3018,3098,2867,3268,3733,2715,4095,3495,3135,3908,3071,4318,3431,3795 | ENSMUSG00000024211,"ENSMUSG00000024211",0,1,1,4,2,2,1,1,3,3,2,0,12,1,3,4,3,5,4,3 | ENSMUSG00000089272,"ENSMUSG00000089272",0,0,0,0,0,0,0,0,0,0,0,0,0,0,0,0,0,0,0,0 |
| ENSMUSG00000030681,"ENSMUSG00000030681",2778,3794,5339,6045,4854,4326,4670,4208,4168,4714,4662,3666,4621,5538,4641,5648,4284,5885,4098,4814 | ENSMUSG00000112547,"ENSMUSG00000112547",8,7,5,4,3,6,10,1,3,8,7,4,1,4,2,1,6,6,3,6 | ENSMUSG00000097837,"ENSMUSG00000097837",0,0,0,0,0,0,0,0,0,0,0,0,0,0,0,0,0,0,0,0 |
| ENSMUSG00000008668,"ENSMUSG00000008668",3194,4237,4886,4826,4010,4021,4141,3019,4019,4337,4682,3944,3788,4032,3774,4888,3776,4877,5483,4265 | ENSMUSG00000113088,"ENSMUSG00000113088",0,3,0,0,0,1,0,1,0,1,0,1,1,1,1,0,0,0,0,2 | ENSMUSG00000084250,"ENSMUSG00000084250",0,0,0,0,0,0,0,0,0,0,0,0,0,0,0,0,0,0,0,0 |
| ENSMUSG00000004558,"ENSMUSG00000004558",1200,1272,1637,1772,2015,1064,1602,1634,1593,1726,1845,1407,2122,1545,1661,1905,1616,2249,1724,2026 | ENSMUSG00000084033,"ENSMUSG00000084033",11,13,16,18,13,9,6,11,11,5,14,9,9,9,8,10,8,11,10,8 | ENSMUSG00000085386,"ENSMUSG00000085386",0,0,0,0,0,0,0,0,0,0,0,0,0,0,0,0,0,0,0,0 |
| ENSMUSG00000024960,"ENSMUSG00000024960",1256,1832,2445,2431,2261,2011,2188,1849,1980,1814,2423,1838,2258,2169,2044,2367,1767,2650,2111,2134 | ENSMUSG00000084088,"ENSMUSG00000084088",3,0,1,5,3,2,4,0,3,1,2,1,1,2,1,9,7,8,3,0 | ENSMUSG00000087102,"ENSMUSG00000087102",0,0,0,0,0,0,0,0,0,0,0,0,0,0,0,0,0,2,1,0 |
| ENSMUSG00000029767,"ENSMUSG00000029767",5087,5313,7593,6513,6712,7454,6326,5300,5447,6278,6689,4724,6906,5896,5782,6168,5809,7026,4537,5979 | ENSMUSG00000071113,"ENSMUSG00000071113",21,11,24,43,24,27,13,31,17,10,25,19,29,27,14,34,13,36,17,19 | ENSMUSG00000075214,"ENSMUSG00000075214",2,0,0,0,0,0,0,0,0,0,0,0,0,0,0,0,0,1,0,0 |
| ENSMUSG00000021210,"ENSMUSG00000021210",0,0,0,0,0,0,1,2,2,0,0,0,0,0,0,0,0,0,2,6 | ENSMUSG00000082193,"ENSMUSG00000082193",8,2,10,10,8,8,3,4,11,11,6,5,6,3,7,12,11,5,0,7 | ENSMUSG00000047149,"ENSMUSG00000047149",0,0,0,0,0,0,0,0,0,0,0,0,0,0,0,0,0,0,0,0 |
| ENSMUSG00000055447,"ENSMUSG00000055447",4897,4521,5951,4999,5361,5776,4348,4232,4728,6657,5042,3844,5220,5782,4723,6132,4700,6106,3421,4719 | ENSMUSG00000086606,"ENSMUSG00000086606",2,11,16,19,6,7,10,15,14,5,18,7,6,7,7,12,10,17,14,12 | ENSMUSG00000110819,"ENSMUSG00000110819",0,0,0,0,0,0,0,0,0,0,0,0,0,0,0,0,0,0,0,0 |
| ENSMUSG00000016921,"ENSMUSG00000016921",4489,3896,4609,4756,4344,4273,4420,3104,3960,4126,4938,4111,3562,4016,3912,4316,3815,4597,3078,3714 | ENSMUSG00000107647,"ENSMUSG00000107647",7,4,0,7,5,7,3,8,8,3,6,4,6,4,4,3,1,7,1,10 | ENSMUSG00000075063,"ENSMUSG00000075063",0,0,0,0,0,0,0,0,0,0,0,0,0,0,0,0,0,0,0,0 |
| ENSMUSG00000044285,"ENSMUSG00000044285",27,41,167,178,115,124,152,53,72,104,67,47,163,181,50,77,90,68,296,71 | ENSMUSG00000097530,"ENSMUSG00000097530",4,7,13,5,13,12,10,7,12,5,5,1,8,9,5,8,11,17,5,7 | ENSMUSG00000098406,"ENSMUSG00000098406",0,0,0,0,0,0,0,0,0,0,0,0,0,0,0,0,0,0,0,0 |
| ENSMUSG00000042688,"ENSMUSG00000042688",11509,9589,10779,11253,11689,9727,10580,7555,10300,10964,8857,9682,7523,10105,8996,11767,8874,11619,6705,9112 | ENSMUSG00000014725,"ENSMUSG00000014725",0,0,0,0,0,0,0,0,0,0,0,0,1,0,0,0,0,1,0,0 | ENSMUSG00000086980,"ENSMUSG00000086980",0,0,0,0,0,0,0,0,0,0,0,0,0,0,0,0,0,1,0,0 |
| ENSMUSG00000020097,"ENSMUSG00000020097",5560,5564,7108,6484,6525,6087,5547,5006,5550,6510,6397,4702,6742,6468,5432,6184,5872,6907,4317,5505 | ENSMUSG00000104094,"ENSMUSG00000104094",13,33,19,24,36,22,23,28,38,17,23,39,20,22,24,27,22,19,7,21 | ENSMUSG00000082435,"ENSMUSG00000082435",0,0,0,0,0,0,0,0,0,0,0,0,0,0,0,0,0,0,0,0 |
| ENSMUSG00000045954,"ENSMUSG00000045954",154,127,183,152,196,131,144,202,154,210,178,138,276,136,156,157,227,239,172,233 | ENSMUSG00000117069,"ENSMUSG00000117069",0,1,11,3,12,11,4,3,2,3,3,3,1,1,12,10,8,5,1,4 | ENSMUSG00000081774,"ENSMUSG00000081774",0,0,0,0,0,0,0,0,0,0,0,0,0,0,0,0,0,0,0,0 |
| ENSMUSG00000026208,"ENSMUSG00000026208",1670,1993,2999,2852,2955,1070,2463,2328,2296,3182,2169,1709,3344,2677,2451,2621,3011,3209,2470,2969 | ENSMUSG00000103804,"ENSMUSG00000103804",8,11,14,5,12,14,5,6,7,3,15,1,1,1,3,2,4,3,2,2 | ENSMUSG00000087473,"ENSMUSG00000087473",0,0,0,2,0,0,0,0,0,0,0,0,0,0,0,1,0,0,0,0 |
| ENSMUSG00000030104,"ENSMUSG00000030104",3122,2739,3543,3429,3597,3212,2863,2782,2769,3205,3327,2560,3573,3211,3029,3702,3028,3986,2264,3124 | ENSMUSG00000048304,"ENSMUSG00000048304",0,0,0,0,1,0,1,2,0,0,0,0,0,1,0,0,0,0,0,0 | ENSMUSG00000109071,"ENSMUSG00000109071",0,0,0,0,0,0,0,0,0,0,0,0,0,0,0,0,0,0,0,0 |
| ENSMUSG00000034781,"ENSMUSG00000034781",683,765,1160,1054,1029,962,1008,763,870,912,1153,853,1060,853,938,1029,913,1209,882,948 | ENSMUSG00000112481,"ENSMUSG00000112481",11,2,20,21,16,9,10,4,10,18,16,5,8,17,13,5,6,14,12,6 | ENSMUSG00000081037,"ENSMUSG00000081037",0,0,1,0,0,0,0,0,0,0,1,1,0,0,0,0,0,0,0,0 |
| ENSMUSG00000025351,"ENSMUSG00000025351",6204,6008,9612,7181,7842,7993,7075,7229,6930,8068,7944,5012,9016,7827,7115,7636,7045,8668,6740,7865 | ENSMUSG00000086796,"ENSMUSG00000086796",5,5,2,9,12,8,9,9,8,4,12,10,6,11,10,6,7,10,8,11 | ENSMUSG00000092201,"ENSMUSG00000092201",0,0,0,0,0,0,0,0,0,0,0,0,0,0,0,0,0,0,0,0 |
| ENSMUSG00000028961,"ENSMUSG00000028961",1759,2352,3245,2850,2708,2690,2614,2722,2510,2555,2923,2148,3159,2663,2534,3099,2458,3517,2929,3123 | ENSMUSG00000115249,"ENSMUSG00000115249",8,5,11,3,9,5,4,1,3,5,3,3,6,5,2,8,2,3,1,2 | ENSMUSG00000081842,"ENSMUSG00000081842",0,0,0,0,0,0,0,0,0,0,0,0,0,0,0,0,0,0,0,0 |
| ENSMUSG00000025950,"ENSMUSG00000025950",603,694,1026,766,890,851,644,824,786,749,915,690,1175,719,646,837,759,978,722,875 | ENSMUSG00000105004,"ENSMUSG00000105004",2,8,1,2,2,3,2,3,1,1,5,1,3,2,0,1,0,6,0,1 | ENSMUSG00000087767,"ENSMUSG00000087767",0,0,0,0,0,0,0,0,0,0,0,0,0,0,0,0,0,0,0,0 |
| ENSMUSG00000020745,"ENSMUSG00000020745",4702,4300,4601,4783,4647,4220,4403,3447,4168,5140,4775,3780,4243,4140,4056,4505,4214,4943,3239,4112 | ENSMUSG00000038115,"ENSMUSG00000038115",2,3,0,1,1,4,1,3,2,3,6,2,5,1,5,2,2,0,1,1 | ENSMUSG00000074872,"ENSMUSG00000074872",0,0,0,0,0,0,0,0,0,0,0,0,0,1,0,0,0,0,0,0 |
| ENSMUSG00000027580,"ENSMUSG00000027580",3709,4300,4878,5675,4786,5426,5626,4885,4904,5694,4516,3063,5158,6158,5185,6940,5092,7288,4004,5166 | ENSMUSG00000092867,"ENSMUSG00000092867",11,14,5,4,11,8,5,11,21,13,7,3,8,18,6,11,23,10,0,10 | ENSMUSG00000065050,"ENSMUSG00000065050",0,0,0,0,0,1,0,0,0,0,0,0,0,0,0,0,0,0,0,0 |
| ENSMUSG00000020538,"ENSMUSG00000020538",1484,1966,2336,2492,2434,2005,2106,1725,2130,1972,2515,2176,2420,2198,1940,2269,1979,2493,2013,2182 | ENSMUSG00000073375,"ENSMUSG00000073375",8,23,29,43,34,2,20,44,52,45,48,55,61,27,49,34,45,27,56,50 | ENSMUSG00000083689,"ENSMUSG00000083689",0,0,0,0,0,0,0,0,0,0,0,0,0,0,0,0,0,0,0,0 |
| ENSMUSG00000007097,"ENSMUSG00000007097",991,1062,1326,1459,1504,494,1338,1141,1221,1526,1406,861,1588,1357,1270,1393,1377,1844,1480,1439 | ENSMUSG00000078680,"ENSMUSG00000078680",0,0,0,0,0,0,0,0,0,0,0,0,0,0,0,0,0,0,0,0 | ENSMUSG00000093338,"ENSMUSG00000093338",0,0,0,0,0,0,0,0,0,0,0,0,0,0,0,0,0,0,0,0 |
| ENSMUSG00000029823,"ENSMUSG00000029823",4337,3994,4448,4758,4771,4415,3724,3397,4104,4728,5108,3559,4112,4196,3935,4448,4142,4984,3050,3792 | ENSMUSG00000081497,"ENSMUSG00000081497",3,3,3,0,4,2,0,0,1,2,3,1,1,5,2,1,0,3,2,2 | ENSMUSG00000027444,"ENSMUSG00000027444",0,0,0,0,0,0,0,0,0,0,0,0,0,0,0,0,0,0,0,0 |
| ENSMUSG00000041459,"ENSMUSG00000041459",4317,3674,5012,4721,4671,4354,4665,3397,3825,4369,5452,3916,4053,4289,3911,4422,3834,5047,3775,3706 | ENSMUSG00000064441,"ENSMUSG00000064441",1,3,6,1,5,4,3,3,5,3,1,0,1,10,3,15,5,1,6,5 | ENSMUSG00000063507,"ENSMUSG00000063507",0,0,0,0,0,0,0,0,0,0,0,0,0,0,0,0,0,0,0,0 |
| ENSMUSG00000000001,"ENSMUSG00000000001",6916,6270,7313,7595,7666,6723,6902,4917,6232,7203,7040,6051,5662,6635,5890,6875,6135,7261,4716,5415 | ENSMUSG00000110542,"ENSMUSG00000110542",52,34,38,44,32,24,42,17,39,43,47,40,33,45,35,39,36,47,27,31 | ENSMUSG00000074739,"ENSMUSG00000074739",0,0,0,0,0,0,0,0,0,0,0,0,0,0,0,0,0,0,0,0 |
| ENSMUSG00000025792,"ENSMUSG00000025792",267,351,628,339,471,457,405,399,417,419,418,340,625,371,385,466,375,544,473,621 | ENSMUSG00000116164,"ENSMUSG00000116164",4,2,3,8,6,12,6,5,8,3,6,9,11,0,1,10,1,8,5,5 | ENSMUSG00000087337,"ENSMUSG00000087337",0,0,0,0,0,0,0,0,0,0,0,0,0,0,0,0,0,0,0,0 |
| ENSMUSG00000000326,"ENSMUSG00000000326",603,839,1155,1088,1013,1037,899,800,801,994,1001,792,1008,914,820,989,859,1028,902,976 | ENSMUSG00000091020,"ENSMUSG00000091020",6,17,16,10,7,7,10,8,15,25,21,6,11,15,10,16,12,9,2,9 | ENSMUSG00000087609,"ENSMUSG00000087609",0,0,0,0,0,0,0,0,0,0,0,0,0,0,0,0,0,0,0,0 |
| ENSMUSG00000059208,"ENSMUSG00000059208",2943,4028,5846,5537,5018,4880,4728,3933,4199,4309,4996,3824,4665,4694,4543,5171,4218,5988,4163,4989 | ENSMUSG00000113880,"ENSMUSG00000113880",6,24,14,21,9,21,14,8,10,21,14,9,13,17,12,21,14,7,8,12 | ENSMUSG00000085946,"ENSMUSG00000085946",0,0,0,0,0,0,0,0,0,0,0,0,0,0,0,0,0,0,0,0 |
| ENSMUSG00000030062,"ENSMUSG00000030062",2925,3792,5177,5114,4675,4414,4620,3896,3894,4224,5276,3873,4454,4674,4386,4628,3942,5066,3730,4551 | ENSMUSG00000029206,"ENSMUSG00000029206",5,0,5,4,3,3,7,3,2,4,9,5,4,3,2,3,2,4,7,4 | ENSMUSG00000087536,"ENSMUSG00000087536",0,0,0,0,0,0,0,0,0,0,1,0,0,0,0,0,0,0,0,0 |
| ENSMUSG00000025207,"ENSMUSG00000025207",50,82,131,219,197,106,90,71,131,103,265,203,197,129,97,168,76,150,159,108 | ENSMUSG00000107061,"ENSMUSG00000107061",0,0,0,0,9,1,1,0,0,0,0,0,0,0,0,4,0,0,0,0 | ENSMUSG00000085350,"ENSMUSG00000085350",0,0,0,0,0,0,0,0,0,0,0,0,0,0,0,0,0,0,0,0 |
| ENSMUSG00000022136,"ENSMUSG00000022136",2948,2694,3680,3864,3693,3479,3196,2892,3077,3209,3897,2583,3508,3353,3251,3531,3250,3627,2342,3042 | ENSMUSG00000101823,"ENSMUSG00000101823",3,0,0,0,0,2,0,0,0,0,0,2,2,0,1,0,0,0,0,1 | ENSMUSG00000038537,"ENSMUSG00000038537",0,0,0,0,0,0,0,0,0,0,0,0,0,0,0,0,0,0,0,0 |
| ENSMUSG00000018042,"ENSMUSG00000018042",1678,2459,3447,3033,3087,2983,2788,2283,2484,2882,2782,2263,3161,2788,2435,2939,2588,3504,2506,2902 | ENSMUSG00000055003,"ENSMUSG00000055003",14,21,22,36,32,17,21,15,27,23,30,32,19,30,28,20,19,29,18,17 | ENSMUSG00000086367,"ENSMUSG00000086367",0,0,0,0,0,0,0,0,0,0,0,0,0,0,0,0,0,0,0,0 |
| ENSMUSG00000020009,"ENSMUSG00000020009",2319,2801,3251,3242,3102,2648,2498,2305,2709,3341,3325,2578,2948,3320,2577,3275,2652,3194,2163,2613 | ENSMUSG00000091177,"ENSMUSG00000091177",0,14,3,12,4,11,1,6,11,5,7,3,5,5,8,8,12,6,4,3 | ENSMUSG00000078881,"ENSMUSG00000078881",0,0,0,0,0,0,0,0,0,1,0,0,0,0,0,0,0,0,0,0 |
| ENSMUSG00000071561,"ENSMUSG00000071561",18782,17805,21314,13900,24062,20945,23882,21393,20856,21760,18312,15991,19773,17252,20640,23145,18076,28753,24229,23510 | ENSMUSG00000099998,"ENSMUSG00000099998",13,8,17,10,25,17,17,8,14,14,7,7,9,9,7,16,6,17,10,10 | ENSMUSG00000078876,"ENSMUSG00000078876",0,0,0,0,0,0,0,0,0,0,0,0,0,0,0,0,0,0,0,0 |
| ENSMUSG00000024621,"ENSMUSG00000024621",5594,6326,9946,7601,8859,7800,5972,7564,6825,7755,7742,5161,9780,8139,6977,8664,7875,9185,7032,8195 | ENSMUSG00000097784,"ENSMUSG00000097784",7,3,14,15,11,11,11,7,5,7,13,7,12,8,1,10,7,14,8,9 | ENSMUSG00000081083,"ENSMUSG00000081083",0,0,0,0,0,0,0,0,0,0,0,0,1,0,0,0,0,0,0,0 |
| ENSMUSG00000046804,"ENSMUSG00000046804",0,0,0,0,1,0,0,0,0,1,0,0,0,0,0,0,0,0,0,0 | ENSMUSG00000097104,"ENSMUSG00000097104",52,27,63,74,46,26,34,36,33,24,46,35,29,29,29,35,43,48,25,36 | ENSMUSG00000045797,"ENSMUSG00000045797",0,0,0,0,0,0,0,0,0,0,0,0,0,0,0,0,0,0,0,0 |
| ENSMUSG00000015846,"ENSMUSG00000015846",1769,2686,3596,3970,3329,2982,2944,2721,2817,2811,3816,3005,3461,3051,2995,3483,2818,3873,2841,2853 | ENSMUSG00000036030,"ENSMUSG00000036030",16,5,9,4,10,9,7,8,6,7,8,4,12,6,14,15,6,19,7,10 | ENSMUSG00000101819,"ENSMUSG00000101819",0,0,0,0,0,0,0,0,0,0,0,0,0,0,0,0,0,0,0,0 |
| ENSMUSG00000032437,"ENSMUSG00000032437",3118,2850,3910,3915,3798,3244,3241,2597,3017,3279,3576,2963,3246,3297,2863,3296,3170,3491,2258,2963 | ENSMUSG00000086058,"ENSMUSG00000086058",4,4,8,2,2,3,5,5,4,4,1,5,3,7,4,6,4,8,4,7 | ENSMUSG00000083439,"ENSMUSG00000083439",0,0,0,0,0,0,0,0,0,0,0,0,0,0,0,0,0,0,0,0 |
| ENSMUSG00000028011,"ENSMUSG00000028011",2,12,13,13,9,2,7,5,12,6,15,13,15,5,13,5,9,7,3,7 | ENSMUSG00000054418,"ENSMUSG00000054418",5,5,7,4,1,8,3,5,12,4,14,7,7,5,10,4,6,6,6,11 | ENSMUSG00000093014,"ENSMUSG00000093014",0,0,0,0,0,0,0,0,0,0,0,0,0,0,0,0,0,0,0,0 |
| ENSMUSG00000066306,"ENSMUSG00000066306",1366,2317,3197,3264,2775,2701,2566,2562,2491,2677,3135,2079,3278,2812,2550,3310,2539,3584,2519,2880 | ENSMUSG00000047511,"ENSMUSG00000047511",21,16,13,35,33,35,29,28,21,33,46,26,30,31,28,43,41,42,23,26 | ENSMUSG00000086048,"ENSMUSG00000086048",0,0,0,0,0,0,0,0,0,0,0,0,0,0,0,0,0,0,0,0 |
| ENSMUSG00000044533,"ENSMUSG00000044533",2768,5019,7181,6587,5576,5159,5704,4618,5176,5904,6474,5318,5467,5981,5436,6847,5213,7221,5720,6277 | ENSMUSG00000031233,"ENSMUSG00000031233",0,0,0,0,0,0,0,0,0,0,0,0,0,0,0,0,0,0,0,0 | ENSMUSG00000062814,"ENSMUSG00000062814",0,0,0,0,0,0,0,0,0,0,0,0,0,0,0,0,0,0,0,0 |
| ENSMUSG00000024292,"ENSMUSG00000024292",0,0,1,0,2,0,0,0,0,1,1,0,1,0,0,1,0,0,1,1 | ENSMUSG00000107118,"ENSMUSG00000107118",6,7,6,4,8,3,6,2,4,9,3,3,3,8,8,3,7,0,4,0 | ENSMUSG00000081812,"ENSMUSG00000081812",0,0,0,0,0,0,0,0,0,0,0,0,0,0,0,0,0,0,0,0 |
| ENSMUSG00000026547,"ENSMUSG00000026547",2932,5021,6331,5974,5604,5692,5529,4398,4926,6155,5194,4333,5125,5586,4819,5725,4784,6867,4884,5322 | ENSMUSG00000111923,"ENSMUSG00000111923",0,0,0,3,0,0,0,0,0,0,0,0,1,0,0,0,0,1,0,1 | ENSMUSG00000084063,"ENSMUSG00000084063",0,0,0,0,0,0,0,0,0,0,0,0,0,0,0,0,0,0,0,0 |
| ENSMUSG00000026656,"ENSMUSG00000026656",15556,15605,22272,18634,20227,19030,15126,15050,15688,19815,16017,11965,17772,20286,16178,18904,17584,19339,14524,15804 | ENSMUSG00000114469,"ENSMUSG00000114469",0,0,0,1,0,1,2,1,0,0,0,1,1,0,1,2,0,1,0,0 | ENSMUSG00000086580,"ENSMUSG00000086580",0,0,0,0,0,0,3,0,0,0,0,0,0,0,0,0,0,0,0,0 |
| ENSMUSG00000030884,"ENSMUSG00000030884",2510,2031,2589,2778,2604,2128,2411,1904,2119,2363,2645,2206,2198,2274,2243,2412,2097,2538,2092,2141 | ENSMUSG00000100237,"ENSMUSG00000100237",0,0,0,0,0,0,0,0,0,0,1,0,0,0,0,0,2,0,0,0 | ENSMUSG00000099711,"ENSMUSG00000099711",0,0,0,0,0,0,0,0,0,0,0,0,0,0,0,0,0,0,0,0 |
| ENSMUSG00000030236,"ENSMUSG00000030236",3,0,2,0,0,0,1,0,3,0,1,0,0,0,0,0,0,0,0,2 | ENSMUSG00000085715,"ENSMUSG00000085715",2,1,0,0,0,0,1,0,0,1,0,0,0,0,0,10,0,1,0,1 | ENSMUSG00000081220,"ENSMUSG00000081220",0,0,0,0,0,0,0,0,0,0,0,0,0,0,0,0,0,0,0,0 |
| ENSMUSG00000037072,"ENSMUSG00000037072",4005,2925,4257,3400,3734,3637,2993,2763,3117,3501,3589,2703,3557,3232,3149,3484,3048,4101,2798,3473 | ENSMUSG00000077148,"ENSMUSG00000077148",10,5,0,14,4,7,2,3,2,5,6,16,2,5,4,5,8,13,11,5 | ENSMUSG00000094945,"ENSMUSG00000094945",0,1,0,0,0,0,0,0,0,0,1,0,0,0,0,0,0,0,0,1 |
| ENSMUSG00000021091,"ENSMUSG00000021091",4849,4332,6060,7391,6128,5924,4537,4058,5374,6838,5031,4135,5104,6526,4631,5967,4585,5687,4856,4887 | ENSMUSG00000047720,"ENSMUSG00000047720",2,2,4,1,6,6,1,6,4,3,1,1,6,4,4,9,3,6,2,12 | ENSMUSG00000095240,"ENSMUSG00000095240",0,0,0,0,0,0,0,0,0,0,0,0,0,0,0,0,0,0,0,0 |
| ENSMUSG00000061665,"ENSMUSG00000061665",3473,2726,2997,4172,3294,2724,2888,2328,2916,3009,3554,3329,2577,2826,2913,3214,2718,3308,2043,2516 | ENSMUSG00000066756,"ENSMUSG00000066756",45,20,57,29,56,19,28,29,19,25,21,21,35,34,57,40,48,27,29,41 | ENSMUSG00000081880,"ENSMUSG00000081880",0,0,0,0,0,0,0,0,0,0,0,0,0,0,0,0,0,0,0,0 |
| ENSMUSG00000035673,"ENSMUSG00000035673",8620,11718,12644,12903,13265,12569,13963,9535,12372,13661,11495,9927,10491,14159,11032,13738,10902,13665,9927,11061 | ENSMUSG00000075316,"ENSMUSG00000075316",1,2,8,6,2,1,1,1,3,0,1,2,3,1,1,1,2,3,3,4 | ENSMUSG00000083450,"ENSMUSG00000083450",0,3,0,0,0,0,0,0,0,0,0,0,0,0,0,0,0,0,0,0 |
| ENSMUSG00000022010,"ENSMUSG00000022010",1993,1564,2213,1581,2062,1649,1972,1861,1887,1954,1783,1235,2335,1991,1736,1986,1802,2165,1599,2230 | ENSMUSG00000021961,"ENSMUSG00000021961",25,20,53,46,28,23,33,39,21,29,37,40,33,28,26,39,12,59,44,21 | ENSMUSG00000083455,"ENSMUSG00000083455",0,0,0,0,0,0,0,0,0,0,0,0,0,0,0,0,0,0,0,0 |
| ENSMUSG00000035783,"ENSMUSG00000035783",417,606,778,855,743,930,701,756,717,889,774,422,985,795,730,837,758,860,822,898 | ENSMUSG00000073407,"ENSMUSG00000073407",23,14,16,23,11,14,11,11,13,10,33,11,25,15,25,16,18,20,24,8 | ENSMUSG00000082660,"ENSMUSG00000082660",0,0,0,0,0,0,0,0,0,0,0,0,0,0,0,0,0,0,0,0 |
| ENSMUSG00000021585,"ENSMUSG00000021585",7816,7372,8232,9971,9454,7199,8034,5918,7452,8386,8135,8101,6542,7603,7389,9404,7300,9599,5401,7395 | ENSMUSG00000039617,"ENSMUSG00000039617",6,7,6,4,12,9,10,10,6,8,10,4,4,5,9,10,9,5,2,6 | ENSMUSG00000031130,"ENSMUSG00000031130",0,0,0,0,0,0,0,0,0,0,0,0,0,0,0,0,0,0,0,0 |
| ENSMUSG00000041841,"ENSMUSG00000041841",3838,4198,5741,5523,4049,4076,4214,3634,4051,5053,5515,4487,4769,5257,4273,5257,4298,5744,4023,4738 | ENSMUSG00000038607,"ENSMUSG00000038607",9,5,11,8,8,7,10,4,5,4,4,6,3,2,9,6,4,6,6,4 | ENSMUSG00000105997,"ENSMUSG00000105997",0,0,0,0,0,0,0,0,0,0,0,0,0,0,0,0,0,0,0,0 |
| ENSMUSG00000005161,"ENSMUSG00000005161",1609,1625,2412,2179,1889,1814,1752,1618,1735,1660,2216,1606,2180,1884,1738,2212,1620,2481,1818,2123 | ENSMUSG00000087307,"ENSMUSG00000087307",6,4,0,2,2,5,4,1,3,8,4,0,3,3,5,5,2,2,1,0 | ENSMUSG00000086185,"ENSMUSG00000086185",0,0,0,0,0,0,0,0,0,0,0,0,0,0,0,0,0,0,0,0 |
| ENSMUSG00000057863,"ENSMUSG00000057863",1816,4995,6545,5677,4594,4910,4877,3615,3960,5158,5762,4580,5113,5143,4749,5723,4630,6815,5387,5532 | ENSMUSG00000045318,"ENSMUSG00000045318",0,0,1,0,0,2,0,5,3,0,1,1,3,1,0,0,1,0,2,4 | ENSMUSG00000081084,"ENSMUSG00000081084",0,0,0,0,0,0,0,0,0,0,0,0,0,0,0,0,0,0,0,0 |
| ENSMUSG00000003072,"ENSMUSG00000003072",1105,2488,3059,3033,2852,2724,2795,2469,2427,2847,3186,2478,2982,2799,2581,3415,2571,3277,2893,2927 | ENSMUSG00000076539,"ENSMUSG00000076539",0,0,0,0,0,0,0,0,0,1,0,0,0,0,0,0,0,0,3,0 | ENSMUSG00000077406,"ENSMUSG00000077406",0,0,0,0,0,0,0,0,0,0,0,0,0,0,0,0,0,0,0,0 |
| ENSMUSG00000032434,"ENSMUSG00000032434",7631,6747,8214,9660,8361,7365,7850,5723,7277,7674,6975,6898,6108,7919,6473,8156,6428,8589,5301,6516 | ENSMUSG00000084507,"ENSMUSG00000084507",15,26,11,16,10,15,18,16,23,16,13,9,13,14,12,12,12,10,7,14 | ENSMUSG00000086207,"ENSMUSG00000086207",0,0,0,0,0,0,0,0,0,0,0,0,0,0,0,0,0,0,0,0 |
| ENSMUSG00000002769,"ENSMUSG00000002769",8,14,12,24,15,15,9,9,22,16,20,25,30,8,8,9,11,22,14,18 | ENSMUSG00000025318,"ENSMUSG00000025318",4,0,3,2,5,4,3,3,4,2,4,3,4,4,4,2,2,6,5,4 | ENSMUSG00000080283,"ENSMUSG00000080283",0,0,0,0,0,0,0,0,0,0,0,0,0,0,0,0,0,0,0,0 |
| ENSMUSG00000031078,"ENSMUSG00000031078",2362,3778,4530,5044,4652,3651,4754,3546,4292,3943,4251,3843,3901,4573,3901,4773,3656,5142,3910,4420 | ENSMUSG00000094989,"ENSMUSG00000094989",10,4,6,13,5,11,8,8,14,14,6,10,4,15,7,8,8,10,4,15 | ENSMUSG00000082907,"ENSMUSG00000082907",0,0,1,0,0,0,0,0,0,0,0,0,0,0,0,0,0,0,0,0 |
| ENSMUSG00000017119,"ENSMUSG00000017119",1643,1718,2295,2138,2084,1874,1864,1831,1897,2030,2338,1621,2415,1872,1849,2295,1833,2205,1728,1855 | ENSMUSG00000107161,"ENSMUSG00000107161",2,3,7,6,16,6,7,9,3,2,3,11,2,6,5,5,19,21,10,5 | ENSMUSG00000080774,"ENSMUSG00000080774",0,0,0,0,0,0,0,0,0,0,0,0,0,0,0,1,0,0,0,0 |
| ENSMUSG00000090862,"ENSMUSG00000090862",4738,5090,6817,6491,5962,5469,5758,4364,4995,5440,6055,5105,5503,5793,5167,6097,5025,7101,4996,5670 | ENSMUSG00000108714,"ENSMUSG00000108714",0,3,4,5,7,10,4,1,2,4,2,6,6,0,2,11,8,4,6,11 | ENSMUSG00000082275,"ENSMUSG00000082275",0,0,0,0,0,0,0,0,0,0,0,0,0,1,0,0,0,0,0,0 |
| ENSMUSG00000032047,"ENSMUSG00000032047",967,1035,1266,1174,1256,1190,1132,948,1043,1079,1435,1078,1292,1068,1032,1142,983,1352,1081,1092 | ENSMUSG00000082079,"ENSMUSG00000082079",0,0,0,3,0,0,0,0,1,0,3,1,0,1,0,1,0,2,2,1 | ENSMUSG00000109156,"ENSMUSG00000109156",0,0,0,0,0,0,0,0,0,0,0,0,0,0,0,0,0,0,0,0 |
| ENSMUSG00000027907,"ENSMUSG00000027907",6300,8690,10166,9050,10139,9567,9729,7878,9097,10621,9033,8413,9044,10330,9177,12160,8733,12045,8241,9978 | ENSMUSG00000082383,"ENSMUSG00000082383",0,3,3,6,2,5,2,5,2,2,5,3,6,1,4,3,5,3,1,1 | ENSMUSG00000085536,"ENSMUSG00000085536",0,0,0,0,0,0,0,0,0,0,0,0,0,0,0,0,0,0,0,0 |
| ENSMUSG00000040152,"ENSMUSG00000040152",5049,5896,6560,6386,4144,6347,5463,7161,6453,7400,5494,3711,5331,7666,4785,5173,5046,6091,3741,6209 | ENSMUSG00000070522,"ENSMUSG00000070522",0,1,0,0,2,1,1,1,0,1,1,1,0,0,1,0,0,1,0,0 | ENSMUSG00000094620,"ENSMUSG00000094620",0,0,0,0,0,0,0,0,0,0,0,0,0,0,0,0,0,0,0,0 |
| ENSMUSG00000056692,"ENSMUSG00000056692",3120,4583,5512,6109,5234,4753,5237,4132,4735,5129,5278,4386,4614,5186,4766,5996,4766,6469,4278,4568 | ENSMUSG00000102817,"ENSMUSG00000102817",3,5,9,3,6,6,6,7,9,6,3,4,4,1,6,10,3,14,11,5 | ENSMUSG00000084337,"ENSMUSG00000084337",0,0,0,1,0,0,0,0,0,0,0,1,0,0,1,0,0,0,0,0 |
| ENSMUSG00000039529,"ENSMUSG00000039529",1497,1182,1339,1429,1419,1322,1414,1212,1372,1498,1410,1109,1140,1367,1388,1398,1298,1694,916,1306 | ENSMUSG00000074527,"ENSMUSG00000074527",1,1,5,1,7,0,1,2,2,1,4,2,5,2,5,1,1,4,0,1 | ENSMUSG00000083024,"ENSMUSG00000083024",0,0,0,0,2,0,0,0,0,0,0,0,0,0,0,0,0,0,0,0 |
| ENSMUSG00000015733,"ENSMUSG00000015733",8043,6077,6917,7026,7518,6942,5645,5205,6323,7579,6538,5021,5866,6783,5677,6861,6320,7591,4401,5706 | ENSMUSG00000108353,"ENSMUSG00000108353",6,6,11,5,15,11,4,10,5,12,26,7,13,12,1,17,12,15,10,12 | ENSMUSG00000054994,"ENSMUSG00000054994",0,0,0,0,0,0,0,0,0,0,0,0,0,1,0,0,0,0,0,0 |
| ENSMUSG00000028936,"ENSMUSG00000028936",3329,3997,5231,4727,4166,4109,4106,3490,3971,4762,5020,4146,4110,4187,3830,4603,3861,5120,3708,4461 | ENSMUSG00000111864,"ENSMUSG00000111864",0,2,0,1,3,3,2,0,1,0,0,1,3,1,1,0,0,1,2,4 | ENSMUSG00000059203,"ENSMUSG00000059203",0,0,0,0,0,0,0,0,0,0,0,0,0,0,0,0,0,0,0,0 |
| ENSMUSG00000033765,"ENSMUSG00000033765",13228,19998,20721,32255,19351,17387,17017,15551,19292,16641,28179,21933,21343,20264,16955,25472,14996,26101,17539,17690 | ENSMUSG00000074824,"ENSMUSG00000074824",2,1,2,2,1,6,5,1,0,2,0,3,4,2,4,4,2,5,3,3 | ENSMUSG00000086441,"ENSMUSG00000086441",0,0,0,0,0,0,0,0,0,0,1,0,0,0,0,0,0,1,0,0 |
| ENSMUSG00000029178,"ENSMUSG00000029178",5730,5607,5407,6506,6371,5169,5936,4636,6066,5764,5766,5397,5185,5208,4825,5981,4830,6209,4198,5455 | ENSMUSG00000093394,"ENSMUSG00000093394",3,0,0,1,3,0,1,0,2,0,1,2,1,4,1,4,0,0,2,8 | ENSMUSG00000081803,"ENSMUSG00000081803",0,0,0,0,0,0,0,0,0,0,0,0,0,0,0,0,0,0,0,0 |
| ENSMUSG00000060733,"ENSMUSG00000060733",2531,2850,2978,3821,3243,2456,3225,2138,2720,3260,3360,3017,2142,3210,3134,2946,2741,2990,2078,2496 | ENSMUSG00000116597,"ENSMUSG00000116597",13,28,46,10,23,28,19,20,20,31,36,42,31,25,28,35,19,60,12,41 | ENSMUSG00000095872,"ENSMUSG00000095872",0,0,0,0,0,0,0,1,0,0,0,0,0,0,0,0,0,0,0,0 |
| ENSMUSG00000030189,"ENSMUSG00000030189",3287,5488,6250,8109,5908,5290,6390,4298,5499,5907,7131,6310,5320,5948,5617,6088,5335,6488,4129,5421 | ENSMUSG00000114235,"ENSMUSG00000114235",11,7,6,11,12,5,2,12,8,8,10,9,11,5,6,9,7,11,8,4 | ENSMUSG00000082974,"ENSMUSG00000082974",0,0,0,0,0,0,0,0,0,0,0,0,0,0,0,0,0,0,0,0 |
| ENSMUSG00000002957,"ENSMUSG00000002957",5314,5370,8749,7011,7653,7760,5785,5478,5782,6564,7121,4500,6749,6836,6159,6902,6280,6881,4994,5945 | ENSMUSG00000104684,"ENSMUSG00000104684",26,22,23,13,15,11,23,16,13,27,8,14,20,15,14,25,20,21,17,18 | ENSMUSG00000083719,"ENSMUSG00000083719",0,0,0,0,0,0,0,0,0,0,0,2,0,0,0,0,1,0,0,0 |
| ENSMUSG00000021069,"ENSMUSG00000021069",843,1005,1352,1000,1161,1464,1085,1276,1000,1395,1171,701,1514,1465,1255,1388,1217,1448,797,1290 | ENSMUSG00000083649,"ENSMUSG00000083649",6,4,5,0,1,3,4,1,3,4,5,3,5,6,4,2,2,5,2,3 | ENSMUSG00000083362,"ENSMUSG00000083362",0,0,0,0,0,0,0,0,0,0,0,0,0,0,0,1,0,0,0,0 |
| ENSMUSG00000002289,"ENSMUSG00000002289",958,1200,1618,1335,991,2023,2060,1110,1028,1952,1313,844,697,1729,1325,1518,1132,1805,644,954 | ENSMUSG00000101660,"ENSMUSG00000101660",8,0,0,6,1,0,5,1,4,3,3,3,1,0,0,3,0,0,3,0 | ENSMUSG00000082026,"ENSMUSG00000082026",0,0,0,0,0,0,0,0,0,0,0,0,0,0,0,0,0,0,0,0 |
| ENSMUSG00000020953,"ENSMUSG00000020953",2088,1883,2194,2902,2203,2818,2043,1356,1906,2156,2350,1423,1423,2018,1550,1684,1687,1644,1475,1527 | ENSMUSG00000026416,"ENSMUSG00000026416",51,60,38,47,44,20,45,22,36,31,25,30,20,44,46,39,31,40,32,59 | ENSMUSG00000100010,"ENSMUSG00000100010",0,0,0,0,0,0,0,0,0,0,0,0,0,0,0,0,0,0,0,0 |
| ENSMUSG00000043131,"ENSMUSG00000043131",6325,4347,5476,5367,5290,5159,4676,3997,4562,4978,5407,4009,4817,4789,4369,4832,4465,5479,3682,4035 | ENSMUSG00000047696,"ENSMUSG00000047696",0,1,2,0,2,2,1,0,0,0,1,2,1,0,0,0,1,0,0,0 | ENSMUSG00000103256,"ENSMUSG00000103256",0,0,0,0,0,0,0,0,0,0,0,0,0,0,0,0,0,0,0,0 |
| ENSMUSG00000029188,"ENSMUSG00000029188",2053,2527,2995,4693,4585,2681,3176,4333,3282,2124,3791,3871,3914,3133,4151,4437,3183,4417,3917,4095 | ENSMUSG00000087371,"ENSMUSG00000087371",6,3,0,7,8,1,8,7,2,1,4,4,1,1,0,1,4,7,5,5 | ENSMUSG00000105009,"ENSMUSG00000105009",0,0,0,0,0,0,0,0,0,0,0,0,0,0,0,0,0,0,0,0 |
| ENSMUSG00000018379,"ENSMUSG00000018379",3419,3089,3623,3893,3775,3575,3494,2764,3085,3502,4009,3050,3158,3194,3092,3454,2999,3679,2989,2993 | ENSMUSG00000003872,"ENSMUSG00000003872",1,7,16,16,10,6,12,8,7,2,5,13,10,8,16,7,9,9,13,8 | ENSMUSG00000092695,"ENSMUSG00000092695",0,0,0,0,0,0,0,0,0,0,0,0,0,0,0,0,0,0,0,0 |
| ENSMUSG00000021196,"ENSMUSG00000021196",3381,4497,5992,6906,6631,5102,4919,5829,4812,4741,5806,5534,5957,5791,5423,6766,4918,7389,5074,5675 | ENSMUSG00000109222,"ENSMUSG00000109222",0,4,3,5,1,3,4,3,2,1,5,0,8,1,2,2,4,3,2,2 | ENSMUSG00000103992,"ENSMUSG00000103992",0,0,0,0,0,0,0,0,0,0,0,0,0,0,0,0,0,0,0,0 |
| ENSMUSG00000034707,"ENSMUSG00000034707",3139,3915,6471,4611,4752,4890,3762,4469,4204,4959,4998,3129,6195,4584,4399,5350,4439,6066,3549,4983 | ENSMUSG00000103270,"ENSMUSG00000103270",0,0,0,1,1,0,1,1,0,0,2,2,2,0,1,0,1,0,1,0 | ENSMUSG00000102498,"ENSMUSG00000102498",0,0,0,0,0,0,0,0,0,0,0,0,0,0,0,0,0,0,0,0 |
| ENSMUSG00000020053,"ENSMUSG00000020053",1962,1649,2548,1985,2333,1984,1399,1688,1517,2068,2099,1535,2137,1621,1668,1634,1925,1588,1254,1615 | ENSMUSG00000041700,"ENSMUSG00000041700",34,31,43,30,28,45,30,37,25,21,47,25,44,35,21,44,25,47,38,47 | ENSMUSG00000104901,"ENSMUSG00000104901",0,0,0,1,0,0,0,0,0,0,0,0,0,0,0,0,0,0,0,0 |
| ENSMUSG00000073940,"ENSMUSG00000073940",8,5,16,16,18,8,6,11,3,6,9,14,14,9,8,11,18,8,2,7 | ENSMUSG00000043633,"ENSMUSG00000043633",8,1,0,4,2,2,1,4,5,2,6,3,2,2,3,3,3,0,4,4 | ENSMUSG00000105284,"ENSMUSG00000105284",0,0,0,0,0,0,0,0,0,0,0,0,0,0,0,0,0,0,0,0 |
| ENSMUSG00000028906,"ENSMUSG00000028906",1186,1857,2191,2225,2147,1766,1746,1970,1800,1995,2303,1812,2215,1999,1936,2349,1796,2658,1789,2025 | ENSMUSG00000107126,"ENSMUSG00000107126",0,0,0,0,0,0,0,0,0,0,0,0,0,2,1,0,0,0,0,0 | ENSMUSG00000106496,"ENSMUSG00000106496",0,1,0,0,0,0,0,0,0,0,0,0,0,0,0,0,0,0,0,0 |
| ENSMUSG00000006740,"ENSMUSG00000006740",5801,4427,4760,5368,5205,4714,4609,3582,4410,4861,4781,4268,3720,4365,3900,4797,4021,5032,3076,3966 | ENSMUSG00000110237,"ENSMUSG00000110237",2,0,2,2,12,8,2,8,0,2,4,2,3,0,3,6,2,2,1,5 | ENSMUSG00000106140,"ENSMUSG00000106140",0,0,0,0,0,0,0,0,0,0,0,0,0,0,0,0,0,0,0,0 |
| ENSMUSG00000031957,"ENSMUSG00000031957",0,0,0,0,0,0,0,1,0,0,2,0,0,1,0,3,5,0,0,1 | ENSMUSG00000115925,"ENSMUSG00000115925",3,5,7,9,8,5,19,6,5,10,30,9,7,6,13,3,2,7,13,10 | ENSMUSG00000094007,"ENSMUSG00000094007",0,0,0,0,0,0,0,0,0,0,0,0,0,0,0,0,0,0,0,0 |
| ENSMUSG00000025993,"ENSMUSG00000025993",436,364,430,334,492,560,399,446,380,518,549,311,598,441,349,422,402,504,299,505 | ENSMUSG00000102697,"ENSMUSG00000102697",2,3,0,2,1,1,2,3,5,0,6,0,3,3,1,0,7,1,0,9 | ENSMUSG00000092986,"ENSMUSG00000092986",0,0,0,0,0,0,0,0,0,2,0,0,0,0,0,0,0,0,1,0 |
| ENSMUSG00000041870,"ENSMUSG00000041870",3127,4053,5683,4722,4725,4808,4409,3952,4112,4742,4887,3738,4980,4613,4481,5259,4333,5743,3681,4731 | ENSMUSG00000110235,"ENSMUSG00000110235",0,0,2,0,10,0,0,2,3,1,1,1,2,1,1,5,2,3,5,1 | ENSMUSG00000103947,"ENSMUSG00000103947",0,0,0,0,0,0,0,0,0,0,0,0,0,0,0,0,0,0,0,0 |
| ENSMUSG00000061232,"ENSMUSG00000061232",3872,1821,2537,2873,2284,1769,1928,2333,2417,2354,2833,2024,2786,2363,2242,2960,2340,3239,2034,2691 | ENSMUSG00000117011,"ENSMUSG00000117011",5,17,15,10,13,12,6,5,8,9,12,8,7,7,11,10,6,12,12,9 | ENSMUSG00000104511,"ENSMUSG00000104511",0,0,0,0,0,0,0,0,0,0,0,0,0,0,0,0,0,0,0,0 |
| ENSMUSG00000055748,"ENSMUSG00000055748",235,96,105,167,123,80,86,141,147,95,167,182,96,111,97,160,78,122,96,91 | ENSMUSG00000101795,"ENSMUSG00000101795",10,1,4,3,6,10,8,6,8,12,12,11,4,6,7,14,13,7,2,1 | ENSMUSG00000104134,"ENSMUSG00000104134",0,0,0,3,0,0,0,0,0,0,0,0,0,0,0,0,0,0,0,0 |
| ENSMUSG00000015243,"ENSMUSG00000015243",5065,4800,7404,4794,6446,5451,4329,4997,5036,6626,5540,3230,6761,5475,4996,6381,5003,6771,3689,5485 | ENSMUSG00000083019,"ENSMUSG00000083019",5,10,12,9,8,15,21,6,10,15,22,7,10,17,12,12,10,9,4,14 | ENSMUSG00000065070,"ENSMUSG00000065070",0,2,0,0,0,0,0,0,0,0,0,0,0,0,0,0,0,0,0,0 |
| ENSMUSG00000027712,"ENSMUSG00000027712",3834,3722,5003,4775,4424,4384,4514,3982,3997,4535,4719,3505,4662,4553,4435,4617,4216,5129,3710,4418 | ENSMUSG00000029307,"ENSMUSG00000029307",8,32,59,27,30,43,32,27,26,21,37,12,46,43,39,24,27,34,26,18 | ENSMUSG00000104471,"ENSMUSG00000104471",0,1,0,0,0,1,0,0,0,0,0,0,0,0,0,0,0,0,1,0 |
| ENSMUSG00000025934,"ENSMUSG00000025934",9,19,10,13,5,11,12,8,6,10,16,14,4,13,4,6,4,23,13,21 | ENSMUSG00000072553,"ENSMUSG00000072553",0,2,2,4,9,6,3,10,3,7,9,7,11,7,6,6,2,6,1,8 | ENSMUSG00000088360,"ENSMUSG00000088360",0,0,0,0,0,0,0,0,0,0,0,0,0,0,0,0,0,0,0,0 |
| ENSMUSG00000038467,"ENSMUSG00000038467",4246,6605,8744,8761,7502,7516,7312,6217,7037,8599,7374,6007,7598,8631,7515,9600,7403,10048,6686,7939 | ENSMUSG00000109198,"ENSMUSG00000109198",20,13,15,18,16,14,19,7,24,20,21,5,11,16,15,6,21,25,9,21 | ENSMUSG00000092049,"ENSMUSG00000092049",0,0,0,0,0,0,0,0,0,0,0,0,0,0,0,0,0,0,0,0 |
| ENSMUSG00000025270,"ENSMUSG00000025270",48,45,63,53,114,55,69,22,19,19,41,50,51,37,33,48,92,61,32,9 | ENSMUSG00000109337,"ENSMUSG00000109337",4,12,16,20,13,6,9,10,7,4,10,6,12,8,7,8,12,19,10,5 | ENSMUSG00000068999,"ENSMUSG00000068999",0,0,0,0,0,0,0,0,0,1,0,0,0,0,0,0,1,0,0,0 |
| ENSMUSG00000023022,"ENSMUSG00000023022",2542,3940,4225,5547,4003,3586,4562,3234,3872,3933,4022,4069,3075,4287,3850,4634,3424,4220,3407,3632 | ENSMUSG00000097193,"ENSMUSG00000097193",2,3,5,7,9,8,1,5,0,3,4,1,3,6,2,8,1,11,4,2 | ENSMUSG00000103641,"ENSMUSG00000103641",0,0,0,0,0,0,0,0,0,0,0,1,0,0,0,0,0,0,2,0 |
| ENSMUSG00000043733,"ENSMUSG00000043733",2876,3295,4142,4288,3866,3478,3829,2942,3330,3698,3903,3299,3526,3737,3468,3836,3336,4300,2913,3325 | ENSMUSG00000001558,"ENSMUSG00000001558",8,5,3,1,4,1,0,1,3,5,3,2,1,2,4,3,0,2,1,1 | ENSMUSG00000097968,"ENSMUSG00000097968",0,0,0,0,0,0,0,0,0,0,0,0,0,0,0,0,0,0,0,0 |
| ENSMUSG00000006498,"ENSMUSG00000006498",3236,4517,5454,5389,5336,5150,5243,4229,4748,4770,5297,4436,4651,5038,4530,5388,4188,5594,4451,5011 | ENSMUSG00000076710,"ENSMUSG00000076710",0,0,0,0,0,0,0,0,0,0,0,0,0,0,0,0,0,0,0,0 | ENSMUSG00000051777,"ENSMUSG00000051777",0,0,0,0,0,0,0,0,0,0,0,0,0,0,0,0,0,0,0,0 |
| ENSMUSG00000079037,"ENSMUSG00000079037",6568,8154,9610,12431,9821,7602,9124,6359,8511,9782,8938,8663,7182,10506,8030,9291,7904,9069,7259,7132 | ENSMUSG00000109780,"ENSMUSG00000109780",0,1,0,0,0,0,2,1,0,0,1,0,2,0,2,5,1,2,1,1 | ENSMUSG00000102422,"ENSMUSG00000102422",0,0,0,0,0,0,0,0,0,0,0,0,0,0,0,0,0,0,0,0 |
| ENSMUSG00000059447,"ENSMUSG00000059447",2201,1656,1888,1876,1995,1809,1961,1593,1655,1866,1987,1551,1835,1793,1664,2062,1729,2283,1497,1658 | ENSMUSG00000090141,"ENSMUSG00000090141",4,10,16,23,26,17,20,16,13,9,18,12,13,14,6,20,23,16,13,17 | ENSMUSG00000065580,"ENSMUSG00000065580",0,0,0,0,0,0,0,0,0,0,0,0,0,0,0,0,0,0,0,0 |
| ENSMUSG00000026123,"ENSMUSG00000026123",2677,2170,2855,2927,2984,2021,2505,2480,2405,2422,2673,2306,2669,2698,2571,2945,2475,3149,2401,2706 | ENSMUSG00000116624,"ENSMUSG00000116624",2,6,10,4,3,10,9,3,1,9,14,7,11,1,5,5,4,8,5,8 | ENSMUSG00000088794,"ENSMUSG00000088794",0,0,0,0,0,0,0,0,0,0,0,0,0,0,0,0,0,0,0,0 |
| ENSMUSG00000066366,"ENSMUSG00000066366",0,4,8,14,10,8,5,10,10,6,5,5,12,11,7,5,7,9,17,3 | ENSMUSG00000081176,"ENSMUSG00000081176",3,5,6,9,6,6,4,9,7,9,4,11,9,5,12,11,2,7,6,5 | ENSMUSG00000103564,"ENSMUSG00000103564",0,0,0,0,1,0,0,0,0,0,0,0,0,0,0,0,0,0,0,0 |
| ENSMUSG00000071172,"ENSMUSG00000071172",4828,4283,4959,5485,5116,4599,4975,3882,4614,4900,5154,4138,4146,4636,4013,4475,3923,5000,3590,4250 | ENSMUSG00000084803,"ENSMUSG00000084803",4,5,7,8,4,2,4,4,5,0,1,1,3,3,4,3,2,0,4,2 | ENSMUSG00000077461,"ENSMUSG00000077461",0,0,0,0,0,0,0,0,0,0,0,0,0,0,0,0,0,0,0,0 |
| ENSMUSG00000021948,"ENSMUSG00000021948",2991,4443,5497,4523,5039,4784,4687,4343,4730,5239,4545,3801,5510,5309,4400,5561,4792,5866,4312,5266 | ENSMUSG00000104214,"ENSMUSG00000104214",1,3,1,1,6,3,2,6,0,0,1,0,2,3,3,2,6,1,1,6 | ENSMUSG00000103695,"ENSMUSG00000103695",0,0,0,0,0,0,0,0,0,0,0,0,0,1,0,0,0,0,0,0 |
| ENSMUSG00000011034,"ENSMUSG00000011034",2285,2397,2061,3822,2975,1444,2787,1685,2640,1969,2932,3553,2017,2413,2341,2735,2025,3014,2018,2236 | ENSMUSG00000073628,"ENSMUSG00000073628",4,2,3,5,2,6,1,3,5,2,2,3,1,9,1,8,0,2,2,1 | ENSMUSG00000065045,"ENSMUSG00000065045",0,0,0,0,0,0,0,0,0,0,0,2,0,0,0,0,0,0,0,0 |
| ENSMUSG00000028719,"ENSMUSG00000028719",2785,2374,2780,3005,2916,2518,2462,1896,2697,2880,2653,2254,2140,2499,2427,2920,2569,3072,1957,2543 | ENSMUSG00000109141,"ENSMUSG00000109141",0,0,0,0,0,1,2,0,0,0,2,0,5,0,0,0,0,0,0,0 | ENSMUSG00000076037,"ENSMUSG00000076037",0,0,1,0,0,0,0,0,0,0,0,0,0,0,1,0,0,0,1,0 |
| ENSMUSG00000027597,"ENSMUSG00000027597",487,477,701,555,511,599,519,416,443,480,537,448,586,516,480,582,427,679,515,566 | ENSMUSG00000091277,"ENSMUSG00000091277",1,4,2,0,1,4,6,0,3,1,2,3,1,1,5,1,0,2,0,1 | ENSMUSG00000103350,"ENSMUSG00000103350",0,0,0,0,0,0,0,0,0,0,0,0,0,0,0,0,0,0,0,0 |
| ENSMUSG00000026922,"ENSMUSG00000026922",435,586,793,635,711,631,678,508,664,825,604,530,639,689,602,700,625,842,473,668 | ENSMUSG00000025652,"ENSMUSG00000025652",0,0,2,0,0,0,1,0,0,0,0,4,0,0,1,0,0,0,1,0 | ENSMUSG00000102462,"ENSMUSG00000102462",0,0,0,0,0,0,0,0,0,0,0,0,0,0,0,3,0,0,0,0 |
| ENSMUSG00000013593,"ENSMUSG00000013593",1777,2325,3120,3003,2969,2438,2649,2080,2484,2838,2776,2184,2899,2712,2522,2937,2445,3176,2571,2818 | ENSMUSG00000083261,"ENSMUSG00000083261",4,0,3,6,3,4,6,3,5,3,8,4,6,3,7,8,4,7,1,4 | ENSMUSG00000097299,"ENSMUSG00000097299",0,0,0,0,0,0,0,0,0,0,0,0,0,0,0,0,0,0,0,0 |
| ENSMUSG00000000544,"ENSMUSG00000000544",0,0,0,0,0,2,0,0,0,3,2,0,1,1,0,1,0,0,0,1 | ENSMUSG00000078125,"ENSMUSG00000078125",6,3,5,3,3,7,5,6,4,7,2,5,2,8,5,7,6,9,4,2 | ENSMUSG00000095388,"ENSMUSG00000095388",0,0,0,0,0,0,0,0,0,0,0,0,0,0,0,0,0,0,0,0 |
| ENSMUSG00000074622,"ENSMUSG00000074622",9476,13179,16864,16516,16911,15345,12469,11232,12675,16692,14500,10879,13557,16584,13750,16619,14825,15756,9842,12170 | ENSMUSG00000117250,"ENSMUSG00000117250",0,2,0,1,2,3,3,2,1,3,5,3,1,1,0,1,3,1,0,0 | ENSMUSG00000088016,"ENSMUSG00000088016",0,0,0,0,0,0,0,0,0,0,0,0,0,0,0,0,0,0,0,0 |
| ENSMUSG00000032458,"ENSMUSG00000032458",3054,3591,4403,4700,4477,4200,3983,3373,3461,4048,4417,3545,4153,4057,3935,4404,3826,4765,3260,3821 | ENSMUSG00000086644,"ENSMUSG00000086644",4,8,9,2,13,3,0,8,9,2,5,4,6,17,6,5,3,4,3,2 | ENSMUSG00000085318,"ENSMUSG00000085318",0,0,0,0,0,0,0,0,0,0,1,0,0,0,0,0,0,0,0,0 |
| ENSMUSG00000024981,"ENSMUSG00000024981",1830,1963,2302,2834,2178,2170,2578,1841,2029,2003,2483,2086,2067,2250,2132,2114,1961,2535,1985,1999 | ENSMUSG00000049571,"ENSMUSG00000049571",2,4,4,3,3,2,3,2,2,1,6,4,7,1,2,4,7,7,1,3 | ENSMUSG00000106036,"ENSMUSG00000106036",0,0,0,0,0,0,0,0,0,0,0,0,0,0,0,0,0,0,0,0 |
| ENSMUSG00000022710,"ENSMUSG00000022710",3373,3971,4327,4456,4200,3964,3991,3145,3936,4284,4556,3762,3817,4022,3713,4236,3801,4629,2982,3537 | ENSMUSG00000110409,"ENSMUSG00000110409",3,5,12,6,1,8,2,5,5,3,5,2,3,7,2,4,3,3,4,7 | ENSMUSG00000106562,"ENSMUSG00000106562",0,0,0,0,0,0,0,0,0,0,0,0,0,0,0,0,0,0,0,0 |
| ENSMUSG00000034243,"ENSMUSG00000034243",1659,2063,2638,2650,2907,2570,2231,2037,2086,2535,2695,1951,2653,2535,2443,2811,2477,2880,1767,2178 | ENSMUSG00000116478,"ENSMUSG00000116478",0,0,0,0,0,1,1,0,0,0,0,1,0,0,0,0,0,0,0,0 | ENSMUSG00000104860,"ENSMUSG00000104860",0,0,0,0,0,0,0,0,0,0,0,0,0,0,0,0,0,0,0,0 |
| ENSMUSG00000051695,"ENSMUSG00000051695",2022,3663,4983,4645,4117,4115,3991,3198,3589,4413,4587,3683,4331,4200,3890,4524,3682,4929,3652,3966 | ENSMUSG00000100084,"ENSMUSG00000100084",10,5,15,9,11,9,5,4,11,5,12,9,6,8,3,5,7,15,3,13 | ENSMUSG00000065552,"ENSMUSG00000065552",0,0,0,0,0,0,0,0,0,0,0,0,0,0,0,0,0,0,0,0 |
| ENSMUSG00000022551,"ENSMUSG00000022551",1428,2285,2821,2663,2573,2231,2605,1959,2195,2408,2791,2215,2421,2410,2370,2593,2278,3105,2638,2504 | ENSMUSG00000022056,"ENSMUSG00000022056",0,0,0,0,0,0,0,0,0,0,0,0,0,0,0,0,0,0,0,0 | ENSMUSG00000106072,"ENSMUSG00000106072",0,0,0,0,0,0,0,0,1,0,0,0,0,0,0,0,0,0,0,2 |
| ENSMUSG00000035852,"ENSMUSG00000035852",30,50,52,76,71,57,53,33,76,42,49,54,79,67,74,103,60,99,52,61 | ENSMUSG00000086228,"ENSMUSG00000086228",12,10,11,12,15,10,8,5,17,14,4,8,3,7,7,20,13,17,6,7 | ENSMUSG00000087877,"ENSMUSG00000087877",0,0,0,0,0,0,0,3,0,0,0,0,0,0,0,0,0,0,0,0 |
| ENSMUSG00000021111,"ENSMUSG00000021111",3508,2983,3670,3634,3779,3617,3341,2890,3087,3306,3891,2778,3233,3329,3265,3606,3242,3983,2494,3065 | ENSMUSG00000086071,"ENSMUSG00000086071",4,4,7,2,1,3,3,6,6,8,14,8,8,4,9,8,6,7,2,9 | ENSMUSG00000105952,"ENSMUSG00000105952",0,0,0,0,0,0,0,0,0,0,0,0,0,0,0,0,0,0,0,0 |

| ENSMUSG00000003053,"ENSMUSG00000003053",0,0,0,0,0,0,1,2,2,0,0,0,0,2,0,0,0,0,1,3 | ENSMUSG00000098449,"ENSMUSG00000098449",5,3,9,6,2,2,6,3,0,8,3,0,6,6,5,10,2,5,1,6 | ENSMUSG00000106141,"ENSMUSG00000106141",0,0,0,0,0,0,0,0,0,0,0,0,0,0,0,0,0,0,0,0 |
| --- | --- | --- |
| ENSMUSG00000028792,"ENSMUSG00000028792",1717,3345,4215,4401,3647,3019,3596,2926,3195,3533,4079,3343,3472,4024,3871,4040,3416,4215,3461,3647 | ENSMUSG00000047085,"ENSMUSG00000047085",2,8,7,4,1,10,2,3,10,5,4,2,3,7,8,0,1,4,4,10 | ENSMUSG00000105880,"ENSMUSG00000105880",0,0,0,0,0,0,0,0,0,0,0,0,0,0,0,0,0,0,0,0 |
| ENSMUSG00000048154,"ENSMUSG00000048154",1659,3089,3782,3465,3821,3517,3300,2711,3182,3197,3943,2738,3601,3517,3206,3936,3456,4170,2625,2931 | ENSMUSG00000097742,"ENSMUSG00000097742",44,30,32,25,38,40,25,34,24,50,28,22,39,34,20,31,28,24,30,33 | ENSMUSG00000102569,"ENSMUSG00000102569",0,0,0,0,0,0,0,0,0,0,0,0,0,0,0,0,0,0,0,0 |
| ENSMUSG00000020580,"ENSMUSG00000020580",2549,2180,2498,2613,2523,2561,2247,1885,2193,2705,2332,1773,1939,2392,2124,2557,2367,2800,1439,1921 | ENSMUSG00000063412,"ENSMUSG00000063412",12,11,15,15,13,9,8,5,8,5,10,7,12,10,11,11,13,16,8,12 | ENSMUSG00000090040,"ENSMUSG00000090040",0,0,0,0,0,0,0,0,0,0,0,0,0,0,0,0,0,0,0,2 |
| ENSMUSG00000060373,"ENSMUSG00000060373",3502,3664,4514,4335,4195,4073,4100,3463,3846,4502,4223,3360,4027,4250,3936,4341,3897,4619,3252,4142 | ENSMUSG00000106494,"ENSMUSG00000106494",0,0,0,0,0,0,0,0,0,0,0,0,0,0,0,0,0,0,0,0 | ENSMUSG00000104696,"ENSMUSG00000104696",0,0,0,0,0,0,0,0,0,0,0,0,0,0,0,0,0,0,0,0 |
| ENSMUSG00000022149,"ENSMUSG00000022149",1,3,0,0,3,1,5,6,7,6,1,2,2,2,1,4,3,0,3,5 | ENSMUSG00000064918,"ENSMUSG00000064918",26,8,13,9,6,10,10,3,14,14,14,8,6,17,7,7,14,8,1,14 | ENSMUSG00000104647,"ENSMUSG00000104647",0,0,0,0,0,0,0,0,0,0,0,0,0,0,0,0,0,0,0,0 |
| ENSMUSG00000001794,"ENSMUSG00000001794",1929,2733,3905,3024,3624,3519,3426,2859,3237,3563,3176,2407,3275,3341,2849,3558,3060,3959,2992,3745 | ENSMUSG00000034452,"ENSMUSG00000034452",17,8,15,12,15,12,11,16,8,22,15,12,17,19,12,25,15,7,4,11 | ENSMUSG00000083632,"ENSMUSG00000083632",0,0,0,0,0,0,0,0,0,0,0,0,0,0,0,0,0,0,0,0 |
| ENSMUSG00000033684,"ENSMUSG00000033684",3777,5002,6920,5777,6338,6516,5753,5547,5279,6352,5224,4075,6016,6674,5463,6599,5268,6719,5238,5978 | ENSMUSG00000092889,"ENSMUSG00000092889",6,8,15,11,9,12,8,5,4,17,12,9,8,10,8,12,12,16,6,12 | ENSMUSG00000081711,"ENSMUSG00000081711",0,0,0,0,0,0,0,0,0,0,0,0,0,0,0,0,0,0,0,0 |
| ENSMUSG00000056076,"ENSMUSG00000056076",3027,4765,6242,6473,5317,5251,5769,4098,4783,5068,5762,4982,4717,5140,4863,5416,4612,5895,4679,5101 | ENSMUSG00000112908,"ENSMUSG00000112908",4,2,16,7,10,14,5,6,6,4,11,11,7,4,7,11,7,10,0,7 | ENSMUSG00000082151,"ENSMUSG00000082151",0,2,0,0,0,0,0,0,0,0,0,1,0,0,0,0,0,0,0,0 |
| ENSMUSG00000075602,"ENSMUSG00000075602",12568,10047,12374,19846,12791,9218,10113,12054,12035,11629,16720,14714,13263,12076,13716,14862,11434,18054,13988,13063 | ENSMUSG00000116288,"ENSMUSG00000116288",3,5,6,4,4,5,2,0,4,2,11,2,2,2,3,5,9,3,5,0 | ENSMUSG00000084181,"ENSMUSG00000084181",0,0,0,0,0,0,0,0,0,0,0,0,0,0,0,0,0,0,0,0 |
| ENSMUSG00000025369,"ENSMUSG00000025369",1096,1905,2646,2454,2231,2236,2222,1932,2151,2198,2449,1931,2468,2347,2236,2352,2063,2832,2033,2188 | ENSMUSG00000103332,"ENSMUSG00000103332",10,5,9,15,18,6,8,2,11,6,13,7,7,7,11,10,2,6,5,10 | ENSMUSG00000085522,"ENSMUSG00000085522",0,0,0,0,0,0,0,0,0,0,0,0,0,0,0,0,2,0,0,0 |
| ENSMUSG00000029201,"ENSMUSG00000029201",1417,1319,1707,1864,1719,1707,1435,1308,1486,1740,1585,1191,1517,1621,1362,1522,1394,1861,1006,1425 | ENSMUSG00000046550,"ENSMUSG00000046550",6,7,14,7,3,3,10,3,16,11,12,5,23,1,8,12,6,14,11,13 | ENSMUSG00000081437,"ENSMUSG00000081437",0,0,0,0,0,0,0,0,0,0,0,0,0,0,0,0,0,0,0,0 |
| ENSMUSG00000029761,"ENSMUSG00000029761",754,833,1283,1023,1049,1561,1157,1070,846,1081,1117,546,1308,1035,968,1047,1001,1440,803,986 | ENSMUSG00000076436,"ENSMUSG00000076436",4,1,0,0,2,0,0,0,3,1,2,2,1,0,0,2,2,2,0,0 | ENSMUSG00000088528,"ENSMUSG00000088528",0,0,0,0,0,0,0,0,0,0,0,0,0,0,0,0,0,0,0,0 |
| ENSMUSG00000026003,"ENSMUSG00000026003",2311,2210,2828,3054,2519,2328,2542,2408,2663,2570,3160,2466,2795,2599,2621,3185,2508,3487,2748,2974 | ENSMUSG00000039563,"ENSMUSG00000039563",9,6,10,6,5,8,8,1,4,10,7,6,10,2,1,9,2,9,1,4 | ENSMUSG00000095258,"ENSMUSG00000095258",0,0,0,0,0,0,0,0,0,0,0,0,0,0,0,0,0,0,0,0 |
| ENSMUSG00000034120,"ENSMUSG00000034120",4962,3946,4683,5283,4900,4340,4348,3258,4268,4030,5078,4050,3835,3999,3902,4619,3731,4758,3868,3817 | ENSMUSG00000100465,"ENSMUSG00000100465",9,12,29,22,12,31,26,19,19,37,18,6,25,32,9,12,15,25,10,14 | ENSMUSG00000096596,"ENSMUSG00000096596",0,0,0,0,0,0,0,0,0,0,0,0,0,0,0,0,0,0,0,0 |
| ENSMUSG00000075700,"ENSMUSG00000075700",5880,3336,3981,4313,4081,3619,3687,2954,3469,4092,4153,3126,3346,3504,3428,3953,3425,4318,2527,3246 | ENSMUSG00000109304,"ENSMUSG00000109304",3,2,5,1,8,1,1,8,2,1,1,0,1,3,1,5,2,1,1,2 | ENSMUSG00000102450,"ENSMUSG00000102450",0,0,0,0,0,0,0,0,0,0,1,0,0,0,0,0,0,0,0,0 |
| ENSMUSG00000019278,"ENSMUSG00000019278",120,211,261,240,209,231,188,186,206,218,215,182,218,217,188,215,204,272,218,183 | ENSMUSG00000049699,"ENSMUSG00000049699",13,8,48,23,20,25,24,34,29,19,29,20,24,20,25,30,32,23,19,45 | ENSMUSG00000081975,"ENSMUSG00000081975",0,0,0,0,0,0,0,0,0,0,0,0,0,0,0,0,0,0,0,0 |
| ENSMUSG00000064294,"ENSMUSG00000064294",12,1,12,9,11,13,2,10,15,9,12,13,5,7,11,2,2,8,8,9 | ENSMUSG00000116270,"ENSMUSG00000116270",5,3,6,8,0,4,3,4,3,6,3,4,9,5,2,1,4,4,4,1 | ENSMUSG00000082363,"ENSMUSG00000082363",0,0,0,0,0,0,0,0,0,0,0,0,0,0,0,0,0,0,0,0 |
| ENSMUSG00000034127,"ENSMUSG00000034127",72,86,110,103,88,91,105,70,68,102,116,85,91,75,93,108,71,84,68,93 | ENSMUSG00000050195,"ENSMUSG00000050195",16,12,20,16,17,10,9,10,16,5,11,12,14,13,21,11,8,8,13,12 | ENSMUSG00000070102,"ENSMUSG00000070102",3,0,0,0,0,0,0,0,0,0,0,0,0,0,0,0,0,0,0,0 |
| ENSMUSG00000038708,"ENSMUSG00000038708",2024,2470,3096,3241,3102,2664,2801,2395,2475,2806,3303,2370,3066,3005,2726,2983,2642,3296,2291,2434 | ENSMUSG00000107211,"ENSMUSG00000107211",4,9,5,3,4,3,5,1,1,4,3,2,5,7,6,3,7,3,2,2 | ENSMUSG00000086790,"ENSMUSG00000086790",0,0,0,0,0,0,0,0,0,0,0,0,0,0,0,0,0,0,0,0 |
| ENSMUSG00000037706,"ENSMUSG00000037706",2332,3421,5067,4820,4672,3547,4241,4181,4036,4122,4744,4232,4174,4236,4295,4613,3838,4496,3565,4420 | ENSMUSG00000098715,"ENSMUSG00000098715",0,0,0,4,0,1,3,5,0,4,0,0,1,0,1,2,2,0,5,2 | ENSMUSG00000082368,"ENSMUSG00000082368",0,0,0,0,0,0,0,0,0,0,0,0,0,0,0,0,0,0,0,0 |
| ENSMUSG00000029922,"ENSMUSG00000029922",1121,1272,1790,1513,1521,1457,1332,1241,1398,1501,1510,1111,1575,1437,1305,1589,1348,1906,1203,1533 | ENSMUSG00000051262,"ENSMUSG00000051262",0,2,3,3,3,1,2,4,0,3,5,2,14,0,4,2,4,2,6,2 | ENSMUSG00000086696,"ENSMUSG00000086696",0,0,0,0,0,0,0,0,0,0,0,0,0,0,0,0,0,0,0,0 |
| ENSMUSG00000019558,"ENSMUSG00000019558",2296,2721,3206,4278,3418,2755,3008,2439,2878,2776,4126,3218,3070,3101,2731,3305,2686,3502,2779,2699 | ENSMUSG00000113441,"ENSMUSG00000113441",0,0,0,2,0,0,0,0,0,0,0,0,0,0,0,0,0,0,1,0 | ENSMUSG00000084173,"ENSMUSG00000084173",0,0,0,0,0,0,0,0,0,0,0,0,0,0,0,0,0,0,0,0 |
| ENSMUSG00000030082,"ENSMUSG00000030082",4276,4133,5250,5665,5183,5198,5496,4100,4850,4703,5429,4025,4490,5034,4448,5048,4250,5404,3894,4771 | ENSMUSG00000073640,"ENSMUSG00000073640",9,5,15,10,10,4,9,10,8,5,10,4,9,8,12,7,8,10,2,7 | ENSMUSG00000095935,"ENSMUSG00000095935",0,0,3,0,0,0,0,0,0,0,0,0,0,0,0,0,0,0,0,0 |
| ENSMUSG00000023070,"ENSMUSG00000023070",27,35,23,48,15,10,6,3,13,15,10,16,18,17,4,15,7,20,7,9 | ENSMUSG00000056306,"ENSMUSG00000056306",0,0,0,0,0,2,0,0,2,0,0,0,0,1,0,0,0,0,0,0 | ENSMUSG00000098217,"ENSMUSG00000098217",0,0,0,0,0,0,0,0,0,0,0,0,0,0,0,0,0,0,0,0 |
| ENSMUSG00000020593,"ENSMUSG00000020593",336,365,540,510,538,363,382,396,392,389,547,333,454,388,419,415,363,422,351,379 | ENSMUSG00000033368,"ENSMUSG00000033368",14,7,14,18,11,19,23,26,22,24,28,18,28,14,16,21,19,19,18,27 | ENSMUSG00000038330,"ENSMUSG00000038330",0,0,0,0,0,0,0,0,0,0,0,0,0,0,0,0,0,0,0,3 |
| ENSMUSG00000044393,"ENSMUSG00000044393",229,226,274,257,258,266,234,163,232,234,275,219,199,251,197,221,185,235,183,219 | ENSMUSG00000106457,"ENSMUSG00000106457",30,22,25,29,12,24,23,12,19,9,19,21,15,18,10,16,11,23,8,20 | ENSMUSG00000063376,"ENSMUSG00000063376",0,0,0,0,0,2,0,0,0,0,0,0,0,1,0,0,0,0,0,0 |
| ENSMUSG00000008393,"ENSMUSG00000008393",3579,3745,4766,4966,4609,4250,4387,3521,3983,4416,3716,3440,3695,4316,4033,5134,4110,5239,2943,4061 | ENSMUSG00000070564,"ENSMUSG00000070564",5,6,6,4,9,2,5,2,2,8,7,2,2,5,6,8,3,3,12,10 | ENSMUSG00000070904,"ENSMUSG00000070904",0,0,0,0,0,0,0,0,0,0,0,1,0,2,0,0,0,0,0,0 |
| ENSMUSG00000020122,"ENSMUSG00000020122",2965,3116,3207,4479,3538,3066,3674,2388,3204,3235,3127,3246,2354,3492,3045,3318,3007,3445,2435,2840 | ENSMUSG00000083394,"ENSMUSG00000083394",2,7,16,8,2,7,10,7,13,8,11,9,8,12,5,11,3,13,7,6 | ENSMUSG00000081508,"ENSMUSG00000081508",0,0,0,0,3,0,0,0,0,0,0,0,0,0,0,0,0,0,0,0 |
| ENSMUSG00000035203,"ENSMUSG00000035203",1056,2498,3622,2893,3166,2878,2720,2565,2508,2749,3214,2430,3307,2782,2656,3318,2865,3625,2710,2913 | ENSMUSG00000097667,"ENSMUSG00000097667",8,9,8,8,15,15,8,11,9,23,11,4,6,7,9,9,11,14,2,12 | ENSMUSG00000070900,"ENSMUSG00000070900",0,0,0,0,0,0,0,0,0,0,0,0,0,0,0,0,0,0,0,0 |
| ENSMUSG00000024949,"ENSMUSG00000024949",2117,3824,4571,4665,4232,4070,3931,3209,4057,4374,4527,3762,3976,4313,3940,4321,3926,5117,3331,3907 | ENSMUSG00000040629,"ENSMUSG00000040629",0,0,0,0,0,0,0,0,0,0,0,0,0,0,0,0,0,0,0,0 | ENSMUSG00000082683,"ENSMUSG00000082683",0,0,0,0,0,0,0,0,0,0,0,0,0,0,0,0,0,0,0,0 |
| ENSMUSG00000078429,"ENSMUSG00000078429",2022,2406,3600,3246,3429,3232,2658,2581,2719,3156,3535,2631,3915,2874,2667,3270,2832,4034,2659,2895 | ENSMUSG00000029155,"ENSMUSG00000029155",0,0,0,0,0,0,3,0,0,0,2,0,0,0,0,0,0,5,0,0 | ENSMUSG00000102578,"ENSMUSG00000102578",0,0,0,0,0,0,0,0,0,0,1,0,0,0,0,0,0,0,0,0 |
| ENSMUSG00000057530,"ENSMUSG00000057530",3067,5452,6046,7753,6206,5782,6311,4919,5988,6310,6267,5349,5193,6364,5443,6615,5470,7372,4807,5204 | ENSMUSG00000106662,"ENSMUSG00000106662",12,10,25,24,33,31,21,21,15,18,18,19,19,9,16,23,18,28,28,25 | ENSMUSG00000087482,"ENSMUSG00000087482",0,0,0,0,0,0,0,0,0,0,0,0,0,0,0,0,0,0,0,0 |
| ENSMUSG00000068267,"ENSMUSG00000068267",1893,2924,4466,3876,3619,3462,3109,2804,2749,3261,3931,2954,3795,3457,3335,3822,3146,4414,2942,3443 | ENSMUSG00000108321,"ENSMUSG00000108321",10,14,19,19,18,19,7,5,6,19,18,6,9,10,7,15,26,13,10,12 | ENSMUSG00000077514,"ENSMUSG00000077514",0,0,0,0,0,0,0,0,0,0,0,0,0,0,0,0,0,0,0,0 |
| ENSMUSG00000020594,"ENSMUSG00000020594",6341,4686,4870,5683,5496,4955,4970,3617,4857,5591,5003,4222,4131,4848,4431,5075,4521,5256,3135,3982 | ENSMUSG00000092222,"ENSMUSG00000092222",0,0,0,0,0,1,1,0,0,0,0,2,0,0,0,1,0,2,0,2 | ENSMUSG00000082958,"ENSMUSG00000082958",0,0,0,0,0,0,0,0,0,0,0,0,0,0,0,0,0,0,0,0 |
| ENSMUSG00000007041,"ENSMUSG00000007041",6420,8292,10552,10250,10384,9041,9779,8287,8934,9837,9149,7456,8932,10208,9338,11079,9094,11656,8128,9771 | ENSMUSG00000116174,"ENSMUSG00000116174",8,6,3,9,6,5,4,4,3,9,7,3,8,9,5,7,6,8,1,9 | ENSMUSG00000095361,"ENSMUSG00000095361",0,1,0,0,0,0,0,0,0,0,0,0,0,0,0,0,0,0,0,0 |
| ENSMUSG00000044786,"ENSMUSG00000044786",2918,7247,7507,6284,9219,8359,9520,11913,12000,9107,6437,4875,9569,10242,8109,9999,8647,9782,7013,11244 | ENSMUSG00000027505,"ENSMUSG00000027505",7,19,29,25,26,24,18,22,31,16,15,17,15,34,18,17,22,22,13,40 | ENSMUSG00000082771,"ENSMUSG00000082771",0,0,0,0,0,0,0,0,0,0,0,0,0,0,0,0,0,0,0,0 |
| ENSMUSG00000055302,"ENSMUSG00000055302",2859,3300,4877,4302,3872,3772,3928,3194,3496,3824,4169,3249,4002,3829,3646,4146,3344,4614,3190,4104 | ENSMUSG00000031965,"ENSMUSG00000031965",2,5,15,1,5,10,5,3,6,7,0,1,8,2,1,2,3,1,0,7 | ENSMUSG00000083904,"ENSMUSG00000083904",0,0,0,0,0,0,0,0,0,0,0,0,0,0,0,0,0,0,0,0 |
| ENSMUSG00000063065,"ENSMUSG00000063065",1126,2153,3199,2430,2908,2482,2565,2432,2434,2503,2763,2264,3275,2604,2643,3018,2362,3580,2350,3232 | ENSMUSG00000087330,"ENSMUSG00000087330",0,0,0,0,0,0,0,0,0,0,0,0,0,0,0,0,0,0,0,0 | ENSMUSG00000057977,"ENSMUSG00000057977",0,0,0,0,0,0,0,0,0,1,0,0,0,1,0,0,1,0,0,0 |
| ENSMUSG00000039952,"ENSMUSG00000039952",1683,2069,2830,2655,2996,2445,2229,2325,2486,2252,2804,2313,2936,2282,2293,2805,2321,3295,2192,2682 | ENSMUSG00000043460,"ENSMUSG00000043460",0,0,1,3,0,0,2,0,0,0,0,1,1,1,0,0,0,0,0,1 | ENSMUSG00000064547,"ENSMUSG00000064547",0,0,0,0,0,0,0,0,0,0,0,0,0,0,0,0,0,0,0,0 |
| ENSMUSG00000062997,"ENSMUSG00000062997",1026,4332,5956,5614,4658,4797,4661,3901,4439,5372,5649,4565,4900,4971,4738,5679,4211,5976,3788,5065 | ENSMUSG00000033255,"ENSMUSG00000033255",1,2,2,0,0,2,0,1,0,0,1,0,3,3,0,0,0,3,2,2 | ENSMUSG00000073764,"ENSMUSG00000073764",0,0,0,0,0,0,0,0,0,0,0,0,0,0,0,0,0,0,0,0 |
| ENSMUSG00000063358,"ENSMUSG00000063358",3183,3194,3786,3781,3512,3545,3368,2795,3234,3594,3952,2978,3291,3243,3154,3508,3138,3872,2724,3148 | ENSMUSG00000065715,"ENSMUSG00000065715",5,3,5,7,7,9,2,3,3,0,13,1,0,3,2,3,1,7,0,1 | ENSMUSG00000080934,"ENSMUSG00000080934",0,0,0,0,0,0,0,0,0,0,0,0,0,0,0,0,0,0,0,0 |
| ENSMUSG00000022463,"ENSMUSG00000022463",2805,3956,4015,4895,4523,3826,4572,3209,4261,3995,4456,4219,3840,4201,3548,4512,3735,4881,3805,3941 | ENSMUSG00000084616,"ENSMUSG00000084616",12,7,5,5,9,1,8,3,2,4,8,3,2,7,7,4,2,2,0,1 | ENSMUSG00000086138,"ENSMUSG00000086138",0,0,0,0,0,0,0,0,0,0,0,0,0,0,0,0,0,0,0,0 |
| ENSMUSG00000032314,"ENSMUSG00000032314",972,1331,1743,1775,1591,1403,1424,1266,1397,1501,1735,1268,1445,1584,1402,1699,1411,1831,1349,1625 | ENSMUSG00000066513,"ENSMUSG00000066513",0,0,0,0,0,0,0,0,0,0,0,0,0,0,0,0,0,0,0,1 | ENSMUSG00000087171,"ENSMUSG00000087171",0,0,0,0,0,0,0,0,0,0,0,0,0,0,0,0,0,0,0,0 |
| ENSMUSG00000026074,"ENSMUSG00000026074",6641,8434,11372,9649,10327,9872,9404,7843,8331,9928,9440,6978,9184,9813,8553,10105,8515,11120,7326,8687 | ENSMUSG00000087408,"ENSMUSG00000087408",12,17,17,12,4,10,10,8,15,9,6,11,11,3,10,7,13,12,11,15 | ENSMUSG00000095878,"ENSMUSG00000095878",0,0,0,0,0,0,0,0,0,0,0,0,0,0,0,0,0,0,0,0 |
| ENSMUSG00000063077,"ENSMUSG00000063077",2369,2404,3058,3083,3181,2561,2879,2372,2695,2884,3301,2584,3227,2729,2687,2996,2639,3177,2135,2772 | ENSMUSG00000075581,"ENSMUSG00000075581",8,8,17,6,5,7,2,7,18,12,13,8,5,5,10,6,9,5,1,12 | ENSMUSG00000095096,"ENSMUSG00000095096",0,0,0,0,0,0,0,0,0,0,0,0,0,0,0,0,0,0,0,0 |
| ENSMUSG00000028405,"ENSMUSG00000028405",750,828,1219,1177,1009,1048,964,839,857,968,1113,771,998,1003,930,874,898,1052,785,858 | ENSMUSG00000020660,"ENSMUSG00000020660",0,4,0,1,6,3,3,2,6,3,3,4,6,1,2,11,3,12,7,2 | ENSMUSG00000078491,"ENSMUSG00000078491",0,0,1,0,0,0,0,0,0,0,0,1,0,0,0,0,0,0,0,1 |
| ENSMUSG00000041736,"ENSMUSG00000041736",2406,3599,5386,5022,4346,4468,3872,4567,4110,4486,4817,3556,5821,4674,4677,5496,4674,6923,4686,5203 | ENSMUSG00000035983,"ENSMUSG00000035983",3,4,17,15,15,7,8,4,9,8,11,11,12,5,9,4,9,8,13,4 | ENSMUSG00000085390,"ENSMUSG00000085390",0,0,0,0,0,0,0,0,0,0,1,0,0,0,0,0,0,0,0,0 |
| ENSMUSG00000017760,"ENSMUSG00000017760",2874,4189,6201,5231,5207,4587,4496,4680,4670,4674,5658,3593,6182,5045,4530,5428,4380,5823,5038,5382 | ENSMUSG00000069911,"ENSMUSG00000069911",22,16,14,7,10,4,13,6,10,14,15,8,10,9,11,11,8,12,20,7 | ENSMUSG00000105722,"ENSMUSG00000105722",0,0,0,0,0,0,3,0,0,0,0,0,0,0,0,0,0,0,0,0 |
| ENSMUSG00000022314,"ENSMUSG00000022314",2359,2536,3161,3096,3097,3002,2679,2364,2690,2952,2810,2381,2868,2645,2623,3133,2542,3424,2094,2904 | ENSMUSG00000026611,"ENSMUSG00000026611",0,1,0,1,0,1,5,0,0,0,3,1,2,1,0,3,0,4,0,1 | ENSMUSG00000105980,"ENSMUSG00000105980",0,0,0,0,0,0,0,0,0,0,0,0,0,0,0,0,0,0,0,0 |
| ENSMUSG00000032536,"ENSMUSG00000032536",1248,1816,2333,2302,2202,1913,2115,1765,1866,2080,2465,1774,2268,2174,2151,2271,2120,2546,1717,1975 | ENSMUSG00000105517,"ENSMUSG00000105517",6,6,1,8,4,5,9,2,6,6,5,3,4,2,2,2,3,11,10,5 | ENSMUSG00000091422,"ENSMUSG00000091422",0,0,0,0,0,0,0,0,0,0,0,0,0,0,0,0,0,0,0,0 |
| ENSMUSG00000030131,"ENSMUSG00000030131",0,0,0,0,0,0,0,3,0,0,0,0,0,0,0,0,0,0,0,0 | ENSMUSG00000110647,"ENSMUSG00000110647",1,5,8,1,10,4,3,12,5,5,10,2,8,2,7,11,4,8,14,11 | ENSMUSG00000106034,"ENSMUSG00000106034",0,0,0,0,0,0,0,0,0,0,0,0,0,0,0,0,0,0,0,0 |
| ENSMUSG00000000631,"ENSMUSG00000000631",1976,3277,3799,4882,3675,2900,3863,3139,3289,3296,4572,3596,3887,3723,3586,4061,3266,4370,3430,3716 | ENSMUSG00000020673,"ENSMUSG00000020673",1,2,1,0,1,8,1,1,1,0,0,0,1,1,0,3,1,1,1,2 | ENSMUSG00000104774,"ENSMUSG00000104774",0,0,0,0,0,0,0,0,0,0,0,0,0,0,0,0,0,0,0,0 |
| ENSMUSG00000031246,"ENSMUSG00000031246",2783,1945,2552,2105,2448,2307,1863,1727,1858,2364,2144,1399,2162,2038,1760,1945,1904,2179,1207,1934 | ENSMUSG00000116004,"ENSMUSG00000116004",3,4,8,8,12,11,12,4,4,3,7,4,8,4,2,8,7,4,8,6 | ENSMUSG00000094105,"ENSMUSG00000094105",0,0,0,0,0,0,0,0,0,0,0,0,0,0,0,0,0,0,0,0 |
| ENSMUSG00000028127,"ENSMUSG00000028127",1326,1141,1268,1445,1255,1170,1306,1098,1272,1196,1596,1212,1326,1213,1200,1364,1070,1482,1088,1241 | ENSMUSG00000054764,"ENSMUSG00000054764",0,1,1,0,0,0,0,2,0,1,0,0,0,0,0,0,0,0,0,2 | ENSMUSG00000104638,"ENSMUSG00000104638",0,0,0,0,0,0,0,0,0,0,0,0,0,0,0,0,0,0,0,0 |
| ENSMUSG00000040225,"ENSMUSG00000040225",3425,4373,4595,5529,4845,4646,5275,3489,4709,4580,4834,4053,3829,4460,3928,4676,4429,4958,3372,3941 | ENSMUSG00000042385,"ENSMUSG00000042385",9,26,16,16,7,2,6,26,17,15,22,5,51,6,9,23,23,37,28,26 | ENSMUSG00000098233,"ENSMUSG00000098233",0,0,0,0,0,0,0,0,0,0,0,0,0,0,0,0,0,0,0,0 |
| ENSMUSG00000020458,"ENSMUSG00000020458",4765,5354,7004,6247,6284,6126,5532,4435,5096,6157,5612,4446,5650,5897,5185,5916,5649,7140,4041,5490 | ENSMUSG00000108371,"ENSMUSG00000108371",0,0,0,3,0,0,0,1,0,0,2,0,0,0,1,0,0,0,0,0 | ENSMUSG00000105571,"ENSMUSG00000105571",0,0,0,0,0,0,0,0,0,0,0,0,0,0,0,0,0,0,0,0 |
| ENSMUSG00000013663,"ENSMUSG00000013663",3354,2756,3567,3252,3081,3198,2594,2536,2813,3140,3593,2621,3122,2767,2771,3011,2964,3512,1862,2789 | ENSMUSG00000041596,"ENSMUSG00000041596",0,0,1,2,3,0,1,1,0,2,0,2,0,0,0,0,0,2,0,4 | ENSMUSG00000105036,"ENSMUSG00000105036",0,0,0,0,0,0,0,0,0,0,0,0,0,0,0,0,0,0,0,0 |
| ENSMUSG00000016256,"ENSMUSG00000016256",2510,4189,6822,6328,5143,4619,4550,5475,4699,5432,5863,3871,6812,5986,5380,6091,5106,6842,5517,6068 | ENSMUSG00000097756,"ENSMUSG00000097756",0,9,9,1,4,2,10,5,3,0,5,5,3,5,2,6,5,6,3,6 | ENSMUSG00000095550,"ENSMUSG00000095550",0,0,0,0,0,0,0,0,0,0,0,0,0,3,0,0,0,0,0,0 |
| ENSMUSG00000040747,"ENSMUSG00000040747",16945,11308,13769,10757,13789,13677,11543,10692,11836,15919,10662,7892,12409,14360,11190,13783,12046,12700,8320,11557 | ENSMUSG00000111006,"ENSMUSG00000111006",1,0,0,1,0,2,0,1,2,5,1,1,3,1,0,2,0,0,0,0 | ENSMUSG00000091049,"ENSMUSG00000091049",0,0,0,0,0,0,0,0,0,0,0,0,0,0,0,0,0,0,0,0 |
| ENSMUSG00000007034,"ENSMUSG00000007034",1,0,4,2,0,5,0,0,0,0,3,1,0,1,1,0,0,2,0,6 | ENSMUSG00000100241,"ENSMUSG00000100241",0,0,0,0,0,0,0,0,0,0,0,0,0,0,0,0,0,0,0,0 | ENSMUSG00000106551,"ENSMUSG00000106551",0,0,0,0,2,0,0,0,0,0,0,0,0,0,0,0,0,0,0,0 |
| ENSMUSG00000042699,"ENSMUSG00000042699",3556,3467,4037,4164,3864,3635,3588,2893,3474,4047,4394,3551,3533,3445,3350,3749,3244,3999,2633,3278 | ENSMUSG00000093722,"ENSMUSG00000093722",14,18,20,6,26,13,18,5,12,25,22,6,11,7,13,14,12,10,11,22 | ENSMUSG00000088327,"ENSMUSG00000088327",0,0,0,0,0,0,0,0,0,0,0,0,0,1,0,0,0,0,0,0 |
| ENSMUSG00000030096,"ENSMUSG00000030096",4816,5504,6477,5978,5912,6191,4701,4134,5105,6083,5972,4635,6019,5723,4482,5811,4718,6796,3934,4712 | ENSMUSG00000103837,"ENSMUSG00000103837",19,19,30,15,28,30,25,6,12,37,4,10,16,27,17,15,8,18,1,8 | ENSMUSG00000106866,"ENSMUSG00000106866",0,0,0,0,0,0,0,0,0,0,0,0,0,0,0,0,0,0,0,0 |
| ENSMUSG00000025060,"ENSMUSG00000025060",5711,6700,7582,8050,7720,6664,6888,5346,6422,7616,7131,6522,6168,6937,6498,7627,6220,8525,4939,6259 | ENSMUSG00000086163,"ENSMUSG00000086163",11,8,13,8,11,11,13,15,12,13,11,8,19,11,20,11,4,17,13,14 | ENSMUSG00000107316,"ENSMUSG00000107316",0,0,0,0,0,0,0,0,0,0,0,0,0,0,0,0,0,0,0,0 |
| ENSMUSG00000060743,"ENSMUSG00000060743",1294,3431,4170,3557,3948,3745,3668,3336,3289,3831,3970,2997,4014,3558,3288,4218,3373,4540,3493,3839 | ENSMUSG00000085054,"ENSMUSG00000085054",4,1,4,2,3,8,7,2,0,0,3,1,5,2,6,5,7,7,5,6 | ENSMUSG00000107131,"ENSMUSG00000107131",0,0,0,0,0,0,0,0,0,0,0,0,0,0,0,0,0,0,0,0 |
| ENSMUSG00000019888,"ENSMUSG00000019888",0,0,0,0,0,0,0,0,0,0,0,0,0,0,0,0,0,0,0,0 | ENSMUSG00000089953,"ENSMUSG00000089953",19,27,22,48,25,29,33,45,26,24,34,31,22,42,32,54,25,66,45,69 | ENSMUSG00000086422,"ENSMUSG00000086422",0,0,0,0,0,0,0,0,0,0,0,0,0,0,0,0,0,0,0,0 |
| ENSMUSG00000020361,"ENSMUSG00000020361",3639,3725,4596,4834,4443,4098,4451,3312,3879,4328,4620,3700,3824,4018,4005,4250,3789,4881,3001,4025 | ENSMUSG00000098889,"ENSMUSG00000098889",1,2,5,8,8,4,3,3,0,2,1,6,5,4,11,7,5,4,1,4 | ENSMUSG00000106924,"ENSMUSG00000106924",0,0,0,0,0,0,0,0,0,0,0,0,0,0,0,0,0,0,0,0 |
| ENSMUSG00000039001,"ENSMUSG00000039001",4235,3711,5277,4729,4047,3649,3296,3013,3795,4460,4305,3488,4176,4358,3354,5014,3169,5069,4892,4577 | ENSMUSG00000050138,"ENSMUSG00000050138",0,2,7,0,1,2,5,1,1,4,3,3,5,4,4,3,4,4,3,2 | ENSMUSG00000105653,"ENSMUSG00000105653",0,0,0,0,0,0,0,0,0,0,0,0,0,0,0,0,0,0,0,0 |
| ENSMUSG00000040848,"ENSMUSG00000040848",3745,3477,4115,4292,4528,3681,3740,3004,3503,4309,3995,3641,3910,4310,3609,4183,3593,4204,3023,3530 | ENSMUSG00000097519,"ENSMUSG00000097519",1,1,7,3,5,3,3,2,0,2,2,2,6,2,7,0,3,2,0,2 | ENSMUSG00000106408,"ENSMUSG00000106408",0,0,0,0,0,0,0,0,0,0,0,0,0,0,0,0,0,1,0,0 |
| ENSMUSG00000029472,"ENSMUSG00000029472",2310,2719,3309,3109,2989,2948,2975,2350,2528,2750,3294,2453,3076,2624,2443,2989,2646,3413,2513,2736 | ENSMUSG00000054003,"ENSMUSG00000054003",10,3,2,12,4,2,4,4,1,15,6,5,6,6,11,6,5,14,3,11 | ENSMUSG00000103544,"ENSMUSG00000103544",0,0,0,0,0,0,0,0,0,0,0,0,0,0,0,0,0,0,0,0 |
| ENSMUSG00000003420,"ENSMUSG00000003420",2294,3152,6440,4049,4173,4392,3534,3995,3248,3984,4769,2735,5219,3896,4001,4298,3782,4147,3630,4124 | ENSMUSG00000051076,"ENSMUSG00000051076",0,0,4,3,0,2,0,0,0,0,0,0,1,0,4,0,3,0,0,2 | ENSMUSG00000106579,"ENSMUSG00000106579",0,0,0,0,0,0,0,0,0,0,0,0,0,0,0,0,0,0,0,0 |
| ENSMUSG00000018171,"ENSMUSG00000018171",6345,4328,5660,5430,5863,5225,5140,4736,4543,5182,5340,3862,5528,5668,4856,5486,4394,6134,4292,4544 | ENSMUSG00000028865,"ENSMUSG00000028865",4,5,1,5,1,1,4,0,2,3,1,0,4,1,0,0,6,2,3,2 | ENSMUSG00000105423,"ENSMUSG00000105423",0,0,0,0,0,0,0,0,0,0,0,0,0,0,0,0,0,0,0,0 |
| ENSMUSG00000021660,"ENSMUSG00000021660",3585,4421,5270,5190,4932,4821,4843,3779,4294,4739,5155,4201,4115,4499,4248,4829,3818,5296,4204,4394 | ENSMUSG00000109587,"ENSMUSG00000109587",7,4,6,7,2,3,7,8,9,6,11,6,11,8,8,7,8,10,2,7 | ENSMUSG00000106337,"ENSMUSG00000106337",0,0,0,1,0,0,0,0,0,0,0,0,0,0,0,0,0,0,0,0 |
| ENSMUSG00000029430,"ENSMUSG00000029430",4043,4502,5619,5646,5029,4744,5371,3854,4383,4902,5588,4504,4370,4830,4645,4882,4302,5413,4507,4885 | ENSMUSG00000116617,"ENSMUSG00000116617",4,9,11,16,8,11,8,4,12,14,16,7,10,12,10,7,12,16,9,8 | ENSMUSG00000105835,"ENSMUSG00000105835",0,0,0,0,0,0,0,0,0,0,0,0,0,0,0,0,0,0,2,0 |
| ENSMUSG00000022533,"ENSMUSG00000022533",3109,2312,2494,2279,2660,2655,2514,1660,2167,2743,2223,2007,1848,2253,1960,2147,2085,2483,1408,1809 | ENSMUSG00000084761,"ENSMUSG00000084761",0,0,0,0,1,0,0,0,1,0,0,0,0,0,0,0,0,0,0,0 | ENSMUSG00000105489,"ENSMUSG00000105489",0,0,0,0,0,0,0,0,0,0,0,0,0,0,0,0,0,0,0,2 |
| ENSMUSG00000031311,"ENSMUSG00000031311",2098,2863,3862,3623,3403,3357,3179,2855,2843,3306,3779,2697,3550,3370,3130,3503,3025,3958,2978,3274 | ENSMUSG00000114206,"ENSMUSG00000114206",0,0,0,0,0,1,0,0,1,0,1,0,0,0,0,0,0,0,0,0 | ENSMUSG00000093644,"ENSMUSG00000093644",0,0,0,0,0,0,0,0,0,0,0,0,0,0,0,0,0,0,0,0 |
| ENSMUSG00000031167,"ENSMUSG00000031167",6868,8138,10486,9286,9671,9178,8427,6380,7747,9341,9517,7222,7647,8405,7358,9542,7330,9892,7254,7575 | ENSMUSG00000042501,"ENSMUSG00000042501",0,3,0,4,1,5,1,2,3,1,4,0,6,1,3,1,3,3,2,1 | ENSMUSG00000063935,"ENSMUSG00000063935",0,0,0,0,0,0,0,0,0,0,0,0,0,0,0,0,2,0,0,0 |
| ENSMUSG00000035000,"ENSMUSG00000035000",453,318,461,512,390,424,358,376,402,330,572,466,435,326,371,459,307,547,431,422 | ENSMUSG00000081643,"ENSMUSG00000081643",0,8,9,14,10,13,5,10,9,9,9,7,15,7,3,14,12,11,8,18 | ENSMUSG00000107027,"ENSMUSG00000107027",0,0,0,0,0,0,0,0,0,0,0,0,0,0,0,0,0,0,0,0 |
| ENSMUSG00000025823,"ENSMUSG00000025823",2701,3566,5862,6391,5004,4675,4670,4812,4000,4325,5611,3508,5020,5203,5449,4654,4649,4575,4141,4787 | ENSMUSG00000080254,"ENSMUSG00000080254",0,0,0,0,0,0,0,0,0,0,1,1,1,1,0,2,2,0,1,0 | ENSMUSG00000035946,"ENSMUSG00000035946",0,0,0,0,1,0,0,0,0,0,0,0,0,0,0,0,0,0,0,0 |
| ENSMUSG00000022150,"ENSMUSG00000022150",15235,14140,18330,17281,17526,19848,14536,13688,14983,19272,16027,10322,14832,18471,14926,14972,17477,15516,10418,14026 | ENSMUSG00000091648,"ENSMUSG00000091648",0,2,2,10,4,3,4,7,2,0,0,0,6,1,5,3,4,11,2,1 | ENSMUSG00000106704,"ENSMUSG00000106704",0,0,0,0,0,0,0,0,0,0,0,0,0,0,0,0,0,0,0,0 |
| ENSMUSG00000003226,"ENSMUSG00000003226",4413,3570,4331,4002,4698,4300,3577,3341,3887,4556,4096,3030,4172,4023,3856,4494,3910,4662,2446,3423 | ENSMUSG00000034990,"ENSMUSG00000034990",16,17,20,31,17,22,29,27,29,37,23,20,51,55,40,31,41,21,20,40 | ENSMUSG00000103684,"ENSMUSG00000103684",0,0,0,0,0,0,0,0,0,0,0,0,0,0,0,0,0,0,0,0 |
| ENSMUSG00000024507,"ENSMUSG00000024507",768,832,1269,1090,1007,994,943,839,821,945,1216,736,1156,943,878,989,834,1021,893,923 | ENSMUSG00000025129,"ENSMUSG00000025129",17,27,37,40,33,13,24,34,29,48,54,11,28,28,33,36,23,49,38,46 | ENSMUSG00000089481,"ENSMUSG00000089481",0,0,0,0,0,0,0,0,0,0,0,0,0,0,0,0,0,0,0,0 |
| ENSMUSG00000028412,"ENSMUSG00000028412",2973,2471,2886,2595,3367,2574,2761,2122,2660,2700,2709,2460,2326,2533,2455,3140,2454,3118,1953,2550 | ENSMUSG00000042540,"ENSMUSG00000042540",17,33,31,80,12,34,49,2,42,21,32,40,18,49,21,13,21,24,20,23 | ENSMUSG00000105704,"ENSMUSG00000105704",0,0,0,0,0,0,0,0,0,0,0,0,0,0,0,0,0,0,0,0 |
| ENSMUSG00000066800,"ENSMUSG00000066800",4337,4610,5242,5263,5177,5800,4571,3863,4107,6273,4740,3728,4181,5880,4859,5749,4610,5520,3106,4126 | ENSMUSG00000074529,"ENSMUSG00000074529",0,1,2,1,0,5,2,4,0,3,3,2,2,3,2,3,9,4,5,2 | ENSMUSG00000075983,"ENSMUSG00000075983",0,0,0,0,0,0,0,0,0,0,0,0,0,0,0,0,0,0,0,0 |
| ENSMUSG00000068086,"ENSMUSG00000068086",0,0,0,0,0,0,0,4,0,0,0,0,0,0,0,0,0,0,1,2 | ENSMUSG00000103135,"ENSMUSG00000103135",8,7,8,9,7,8,4,4,9,9,6,5,7,3,3,4,4,4,4,9 | ENSMUSG00000105246,"ENSMUSG00000105246",0,0,0,0,0,0,0,0,0,0,0,0,0,0,0,0,0,0,0,0 |
| ENSMUSG00000024085,"ENSMUSG00000024085",4626,4454,5554,4983,5355,5768,4260,4062,4727,5691,5054,3368,5345,4965,4582,5620,5029,6256,2893,4537 | ENSMUSG00000097216,"ENSMUSG00000097216",1,7,26,18,12,9,11,11,5,17,14,8,7,16,3,18,11,12,10,8 | ENSMUSG00000064156,"ENSMUSG00000064156",0,0,0,0,0,0,0,0,0,0,0,0,0,0,0,0,0,0,0,0 |
| ENSMUSG00000027546,"ENSMUSG00000027546",714,1021,1325,1326,1265,1095,1315,1084,1051,983,1332,1138,1133,1180,1102,1322,1043,1506,1184,1254 | ENSMUSG00000105601,"ENSMUSG00000105601",10,1,5,3,2,2,0,2,2,1,6,0,4,2,0,3,0,1,0,0 | ENSMUSG00000106725,"ENSMUSG00000106725",0,0,0,0,0,0,0,0,0,0,0,0,0,0,0,0,0,0,0,0 |
| ENSMUSG00000007836,"ENSMUSG00000007836",2066,4736,6432,5722,4891,5601,4539,4050,4634,5470,5936,4707,5616,5418,4889,5804,4759,6369,3841,4864 | ENSMUSG00000110996,"ENSMUSG00000110996",0,0,0,0,0,0,0,0,0,0,0,0,0,0,0,0,0,0,0,0 | ENSMUSG00000104545,"ENSMUSG00000104545",0,0,0,0,0,0,0,0,0,0,0,0,0,0,0,0,0,0,0,0 |
| ENSMUSG00000017421,"ENSMUSG00000017421",4198,3707,4184,4246,4288,4061,3807,3003,3707,4190,4298,3463,3716,3583,3444,4178,3485,4105,3069,3439 | ENSMUSG00000110627,"ENSMUSG00000110627",4,4,1,9,4,5,4,4,9,3,7,5,9,11,8,8,6,5,4,6 | ENSMUSG00000090146,"ENSMUSG00000090146",0,0,0,0,0,0,0,0,0,0,0,0,0,0,0,0,0,0,0,0 |
| ENSMUSG00000037965,"ENSMUSG00000037965",7085,5731,6170,6690,6820,5890,5797,4478,6000,6797,5929,5390,5035,6110,5226,6713,5435,7119,4184,5229 | ENSMUSG00000111828,"ENSMUSG00000111828",3,1,4,0,0,2,1,3,2,3,0,1,5,1,0,0,1,2,1,2 | ENSMUSG00000105018,"ENSMUSG00000105018",0,0,0,0,0,0,0,0,0,0,0,0,0,0,0,0,0,0,0,1 |
| ENSMUSG00000025366,"ENSMUSG00000025366",2083,2526,3506,3446,3322,3400,2965,2978,2876,2994,3101,2007,3502,3249,2936,3283,3012,3834,2617,3211 | ENSMUSG00000081399,"ENSMUSG00000081399",3,6,6,13,7,9,1,4,8,11,10,7,13,6,19,12,4,11,9,10 | ENSMUSG00000095133,"ENSMUSG00000095133",0,0,0,0,0,0,0,0,0,0,0,0,0,0,0,0,0,0,2,0 |
| ENSMUSG00000018339,"ENSMUSG00000018339",1180,1601,2675,1559,1942,2028,1477,1849,1680,1815,2090,1178,2595,1804,1711,1790,1690,2110,1793,2118 | ENSMUSG00000022525,"ENSMUSG00000022525",5,6,10,3,9,3,10,2,8,8,5,4,10,7,3,6,9,6,11,6 | ENSMUSG00000106594,"ENSMUSG00000106594",0,0,0,0,0,0,0,0,0,0,0,0,0,0,0,0,0,0,0,0 |
| ENSMUSG00000032902,"ENSMUSG00000032902",3052,2661,2989,3199,3089,2925,2631,2181,2427,2611,3267,2708,2416,2536,2449,2757,2228,2977,1948,2273 | ENSMUSG00000087120,"ENSMUSG00000087120",0,0,4,4,2,7,4,0,3,2,7,2,5,6,5,5,5,6,5,10 | ENSMUSG00000090961,"ENSMUSG00000090961",0,0,0,0,0,0,0,0,0,0,0,0,0,0,0,0,0,0,0,0 |
| ENSMUSG00000028410,"ENSMUSG00000028410",1560,2202,2799,2706,2404,2711,2447,2009,2174,2795,2704,1951,2346,2399,2315,2426,2183,2669,1902,2202 | ENSMUSG00000022580,"ENSMUSG00000022580",7,4,5,2,10,6,5,7,6,4,7,10,5,3,3,0,14,1,4,3 | ENSMUSG00000093510,"ENSMUSG00000093510",0,0,0,0,0,0,0,0,0,0,0,0,0,0,0,0,0,0,0,0 |
| ENSMUSG00000035325,"ENSMUSG00000035325",2387,2590,3198,3476,3296,3208,3393,2592,3027,3027,3350,2633,2980,3026,2831,3085,2617,3262,2680,3005 | ENSMUSG00000096141,"ENSMUSG00000096141",21,21,22,32,24,22,32,25,19,16,16,24,21,10,17,21,22,21,15,31 | ENSMUSG00000105603,"ENSMUSG00000105603",0,0,0,0,0,0,0,0,0,0,0,0,0,0,0,0,0,0,0,0 |
| ENSMUSG00000022347,"ENSMUSG00000022347",0,0,0,0,0,0,0,0,0,0,0,0,0,0,0,1,0,0,0,0 | ENSMUSG00000036586,"ENSMUSG00000036586",0,0,2,0,0,0,1,0,0,1,2,0,0,0,0,1,0,1,0,0 | ENSMUSG00000096949,"ENSMUSG00000096949",0,0,0,0,0,0,0,0,0,0,0,0,0,0,0,0,0,0,0,0 |
| ENSMUSG00000026457,"ENSMUSG00000026457",3566,3462,4886,4210,4268,4215,4025,3711,3890,4356,4125,3295,4503,4243,3606,4804,3767,4953,3440,4374 | ENSMUSG00000042976,"ENSMUSG00000042976",7,7,10,11,1,11,10,3,10,10,12,9,0,5,4,9,7,14,11,21 | ENSMUSG00000098027,"ENSMUSG00000098027",0,0,0,0,0,0,0,0,0,0,0,0,0,0,0,0,0,0,0,0 |
| ENSMUSG00000038462,"ENSMUSG00000038462",1983,2216,2613,2810,2654,2209,2406,1707,2163,2440,2382,2168,2229,2265,2174,2530,2112,2760,1979,2241 | ENSMUSG00000091160,"ENSMUSG00000091160",11,12,8,13,15,9,12,3,6,10,12,6,12,13,5,11,8,6,2,5 | ENSMUSG00000107353,"ENSMUSG00000107353",0,0,0,0,0,0,0,0,0,0,0,0,0,0,0,0,0,0,0,0 |
| ENSMUSG00000025283,"ENSMUSG00000025283",6438,6319,8217,6514,6740,7206,6709,5912,6800,8325,6260,4857,6878,6798,6158,7630,6138,8008,4703,7029 | ENSMUSG00000100481,"ENSMUSG00000100481",0,0,2,0,0,0,0,0,0,0,0,1,0,0,0,1,0,0,0,0 | ENSMUSG00000099047,"ENSMUSG00000099047",0,1,0,0,0,0,0,0,0,0,0,0,0,0,0,0,0,0,0,0 |
| ENSMUSG00000031451,"ENSMUSG00000031451",2070,3319,6249,4489,4556,4346,3410,4198,3899,4210,4911,3313,5775,4136,4139,4827,4235,5827,4672,5067 | ENSMUSG00000097433,"ENSMUSG00000097433",22,12,15,12,15,13,15,10,14,17,8,8,4,11,12,18,19,14,8,11 | ENSMUSG00000088085,"ENSMUSG00000088085",0,0,0,0,0,0,0,0,0,0,0,0,0,0,0,0,0,0,0,0 |
| ENSMUSG00000032349,"ENSMUSG00000032349",628,684,927,935,868,952,714,654,737,945,960,650,880,907,662,781,758,961,655,786 | ENSMUSG00000053916,"ENSMUSG00000053916",7,2,12,4,1,10,5,2,2,2,5,6,3,0,5,1,3,4,4,1 | ENSMUSG00000107144,"ENSMUSG00000107144",0,0,0,0,0,0,0,0,0,0,0,0,0,0,0,0,0,0,0,0 |
| ENSMUSG00000020891,"ENSMUSG00000020891",10559,16325,14010,23577,26647,10676,18880,17813,16446,13719,12901,19459,13744,19946,22299,24758,17480,21736,19818,17483 | ENSMUSG00000086638,"ENSMUSG00000086638",1,2,7,7,6,4,2,3,1,2,4,0,9,5,4,4,0,9,1,4 | ENSMUSG00000084548,"ENSMUSG00000084548",0,0,0,0,0,0,0,0,0,0,0,0,0,0,0,0,0,0,0,0 |
| ENSMUSG00000014313,"ENSMUSG00000014313",1846,2351,2749,2355,2375,2252,2267,1900,2206,2759,2806,2145,2265,2309,2043,2730,2134,2745,2133,2419 | ENSMUSG00000104621,"ENSMUSG00000104621",5,3,5,4,12,4,6,3,6,9,5,6,5,3,9,4,3,6,2,5 | ENSMUSG00000107053,"ENSMUSG00000107053",0,0,0,0,0,0,0,0,0,0,0,0,0,0,0,0,0,0,0,0 |
| ENSMUSG00000040462,"ENSMUSG00000040462",880,2246,3307,3159,2739,2846,2743,2669,2602,2749,2974,2223,3378,2932,2817,3357,2623,3459,2934,3072 | ENSMUSG00000076596,"ENSMUSG00000076596",0,0,0,0,0,0,0,0,0,0,0,3,3,0,0,0,0,2,0,0 | ENSMUSG00000105593,"ENSMUSG00000105593",0,0,0,0,0,0,0,0,0,0,0,0,0,3,0,0,0,0,0,0 |
| ENSMUSG00000000826,"ENSMUSG00000000826",5078,5243,6291,7387,6903,5917,5690,4827,5633,5884,5829,5498,5125,6083,5642,6753,5406,6657,4531,5285 | ENSMUSG00000043794,"ENSMUSG00000043794",3,0,8,2,6,3,5,4,0,3,3,3,4,5,3,5,3,2,5,6 | ENSMUSG00000104825,"ENSMUSG00000104825",0,0,0,0,0,0,0,0,0,0,0,0,0,0,0,0,0,0,0,0 |
| ENSMUSG00000028557,"ENSMUSG00000028557",5274,4256,4760,5157,5225,4835,4970,3664,4948,5103,4468,4184,4237,4749,4190,5203,4247,5065,3409,4342 | ENSMUSG00000115017,"ENSMUSG00000115017",4,7,6,10,5,5,2,2,0,5,6,4,7,3,3,5,8,9,0,3 | ENSMUSG00000105308,"ENSMUSG00000105308",0,0,0,0,0,0,0,0,0,0,0,0,0,0,0,0,0,0,0,0 |
| ENSMUSG00000045294,"ENSMUSG00000045294",837,852,905,1108,1043,918,823,692,801,835,904,1050,941,921,776,917,823,979,643,894 | ENSMUSG00000085069,"ENSMUSG00000085069",9,11,11,24,17,7,9,14,17,11,12,9,17,28,12,19,8,15,26,20 | ENSMUSG00000106729,"ENSMUSG00000106729",0,0,0,0,0,0,0,0,0,0,0,0,0,0,0,0,0,0,0,1 |
| ENSMUSG00000029076,"ENSMUSG00000029076",2458,3243,4504,4152,4041,3616,3334,3081,3176,3723,4037,3009,3882,3815,3542,4168,3408,4553,3203,3712 | ENSMUSG00000083169,"ENSMUSG00000083169",0,0,0,0,0,0,0,0,0,0,0,0,0,0,0,1,0,0,0,0 | ENSMUSG00000105470,"ENSMUSG00000105470",0,0,0,0,0,0,0,0,0,0,0,0,0,0,0,0,0,0,0,0 |
| ENSMUSG00000036402,"ENSMUSG00000036402",2857,3080,3693,4103,3778,3471,3123,2631,3140,3357,3764,3006,3010,3247,2931,3400,2964,3703,2591,2731 | ENSMUSG00000104061,"ENSMUSG00000104061",10,5,5,5,3,11,3,9,3,3,3,6,12,3,4,4,3,4,5,1 | ENSMUSG00000105584,"ENSMUSG00000105584",0,0,0,0,0,0,0,0,0,0,0,0,0,0,0,0,0,0,0,0 |
| ENSMUSG00000021749,"ENSMUSG00000021749",0,1,2,0,0,0,1,0,2,0,0,0,0,1,0,0,0,1,0,1 | ENSMUSG00000103976,"ENSMUSG00000103976",4,2,11,7,6,6,5,1,4,1,4,5,2,2,2,2,5,4,2,8 | ENSMUSG00000105720,"ENSMUSG00000105720",0,0,0,0,0,0,0,0,0,0,0,0,0,0,0,0,0,0,0,0 |
| ENSMUSG00000095041,"ENSMUSG00000095041",3444,4243,6170,5177,5512,5374,4598,4755,5021,4614,6241,3612,5961,4407,5274,5391,4606,6089,5331,5653 | ENSMUSG00000031202,"ENSMUSG00000031202",7,4,3,6,11,4,10,6,3,7,1,6,2,7,9,9,5,5,5,4 | ENSMUSG00000106090,"ENSMUSG00000106090",0,0,0,0,0,0,0,0,1,0,0,0,0,0,0,0,0,0,0,0 |
| ENSMUSG00000021000,"ENSMUSG00000021000",2063,1950,2167,2459,2256,2017,2145,1643,2070,2104,2266,1846,2070,1917,1749,2171,1912,2550,1516,1877 | ENSMUSG00000089687,"ENSMUSG00000089687",1,16,8,3,3,3,1,2,3,7,11,1,18,5,2,7,7,3,4,0 | ENSMUSG00000105390,"ENSMUSG00000105390",0,0,0,0,0,0,0,0,0,0,0,0,0,0,0,0,0,0,0,0 |
| ENSMUSG00000022139,"ENSMUSG00000022139",3947,3295,3553,4160,4368,3687,3414,3230,3745,4400,3997,3429,3711,3731,3335,4034,3343,4362,2534,3538 | ENSMUSG00000043456,"ENSMUSG00000043456",16,2,8,7,5,4,7,13,1,3,3,2,4,2,6,6,2,5,3,8 | ENSMUSG00000107098,"ENSMUSG00000107098",0,0,2,0,0,0,0,0,0,0,0,0,0,0,0,0,0,0,0,0 |
| ENSMUSG00000055436,"ENSMUSG00000055436",4079,3232,3629,3788,3652,3484,3336,2693,3425,3499,3804,2999,3083,3361,3044,3374,3068,3725,2748,2949 | ENSMUSG00000020723,"ENSMUSG00000020723",1,1,6,4,3,4,3,3,3,3,6,4,1,1,0,2,0,0,2,1 | ENSMUSG00000092764,"ENSMUSG00000092764",0,0,0,0,0,0,0,0,0,0,0,0,0,0,0,0,0,0,0,0 |
| ENSMUSG00000085438,"ENSMUSG00000085438",2297,1860,2373,2289,2617,2212,1914,1802,1794,2280,2670,1954,2550,1964,1909,2241,2008,2554,1546,1771 | ENSMUSG00000085260,"ENSMUSG00000085260",2,6,0,0,0,2,3,4,1,1,0,0,6,2,0,3,3,1,0,3 | ENSMUSG00000092674,"ENSMUSG00000092674",0,0,0,0,0,0,0,0,0,0,0,0,0,0,0,0,0,0,0,0 |
| ENSMUSG00000025780,"ENSMUSG00000025780",307,274,256,466,296,382,286,184,226,253,359,289,273,208,227,270,244,300,232,193 | ENSMUSG00000113750,"ENSMUSG00000113750",2,2,3,1,3,4,1,5,2,4,5,4,8,6,0,8,1,2,7,0 | ENSMUSG00000107279,"ENSMUSG00000107279",0,0,0,0,0,0,0,0,0,0,0,0,0,0,0,2,0,0,0,0 |
| ENSMUSG00000051853,"ENSMUSG00000051853",3381,3510,4573,4653,4459,4235,3941,3390,3655,4209,4275,3351,4159,4085,3802,4159,3727,4850,3585,3706 | ENSMUSG00000097567,"ENSMUSG00000097567",7,12,18,26,10,9,7,17,12,25,19,15,22,20,10,17,15,22,9,13 | ENSMUSG00000084717,"ENSMUSG00000084717",0,0,0,0,0,0,0,0,0,0,0,0,0,0,0,0,0,0,0,0 |
| ENSMUSG00000038375,"ENSMUSG00000038375",842,1240,1513,1580,1431,1199,1259,976,1271,1359,1712,1270,1558,1397,1171,1271,1188,1483,1255,1244 | ENSMUSG00000063687,"ENSMUSG00000063687",9,13,16,11,14,17,8,20,4,20,26,19,21,15,11,9,12,19,13,13 | ENSMUSG00000107967,"ENSMUSG00000107967",0,0,0,0,1,0,0,0,1,0,0,0,0,0,0,0,1,0,0,0 |
| ENSMUSG00000032637,"ENSMUSG00000032637",1928,4066,4641,5063,4798,4143,4328,3802,4111,4327,4561,3853,4134,4638,4319,4990,3999,5153,3828,3839 | ENSMUSG00000073010,"ENSMUSG00000073010",41,29,32,33,42,27,23,12,17,12,43,30,19,34,21,22,32,29,14,15 | ENSMUSG00000098326,"ENSMUSG00000098326",0,0,0,0,0,0,0,0,3,0,0,0,0,0,0,0,0,0,0,0 |
| ENSMUSG00000027002,"ENSMUSG00000027002",6137,4310,5045,5273,5499,4528,5064,3572,4669,4801,5137,4424,3896,4142,4409,4854,4127,5361,3335,4220 | ENSMUSG00000048916,"ENSMUSG00000048916",0,0,4,1,0,0,0,2,0,1,1,0,2,0,1,1,0,0,0,0 | ENSMUSG00000107847,"ENSMUSG00000107847",0,0,0,0,0,0,0,0,0,0,0,0,0,0,0,0,0,0,0,0 |
| ENSMUSG00000028381,"ENSMUSG00000028381",7796,6633,7548,7096,7896,7446,6946,6504,7896,8196,7662,6323,7133,7526,7462,7990,6677,8808,5191,7786 | ENSMUSG00000068417,"ENSMUSG00000068417",1,15,6,17,8,4,4,9,4,11,3,5,10,8,8,9,3,16,8,12 | ENSMUSG00000107422,"ENSMUSG00000107422",0,0,0,0,0,0,0,0,0,0,0,0,0,0,0,0,0,0,0,0 |
| ENSMUSG00000034024,"ENSMUSG00000034024",3883,4356,4934,5330,4634,4275,4769,3323,4209,4321,4708,4032,3739,4342,3993,4488,3929,4910,3666,4021 | ENSMUSG00000042761,"ENSMUSG00000042761",0,2,0,1,3,1,0,1,5,0,1,0,0,0,4,1,3,1,0,3 | ENSMUSG00000107562,"ENSMUSG00000107562",0,0,0,0,0,0,0,0,0,0,0,0,0,0,0,0,0,0,0,0 |
| ENSMUSG00000032116,"ENSMUSG00000032116",3856,3544,4399,4365,4328,4111,4022,3420,3437,3582,4306,3172,3749,4001,3595,3745,3546,4427,2925,3635 | ENSMUSG00000064140,"ENSMUSG00000064140",0,0,0,0,2,0,0,1,0,0,1,1,0,0,1,1,0,0,0,0 | ENSMUSG00000100101,"ENSMUSG00000100101",0,1,0,0,0,0,0,0,0,0,0,0,0,0,0,0,0,0,0,0 |
| ENSMUSG00000024073,"ENSMUSG00000024073",3101,2670,3527,3261,3618,3340,3148,2577,2950,3069,3197,2637,3030,3005,2790,3154,2796,3635,2114,2499 | ENSMUSG00000085001,"ENSMUSG00000085001",0,1,2,0,3,0,0,1,0,1,2,1,5,2,1,4,1,1,1,0 | ENSMUSG00000106826,"ENSMUSG00000106826",0,0,0,0,0,0,0,0,0,0,0,0,0,0,0,0,0,2,0,0 |
| ENSMUSG00000023073,"ENSMUSG00000023073",0,0,0,0,0,0,0,0,0,0,0,0,0,0,0,0,0,0,0,0 | ENSMUSG00000027777,"ENSMUSG00000027777",4,9,11,9,14,13,10,13,11,16,10,5,12,17,13,8,9,17,6,9 | ENSMUSG00000106989,"ENSMUSG00000106989",0,0,0,0,0,0,0,0,0,0,0,0,0,2,0,0,0,0,0,1 |
| ENSMUSG00000038145,"ENSMUSG00000038145",1659,1761,2154,2278,2079,1671,1940,1786,1968,1931,2141,1657,2260,1915,1849,2323,1612,2473,1738,1819 | ENSMUSG00000109509,"ENSMUSG00000109509",3,3,16,10,14,3,15,7,12,11,15,7,14,8,16,8,10,10,1,12 | ENSMUSG00000090692,"ENSMUSG00000090692",0,0,0,0,0,0,0,0,0,0,0,0,0,0,0,0,0,0,0,0 |
| ENSMUSG00000035569,"ENSMUSG00000035569",2508,4065,4497,4880,4576,4372,4448,3496,4135,4409,4838,3694,4129,4496,3968,4491,4053,5013,3384,3694 | ENSMUSG00000029875,"ENSMUSG00000029875",2,0,5,4,6,4,5,5,1,6,7,4,18,2,4,13,5,12,1,6 | ENSMUSG00000098260,"ENSMUSG00000098260",0,0,0,0,0,0,0,0,0,0,0,0,0,0,0,0,0,0,0,0 |
| ENSMUSG00000058126,"ENSMUSG00000058126",4175,5577,6736,6406,6954,6929,6586,5450,6226,6651,5902,4861,5551,6238,5352,6639,5749,6948,4645,6270 | ENSMUSG00000106668,"ENSMUSG00000106668",0,0,0,0,0,0,0,0,0,0,0,1,0,0,0,0,0,0,0,0 | ENSMUSG00000086720,"ENSMUSG00000086720",0,0,0,0,2,0,0,0,0,0,0,0,0,0,0,0,0,0,0,0 |
| ENSMUSG00000022680,"ENSMUSG00000022680",1639,1579,2070,2036,1875,1785,1798,1425,1656,1647,2004,1453,1726,1745,1506,1867,1662,2128,1320,1585 | ENSMUSG00000067261,"ENSMUSG00000067261",1,9,13,6,9,8,14,5,6,15,25,10,8,15,7,11,14,14,8,5 | ENSMUSG00000103833,"ENSMUSG00000103833",0,0,0,0,0,0,0,0,0,0,0,0,0,0,0,0,1,0,0,0 |
| ENSMUSG00000024953,"ENSMUSG00000024953",2236,3218,4918,4392,3733,3586,3222,3980,3333,3533,4824,3315,5494,4373,3889,4907,3601,5834,4252,4704 | ENSMUSG00000106028,"ENSMUSG00000106028",2,8,5,5,4,7,6,0,11,7,8,3,7,4,3,9,3,8,8,3 | ENSMUSG00000107650,"ENSMUSG00000107650",0,0,0,0,0,0,0,0,0,0,0,0,0,0,0,0,0,0,0,0 |
| ENSMUSG00000028042,"ENSMUSG00000028042",833,2077,3188,2864,2821,2612,2464,2713,2358,2575,3087,2209,3372,2636,2791,2851,2445,3731,2782,2902 | ENSMUSG00000110998,"ENSMUSG00000110998",2,1,0,1,2,0,1,0,1,0,0,0,0,0,0,0,0,0,3,1 | ENSMUSG00000107465,"ENSMUSG00000107465",0,0,0,0,0,0,0,0,0,0,0,0,0,0,0,0,0,0,0,0 |
| ENSMUSG00000019122,"ENSMUSG00000019122",17013,11874,15985,15965,14546,12754,10762,9863,13288,18583,13167,9980,9614,15296,12058,13067,12893,11230,7363,9896 | ENSMUSG00000085941,"ENSMUSG00000085941",3,8,18,7,10,7,9,5,5,2,9,2,4,14,10,8,6,9,2,5 | ENSMUSG00000107920,"ENSMUSG00000107920",0,0,0,0,1,0,0,0,0,0,0,0,0,0,0,0,0,0,0,0 |
| ENSMUSG00000033634,"ENSMUSG00000033634",0,0,0,0,0,1,1,0,0,0,4,2,0,0,5,0,0,0,1,1 | ENSMUSG00000103592,"ENSMUSG00000103592",1,0,3,0,0,0,1,1,2,0,2,0,2,0,2,0,0,3,1,0 | ENSMUSG00000081872,"ENSMUSG00000081872",0,0,0,0,1,0,0,0,0,2,0,0,0,0,0,0,0,0,0,0 |
| ENSMUSG00000022051,"ENSMUSG00000022051",3442,3560,4538,4830,4923,3729,3861,4245,3952,4046,4359,4183,4838,4197,4460,5675,4040,6037,4036,4870 | ENSMUSG00000098661,"ENSMUSG00000098661",0,9,4,3,6,0,6,5,6,3,5,10,9,2,4,3,3,4,4,1 | ENSMUSG00000095670,"ENSMUSG00000095670",0,0,0,0,0,0,0,0,0,0,0,0,0,0,0,0,0,0,0,0 |
| ENSMUSG00000046062,"ENSMUSG00000046062",3211,2876,2931,3262,3028,3238,3043,2670,3084,3774,3240,2447,2840,3322,2966,3483,2982,3686,2018,2970 | ENSMUSG00000110710,"ENSMUSG00000110710",6,4,6,5,5,1,5,5,0,2,5,2,4,4,3,4,4,4,3,4 | ENSMUSG00000096371,"ENSMUSG00000096371",0,0,0,0,0,0,0,0,0,0,0,0,0,0,0,0,0,0,0,0 |
| ENSMUSG00000055670,"ENSMUSG00000055670",2243,2179,2555,2435,2545,2430,2348,2067,2252,2408,2293,1773,2365,2419,2182,2584,2213,2893,1788,2152 | ENSMUSG00000111424,"ENSMUSG00000111424",2,4,4,2,5,3,5,9,7,7,8,0,2,8,4,1,10,19,7,5 | ENSMUSG00000108262,"ENSMUSG00000108262",0,0,0,0,0,0,0,0,0,0,0,2,0,0,0,0,0,0,0,0 |
| ENSMUSG00000060615,"ENSMUSG00000060615",0,0,0,0,1,0,0,0,2,0,0,0,0,0,0,0,0,1,1,0 | ENSMUSG00000102573,"ENSMUSG00000102573",18,19,17,22,31,21,30,14,10,24,25,16,29,16,23,26,28,16,20,28 | ENSMUSG00000115170,"ENSMUSG00000115170",0,0,0,0,0,0,0,0,0,0,0,0,0,0,0,0,0,0,0,0 |
| ENSMUSG00000040054,"ENSMUSG00000040054",3216,3609,4385,4505,4640,3830,4092,3527,4010,4059,4374,3522,4294,4076,3805,4614,3946,5112,3619,3956 | ENSMUSG00000113775,"ENSMUSG00000113775",7,3,8,3,8,14,6,8,5,13,2,5,6,6,9,8,5,6,5,6 | ENSMUSG00000106480,"ENSMUSG00000106480",0,0,0,0,0,0,0,0,0,0,0,0,0,0,0,0,0,0,0,0 |
| ENSMUSG00000020358,"ENSMUSG00000020358",1616,4662,6048,5692,5098,5311,5086,3598,3998,5131,5614,4793,4830,4791,4783,5263,4449,5582,4434,4652 | ENSMUSG00000112254,"ENSMUSG00000112254",8,9,6,11,11,7,11,6,9,13,7,8,15,3,2,14,5,11,4,7 | ENSMUSG00000105463,"ENSMUSG00000105463",0,0,0,0,0,0,0,0,0,0,0,0,0,0,0,0,0,0,0,0 |

| ENSMUSG00000026796,"ENSMUSG00000026796",7587,11449,14028,14779,13459,11949,13517,10485,12089,13336,12411,10931,12087,13695,11914,15302,12019,16529,11471,13313 | ENSMUSG00000070570,"ENSMUSG00000070570",6,6,19,12,16,12,24,8,12,11,7,2,16,6,10,15,8,12,5,17 | ENSMUSG00000106315,"ENSMUSG00000106315",0,0,0,0,0,0,0,0,0,0,0,0,0,0,0,0,0,0,0,0 |
| --- | --- | --- |
| ENSMUSG00000006522,"ENSMUSG00000006522",26,25,58,29,44,26,39,33,43,74,46,24,51,46,42,47,30,35,33,41 | ENSMUSG00000090535,"ENSMUSG00000090535",0,0,0,0,0,0,0,0,0,0,0,0,0,0,0,0,0,0,0,0 | ENSMUSG00000108073,"ENSMUSG00000108073",0,0,0,0,0,0,0,0,0,0,0,0,0,0,0,0,0,0,0,0 |
| ENSMUSG00000021877,"ENSMUSG00000021877",2686,3399,4035,4803,4142,3796,3945,3204,4048,3858,3855,3083,3292,3800,3461,4348,3487,4439,3181,3800 | ENSMUSG00000074461,"ENSMUSG00000074461",3,1,2,0,1,1,1,3,2,0,3,3,2,0,1,2,1,2,3,0 | ENSMUSG00000106154,"ENSMUSG00000106154",0,0,0,0,0,0,0,0,0,0,0,0,0,0,0,0,0,0,0,0 |
| ENSMUSG00000024456,"ENSMUSG00000024456",4446,5459,6140,5954,6021,6001,5651,4720,5298,6906,5212,4466,5429,6390,5346,6740,5710,6990,3649,5608 | ENSMUSG00000103037,"ENSMUSG00000103037",1,8,13,3,2,4,7,3,5,4,3,2,10,16,5,11,8,4,7,6 | ENSMUSG00000096051,"ENSMUSG00000096051",0,0,0,0,0,0,0,0,0,0,0,0,0,0,0,0,0,1,0,0 |
| ENSMUSG00000028820,"ENSMUSG00000028820",3004,3403,4078,3780,3796,4159,3719,2568,3273,4053,4134,3206,3347,3331,3158,3698,3220,4138,2554,3149 | ENSMUSG00000050840,"ENSMUSG00000050840",1,1,1,0,0,0,0,0,3,1,3,2,0,0,3,0,0,0,0,4 | ENSMUSG00000064724,"ENSMUSG00000064724",0,1,0,0,0,0,0,0,0,0,0,0,0,0,0,0,0,0,0,0 |
| ENSMUSG00000042079,"ENSMUSG00000042079",3099,3856,4516,4899,4877,4480,4632,3500,3761,4173,4389,3713,3692,4228,3919,4228,3723,4748,4343,3762 | ENSMUSG00000114306,"ENSMUSG00000114306",0,0,0,0,0,0,0,0,0,0,0,2,2,0,0,0,0,0,0,0 | ENSMUSG00000107887,"ENSMUSG00000107887",0,0,0,0,0,0,0,0,0,0,0,0,0,0,0,0,0,0,0,0 |
| ENSMUSG00000027940,"ENSMUSG00000027940",4248,5338,6429,6498,5917,5860,5952,4828,5335,6477,5815,4403,5607,6500,5516,6332,5740,7297,5111,5876 | ENSMUSG00000028004,"ENSMUSG00000028004",3,1,3,3,4,0,5,13,3,3,4,1,3,5,3,6,3,1,3,3 | ENSMUSG00000107919,"ENSMUSG00000107919",0,0,0,1,0,0,0,0,2,0,0,0,0,0,0,0,0,0,0,0 |
| ENSMUSG00000000805,"ENSMUSG00000000805",332,701,1061,959,888,895,831,914,685,973,1053,552,1001,1026,927,818,878,900,716,840 | ENSMUSG00000028950,"ENSMUSG00000028950",8,9,7,6,3,6,4,3,3,9,14,2,12,13,6,9,3,4,7,2 | ENSMUSG00000088265,"ENSMUSG00000088265",0,0,1,0,0,0,0,0,1,1,0,0,0,0,0,0,0,0,0,0 |
| ENSMUSG00000028478,"ENSMUSG00000028478",3532,4699,7122,5998,5993,5776,5104,4806,4605,5747,5905,4174,6059,5621,5544,5886,5719,6376,4657,5953 | ENSMUSG00000062458,"ENSMUSG00000062458",2,8,7,10,9,7,10,5,5,11,13,7,11,3,5,19,9,14,1,11 | ENSMUSG00000107823,"ENSMUSG00000107823",0,0,0,0,1,0,0,0,0,0,0,0,0,0,0,0,0,0,0,2 |
| ENSMUSG00000033880,"ENSMUSG00000033880",2806,2942,4575,5971,3902,3648,3587,5682,4729,3756,4805,3106,5472,4226,4556,6012,4375,6589,5080,5485 | ENSMUSG00000087249,"ENSMUSG00000087249",3,3,3,6,9,6,6,6,10,8,3,1,7,9,8,7,6,13,5,3 | ENSMUSG00000107714,"ENSMUSG00000107714",0,0,0,0,0,0,0,0,0,0,0,0,0,0,0,0,0,0,0,0 |
| ENSMUSG00000026878,"ENSMUSG00000026878",4798,3933,4930,4819,5160,4794,4205,3469,4183,4708,4876,3762,3897,4222,3863,4491,4135,4587,3434,3841 | ENSMUSG00000045498,"ENSMUSG00000045498",5,7,13,17,5,7,6,4,3,10,6,1,16,11,8,14,10,14,6,9 | ENSMUSG00000107541,"ENSMUSG00000107541",0,0,0,0,0,0,0,0,0,0,0,0,0,0,0,0,0,0,1,0 |
| ENSMUSG00000009470,"ENSMUSG00000009470",5752,4158,5233,4822,5673,5001,4141,3959,4445,4778,4920,4127,4438,4437,4220,4806,4324,5556,2968,4081 | ENSMUSG00000103553,"ENSMUSG00000103553",5,5,3,12,4,13,2,12,10,15,9,4,8,4,10,9,3,7,8,5 | ENSMUSG00000089115,"ENSMUSG00000089115",0,0,0,0,0,0,0,0,0,0,0,0,0,0,0,0,0,0,0,0 |
| ENSMUSG00000023067,"ENSMUSG00000023067",5492,7239,8370,7491,8184,7210,8352,7459,7990,7773,5915,5395,7127,9104,7030,8816,7041,8461,7023,8085 | ENSMUSG00000064916,"ENSMUSG00000064916",2,4,7,4,13,9,5,7,5,8,14,11,4,2,6,8,3,19,4,7 | ENSMUSG00000095086,"ENSMUSG00000095086",0,0,0,0,0,0,0,0,0,0,2,0,0,0,0,0,0,0,0,0 |
| ENSMUSG00000018909,"ENSMUSG00000018909",811,1142,1723,1503,1830,1369,1009,1701,1222,1170,1711,1218,2376,1381,1538,1926,1434,2184,1349,1544 | ENSMUSG00000076489,"ENSMUSG00000076489",10,2,2,3,3,7,3,3,1,1,6,4,5,3,5,4,2,3,6,0 | ENSMUSG00000107471,"ENSMUSG00000107471",0,0,0,0,0,0,0,0,0,0,0,0,1,0,0,0,0,0,0,0 |
| ENSMUSG00000041417,"ENSMUSG00000041417",1747,1665,2235,2265,2205,2257,1759,1748,1789,2140,2391,1496,2206,1995,1814,2018,1916,2379,1438,1873 | ENSMUSG00000115593,"ENSMUSG00000115593",9,10,10,10,8,12,15,3,6,3,5,7,6,8,3,7,7,8,5,10 | ENSMUSG00000088835,"ENSMUSG00000088835",0,0,0,0,0,0,0,0,0,0,0,0,1,0,0,0,0,0,0,0 |
| ENSMUSG00000038650,"ENSMUSG00000038650",7836,6964,8339,9959,8420,7230,8196,6267,7762,7806,8207,7148,7152,8289,7156,8749,7073,9081,6302,7660 | ENSMUSG00000031204,"ENSMUSG00000031204",3,7,7,5,8,6,7,6,4,18,5,2,13,13,7,11,10,9,7,6 | ENSMUSG00000107623,"ENSMUSG00000107623",0,0,1,0,0,0,0,0,0,0,0,0,0,0,0,0,0,0,0,0 |
| ENSMUSG00000019432,"ENSMUSG00000019432",2226,3211,3829,4084,3605,3338,3965,2829,3543,3498,4196,3132,3228,3566,3315,3680,3130,3859,3406,3197 | ENSMUSG00000110887,"ENSMUSG00000110887",10,0,3,0,2,2,1,5,8,1,3,2,5,4,1,3,6,3,1,1 | ENSMUSG00000107253,"ENSMUSG00000107253",0,0,0,0,0,0,0,0,0,0,0,0,0,0,0,0,0,0,0,0 |
| ENSMUSG00000032115,"ENSMUSG00000032115",2541,2834,4069,4775,3816,3686,4197,3364,3426,3375,4042,2864,3575,3902,3935,3770,3393,3784,3406,3489 | ENSMUSG00000083677,"ENSMUSG00000083677",50,40,20,9,38,21,33,22,31,40,28,24,23,44,20,37,17,22,18,35 | ENSMUSG00000067541,"ENSMUSG00000067541",0,0,0,0,0,0,0,0,0,0,0,0,0,0,0,0,0,0,0,0 |
| ENSMUSG00000027195,"ENSMUSG00000027195",2981,2894,3382,3717,3382,3124,3359,2358,3087,3050,3504,2887,2830,3290,2705,3044,2745,3264,2321,2678 | ENSMUSG00000026459,"ENSMUSG00000026459",21,46,56,47,28,14,33,57,39,45,23,28,29,34,29,33,29,35,35,47 | ENSMUSG00000108248,"ENSMUSG00000108248",0,0,0,0,0,0,0,0,0,0,0,0,0,0,0,0,0,0,0,0 |
| ENSMUSG00000022234,"ENSMUSG00000022234",3138,3445,4077,4352,3768,3456,3644,2681,3013,3685,3975,3115,3185,3615,3340,3797,3220,3915,3122,3422 | ENSMUSG00000106961,"ENSMUSG00000106961",19,4,16,10,23,11,19,10,18,12,20,11,15,13,15,9,17,33,15,14 | ENSMUSG00000108047,"ENSMUSG00000108047",0,0,0,0,0,0,0,0,0,0,0,0,0,0,0,0,0,0,0,0 |
| ENSMUSG00000078672,"ENSMUSG00000078672",0,0,0,0,0,0,3,0,7,0,0,0,0,0,0,0,0,0,2,3 | ENSMUSG00000032017,"ENSMUSG00000032017",0,3,2,4,3,0,0,0,0,8,1,2,4,3,1,2,0,0,1,5 | ENSMUSG00000107891,"ENSMUSG00000107891",0,0,0,0,0,0,0,0,0,0,0,0,0,0,0,0,0,0,0,0 |
| ENSMUSG00000003948,"ENSMUSG00000003948",199,166,249,137,216,199,173,183,218,187,243,148,233,146,139,182,171,224,150,211 | ENSMUSG00000113650,"ENSMUSG00000113650",10,3,7,1,1,3,4,5,6,3,8,2,6,9,2,3,8,8,1,7 | ENSMUSG00000107398,"ENSMUSG00000107398",0,0,0,0,0,0,0,0,0,0,0,0,1,0,0,0,0,0,0,0 |
| ENSMUSG00000006529,"ENSMUSG00000006529",7,17,9,4,17,11,5,9,8,14,3,9,4,13,4,3,7,5,4,8 | ENSMUSG00000116505,"ENSMUSG00000116505",1,7,6,7,11,8,8,4,5,7,5,5,7,2,7,6,5,4,8,8 | ENSMUSG00000087302,"ENSMUSG00000087302",0,0,0,0,0,0,0,0,0,0,0,0,0,0,0,0,0,0,0,0 |
| ENSMUSG00000030403,"ENSMUSG00000030403",1679,4319,5718,4417,4990,5478,4536,4674,4282,5790,4905,3509,6052,5649,4971,6163,4996,6626,4207,5754 | ENSMUSG00000090217,"ENSMUSG00000090217",0,2,2,0,1,3,1,1,1,0,3,0,1,1,5,2,1,0,0,1 | ENSMUSG00000085600,"ENSMUSG00000085600",0,0,0,0,0,0,0,2,0,0,0,0,0,0,0,0,1,0,0,0 |
| ENSMUSG00000031725,"ENSMUSG00000031725",13,11,7,8,18,10,6,7,8,19,5,7,6,6,8,2,13,25,7,9 | ENSMUSG00000061833,"ENSMUSG00000061833",10,7,30,19,15,24,9,17,10,21,34,14,22,28,18,20,23,15,14,26 | ENSMUSG00000107405,"ENSMUSG00000107405",0,0,0,0,0,0,0,0,0,0,0,0,0,0,0,0,0,0,0,0 |
| ENSMUSG00000022365,"ENSMUSG00000022365",2222,2317,3164,3101,2980,2747,2696,2435,2675,2740,3039,2373,2877,2753,2650,2851,2544,3326,2401,2828 | ENSMUSG00000026650,"ENSMUSG00000026650",2,5,3,6,2,2,2,0,4,10,4,4,6,10,0,9,6,6,4,1 | ENSMUSG00000108712,"ENSMUSG00000108712",0,0,0,0,2,0,0,0,0,0,0,0,0,0,0,0,0,0,0,0 |
| ENSMUSG00000024782,"ENSMUSG00000024782",1372,1015,1144,1323,1307,1196,1238,813,1035,1079,1349,1102,1170,1048,1035,1121,1021,1211,895,1098 | ENSMUSG00000088008,"ENSMUSG00000088008",6,8,10,12,5,8,10,6,10,11,10,10,12,9,13,8,9,13,12,9 | ENSMUSG00000035177,"ENSMUSG00000035177",0,0,0,0,0,0,0,0,0,0,0,0,0,0,0,0,0,0,0,0 |
| ENSMUSG00000027288,"ENSMUSG00000027288",3444,3700,4668,4249,4687,3673,3982,3402,3688,4175,4180,3389,4230,4012,4005,4382,4123,4880,3135,3814 | ENSMUSG00000097509,"ENSMUSG00000097509",5,3,2,3,2,3,3,4,2,5,6,2,0,3,6,2,2,9,7,2 | ENSMUSG00000109283,"ENSMUSG00000109283",0,0,0,3,0,0,0,0,0,0,0,0,0,0,0,0,0,0,0,0 |
| ENSMUSG00000067212,"ENSMUSG00000067212",5163,5761,7885,9789,7382,6598,6647,7685,6913,6861,9132,6206,9354,8133,8308,9264,7327,11183,7449,8352 | ENSMUSG00000091443,"ENSMUSG00000091443",4,3,12,10,10,2,11,3,8,5,18,2,11,9,5,7,10,6,1,8 | ENSMUSG00000109177,"ENSMUSG00000109177",0,0,0,1,0,0,0,0,0,0,0,0,0,1,0,0,0,0,0,0 |
| ENSMUSG00000028868,"ENSMUSG00000028868",2312,3375,4283,4351,4016,3684,3630,3505,3797,4097,4160,3202,4035,3689,3511,4455,3737,4688,3198,3837 | ENSMUSG00000065547,"ENSMUSG00000065547",1,14,9,12,10,12,8,7,12,5,10,7,10,10,10,7,11,19,2,24 | ENSMUSG00000092624,"ENSMUSG00000092624",0,0,0,0,0,3,0,0,0,0,0,0,0,0,0,0,0,0,0,0 |
| ENSMUSG00000072849,"ENSMUSG00000072849",0,0,0,0,0,1,0,0,1,3,1,0,0,4,0,1,1,9,1,0 | ENSMUSG00000103763,"ENSMUSG00000103763",3,10,1,1,7,4,4,2,0,10,4,3,0,1,1,1,1,7,3,5 | ENSMUSG00000082872,"ENSMUSG00000082872",0,0,0,0,0,0,0,0,0,0,0,0,0,0,0,0,0,0,0,0 |
| ENSMUSG00000021248,"ENSMUSG00000021248",1870,3030,3908,3857,3749,3354,3420,3084,3215,3459,3648,2748,3562,3449,3147,3585,3230,4129,3193,3540 | ENSMUSG00000079697,"ENSMUSG00000079697",3,2,4,9,8,11,5,4,7,2,19,12,8,4,12,7,2,13,2,12 | ENSMUSG00000110365,"ENSMUSG00000110365",0,0,0,0,0,0,0,0,0,0,0,0,0,0,1,0,0,0,0,0 |
| ENSMUSG00000058546,"ENSMUSG00000058546",441,4223,4514,4981,4192,4378,4424,3384,3646,4304,4431,3863,4146,3933,4199,5085,3658,4732,4915,4228 | ENSMUSG00000097728,"ENSMUSG00000097728",4,1,19,0,11,6,7,4,6,8,6,1,10,3,8,4,8,16,7,7 | ENSMUSG00000109159,"ENSMUSG00000109159",0,0,0,0,0,0,0,0,0,0,0,0,0,0,0,0,0,0,0,0 |
| ENSMUSG00000005506,"ENSMUSG00000005506",2562,2541,3022,2935,2942,2724,2854,2250,2438,2805,2946,2190,2545,2775,2468,2766,2424,3048,2090,2412 | ENSMUSG00000108660,"ENSMUSG00000108660",1,6,3,2,5,6,0,2,5,2,0,1,2,1,0,0,4,0,1,7 | ENSMUSG00000070150,"ENSMUSG00000070150",0,0,0,0,0,0,0,0,0,0,0,0,0,0,0,0,0,0,0,0 |
| ENSMUSG00000005125,"ENSMUSG00000005125",7115,8081,10570,10569,14037,9869,9148,12057,9124,9059,7849,7610,10274,10913,10105,13262,9798,14411,8656,10638 | ENSMUSG00000090070,"ENSMUSG00000090070",3,6,3,3,2,6,3,2,6,5,4,1,9,6,5,8,4,9,7,7 | ENSMUSG00000108675,"ENSMUSG00000108675",0,0,0,0,0,0,0,0,0,0,0,0,3,0,0,0,0,0,0,0 |
| ENSMUSG00000049470,"ENSMUSG00000049470",5277,5027,5351,5514,5917,5217,5473,4095,5332,5874,4878,4631,4719,5120,4697,5654,4976,6004,3180,4498 | ENSMUSG00000113268,"ENSMUSG00000113268",23,40,30,39,49,42,39,13,34,61,21,35,14,32,16,29,27,31,17,24 | ENSMUSG00000108546,"ENSMUSG00000108546",0,0,0,0,0,0,0,0,0,0,0,0,0,0,0,0,0,0,0,0 |
| ENSMUSG00000007564,"ENSMUSG00000007564",3151,3545,4306,4462,3970,3786,4179,3241,3760,3957,4105,3390,3690,3941,3451,4090,3452,4693,3589,3888 | ENSMUSG00000030344,"ENSMUSG00000030344",0,0,2,1,0,2,0,0,2,0,0,0,1,0,0,1,0,0,0,0 | ENSMUSG00000078759,"ENSMUSG00000078759",0,0,0,0,0,0,0,0,0,0,0,0,0,0,0,0,0,0,0,0 |
| ENSMUSG00000034908,"ENSMUSG00000034908",3800,4483,5276,5279,5134,4845,4876,4179,4686,4831,4688,3969,4922,5010,4133,5623,4346,5788,3869,4914 | ENSMUSG00000045942,"ENSMUSG00000045942",4,2,3,5,6,4,5,3,5,6,6,10,5,4,3,6,5,7,3,1 | ENSMUSG00000084296,"ENSMUSG00000084296",0,0,0,0,0,0,0,0,0,0,0,0,0,0,0,0,0,0,0,0 |
| ENSMUSG00000032328,"ENSMUSG00000032328",3243,2282,2790,2603,2902,2656,2341,2246,2287,2522,2843,2074,2732,2513,2251,2451,2379,2957,1875,2453 | ENSMUSG00000082644,"ENSMUSG00000082644",0,0,0,0,0,0,1,0,0,0,0,0,0,0,0,0,0,0,0,0 | ENSMUSG00000081768,"ENSMUSG00000081768",0,0,0,0,0,0,0,0,3,0,0,0,0,0,0,0,0,0,0,0 |
| ENSMUSG00000005312,"ENSMUSG00000005312",2571,2645,3105,3201,3093,2861,3047,2226,2749,2814,3251,2685,2707,2659,2661,3029,2450,3288,2443,2782 | ENSMUSG00000111738,"ENSMUSG00000111738",0,2,1,1,3,3,3,7,7,1,5,3,3,1,2,3,2,1,6,0 | ENSMUSG00000083434,"ENSMUSG00000083434",0,0,0,0,0,0,0,0,0,0,0,0,0,0,0,0,0,0,0,0 |
| ENSMUSG00000015405,"ENSMUSG00000015405",1211,1218,1632,1476,1259,1287,850,1212,1116,942,1522,987,1417,1181,1095,1792,1038,1968,1492,1423 | ENSMUSG00000089317,"ENSMUSG00000089317",4,4,11,6,2,10,5,4,1,3,5,0,4,0,3,7,1,5,3,1 | ENSMUSG00000101410,"ENSMUSG00000101410",0,0,0,0,0,0,0,0,0,0,0,0,0,0,0,0,0,0,0,0 |
| ENSMUSG00000002812,"ENSMUSG00000002812",2878,3443,4640,4435,4306,3919,4288,3766,4006,4061,4013,3181,4143,4521,3844,4622,3867,4988,3623,4258 | ENSMUSG00000099238,"ENSMUSG00000099238",0,5,3,6,1,7,4,6,2,2,2,2,2,7,7,2,6,3,9,3 | ENSMUSG00000094920,"ENSMUSG00000094920",0,0,0,0,1,1,0,0,0,0,0,0,0,0,0,0,0,0,0,0 |
| ENSMUSG00000052337,"ENSMUSG00000052337",2221,2134,2651,2865,2790,2291,2394,2016,2398,2421,2710,2234,2181,2381,2136,2530,2223,2726,1946,2312 | ENSMUSG00000055197,"ENSMUSG00000055197",0,0,0,0,0,0,0,0,0,0,0,0,0,0,0,0,0,0,0,0 | ENSMUSG00000101470,"ENSMUSG00000101470",0,0,0,0,0,0,0,0,0,0,0,0,0,0,0,0,0,2,0,0 |
| ENSMUSG00000037400,"ENSMUSG00000037400",4216,3747,4405,4362,5039,4410,4003,3795,4130,4787,4328,3595,4223,4471,4107,5110,4031,5776,2854,3974 | ENSMUSG00000112265,"ENSMUSG00000112265",17,7,18,19,12,19,12,10,19,15,12,13,18,11,16,7,21,13,9,12 | ENSMUSG00000078737,"ENSMUSG00000078737",0,0,0,1,0,0,0,0,0,0,0,0,0,0,0,0,0,0,0,0 |
| ENSMUSG00000005148,"ENSMUSG00000005148",3299,4168,3698,4382,4136,3455,4597,2854,4538,4177,4224,3957,3176,3783,3363,4290,3378,3987,2603,3843 | ENSMUSG00000096938,"ENSMUSG00000096938",5,1,3,1,0,2,2,0,2,3,1,0,1,4,4,0,7,1,3,0 | ENSMUSG00000091004,"ENSMUSG00000091004",0,0,0,0,0,0,0,0,0,0,0,0,0,0,0,0,0,0,0,0 |
| ENSMUSG00000059316,"ENSMUSG00000059316",1831,2706,3350,3177,2958,3414,3244,2918,2890,3180,3118,2517,3522,3469,2788,3630,2901,4177,2866,3603 | ENSMUSG00000097334,"ENSMUSG00000097334",2,5,2,3,4,3,3,2,0,5,4,5,1,0,3,6,3,1,5,3 | ENSMUSG00000108459,"ENSMUSG00000108459",0,0,0,0,0,0,0,0,0,0,0,0,0,0,0,0,0,0,0,0 |
| ENSMUSG00000024190,"ENSMUSG00000024190",6975,7797,9021,8752,9130,6388,10808,15989,16796,11145,5983,5176,9707,15956,10464,10423,11695,9191,7065,14702 | ENSMUSG00000082100,"ENSMUSG00000082100",1,0,3,1,1,1,4,2,1,0,2,2,2,2,1,0,0,2,3,0 | ENSMUSG00000108719,"ENSMUSG00000108719",0,0,0,0,0,0,0,0,0,0,0,0,0,0,0,0,0,0,0,0 |
| ENSMUSG00000036698,"ENSMUSG00000036698",3549,3499,3826,4013,4063,3667,4226,3188,3776,3916,3974,3271,3501,3865,3573,4048,3579,4146,2767,3378 | ENSMUSG00000053654,"ENSMUSG00000053654",8,29,32,48,38,43,29,36,37,28,44,21,16,52,33,31,42,40,39,41 | ENSMUSG00000110387,"ENSMUSG00000110387",0,0,0,0,0,0,0,0,0,0,0,0,0,0,0,0,0,0,0,0 |
| ENSMUSG00000029632,"ENSMUSG00000029632",3078,4442,4888,5705,4983,3940,4857,3505,4470,5248,5613,5048,4251,4744,4952,5337,4254,5798,4810,4601 | ENSMUSG00000086847,"ENSMUSG00000086847",0,1,0,0,0,1,0,0,0,0,0,0,0,0,0,0,0,0,0,0 | ENSMUSG00000070551,"ENSMUSG00000070551",0,0,0,0,0,0,0,0,0,0,0,0,0,0,0,0,0,0,0,0 |
| ENSMUSG00000025613,"ENSMUSG00000025613",3840,3998,4905,5014,4465,4203,4649,3167,3641,4442,4884,3903,3815,4195,3781,4164,3831,4708,3248,3892 | ENSMUSG00000115326,"ENSMUSG00000115326",2,1,0,0,0,0,0,0,0,0,0,0,0,0,0,1,0,0,1,1 | ENSMUSG00000108986,"ENSMUSG00000108986",0,0,0,0,0,0,0,0,0,0,0,0,0,1,0,0,0,0,0,0 |
| ENSMUSG00000031489,"ENSMUSG00000031489",11,4,11,9,1,1,5,7,2,7,15,8,7,9,9,8,7,3,16,6 | ENSMUSG00000076476,"ENSMUSG00000076476",3,2,12,1,8,3,1,8,5,4,10,2,4,3,8,3,2,7,4,5 | ENSMUSG00000097682,"ENSMUSG00000097682",0,0,0,0,0,0,0,0,0,0,0,0,0,0,0,0,0,0,0,0 |
| ENSMUSG00000028581,"ENSMUSG00000028581",6055,6736,9869,6951,8670,9278,6053,7331,6701,8959,8385,5220,9979,8117,7463,8236,7811,9728,6013,7950 | ENSMUSG00000019785,"ENSMUSG00000019785",0,0,0,0,0,0,0,0,0,0,0,0,0,0,0,0,0,0,0,0 | ENSMUSG00000108629,"ENSMUSG00000108629",0,2,0,0,1,0,0,0,0,0,0,0,0,0,0,0,0,0,0,0 |
| ENSMUSG00000024588,"ENSMUSG00000024588",607,622,794,775,819,719,695,598,740,691,866,606,835,718,549,693,737,781,611,668 | ENSMUSG00000067106,"ENSMUSG00000067106",2,4,5,8,4,5,2,5,6,3,3,6,8,4,7,14,9,16,2,5 | ENSMUSG00000099558,"ENSMUSG00000099558",0,0,0,0,0,0,0,0,0,0,0,0,0,0,0,0,0,0,0,0 |
| ENSMUSG00000024899,"ENSMUSG00000024899",653,674,974,735,929,843,899,870,712,998,882,587,936,951,955,918,771,888,601,996 | ENSMUSG00000080746,"ENSMUSG00000080746",7,7,10,5,9,5,4,5,6,14,5,6,2,12,7,12,12,5,2,7 | ENSMUSG00000065729,"ENSMUSG00000065729",0,0,0,0,0,0,0,0,0,0,0,0,0,0,0,0,0,0,0,0 |
| ENSMUSG00000055435,"ENSMUSG00000055435",4757,5054,7396,6040,6805,6668,5080,5400,5368,6198,6984,4567,6823,6311,5940,6008,6035,6754,4701,5750 | ENSMUSG00000086241,"ENSMUSG00000086241",6,11,13,17,17,8,13,12,4,11,9,15,17,20,14,18,11,11,15,18 | ENSMUSG00000108970,"ENSMUSG00000108970",0,0,0,0,0,0,0,0,0,0,0,0,0,0,0,1,0,0,0,0 |
| ENSMUSG00000034422,"ENSMUSG00000034422",7955,7208,8150,9126,8702,8525,8219,7552,8275,8945,8326,6381,8270,8534,8334,11032,8490,11303,5628,7640 | ENSMUSG00000031952,"ENSMUSG00000031952",5,12,8,9,4,5,1,5,8,7,2,4,6,13,6,5,5,10,12,19 | ENSMUSG00000089111,"ENSMUSG00000089111",0,0,0,1,0,1,0,0,0,0,0,0,0,0,0,0,0,0,0,0 |
| ENSMUSG00000018965,"ENSMUSG00000018965",2016,2514,3468,2904,3157,2947,2862,2620,2667,2988,2813,2275,2919,2859,2685,3118,2558,3464,2656,3131 | ENSMUSG00000115793,"ENSMUSG00000115793",5,2,5,2,9,10,4,2,0,0,4,1,3,1,2,4,3,6,5,6 | ENSMUSG00000109106,"ENSMUSG00000109106",0,0,0,0,0,0,0,0,0,0,0,0,0,0,0,0,0,0,0,0 |
| ENSMUSG00000022425,"ENSMUSG00000022425",4559,4220,9325,6820,6715,7251,4379,4436,4529,5257,6865,4018,6851,5238,5049,5282,4798,5960,3490,4937 | ENSMUSG00000068617,"ENSMUSG00000068617",14,13,15,18,11,20,19,10,10,18,15,9,25,13,6,17,13,22,9,31 | ENSMUSG00000108993,"ENSMUSG00000108993",0,0,0,0,0,0,0,0,0,0,0,0,0,0,0,0,0,0,0,0 |
| ENSMUSG00000027282,"ENSMUSG00000027282",1528,1510,1829,1808,1765,1565,1689,1239,1483,1680,1754,1380,1596,1439,1420,1679,1425,1816,1385,1453 | ENSMUSG00000022652,"ENSMUSG00000022652",49,22,33,16,37,36,33,25,28,41,21,20,36,20,36,36,39,39,24,29 | ENSMUSG00000094780,"ENSMUSG00000094780",0,0,0,0,0,0,0,0,0,0,0,0,0,0,1,0,0,0,0,0 |
| ENSMUSG00000030754,"ENSMUSG00000030754",4696,3389,4064,3936,4099,3624,3617,2990,3388,3792,3910,3181,3303,3594,3344,3801,3381,4140,2631,3257 | ENSMUSG00000085403,"ENSMUSG00000085403",8,14,8,17,15,12,17,8,7,6,19,15,1,17,11,8,19,8,10,6 | ENSMUSG00000109037,"ENSMUSG00000109037",0,0,0,0,0,0,0,0,0,0,0,0,0,0,0,0,0,0,0,0 |
| ENSMUSG00000032479,"ENSMUSG00000032479",2003,2830,4165,3736,3710,3480,3449,3422,3335,3725,3637,2523,4307,3684,3501,3823,3661,4460,3027,3887 | ENSMUSG00000054966,"ENSMUSG00000054966",2,0,0,0,0,0,0,0,2,0,2,1,10,0,3,0,2,1,1,0 | ENSMUSG00000108786,"ENSMUSG00000108786",0,0,0,0,0,0,0,0,0,0,0,0,0,0,0,0,0,0,0,0 |
| ENSMUSG00000039367,"ENSMUSG00000039367",2543,3047,3743,3852,3768,3273,3724,3072,3555,3263,3907,2998,3459,3470,3434,3751,3103,4266,3276,3417 | ENSMUSG00000009471,"ENSMUSG00000009471",54,33,36,41,43,15,44,31,39,39,35,27,34,35,34,36,19,35,33,29 | ENSMUSG00000065698,"ENSMUSG00000065698",0,0,0,0,0,0,0,0,0,0,0,0,0,0,0,0,0,0,0,0 |
| ENSMUSG00000022555,"ENSMUSG00000022555",1330,1646,1900,1511,1823,1580,1940,1454,1572,1672,1563,1253,1423,1696,1505,1902,1439,1982,1425,1669 | ENSMUSG00000097644,"ENSMUSG00000097644",0,0,0,0,0,0,0,0,0,0,0,0,0,0,0,0,0,0,0,0 | ENSMUSG00000108965,"ENSMUSG00000108965",0,0,0,0,0,0,0,0,0,0,0,0,0,0,0,0,0,0,0,0 |
| ENSMUSG00000035697,"ENSMUSG00000035697",1504,2323,3580,2433,2922,3131,2368,2950,2583,2928,3370,1959,4269,2933,2697,3394,2887,3997,2805,3270 | ENSMUSG00000066270,"ENSMUSG00000066270",3,4,7,7,3,3,7,1,8,12,18,8,2,11,7,11,8,12,5,9 | ENSMUSG00000109906,"ENSMUSG00000109906",0,0,0,0,0,0,0,0,0,0,0,0,0,0,3,0,0,0,0,0 |
| ENSMUSG00000022403,"ENSMUSG00000022403",1664,1580,2062,1905,1767,1764,1632,1612,1654,1658,2002,1512,1879,1696,1785,1748,1629,2047,1595,1840 | ENSMUSG00000100775,"ENSMUSG00000100775",4,1,1,6,0,2,0,4,0,1,4,1,1,0,3,3,0,3,1,1 | ENSMUSG00000083216,"ENSMUSG00000083216",0,0,0,0,0,0,0,0,0,0,0,0,0,0,0,0,0,0,0,0 |
| ENSMUSG00000033327,"ENSMUSG00000033327",1229,1753,2307,2611,1977,2237,2206,1651,1642,2172,2274,1797,2280,2110,2016,1893,1858,2765,1704,2007 | ENSMUSG00000085084,"ENSMUSG00000085084",6,5,11,4,5,8,6,3,3,1,2,5,4,5,1,2,3,1,1,2 | ENSMUSG00000043073,"ENSMUSG00000043073",0,0,2,0,0,0,0,0,0,0,0,0,0,0,0,0,0,0,0,0 |
| ENSMUSG00000081684,"ENSMUSG00000081684",21,33,49,41,44,47,42,40,51,37,42,34,42,48,30,210,52,52,42,52 | ENSMUSG00000093793,"ENSMUSG00000093793",7,6,16,6,8,16,6,3,5,9,6,6,1,9,5,7,6,16,2,7 | ENSMUSG00000062369,"ENSMUSG00000062369",0,0,0,0,0,0,0,0,0,0,0,0,0,0,0,0,0,0,0,0 |
| ENSMUSG00000024811,"ENSMUSG00000024811",5208,3917,4598,4470,4752,4407,3943,3395,3926,4425,4245,3367,3748,4042,3886,4273,3843,4464,2863,3803 | ENSMUSG00000104872,"ENSMUSG00000104872",1,13,9,4,2,11,9,9,11,7,6,13,15,10,10,8,5,9,11,5 | ENSMUSG00000073915,"ENSMUSG00000073915",0,0,0,0,0,0,0,0,0,0,0,0,0,0,0,0,3,0,0,0 |
| ENSMUSG00000026970,"ENSMUSG00000026970",11836,11359,11924,13480,13615,11031,11952,8142,11719,13613,10654,10849,8278,12398,10675,13106,11644,12986,7284,10084 | ENSMUSG00000108543,"ENSMUSG00000108543",3,8,3,9,6,8,6,4,0,4,6,6,2,8,3,3,7,9,0,4 | ENSMUSG00000073913,"ENSMUSG00000073913",0,0,0,0,0,0,0,0,0,0,0,0,0,3,0,0,0,0,0,0 |
| ENSMUSG00000021427,"ENSMUSG00000021427",4591,4500,5372,5858,5835,5243,5100,3748,4575,5055,5691,4519,4374,4887,4537,5049,4337,5558,3549,4455 | ENSMUSG00000097718,"ENSMUSG00000097718",0,4,4,7,1,1,4,2,6,6,4,4,3,3,5,4,3,4,6,4 | ENSMUSG00000098945,"ENSMUSG00000098945",0,0,0,0,0,0,0,0,0,0,0,0,0,0,0,0,0,0,0,0 |
| ENSMUSG00000027227,"ENSMUSG00000027227",146,138,218,171,205,164,134,176,159,179,275,144,292,171,178,224,152,204,176,196 | ENSMUSG00000031893,"ENSMUSG00000031893",6,5,4,0,2,4,3,3,0,3,2,2,0,2,3,1,0,3,4,3 | ENSMUSG00000073900,"ENSMUSG00000073900",0,0,0,0,0,0,0,0,0,0,0,0,0,0,0,0,0,0,0,0 |
| ENSMUSG00000021457,"ENSMUSG00000021457",3001,5233,6274,4126,5804,6578,5007,4974,4776,7228,5034,3551,6564,6989,4923,6989,4870,7100,3550,5735 | ENSMUSG00000039313,"ENSMUSG00000039313",0,2,0,1,3,1,2,2,0,1,0,1,0,0,0,1,2,4,1,1 | ENSMUSG00000096151,"ENSMUSG00000096151",0,0,0,0,0,0,0,0,0,0,0,0,0,0,0,0,0,0,0,0 |
| ENSMUSG00000010097,"ENSMUSG00000010097",3123,3287,4112,4298,4475,3851,3961,3299,3753,4036,4120,3393,3843,3976,3873,4094,3629,4762,3572,3756 | ENSMUSG00000099364,"ENSMUSG00000099364",9,9,13,14,10,11,11,10,7,10,16,3,6,8,12,15,5,17,15,9 | ENSMUSG00000046431,"ENSMUSG00000046431",0,0,0,0,0,0,0,0,0,0,0,0,0,0,0,0,0,0,0,0 |
| ENSMUSG00000031812,"ENSMUSG00000031812",3345,3261,4280,4320,3508,3223,3285,2968,3268,3745,4036,3332,3795,3780,3285,3949,3285,3990,2951,3332 | ENSMUSG00000105708,"ENSMUSG00000105708",2,6,4,2,1,1,1,1,0,0,1,3,1,0,0,0,0,5,2,0 | ENSMUSG00000109611,"ENSMUSG00000109611",0,0,0,0,0,0,0,0,0,0,0,0,0,0,0,0,0,0,0,0 |
| ENSMUSG00000054263,"ENSMUSG00000054263",958,874,1137,1136,1080,1180,871,1104,979,1151,1317,830,1123,969,1056,1131,1077,1431,823,1051 | ENSMUSG00000084923,"ENSMUSG00000084923",0,0,0,0,0,0,0,0,1,0,0,0,0,0,0,0,0,0,0,0 | ENSMUSG00000101755,"ENSMUSG00000101755",0,0,0,0,0,0,0,0,0,0,0,0,0,0,0,0,0,0,0,0 |
| ENSMUSG00000026110,"ENSMUSG00000026110",743,731,810,660,893,940,716,713,749,962,756,547,905,852,665,1046,789,1040,538,881 | ENSMUSG00000085058,"ENSMUSG00000085058",8,7,19,11,7,14,6,5,6,5,12,15,4,15,10,8,9,12,11,4 | ENSMUSG00000108401,"ENSMUSG00000108401",0,0,0,0,0,0,0,0,0,0,0,0,0,0,0,0,0,0,0,0 |
| ENSMUSG00000005803,"ENSMUSG00000005803",299,363,485,406,451,461,405,415,351,442,369,293,461,427,402,442,450,620,400,456 | ENSMUSG00000111977,"ENSMUSG00000111977",6,11,16,11,11,7,9,10,10,10,12,5,28,5,7,5,9,14,5,10 | ENSMUSG00000084492,"ENSMUSG00000084492",0,1,0,0,0,0,0,0,0,0,0,0,0,0,0,0,0,0,1,0 |
| ENSMUSG00000021109,"ENSMUSG00000021109",13076,10561,10967,11725,13299,11538,10828,8730,10547,12790,9987,8763,8729,11621,9892,11685,10136,11441,7332,9766 | ENSMUSG00000076633,"ENSMUSG00000076633",0,0,0,0,0,0,0,0,0,0,0,0,0,0,0,0,0,0,0,0 | ENSMUSG00000109414,"ENSMUSG00000109414",0,0,0,0,0,0,0,0,0,0,0,0,0,0,0,0,0,0,0,0 |
| ENSMUSG00000023845,"ENSMUSG00000023845",2885,2454,2863,2392,2788,2860,2108,2301,2471,2903,2864,1950,2792,2405,2432,2685,2490,3136,1565,2274 | ENSMUSG00000101223,"ENSMUSG00000101223",2,15,9,11,12,16,11,22,7,9,13,14,11,9,8,10,8,13,13,18 | ENSMUSG00000098267,"ENSMUSG00000098267",0,0,0,0,0,1,0,0,0,0,0,0,0,0,0,0,0,0,0,0 |
| ENSMUSG00000024858,"ENSMUSG00000024858",2664,3702,4977,4190,4653,4257,4136,3750,4050,4357,4664,3371,4737,4367,3912,4701,4232,5118,3797,4181 | ENSMUSG00000078685,"ENSMUSG00000078685",0,0,0,0,0,2,0,0,0,0,0,0,0,0,0,0,0,0,0,0 | ENSMUSG00000064677,"ENSMUSG00000064677",0,0,0,0,0,0,0,0,0,0,0,0,0,0,0,0,0,0,0,0 |
| ENSMUSG00000027215,"ENSMUSG00000027215",473,960,1390,1125,1230,1133,1053,1177,874,1227,1161,812,1413,1201,1111,1366,1025,1600,1051,1286 | ENSMUSG00000049410,"ENSMUSG00000049410",2,11,6,7,24,10,8,10,14,6,13,11,26,17,6,20,8,20,17,16 | ENSMUSG00000109981,"ENSMUSG00000109981",0,0,0,0,0,0,0,0,0,0,0,0,0,0,0,0,0,0,0,0 |
| ENSMUSG00000000420,"ENSMUSG00000000420",3027,2632,3442,3527,3564,2848,2938,2245,2871,3161,3131,2770,2806,2924,2713,3147,2666,3332,1887,2619 | ENSMUSG00000022603,"ENSMUSG00000022603",10,1,5,12,6,5,5,6,13,4,9,11,1,12,5,4,3,5,3,7 | ENSMUSG00000110319,"ENSMUSG00000110319",0,0,0,0,0,0,0,0,0,0,0,0,0,0,0,0,0,0,0,0 |
| ENSMUSG00000024392,"ENSMUSG00000024392",1630,2470,3221,3128,2870,2706,2822,2397,2469,2823,3123,2308,2989,2874,2647,3022,2449,3455,2362,2711 | ENSMUSG00000056061,"ENSMUSG00000056061",0,0,0,0,0,0,0,0,0,0,0,0,0,0,0,0,0,0,0,0 | ENSMUSG00000051180,"ENSMUSG00000051180",0,0,0,0,0,0,0,0,0,0,0,0,0,0,0,0,0,0,0,0 |
| ENSMUSG00000025510,"ENSMUSG00000025510",1671,2123,2803,2911,2649,2174,2461,1986,2144,2399,2820,2211,2417,2551,2404,2447,2191,2747,2347,2323 | ENSMUSG00000062758,"ENSMUSG00000062758",4,8,12,15,9,5,9,5,9,10,13,5,12,19,5,15,14,13,8,12 | ENSMUSG00000108934,"ENSMUSG00000108934",0,0,0,0,0,0,0,0,1,0,0,0,0,0,0,0,0,0,0,0 |
| ENSMUSG00000026421,"ENSMUSG00000026421",1742,2085,2856,3334,2688,2403,2304,2918,2606,2301,2799,2230,2812,2788,2661,3659,2414,3768,2784,3253 | ENSMUSG00000073601,"ENSMUSG00000073601",62,23,37,35,38,48,20,26,44,34,20,17,34,35,27,59,17,41,49,24 | ENSMUSG00000093221,"ENSMUSG00000093221",0,0,0,0,0,0,0,0,0,0,0,0,0,0,0,0,0,0,0,0 |
| ENSMUSG00000026034,"ENSMUSG00000026034",3545,3019,3624,3216,3673,3413,3046,2557,2984,3532,3819,2912,3337,3166,2885,3513,2853,3954,2236,2557 | ENSMUSG00000083087,"ENSMUSG00000083087",2,18,17,23,9,16,9,7,18,15,18,21,16,12,11,26,9,20,11,15 | ENSMUSG00000111890,"ENSMUSG00000111890",0,0,0,0,0,0,0,0,0,3,0,0,0,0,0,0,0,0,0,0 |
| ENSMUSG00000040322,"ENSMUSG00000040322",2080,1631,1800,1632,1754,1755,1721,1155,1612,1867,1572,1310,1340,1634,1435,1735,1574,1768,1016,1510 | ENSMUSG00000106630,"ENSMUSG00000106630",0,0,0,0,0,0,0,0,0,0,0,0,0,0,0,0,0,0,0,0 | ENSMUSG00000094906,"ENSMUSG00000094906",0,0,0,0,0,0,0,0,0,0,0,0,0,0,0,0,0,0,0,0 |
| ENSMUSG00000028393,"ENSMUSG00000028393",315,341,425,421,407,438,377,299,307,324,477,326,436,361,347,284,345,406,394,344 | ENSMUSG00000106133,"ENSMUSG00000106133",5,8,11,8,3,5,15,4,7,7,5,11,2,7,10,10,10,7,3,15 | ENSMUSG00000098065,"ENSMUSG00000098065",0,0,0,0,0,0,0,0,1,0,1,0,0,0,0,0,0,0,0,0 |
| ENSMUSG00000027177,"ENSMUSG00000027177",3531,3260,3632,3726,3953,3300,3304,2547,3211,3734,3708,3046,3067,3369,2765,3455,3114,3710,2288,2681 | ENSMUSG00000071568,"ENSMUSG00000071568",2,1,1,0,0,0,0,0,0,0,0,0,0,0,1,0,0,0,3,0 | ENSMUSG00000065096,"ENSMUSG00000065096",0,0,0,0,0,0,0,0,0,0,0,0,0,0,0,0,0,0,0,0 |
| ENSMUSG00000047866,"ENSMUSG00000047866",615,695,863,858,784,752,736,759,716,768,835,580,811,839,751,804,774,898,626,754 | ENSMUSG00000109096,"ENSMUSG00000109096",11,2,12,10,8,21,8,8,9,10,12,4,7,9,12,5,10,20,11,5 | ENSMUSG00000094977,"ENSMUSG00000094977",0,0,0,0,0,0,1,0,1,0,0,0,0,0,1,0,0,0,0,0 |
| ENSMUSG00000026509,"ENSMUSG00000026509",5305,6371,7477,7480,7837,6782,7918,5174,6635,7356,6509,5980,5348,6972,6430,7164,6460,7964,5046,6241 | ENSMUSG00000041165,"ENSMUSG00000041165",3,2,0,4,3,0,0,2,4,0,4,0,3,5,1,4,1,1,0,0 | ENSMUSG00000099749,"ENSMUSG00000099749",0,0,0,0,0,0,0,0,0,0,0,0,0,0,1,0,0,0,0,0 |
| ENSMUSG00000054387,"ENSMUSG00000054387",2515,2442,2736,2869,2961,2923,2394,2307,2542,2657,3023,2255,2813,2518,2515,2920,2461,2984,2006,2406 | ENSMUSG00000082321,"ENSMUSG00000082321",4,8,11,7,14,11,6,5,4,9,3,13,2,4,3,10,3,4,3,11 | ENSMUSG00000069707,"ENSMUSG00000069707",0,0,0,0,0,0,0,0,0,0,0,0,0,0,0,0,0,0,0,0 |
| ENSMUSG00000024026,"ENSMUSG00000024026",4257,3845,4878,5618,4805,3925,4219,3698,3955,4316,4659,4464,3710,4271,4196,4946,3824,5235,3837,4080 | ENSMUSG00000106375,"ENSMUSG00000106375",10,8,3,3,11,9,12,8,21,23,11,6,7,10,10,15,13,21,3,6 | ENSMUSG00000039098,"ENSMUSG00000039098",0,0,0,0,0,0,0,0,0,0,0,0,0,0,0,0,0,0,0,0 |
| ENSMUSG00000066551,"ENSMUSG00000066551",1663,2401,3127,2420,2485,2499,2316,2043,2207,2681,2709,2032,2626,2342,2240,2509,2096,2926,2264,2417 | ENSMUSG00000022342,"ENSMUSG00000022342",38,24,37,37,21,15,14,27,35,17,54,33,17,22,17,41,20,37,26,24 | ENSMUSG00000112472,"ENSMUSG00000112472",0,0,0,0,0,0,0,0,0,0,0,0,0,0,0,0,0,0,0,0 |
| ENSMUSG00000000275,"ENSMUSG00000000275",3665,3445,4123,3955,3887,4350,4027,3865,4097,4605,4137,2908,4369,4216,3839,4938,3690,5083,3148,4323 | ENSMUSG00000115044,"ENSMUSG00000115044",2,2,8,5,5,10,2,8,3,4,9,5,4,9,2,6,6,17,2,12 | ENSMUSG00000110758,"ENSMUSG00000110758",0,0,0,0,0,0,0,0,0,0,0,0,0,0,0,0,0,0,0,2 |
| ENSMUSG00000015714,"ENSMUSG00000015714",1747,2243,2779,2667,2647,2695,2242,2078,2250,2662,2392,1688,2235,2701,2238,2488,2273,2752,1994,2217 | ENSMUSG00000087397,"ENSMUSG00000087397",1,1,3,4,6,2,8,3,3,2,1,3,4,5,7,8,7,2,1,5 | ENSMUSG00000110822,"ENSMUSG00000110822",0,0,0,0,0,0,0,0,0,0,0,0,1,0,0,0,0,0,0,0 |
| ENSMUSG00000025027,"ENSMUSG00000025027",1341,1912,2607,2657,2444,2156,2202,1891,1902,2007,2518,2001,2262,2204,2223,2460,1931,2607,1915,2309 | ENSMUSG00000112599,"ENSMUSG00000112599",5,11,20,7,14,21,7,15,22,22,13,12,26,13,25,15,11,21,6,23 | ENSMUSG00000088165,"ENSMUSG00000088165",0,0,0,1,0,0,0,0,0,0,0,0,0,1,0,0,0,0,0,0 |
| ENSMUSG00000022464,"ENSMUSG00000022464",51,43,42,29,29,133,68,78,49,37,42,28,63,44,62,57,58,59,28,62 | ENSMUSG00000035580,"ENSMUSG00000035580",0,1,0,1,0,0,0,0,0,0,0,0,0,3,0,0,0,0,0,0 | ENSMUSG00000111494,"ENSMUSG00000111494",1,0,0,0,0,0,1,0,0,0,0,0,0,0,0,0,0,0,0,0 |
| ENSMUSG00000058799,"ENSMUSG00000058799",2309,2249,2736,2517,2489,2842,2614,1961,2142,2508,2786,2101,2364,2398,2301,2562,2226,2967,1990,2471 | ENSMUSG00000103662,"ENSMUSG00000103662",1,5,4,7,0,4,4,1,3,2,1,4,17,1,4,2,3,12,4,4 | ENSMUSG00000111436,"ENSMUSG00000111436",0,0,0,0,0,0,0,0,0,0,0,0,0,0,0,0,0,0,0,0 |
| ENSMUSG00000054766,"ENSMUSG00000054766",3329,3810,4703,4861,4267,3840,3839,3009,3551,4030,4752,3742,3524,3754,3778,4107,3512,4593,3385,3882 | ENSMUSG00000073094,"ENSMUSG00000073094",0,0,0,0,0,0,0,0,0,1,0,0,0,0,0,0,0,0,0,0 | ENSMUSG00000111374,"ENSMUSG00000111374",0,0,0,0,0,0,1,0,0,0,0,0,0,0,0,0,0,0,0,0 |
| ENSMUSG00000023951,"ENSMUSG00000023951",1336,1892,2806,2479,3540,2245,1985,3489,2439,1778,2590,2048,3184,2479,2547,3565,2305,3804,2841,2747 | ENSMUSG00000106691,"ENSMUSG00000106691",2,0,11,2,6,6,10,1,0,2,6,1,7,5,2,3,1,9,3,3 | ENSMUSG00000111935,"ENSMUSG00000111935",0,0,0,0,0,0,0,0,0,0,0,0,0,0,0,0,0,0,0,0 |
| ENSMUSG00000027204,"ENSMUSG00000027204",4929,5466,7788,7032,6191,10016,6626,6371,5457,5925,7091,4648,8059,5862,5739,5714,5962,7482,4647,6160 | ENSMUSG00000097527,"ENSMUSG00000097527",2,5,4,4,2,2,6,7,1,10,3,1,6,6,2,3,1,5,4,0 | ENSMUSG00000112337,"ENSMUSG00000112337",0,0,0,0,0,0,0,0,0,0,0,0,0,0,0,0,0,0,0,0 |
| ENSMUSG00000022994,"ENSMUSG00000022994",260,405,530,517,478,544,424,438,473,468,554,344,643,466,478,470,397,529,455,451 | ENSMUSG00000110125,"ENSMUSG00000110125",0,2,0,2,0,0,1,0,2,0,1,0,2,0,0,1,1,0,0,0 | ENSMUSG00000092914,"ENSMUSG00000092914",0,0,1,0,0,0,0,1,0,0,0,0,0,0,0,0,0,0,0,1 |
| ENSMUSG00000024404,"ENSMUSG00000024404",5523,4902,5399,5559,5571,5211,5170,3634,4842,5914,5182,4426,4272,5157,4452,5599,4469,5399,3494,4297 | ENSMUSG00000085440,"ENSMUSG00000085440",2,0,4,2,0,0,0,2,2,3,6,1,0,3,1,2,1,4,3,3 | ENSMUSG00000112552,"ENSMUSG00000112552",0,0,0,0,0,0,0,0,0,0,0,0,0,0,0,0,0,0,0,0 |
| ENSMUSG00000020611,"ENSMUSG00000020611",5597,4853,5240,5179,5682,5291,4772,4767,5167,6220,5131,4128,5335,5731,4798,6095,5605,6459,3431,4905 | ENSMUSG00000069273,"ENSMUSG00000069273",0,9,9,4,5,5,1,1,6,8,5,8,7,8,7,6,2,10,0,4 | ENSMUSG00000112576,"ENSMUSG00000112576",0,0,0,0,0,0,0,0,0,0,0,0,0,0,0,0,0,1,0,0 |
| ENSMUSG00000040725,"ENSMUSG00000040725",1677,3208,4344,4230,3833,3695,3638,3215,3408,3720,4058,3275,3821,3707,3577,4230,3569,4597,3088,3788 | ENSMUSG00000111971,"ENSMUSG00000111971",15,10,13,15,16,10,13,6,24,30,10,17,6,17,12,14,10,21,12,11 | ENSMUSG00000112018,"ENSMUSG00000112018",0,0,0,0,0,0,0,0,0,0,0,0,0,0,0,0,0,0,0,0 |
| ENSMUSG00000038976,"ENSMUSG00000038976",1623,3584,5028,4504,4395,4214,3701,3360,3587,4055,4518,3459,4529,4212,3936,4666,4099,5096,3644,3760 | ENSMUSG00000104018,"ENSMUSG00000104018",12,8,8,3,3,3,2,7,5,8,6,7,5,12,12,4,8,5,3,5 | ENSMUSG00000112378,"ENSMUSG00000112378",0,0,0,0,0,0,0,0,0,0,0,0,0,0,0,0,0,0,0,0 |
| ENSMUSG00000003746,"ENSMUSG00000003746",2437,2405,3759,2908,2975,3270,2581,2514,2498,3267,2939,1877,3066,2988,2701,2663,2827,2963,1978,2476 | ENSMUSG00000053166,"ENSMUSG00000053166",5,11,6,9,14,15,10,11,8,5,11,7,17,10,18,14,18,12,18,17 | ENSMUSG00000111908,"ENSMUSG00000111908",0,0,0,0,0,0,0,0,0,0,0,0,0,0,0,0,0,0,0,0 |
| ENSMUSG00000020859,"ENSMUSG00000020859",5478,4725,5666,5646,5694,5281,5063,4441,5029,5856,5173,4271,5204,5538,4957,5633,4997,5967,3463,4815 | ENSMUSG00000094983,"ENSMUSG00000094983",0,1,0,1,0,1,0,0,0,0,1,0,0,4,0,0,2,0,1,0 | ENSMUSG00000112434,"ENSMUSG00000112434",0,0,2,0,0,0,0,0,1,0,0,0,0,0,0,0,0,0,0,0 |
| ENSMUSG00000020664,"ENSMUSG00000020664",2377,1852,2238,2131,2086,1920,1989,1585,1842,2218,2140,1763,1848,2060,1666,2044,1817,2180,1555,1819 | ENSMUSG00000096924,"ENSMUSG00000096924",8,10,17,9,4,13,7,4,8,12,7,7,11,8,10,11,12,10,5,2 | ENSMUSG00000112333,"ENSMUSG00000112333",0,0,0,0,0,0,0,0,0,0,0,0,0,0,0,0,0,0,0,0 |
| ENSMUSG00000003660,"ENSMUSG00000003660",2355,2544,3337,3198,3088,2944,3047,2531,2698,2813,3170,2307,2868,2925,2671,3165,2747,3573,2359,2772 | ENSMUSG00000003227,"ENSMUSG00000003227",8,4,4,6,1,10,11,6,7,6,9,18,7,5,6,8,4,14,12,7 | ENSMUSG00000090212,"ENSMUSG00000090212",0,0,0,0,0,0,0,1,0,0,0,0,0,0,0,0,0,0,0,0 |
| ENSMUSG00000020051,"ENSMUSG00000020051",3,0,4,0,0,0,0,2,0,0,0,0,0,1,0,2,1,1,5,2 | ENSMUSG00000090610,"ENSMUSG00000090610",8,2,6,8,7,4,2,4,8,6,7,7,9,13,5,2,2,7,2,3 | ENSMUSG00000112608,"ENSMUSG00000112608",1,0,0,0,0,0,0,0,0,0,0,0,0,0,0,0,0,0,0,0 |
| ENSMUSG00000071713,"ENSMUSG00000071713",9316,13966,15466,13161,15340,17515,14933,11474,12335,17789,12857,8774,14975,17152,12437,16484,13326,16472,9201,12396 | ENSMUSG00000032530,"ENSMUSG00000032530",6,13,13,4,9,6,8,7,4,14,6,2,3,7,8,9,7,10,7,1 | ENSMUSG00000112414,"ENSMUSG00000112414",0,0,0,0,0,0,0,0,0,0,0,0,0,0,0,0,0,0,0,0 |
| ENSMUSG00000022500,"ENSMUSG00000022500",7411,7930,8941,9237,9172,8736,9388,8060,8560,10147,8035,6657,8238,9260,7882,10042,8195,9802,6399,8757 | ENSMUSG00000076755,"ENSMUSG00000076755",0,0,1,2,0,3,0,1,0,0,2,0,1,0,0,0,1,0,0,3 | ENSMUSG00000112241,"ENSMUSG00000112241",0,1,0,0,2,0,0,0,0,0,0,0,0,0,0,0,0,0,0,0 |
| ENSMUSG00000031422,"ENSMUSG00000031422",4236,4462,5694,5301,5309,5055,4928,4058,4388,5292,5428,4181,4697,4897,4643,5094,4459,5672,3972,4789 | ENSMUSG00000094251,"ENSMUSG00000094251",3,5,8,9,14,14,9,16,6,2,15,3,7,3,7,15,6,8,13,5 | ENSMUSG00000095721,"ENSMUSG00000095721",0,1,0,0,0,2,0,0,0,0,0,0,0,0,0,0,0,0,0,0 |
| ENSMUSG00000032042,"ENSMUSG00000032042",2721,2858,3869,3927,3467,3473,3494,2738,3196,3287,3591,2694,2995,3469,3103,3526,2934,3872,2882,3129 | ENSMUSG00000093656,"ENSMUSG00000093656",1,3,2,6,5,2,1,4,5,4,10,1,11,2,4,4,4,7,6,3 | ENSMUSG00000112051,"ENSMUSG00000112051",2,0,0,0,0,0,0,0,0,0,0,0,0,0,0,0,0,0,0,0 |
| ENSMUSG00000004056,"ENSMUSG00000004056",1942,2564,3369,3164,3234,2832,3063,2470,2579,3038,3051,2345,2798,3015,2643,2983,2647,3450,2537,2766 | ENSMUSG00000108484,"ENSMUSG00000108484",11,10,9,14,5,10,12,7,11,8,14,14,11,5,8,6,9,6,2,14 | ENSMUSG00000112735,"ENSMUSG00000112735",0,0,0,0,0,0,0,0,0,0,0,0,0,0,0,0,0,0,0,0 |
| ENSMUSG00000036309,"ENSMUSG00000036309",3371,2686,2997,3632,2770,2568,2601,1937,2349,2741,3166,2772,2254,2400,2258,2701,2104,2825,1904,2229 | ENSMUSG00000085663,"ENSMUSG00000085663",9,17,9,7,13,5,2,10,7,8,15,6,8,12,7,6,6,8,7,9 | ENSMUSG00000112183,"ENSMUSG00000112183",0,0,0,0,0,0,1,0,0,0,0,0,0,0,0,0,0,0,0,0 |
| ENSMUSG00000068039,"ENSMUSG00000068039",4060,3229,3851,3837,3615,3614,3565,2512,2945,3540,3811,3028,2957,3315,2997,3308,2892,3651,2456,3105 | ENSMUSG00000050074,"ENSMUSG00000050074",0,1,2,1,0,0,1,0,4,1,3,0,1,4,0,3,0,2,1,3 | ENSMUSG00000112725,"ENSMUSG00000112725",0,0,0,0,1,0,0,0,0,0,0,0,0,0,0,0,0,0,0,0 |
| ENSMUSG00000002015,"ENSMUSG00000002015",1349,1465,1835,1784,1762,1538,1678,1387,1443,1610,1730,1392,1750,1601,1456,1647,1380,1950,1296,1611 | ENSMUSG00000115961,"ENSMUSG00000115961",44,26,25,18,30,36,20,28,15,32,17,16,32,32,36,26,16,26,14,36 | ENSMUSG00000112025,"ENSMUSG00000112025",0,0,0,1,0,0,0,0,0,0,0,0,0,0,0,0,2,0,0,0 |
| ENSMUSG00000002997,"ENSMUSG00000002997",280,237,342,285,330,268,222,236,276,239,357,208,354,258,243,216,207,271,219,259 | ENSMUSG00000039154,"ENSMUSG00000039154",10,2,3,3,3,5,9,0,6,5,4,3,3,4,6,3,4,5,4,3 | ENSMUSG00000094523,"ENSMUSG00000094523",0,0,0,0,0,0,0,0,0,0,0,0,1,0,0,0,0,0,0,0 |
| ENSMUSG00000024870,"ENSMUSG00000024870",1392,2362,3450,2937,3066,2739,2493,2319,2271,2747,2975,2324,2998,2685,2645,3111,2464,3474,2609,2817 | ENSMUSG00000097888,"ENSMUSG00000097888",9,13,11,14,4,16,21,4,15,14,13,13,16,8,9,14,16,19,9,10 | ENSMUSG00000112503,"ENSMUSG00000112503",0,0,0,0,0,0,0,0,1,0,0,0,0,0,0,0,0,0,0,0 |
| ENSMUSG00000005198,"ENSMUSG00000005198",2706,3484,4439,3655,4491,4328,3931,3220,3953,4546,4219,3122,3913,4170,3604,4317,3625,4644,2967,3574 | ENSMUSG00000041878,"ENSMUSG00000041878",5,5,1,3,9,5,1,4,5,3,3,4,4,2,0,4,1,4,3,4 | ENSMUSG00000113032,"ENSMUSG00000113032",0,0,0,0,0,0,0,0,0,0,0,0,0,0,0,0,0,0,0,0 |
| ENSMUSG00000070738,"ENSMUSG00000070738",895,931,1197,1007,1176,1049,1054,940,1047,1237,1262,796,1130,1202,938,1174,976,1342,849,1183 | ENSMUSG00000108170,"ENSMUSG00000108170",3,0,8,1,2,3,1,1,1,0,0,1,3,5,1,5,1,15,0,2 | ENSMUSG00000112304,"ENSMUSG00000112304",0,0,0,0,0,0,0,0,0,0,0,0,0,1,0,0,0,0,0,0 |
| ENSMUSG00000006676,"ENSMUSG00000006676",2541,2851,3453,3704,3244,2926,3385,2854,3093,3183,3526,2840,3275,3387,3110,3477,2824,3694,3053,3189 | ENSMUSG00000107928,"ENSMUSG00000107928",2,6,10,14,6,5,14,9,15,12,12,5,7,14,6,13,8,8,10,6 | ENSMUSG00000112845,"ENSMUSG00000112845",0,0,0,0,0,0,0,0,0,0,0,0,0,0,0,0,0,0,0,0 |
| ENSMUSG00000024587,"ENSMUSG00000024587",2446,2916,3736,3937,3211,3078,3425,2608,2961,3247,3721,2871,3264,3297,3159,3536,2847,3712,2664,3041 | ENSMUSG00000112831,"ENSMUSG00000112831",0,0,0,0,0,0,0,0,0,0,0,0,0,1,0,0,0,0,0,0 | ENSMUSG00000112176,"ENSMUSG00000112176",0,0,0,0,0,0,0,0,0,0,0,0,0,0,0,0,0,0,0,0 |
| ENSMUSG00000029840,"ENSMUSG00000029840",3263,2897,3584,3387,3474,3498,3239,2663,2834,3297,3437,2654,2969,3030,2902,3335,3111,3683,2363,3040 | ENSMUSG00000097706,"ENSMUSG00000097706",1,0,3,3,0,1,1,1,0,4,1,2,0,5,2,3,1,4,0,4 | ENSMUSG00000111910,"ENSMUSG00000111910",0,0,0,0,0,0,0,0,0,0,0,0,0,0,0,0,0,0,0,0 |
| ENSMUSG00000024695,"ENSMUSG00000024695",2951,3018,3395,3879,3561,3135,3055,2381,2945,3410,3504,3128,2690,2927,2718,3330,3040,3586,2244,2724 | ENSMUSG00000097903,"ENSMUSG00000097903",20,7,11,4,14,15,6,9,11,24,11,6,15,15,7,16,4,11,8,11 | ENSMUSG00000112260,"ENSMUSG00000112260",0,0,0,0,0,0,0,0,0,0,0,0,0,0,0,0,0,0,0,0 |
| ENSMUSG00000029328,"ENSMUSG00000029328",2657,3787,3917,4563,4042,3429,3502,2810,3588,4056,4334,3837,3348,3901,3228,3655,3452,4218,3047,3351 | ENSMUSG00000111546,"ENSMUSG00000111546",24,28,34,27,47,29,22,33,19,37,37,16,29,24,50,35,35,27,29,34 | ENSMUSG00000112357,"ENSMUSG00000112357",0,0,0,0,0,0,0,0,0,0,0,2,0,0,0,0,0,0,0,0 |
| ENSMUSG00000032786,"ENSMUSG00000032786",1183,1274,1529,1429,1655,1458,1644,1610,1727,1505,1385,993,1771,1610,1399,1787,1388,1852,1621,2029 | ENSMUSG00000048978,"ENSMUSG00000048978",0,0,0,0,0,0,0,0,0,0,1,0,0,0,0,0,0,0,2,0 | ENSMUSG00000089667,"ENSMUSG00000089667",0,0,0,0,0,0,0,0,0,0,0,0,0,0,0,0,0,0,0,0 |
| ENSMUSG00000030357,"ENSMUSG00000030357",584,1070,1437,1554,1173,1148,1307,966,1029,1134,1593,1089,1285,1272,1202,1197,1103,1434,1393,1393 | ENSMUSG00000113263,"ENSMUSG00000113263",1,8,7,7,8,11,5,12,10,7,6,5,10,8,8,8,3,5,4,10 | ENSMUSG00000112470,"ENSMUSG00000112470",0,0,0,0,0,0,0,0,0,0,0,0,0,0,0,0,0,0,0,0 |
| ENSMUSG00000024140,"ENSMUSG00000024140",1972,2190,3493,2484,2749,3569,2785,2229,2163,3047,2526,1542,2550,2802,2362,2566,2338,3062,2030,2342 | ENSMUSG00000027434,"ENSMUSG00000027434",0,0,0,1,1,0,0,0,0,0,0,0,0,0,0,0,0,0,1,0 | ENSMUSG00000112322,"ENSMUSG00000112322",0,0,0,0,0,0,0,0,0,0,0,0,0,0,0,0,0,0,0,0 |
| ENSMUSG00000032484,"ENSMUSG00000032484",77,66,83,44,22,154,119,89,39,111,71,13,52,120,72,69,104,136,4,68 | ENSMUSG00000073293,"ENSMUSG00000073293",8,4,5,2,3,3,6,4,7,7,6,0,1,2,4,2,1,3,3,5 | ENSMUSG00000112780,"ENSMUSG00000112780",0,0,0,0,0,0,0,0,0,0,0,0,0,1,0,0,1,0,0,0 |
| ENSMUSG00000024378,"ENSMUSG00000024378",817,665,917,973,1022,804,648,710,783,704,1136,984,1080,816,820,910,772,983,739,873 | ENSMUSG00000108239,"ENSMUSG00000108239",1,5,1,3,2,0,9,2,1,0,3,5,4,0,8,3,5,6,5,2 | ENSMUSG00000112415,"ENSMUSG00000112415",0,0,0,0,0,0,0,0,0,0,0,0,0,0,0,0,0,0,0,0 |
| ENSMUSG00000020671,"ENSMUSG00000020671",4887,3949,4709,5433,5118,4169,4368,3576,4582,4620,4652,4402,4029,4151,4092,4955,3964,5386,3524,4191 | ENSMUSG00000113998,"ENSMUSG00000113998",36,18,33,17,40,29,24,11,19,46,25,26,26,26,26,37,23,42,13,6 | ENSMUSG00000109803,"ENSMUSG00000109803",0,0,0,0,0,0,0,0,0,0,0,0,0,0,0,0,0,0,0,0 |
| ENSMUSG00000067851,"ENSMUSG00000067851",2827,2451,2599,2800,2914,2499,2438,2008,2365,2696,2714,2160,2357,2434,2382,2734,2455,3032,1683,2261 | ENSMUSG00000049605,"ENSMUSG00000049605",0,0,0,0,0,0,0,0,0,0,0,0,0,0,0,0,0,0,0,0 | ENSMUSG00000088687,"ENSMUSG00000088687",0,0,0,0,0,0,0,0,0,0,0,0,0,0,0,0,0,0,0,0 |
| ENSMUSG00000030120,"ENSMUSG00000030120",1113,2125,3016,2627,2403,2475,2356,2066,2164,2382,2707,2132,2755,2418,2341,2661,2320,3087,2356,2562 | ENSMUSG00000085761,"ENSMUSG00000085761",5,12,8,13,13,4,12,12,11,9,10,17,7,9,13,12,3,13,13,15 | ENSMUSG00000059134,"ENSMUSG00000059134",0,0,0,0,0,0,0,0,0,0,0,0,0,0,0,0,0,0,0,0 |
| ENSMUSG00000018677,"ENSMUSG00000018677",804,1087,1578,1296,1321,1215,1217,1044,1106,1173,1296,1051,1253,1198,1049,1349,1042,1550,1195,1250 | ENSMUSG00000109784,"ENSMUSG00000109784",14,8,11,14,17,9,10,8,9,3,10,13,12,4,2,4,5,14,10,10 | ENSMUSG00000058084,"ENSMUSG00000058084",0,0,0,0,0,0,0,0,0,0,0,0,0,0,0,0,0,0,0,0 |
| ENSMUSG00000025228,"ENSMUSG00000025228",2766,3561,4849,4555,4331,3846,4091,3508,3835,3901,4547,3505,4154,4036,3803,4571,3616,5051,3664,4398 | ENSMUSG00000110459,"ENSMUSG00000110459",1,0,0,3,2,1,1,4,2,0,4,0,0,0,0,0,0,2,0,3 | ENSMUSG00000091511,"ENSMUSG00000091511",0,0,0,0,0,0,0,0,0,0,0,0,0,0,0,0,0,0,0,0 |

| ENSMUSG00000035441,"ENSMUSG00000035441",609,754,718,807,848,849,914,657,798,741,653,659,786,816,740,849,765,905,557,829 | ENSMUSG00000001497,"ENSMUSG00000001497",13,6,12,8,7,11,7,8,10,11,1,10,17,8,14,9,19,21,10,14 | ENSMUSG00000108848,"ENSMUSG00000108848",0,0,0,0,0,0,0,0,0,0,0,0,0,0,0,0,0,0,0,0 |
| --- | --- | --- |
| ENSMUSG00000025935,"ENSMUSG00000025935",4190,3172,4207,3863,4275,3792,3521,3106,3577,3786,4072,3219,3593,3662,3405,3973,3390,4187,2395,3582 | ENSMUSG00000090021,"ENSMUSG00000090021",9,7,10,2,6,12,5,4,5,8,2,7,15,7,6,3,6,4,3,2 | ENSMUSG00000109162,"ENSMUSG00000109162",0,0,0,0,0,0,0,0,0,0,0,0,0,0,0,0,0,0,0,0 |
| ENSMUSG00000022895,"ENSMUSG00000022895",6018,7720,7922,6672,7577,8072,7891,7580,8672,9776,6626,5399,7927,9913,6928,9463,7403,9104,5202,8236 | ENSMUSG00000100615,"ENSMUSG00000100615",0,1,2,10,5,2,5,10,2,8,4,2,9,5,3,9,7,5,4,4 | ENSMUSG00000093319,"ENSMUSG00000093319",0,0,0,0,0,0,0,0,0,0,0,0,0,0,0,0,0,0,0,0 |
| ENSMUSG00000026185,"ENSMUSG00000026185",4668,6120,9191,7378,8868,7521,6762,6485,6885,6333,7398,5163,10592,5693,5787,6134,6590,7764,6626,7487 | ENSMUSG00000082826,"ENSMUSG00000082826",23,33,17,52,39,28,32,33,44,43,35,37,27,33,26,41,31,43,20,38 | ENSMUSG00000074473,"ENSMUSG00000074473",0,0,0,0,0,0,0,0,0,0,0,0,0,0,0,0,0,0,0,0 |
| ENSMUSG00000041737,"ENSMUSG00000041737",156,179,279,77,230,222,153,230,204,187,135,133,203,179,126,340,136,446,247,303 | ENSMUSG00000109781,"ENSMUSG00000109781",9,2,11,7,3,12,6,8,16,7,12,9,3,12,6,7,4,6,8,12 | ENSMUSG00000101899,"ENSMUSG00000101899",0,0,0,0,0,0,0,0,0,0,0,0,0,0,0,0,0,0,0,0 |
| ENSMUSG00000007458,"ENSMUSG00000007458",2771,3118,4027,3720,3576,3245,3072,3060,3137,3566,3827,2885,3672,3475,3122,3793,3412,4210,2976,3478 | ENSMUSG00000025588,"ENSMUSG00000025588",0,0,0,0,0,0,1,0,0,2,0,0,0,0,0,4,0,0,0,0 | ENSMUSG00000088641,"ENSMUSG00000088641",0,0,0,0,0,0,0,0,0,0,0,0,0,0,1,0,0,0,0,0 |
| ENSMUSG00000029372,"ENSMUSG00000029372",135,80,118,75,89,114,85,109,126,133,119,39,125,79,94,91,90,56,57,106 | ENSMUSG00000103579,"ENSMUSG00000103579",29,19,34,34,41,31,28,18,20,22,48,20,23,31,27,36,24,49,34,24 | ENSMUSG00000104767,"ENSMUSG00000104767",0,0,0,0,0,0,0,0,0,0,0,0,0,0,0,0,0,0,0,0 |
| ENSMUSG00000020572,"ENSMUSG00000020572",7494,6324,6664,7485,7760,7328,6701,6475,6237,7973,6639,5427,6766,7295,6574,8199,6415,8984,3900,6838 | ENSMUSG00000059864,"ENSMUSG00000059864",14,16,16,6,28,26,34,20,9,35,18,11,10,36,19,50,17,32,9,16 | ENSMUSG00000087892,"ENSMUSG00000087892",0,0,0,0,0,0,0,0,0,0,0,0,0,0,0,0,0,0,0,0 |
| ENSMUSG00000020198,"ENSMUSG00000020198",1912,2544,3250,3242,3247,2844,2927,2694,2837,2805,3221,2325,3131,3086,2716,3134,2755,3232,2768,3031 | ENSMUSG00000091742,"ENSMUSG00000091742",0,2,1,2,2,2,1,4,2,3,4,2,2,3,1,4,1,4,0,0 | ENSMUSG00000110056,"ENSMUSG00000110056",0,0,0,0,0,0,0,0,0,0,0,0,0,0,0,0,0,0,0,0 |
| ENSMUSG00000024900,"ENSMUSG00000024900",1117,1419,2054,1556,1411,1588,1711,1529,1555,1696,1896,1056,1455,1504,1249,1588,1464,1935,890,1195 | ENSMUSG00000068855,"ENSMUSG00000068855",0,1,1,1,1,1,1,0,2,4,0,4,2,8,0,1,6,0,1,1 | ENSMUSG00000096265,"ENSMUSG00000096265",0,0,0,0,0,0,0,0,0,0,0,0,0,1,0,0,0,0,0,0 |
| ENSMUSG00000050953,"ENSMUSG00000050953",11279,11261,14042,13378,14754,15207,14150,10540,12414,14156,14458,9815,11938,14928,11497,12076,11541,12606,8320,10975 | ENSMUSG00000096862,"ENSMUSG00000096862",0,0,0,0,0,0,0,0,0,0,0,0,0,0,0,0,0,0,0,0 | ENSMUSG00000094534,"ENSMUSG00000094534",0,0,0,0,0,0,0,0,0,0,1,0,0,0,0,0,0,0,1,0 |
| ENSMUSG00000025357,"ENSMUSG00000025357",1530,1801,2638,2596,2313,1864,2140,1622,1970,2341,2062,1511,1754,2527,2067,2282,1934,2175,1634,1861 | ENSMUSG00000079853,"ENSMUSG00000079853",3,5,2,1,5,3,2,5,1,2,5,1,3,0,4,3,2,2,2,1 | ENSMUSG00000094516,"ENSMUSG00000094516",0,0,0,0,0,0,0,0,0,0,0,0,0,0,0,0,0,0,0,0 |
| ENSMUSG00000004897,"ENSMUSG00000004897",2388,3547,4491,4241,4026,3540,3872,3028,3529,3990,4657,3548,4167,3798,3487,4251,3518,4848,3680,4104 | ENSMUSG00000053367,"ENSMUSG00000053367",0,0,0,0,0,0,0,0,0,0,0,0,0,0,0,0,0,0,0,0 | ENSMUSG00000095221,"ENSMUSG00000095221",0,0,0,0,0,0,0,0,0,0,0,0,0,0,0,0,0,0,0,0 |
| ENSMUSG00000028064,"ENSMUSG00000028064",2413,3207,3377,3312,3585,3071,3320,2760,3061,3273,3520,2809,3494,3446,3130,3858,2967,4219,2890,3348 | ENSMUSG00000074183,"ENSMUSG00000074183",2,8,8,7,3,8,8,2,10,11,6,0,2,3,4,14,5,8,8,9 | ENSMUSG00000110616,"ENSMUSG00000110616",0,0,0,1,0,0,1,0,0,1,0,0,0,0,0,0,0,0,0,0 |
| ENSMUSG00000017776,"ENSMUSG00000017776",3537,3346,4098,4024,4223,3832,3596,2971,3104,3941,3787,2952,3272,3657,3591,3791,3326,4180,2864,3123 | ENSMUSG00000055602,"ENSMUSG00000055602",1,6,11,8,9,8,11,7,10,22,7,5,7,10,10,18,15,30,15,10 | ENSMUSG00000088736,"ENSMUSG00000088736",0,0,0,0,0,0,0,0,0,0,0,0,0,0,0,0,1,0,0,0 |
| ENSMUSG00000030342,"ENSMUSG00000030342",8718,6919,7955,7356,7787,7252,7927,5950,6821,7525,7291,6401,6985,7265,6300,7985,6137,8347,5947,7547 | ENSMUSG00000046101,"ENSMUSG00000046101",7,1,2,3,3,8,1,3,1,3,3,1,0,3,2,2,2,2,0,1 | ENSMUSG00000110359,"ENSMUSG00000110359",0,0,0,0,0,0,0,0,0,0,0,0,0,0,0,0,0,0,0,0 |
| ENSMUSG00000026277,"ENSMUSG00000026277",1010,1248,1594,1553,1511,1344,1426,1207,1328,1371,1475,1182,1317,1330,1272,1446,1229,1654,1439,1325 | ENSMUSG00000086881,"ENSMUSG00000086881",4,1,0,4,4,0,12,3,3,4,11,6,3,4,3,2,3,1,2,2 | ENSMUSG00000109668,"ENSMUSG00000109668",0,0,0,0,0,0,0,0,0,0,0,0,0,0,0,0,0,0,0,0 |
| ENSMUSG00000031402,"ENSMUSG00000031402",1372,1056,1731,1532,1454,1400,1102,1232,1229,1520,1637,1095,1641,1354,1348,1707,1383,1904,1227,1606 | ENSMUSG00000051008,"ENSMUSG00000051008",2,2,3,4,6,2,3,2,7,3,9,2,5,3,3,6,3,3,2,1 | ENSMUSG00000064519,"ENSMUSG00000064519",0,0,0,0,0,0,0,0,0,0,0,0,0,0,0,0,0,0,0,0 |
| ENSMUSG00000009090,"ENSMUSG00000009090",4352,5009,7285,6627,6300,6227,5477,5337,5549,5504,6509,4398,6072,6067,5364,5802,5384,6280,5148,5690 | ENSMUSG00000092495,"ENSMUSG00000092495",3,10,3,8,13,4,14,10,5,10,6,1,9,8,4,4,11,7,6,9 | ENSMUSG00000109725,"ENSMUSG00000109725",0,0,0,0,0,0,0,0,0,0,0,0,0,0,0,0,0,0,0,0 |
| ENSMUSG00000028337,"ENSMUSG00000028337",346,440,465,466,540,479,447,420,528,459,504,415,458,484,478,543,412,567,335,405 | ENSMUSG00000031302,"ENSMUSG00000031302",6,6,2,3,10,5,1,2,5,7,9,0,5,3,3,3,5,9,5,2 | ENSMUSG00000110406,"ENSMUSG00000110406",0,0,0,0,0,0,0,0,0,0,0,0,0,0,0,0,0,0,0,0 |
| ENSMUSG00000034342,"ENSMUSG00000034342",4389,4072,4245,4209,4765,4261,4097,3618,4105,4657,4083,3626,4247,4583,4046,4611,4356,4664,2897,3821 | ENSMUSG00000106705,"ENSMUSG00000106705",0,0,0,0,0,0,0,0,0,0,0,0,0,0,0,0,0,0,0,0 | ENSMUSG00000109740,"ENSMUSG00000109740",0,0,0,0,0,0,0,0,0,0,1,0,0,0,0,0,0,0,0,0 |
| ENSMUSG00000031299,"ENSMUSG00000031299",1569,1947,2289,2287,2184,1961,2042,1532,1881,2263,2432,1684,2062,2066,1858,2009,1843,2347,1807,1965 | ENSMUSG00000068962,"ENSMUSG00000068962",0,4,4,0,3,4,1,1,2,5,2,2,0,2,2,2,1,3,2,0 | ENSMUSG00000109744,"ENSMUSG00000109744",0,0,0,0,0,0,0,0,0,0,0,0,0,0,0,0,0,0,0,0 |
| ENSMUSG00000019970,"ENSMUSG00000019970",2368,2453,2927,2679,2297,2780,2594,3059,3580,2612,2305,1795,2907,2952,2434,2743,2655,2871,1913,3332 | ENSMUSG00000110114,"ENSMUSG00000110114",17,18,27,26,26,46,24,33,27,23,39,13,30,24,39,59,19,43,21,42 | ENSMUSG00000110533,"ENSMUSG00000110533",0,0,0,0,0,0,0,0,0,0,0,0,0,0,0,1,0,0,0,0 |
| ENSMUSG00000032350,"ENSMUSG00000032350",837,809,882,882,796,886,777,589,713,877,874,720,693,819,682,802,759,787,526,660 | ENSMUSG00000109857,"ENSMUSG00000109857",13,11,16,22,3,8,14,7,4,10,5,11,8,4,6,4,11,6,6,6 | ENSMUSG00000107280,"ENSMUSG00000107280",0,0,0,0,0,0,0,0,0,0,0,0,0,0,0,0,0,0,0,0 |
| ENSMUSG00000059495,"ENSMUSG00000059495",2989,2840,3247,3636,3356,2839,3316,2369,2870,2935,3444,2808,2908,2889,2619,2992,2811,3342,2180,2680 | ENSMUSG00000040140,"ENSMUSG00000040140",1,1,1,1,2,0,3,1,1,1,2,3,0,3,0,3,2,6,2,3 | ENSMUSG00000074252,"ENSMUSG00000074252",0,0,0,0,1,0,1,0,0,0,0,0,0,0,0,0,0,1,0,0 |
| ENSMUSG00000020914,"ENSMUSG00000020914",2151,1872,2708,1978,2025,2314,1683,1948,1695,1890,2246,1715,2386,1709,1719,2237,1651,2726,1696,2333 | ENSMUSG00000024134,"ENSMUSG00000024134",0,2,3,0,2,2,1,0,1,3,0,0,0,2,2,1,0,0,0,6 | ENSMUSG00000110640,"ENSMUSG00000110640",0,0,0,0,0,0,0,0,0,0,0,0,0,0,0,0,0,0,0,0 |
| ENSMUSG00000024759,"ENSMUSG00000024759",2826,2470,2974,3022,2871,2755,2512,2075,2544,2732,3311,2428,2671,2474,2225,2660,2381,2876,1822,2208 | ENSMUSG00000104501,"ENSMUSG00000104501",0,0,0,0,0,0,0,0,0,0,0,0,0,1,0,0,0,0,0,0 | ENSMUSG00000110448,"ENSMUSG00000110448",0,0,0,0,0,0,0,0,0,0,0,0,0,0,0,0,0,0,0,0 |
| ENSMUSG00000058600,"ENSMUSG00000058600",1569,1797,2086,2140,1782,1680,1725,1329,1930,1952,1856,1700,1575,1707,1517,1835,1581,2010,1369,1700 | ENSMUSG00000062074,"ENSMUSG00000062074",0,1,2,3,5,2,2,3,5,0,9,3,9,3,1,2,2,1,1,7 | ENSMUSG00000098684,"ENSMUSG00000098684",0,0,0,0,0,0,1,0,0,0,0,0,0,0,0,0,0,0,0,0 |
| ENSMUSG00000040134,"ENSMUSG00000040134",0,0,0,0,0,0,0,2,0,0,0,0,1,0,3,0,0,0,0,2 | ENSMUSG00000109295,"ENSMUSG00000109295",0,0,0,0,0,0,0,0,0,0,0,0,0,0,0,0,0,0,0,0 | ENSMUSG00000098360,"ENSMUSG00000098360",0,0,0,0,0,0,0,0,0,0,0,0,0,0,0,0,0,0,0,0 |
| ENSMUSG00000062345,"ENSMUSG00000062345",21671,17324,15223,16809,18265,14641,18753,9557,15329,21750,13107,15105,9236,17614,15274,17838,15137,15762,8795,14398 | ENSMUSG00000111253,"ENSMUSG00000111253",0,0,0,0,0,0,0,1,0,0,0,0,0,0,0,0,0,0,0,0 | ENSMUSG00000089179,"ENSMUSG00000089179",0,0,0,0,0,0,0,0,0,0,0,0,0,2,0,0,0,0,0,0 |
| ENSMUSG00000024853,"ENSMUSG00000024853",1400,2652,3616,3582,2960,2876,3153,2697,2859,3013,3606,2555,3091,3276,2989,3467,2725,3719,2880,3194 | ENSMUSG00000081738,"ENSMUSG00000081738",2,4,9,6,7,11,5,3,4,10,8,6,5,6,5,10,11,3,6,4 | ENSMUSG00000110073,"ENSMUSG00000110073",0,0,0,0,0,0,0,0,0,0,0,0,0,0,0,0,0,0,0,0 |
| ENSMUSG00000006169,"ENSMUSG00000006169",2982,3362,4356,4270,4501,3937,3778,3331,3627,4042,4108,3249,3764,4113,3785,4520,3720,4888,3384,4108 | ENSMUSG00000110824,"ENSMUSG00000110824",3,13,10,17,14,14,19,7,9,16,15,11,12,14,13,14,7,14,7,3 | ENSMUSG00000110670,"ENSMUSG00000110670",0,0,0,0,0,0,0,0,0,0,0,0,0,0,0,0,0,0,0,0 |
| ENSMUSG00000036112,"ENSMUSG00000036112",2412,2479,3141,3288,2928,2830,2933,2041,2472,2768,3087,2574,2425,2635,2542,2735,2383,3094,2247,2465 | ENSMUSG00000099517,"ENSMUSG00000099517",1,5,6,2,3,6,2,2,2,2,0,2,1,2,2,2,2,3,6,2 | ENSMUSG00000098116,"ENSMUSG00000098116",0,0,0,0,0,0,0,0,0,0,0,0,0,0,0,0,0,0,0,0 |
| ENSMUSG00000005142,"ENSMUSG00000005142",2928,3691,5386,3795,4557,4781,3605,3743,3759,4038,4466,2596,4960,3950,3586,4487,3689,5266,3555,4005 | ENSMUSG00000022155,"ENSMUSG00000022155",0,0,0,0,0,0,0,0,0,0,0,0,0,0,0,1,0,0,0,0 | ENSMUSG00000110014,"ENSMUSG00000110014",0,0,0,0,0,0,0,0,0,0,0,0,0,0,0,0,0,0,0,0 |
| ENSMUSG00000026773,"ENSMUSG00000026773",1674,2246,3059,2774,3413,2544,1970,3163,2464,2722,3157,2514,3794,3102,2845,3487,2751,3694,1939,2922 | ENSMUSG00000054934,"ENSMUSG00000054934",10,9,18,16,13,10,17,14,13,16,14,9,20,5,14,13,6,23,10,22 | ENSMUSG00000110479,"ENSMUSG00000110479",0,0,0,0,0,0,0,0,0,0,0,0,0,0,0,0,0,0,0,0 |
| ENSMUSG00000042406,"ENSMUSG00000042406",2140,4526,5132,5677,4999,4580,5312,3703,4973,5099,5143,4453,3935,4791,4447,5264,4291,5067,3610,4501 | ENSMUSG00000115801,"ENSMUSG00000115801",3,10,12,11,5,9,9,7,8,10,8,15,9,14,5,7,6,12,14,9 | ENSMUSG00000095941,"ENSMUSG00000095941",0,0,0,0,0,0,0,0,0,0,0,0,0,0,0,0,0,0,0,0 |
| ENSMUSG00000063903,"ENSMUSG00000063903",118,138,224,189,168,224,111,258,156,157,148,87,278,153,189,352,237,373,293,219 | ENSMUSG00000090026,"ENSMUSG00000090026",6,0,3,6,4,11,8,6,6,5,7,7,3,7,1,5,2,1,4,5 | ENSMUSG00000088428,"ENSMUSG00000088428",0,0,0,0,0,0,0,0,0,0,0,0,0,0,0,0,0,0,0,0 |
| ENSMUSG00000020964,"ENSMUSG00000020964",2508,2397,3403,3635,3524,3175,2914,2538,2831,3121,3279,2633,3148,3055,2821,3204,2749,3398,2361,2783 | ENSMUSG00000098620,"ENSMUSG00000098620",6,7,5,4,2,5,1,2,3,3,1,5,1,9,7,6,6,6,6,2 | ENSMUSG00000065090,"ENSMUSG00000065090",0,0,0,0,0,0,0,0,0,1,0,0,0,0,0,0,0,0,0,0 |
| ENSMUSG00000024331,"ENSMUSG00000024331",3187,2500,3831,3175,3641,2380,2664,3267,3187,2404,3210,2991,3590,2742,3257,4043,2545,4396,2533,4146 | ENSMUSG00000079657,"ENSMUSG00000079657",3,4,24,7,15,14,11,4,15,6,13,9,8,9,11,11,4,18,8,21 | ENSMUSG00000077421,"ENSMUSG00000077421",0,0,0,0,0,0,0,0,0,0,0,0,0,0,0,0,0,0,0,0 |
| ENSMUSG00000048126,"ENSMUSG00000048126",5365,5589,8511,8342,7015,10472,8128,6350,7356,7369,7516,4655,8172,7297,6705,6509,6883,7314,6096,6683 | ENSMUSG00000081810,"ENSMUSG00000081810",5,6,8,11,3,5,3,2,11,11,8,13,5,7,3,18,2,5,2,5 | ENSMUSG00000090074,"ENSMUSG00000090074",0,0,0,0,0,0,0,0,0,0,0,0,0,0,0,0,0,0,0,0 |
| ENSMUSG00000026478,"ENSMUSG00000026478",1482,2008,2670,2495,2285,2781,2497,2047,2228,2691,2339,1596,2675,2187,2069,2490,2180,3079,2031,2351 | ENSMUSG00000014747,"ENSMUSG00000014747",0,5,14,10,10,5,7,5,7,5,8,4,0,3,4,12,7,7,6,10 | ENSMUSG00000088580,"ENSMUSG00000088580",0,0,0,0,0,0,0,0,0,0,0,0,0,0,0,0,0,0,0,0 |
| ENSMUSG00000062234,"ENSMUSG00000062234",2170,2500,3318,3398,2979,2883,2914,2569,2822,3077,3184,2579,3216,3049,2759,3275,2847,3663,2731,2949 | ENSMUSG00000080932,"ENSMUSG00000080932",3,4,6,8,4,0,7,9,16,16,14,7,6,11,15,7,10,16,8,14 | ENSMUSG00000091022,"ENSMUSG00000091022",0,0,0,0,0,0,0,0,0,0,0,0,0,0,0,0,0,0,0,0 |
| ENSMUSG00000037503,"ENSMUSG00000037503",2972,3191,3913,3933,3872,3448,3489,2782,3375,3538,3996,3014,3345,3436,3026,3503,3188,4007,2765,3014 | ENSMUSG00000074109,"ENSMUSG00000074109",25,26,23,33,24,17,13,25,13,22,16,14,24,15,28,24,25,36,15,13 | ENSMUSG00000091114,"ENSMUSG00000091114",0,0,0,0,0,0,0,0,0,0,0,0,0,0,0,0,0,0,0,0 |
| ENSMUSG00000035561,"ENSMUSG00000035561",45,71,101,98,113,111,79,107,91,82,81,51,108,100,83,124,98,174,107,110 | ENSMUSG00000086474,"ENSMUSG00000086474",3,0,0,0,0,1,0,0,2,2,0,2,3,0,0,1,2,2,0,1 | ENSMUSG00000096197,"ENSMUSG00000096197",0,0,0,0,0,0,0,0,0,0,0,0,0,0,0,0,0,0,0,0 |
| ENSMUSG00000027406,"ENSMUSG00000027406",1523,1564,1975,1915,1802,1569,1748,1442,1556,1690,1930,1504,1754,1696,1666,1682,1542,2025,1698,1702 | ENSMUSG00000113015,"ENSMUSG00000113015",5,8,17,36,17,20,31,12,13,22,23,21,16,20,23,30,20,28,31,25 | ENSMUSG00000095797,"ENSMUSG00000095797",0,0,0,0,0,0,0,0,0,0,0,0,0,0,0,0,0,0,0,0 |
| ENSMUSG00000039100,"ENSMUSG00000039100",1843,1629,2031,1830,2111,1826,1855,1626,1858,1894,1909,1468,1950,1814,1576,1785,1628,2192,1321,1675 | ENSMUSG00000116827,"ENSMUSG00000116827",27,9,21,14,24,48,23,18,25,35,29,8,17,19,10,22,19,31,21,24 | ENSMUSG00000095991,"ENSMUSG00000095991",0,0,0,0,0,0,0,0,0,0,0,0,0,1,0,0,0,0,0,0 |
| ENSMUSG00000027398,"ENSMUSG00000027398",13459,13102,16796,7178,17428,21125,15499,17915,14669,15600,11103,6923,19975,15192,11450,19796,11295,20421,10521,17124 | ENSMUSG00000071653,"ENSMUSG00000071653",1,4,7,5,10,3,10,7,1,4,9,5,4,0,2,2,4,5,9,7 | ENSMUSG00000097199,"ENSMUSG00000097199",0,0,0,0,0,0,0,0,0,0,0,1,0,0,0,0,0,0,0,0 |
| ENSMUSG00000022100,"ENSMUSG00000022100",1268,1098,1383,1346,1292,1241,1177,918,1173,1079,1437,1095,1197,1058,1139,1164,1062,1346,975,1144 | ENSMUSG00000081965,"ENSMUSG00000081965",2,5,7,4,7,0,3,1,1,5,5,2,3,3,2,2,1,3,5,0 | ENSMUSG00000072738,"ENSMUSG00000072738",0,0,0,0,0,0,0,0,0,0,0,0,0,0,0,0,0,0,0,0 |
| ENSMUSG00000031010,"ENSMUSG00000031010",2840,2222,2762,2488,2862,2573,2076,2181,2163,2665,2513,1862,2635,2389,2305,2553,2441,2641,1444,2080 | ENSMUSG00000022296,"ENSMUSG00000022296",23,23,15,34,22,32,38,17,30,27,27,13,32,25,27,31,31,26,27,22 | ENSMUSG00000091227,"ENSMUSG00000091227",0,0,0,0,0,0,0,0,0,0,0,0,0,0,0,0,0,0,0,0 |
| ENSMUSG00000058624,"ENSMUSG00000058624",5043,4649,4902,5763,5265,5222,4265,3313,4390,5874,5011,3894,4036,5141,4191,5278,4370,5717,2741,3900 | ENSMUSG00000104520,"ENSMUSG00000104520",2,0,4,7,3,3,0,1,1,4,2,1,0,0,0,0,1,2,0,1 | ENSMUSG00000114467,"ENSMUSG00000114467",0,0,0,0,0,0,0,0,0,0,0,0,0,0,0,0,0,0,0,0 |
| ENSMUSG00000039515,"ENSMUSG00000039515",1564,2015,2754,2564,2327,2418,2310,1950,2060,2317,2723,1937,2462,2362,2071,2377,2121,2728,2141,2337 | ENSMUSG00000069136,"ENSMUSG00000069136",1,1,5,2,2,3,1,1,0,3,1,1,4,0,1,1,6,2,3,4 | ENSMUSG00000077158,"ENSMUSG00000077158",0,0,0,0,0,0,1,0,0,0,0,0,0,0,0,0,0,0,0,0 |
| ENSMUSG00000037053,"ENSMUSG00000037053",0,0,0,0,0,0,1,0,0,0,0,0,0,0,1,0,0,0,2,0 | ENSMUSG00000068243,"ENSMUSG00000068243",0,12,5,2,6,3,5,4,5,8,5,3,6,7,0,4,3,3,2,5 | ENSMUSG00000114676,"ENSMUSG00000114676",0,0,0,0,0,0,0,0,0,0,0,0,0,0,0,0,0,0,0,0 |
| ENSMUSG00000037373,"ENSMUSG00000037373",2304,2305,3098,2850,2755,2594,2460,1982,2347,2843,2858,2151,2523,2575,2406,2880,2391,3373,2182,2517 | ENSMUSG00000115662,"ENSMUSG00000115662",7,4,10,11,0,3,6,2,4,6,6,2,6,9,5,11,2,2,2,2 | ENSMUSG00000114698,"ENSMUSG00000114698",0,0,0,0,0,0,0,0,0,0,0,0,0,0,0,0,0,0,0,0 |
| ENSMUSG00000030275,"ENSMUSG00000030275",2332,1818,2014,2252,2216,2289,1967,1875,1886,2276,2216,1608,2214,1983,2044,2295,1997,2593,1415,1836 | ENSMUSG00000113416,"ENSMUSG00000113416",21,8,5,14,16,17,11,13,14,3,4,4,16,11,3,4,20,23,11,12 | ENSMUSG00000114678,"ENSMUSG00000114678",0,0,0,0,0,0,0,0,0,0,0,0,0,0,0,0,0,0,0,0 |
| ENSMUSG00000050565,"ENSMUSG00000050565",3962,3011,3620,4391,3430,3150,3170,2938,3480,3536,3539,2787,3047,3076,3092,3937,2896,3908,2610,3117 | ENSMUSG00000038540,"ENSMUSG00000038540",6,5,1,1,1,2,1,3,0,1,1,5,0,0,0,7,1,3,0,0 | ENSMUSG00000105635,"ENSMUSG00000105635",0,0,0,0,0,0,0,0,0,0,0,0,0,0,0,0,0,0,0,0 |
| ENSMUSG00000028757,"ENSMUSG00000028757",2244,2676,3927,3696,3460,3381,3409,2879,3082,2894,3411,2800,3481,3242,3342,3547,2757,3829,3180,3447 | ENSMUSG00000108696,"ENSMUSG00000108696",9,3,9,8,6,7,9,10,5,6,9,2,11,8,6,15,14,18,11,14 | ENSMUSG00000114636,"ENSMUSG00000114636",0,0,0,0,0,0,0,0,0,0,0,1,0,0,0,0,0,0,0,0 |
| ENSMUSG00000030671,"ENSMUSG00000030671",493,448,642,495,551,580,420,498,507,563,542,367,732,486,559,570,575,670,342,550 | ENSMUSG00000111282,"ENSMUSG00000111282",0,1,0,0,0,0,0,0,0,0,0,0,0,0,0,0,2,0,0,0 | ENSMUSG00000114436,"ENSMUSG00000114436",0,0,0,0,1,0,0,0,0,0,0,0,0,0,0,0,0,0,0,0 |
| ENSMUSG00000037458,"ENSMUSG00000037458",2555,2129,2111,2573,2305,2125,2250,1666,2158,2682,2211,1881,1696,2399,1945,2038,1951,2228,1297,1729 | ENSMUSG00000110179,"ENSMUSG00000110179",27,28,22,57,44,25,28,11,24,25,47,41,14,33,32,43,42,39,35,37 | ENSMUSG00000114416,"ENSMUSG00000114416",0,0,0,0,0,0,0,0,0,0,0,0,0,0,0,0,0,1,0,1 |
| ENSMUSG00000020839,"ENSMUSG00000020839",810,537,560,605,621,502,677,377,657,574,412,455,362,517,457,574,516,619,402,554 | ENSMUSG00000050321,"ENSMUSG00000050321",0,0,3,0,1,0,0,0,0,0,0,1,0,0,1,0,0,1,0,0 | ENSMUSG00000075245,"ENSMUSG00000075245",0,0,0,0,0,0,2,0,0,0,0,0,0,0,0,0,0,0,0,0 |
| ENSMUSG00000021096,"ENSMUSG00000021096",2161,2281,2647,2673,2473,2307,2325,1794,2254,2449,2327,2041,2072,2316,2060,2449,2159,2630,1807,2124 | ENSMUSG00000084771,"ENSMUSG00000084771",0,2,2,6,2,1,7,1,1,1,6,6,1,2,2,4,0,0,4,2 | ENSMUSG00000115445,"ENSMUSG00000115445",0,0,0,0,0,0,0,0,0,0,0,0,0,0,0,0,0,0,0,0 |
| ENSMUSG00000006005,"ENSMUSG00000006005",2590,2715,3360,3421,3121,3055,2638,2584,2713,3079,3464,2651,3155,2918,2810,3206,2890,3506,2135,2716 | ENSMUSG00000083246,"ENSMUSG00000083246",0,0,4,3,12,2,4,4,2,10,5,2,1,4,5,0,4,8,1,3 | ENSMUSG00000115760,"ENSMUSG00000115760",0,0,0,0,0,0,0,0,0,0,0,0,0,0,0,0,0,0,0,0 |
| ENSMUSG00000020532,"ENSMUSG00000020532",874,1175,1237,1409,1445,1289,1230,985,1214,1042,1462,1217,1240,1197,1036,1203,1039,1401,1171,1132 | ENSMUSG00000107605,"ENSMUSG00000107605",0,1,0,0,0,0,0,0,0,0,0,0,0,0,0,1,1,0,0,0 | ENSMUSG00000115248,"ENSMUSG00000115248",0,0,0,0,0,0,0,0,1,0,0,0,0,0,0,0,0,0,0,0 |
| ENSMUSG00000037331,"ENSMUSG00000037331",2320,3332,3894,4865,3730,3462,3873,2780,3371,3504,4354,3691,3046,3597,3464,3785,3322,4198,2955,3107 | ENSMUSG00000093826,"ENSMUSG00000093826",2,13,9,8,9,5,4,3,7,5,7,5,4,13,8,9,6,7,11,4 | ENSMUSG00000091131,"ENSMUSG00000091131",0,0,0,0,0,0,0,0,0,0,0,0,0,0,0,0,0,0,0,0 |
| ENSMUSG00000025465,"ENSMUSG00000025465",771,844,1110,1197,982,922,892,702,831,919,1229,955,969,931,808,959,790,975,918,811 | ENSMUSG00000098404,"ENSMUSG00000098404",10,7,9,11,7,11,4,7,8,8,6,10,5,10,12,5,3,9,4,9 | ENSMUSG00000114852,"ENSMUSG00000114852",0,0,0,0,0,0,0,0,0,0,0,0,0,0,0,0,0,0,0,0 |
| ENSMUSG00000020089,"ENSMUSG00000020089",2630,2237,2529,3203,2451,2486,2826,1796,2322,2557,2486,2344,1845,2498,2357,2663,2262,2917,1807,2226 | ENSMUSG00000063177,"ENSMUSG00000063177",4,16,16,10,13,21,6,23,16,10,15,5,15,11,17,25,13,22,20,13 | ENSMUSG00000072605,"ENSMUSG00000072605",0,0,0,0,0,0,0,0,0,0,0,0,0,0,0,0,0,0,0,0 |
| ENSMUSG00000068547,"ENSMUSG00000068547",0,0,0,2,1,2,0,0,0,0,0,0,0,0,0,2,1,0,0,4 | ENSMUSG00000103622,"ENSMUSG00000103622",3,5,17,14,14,10,5,11,4,5,9,10,7,3,5,8,7,11,9,13 | ENSMUSG00000091733,"ENSMUSG00000091733",0,0,0,0,0,3,0,0,0,0,0,0,0,0,0,0,0,0,0,0 |
| ENSMUSG00000003847,"ENSMUSG00000003847",6557,5040,5621,5393,6178,5252,5326,4320,5331,6105,5394,4650,5247,5279,5168,6014,5374,6381,3404,4692 | ENSMUSG00000086438,"ENSMUSG00000086438",0,0,0,0,0,0,0,0,0,0,0,2,0,2,1,0,1,0,0,0 | ENSMUSG00000094151,"ENSMUSG00000094151",0,0,0,0,0,0,0,0,0,0,0,0,0,0,0,0,0,0,0,0 |
| ENSMUSG00000020088,"ENSMUSG00000020088",3698,3237,4175,4030,3872,3833,3634,3121,3367,3994,4203,3129,3704,3703,3498,3849,3464,4372,2911,3720 | ENSMUSG00000050141,"ENSMUSG00000050141",23,15,15,25,19,13,9,8,6,16,19,14,17,13,14,5,9,27,13,8 | ENSMUSG00000091707,"ENSMUSG00000091707",0,0,1,0,0,0,0,0,0,0,0,0,0,0,1,0,1,0,0,0 |
| ENSMUSG00000035248,"ENSMUSG00000035248",3308,3657,4175,4253,4243,4186,3867,3510,3710,4529,3683,2858,3847,4258,3656,4387,3735,4842,2638,3624 | ENSMUSG00000052241,"ENSMUSG00000052241",6,3,12,10,9,9,2,4,9,6,16,7,12,6,5,10,2,8,5,3 | ENSMUSG00000115500,"ENSMUSG00000115500",0,0,0,0,0,0,0,0,0,0,0,0,0,0,0,0,0,0,0,0 |
| ENSMUSG00000038224,"ENSMUSG00000038224",3,0,7,7,1,1,10,2,4,4,3,3,0,6,2,7,4,1,10,6 | ENSMUSG00000076571,"ENSMUSG00000076571",0,0,0,0,0,0,0,0,0,0,0,0,0,0,0,0,0,0,0,0 | ENSMUSG00000115054,"ENSMUSG00000115054",0,0,0,0,0,0,0,0,0,0,0,0,0,0,0,0,0,0,0,0 |
| ENSMUSG00000022312,"ENSMUSG00000022312",2283,3153,3968,3943,3548,3301,3604,2771,3159,3472,3759,3111,3358,3515,3180,3807,2999,4076,3103,3352 | ENSMUSG00000097245,"ENSMUSG00000097245",0,0,0,3,0,2,2,3,3,1,7,1,1,3,9,3,0,1,1,4 | ENSMUSG00000115421,"ENSMUSG00000115421",1,0,0,0,1,0,0,0,0,0,0,0,0,0,0,0,0,0,0,0 |
| ENSMUSG00000024360,"ENSMUSG00000024360",5595,4731,4894,5344,5136,4951,5007,3667,4697,5197,4857,4366,3864,4815,3947,4740,4267,4947,3321,4145 | ENSMUSG00000086360,"ENSMUSG00000086360",7,5,3,2,4,6,4,2,3,4,4,3,1,4,3,1,2,7,2,1 | ENSMUSG00000115046,"ENSMUSG00000115046",0,0,0,0,0,0,0,0,0,0,1,0,0,0,0,0,1,0,0,0 |
| ENSMUSG00000096210,"ENSMUSG00000096210",1491,2096,2411,2204,2250,2019,1903,1862,2113,2113,2315,2000,2393,2134,1931,2079,1861,2514,2298,2605 | ENSMUSG00000096107,"ENSMUSG00000096107",0,0,0,0,1,2,0,0,0,0,2,0,1,0,0,0,0,0,1,0 | ENSMUSG00000048080,"ENSMUSG00000048080",0,0,0,0,0,0,0,0,0,0,0,0,0,0,0,0,0,0,0,0 |
| ENSMUSG00000030058,"ENSMUSG00000030058",2049,2158,2961,3044,2797,2755,2833,2328,2318,2508,2879,2104,2691,2725,2630,2806,2388,2799,2341,2553 | ENSMUSG00000097632,"ENSMUSG00000097632",0,4,1,1,2,3,2,1,2,3,1,2,6,2,5,3,3,1,0,3 | ENSMUSG00000091873,"ENSMUSG00000091873",0,0,0,0,0,0,0,0,0,0,0,0,0,0,0,0,0,0,0,0 |
| ENSMUSG00000001467,"ENSMUSG00000001467",3133,2911,2691,4775,3719,2744,3183,2300,3278,2907,3618,3882,2892,3201,2918,3245,2781,3497,2966,2773 | ENSMUSG00000082436,"ENSMUSG00000082436",31,11,19,25,9,26,19,24,14,22,15,9,14,32,12,26,29,20,12,15 | ENSMUSG00000045306,"ENSMUSG00000045306",0,0,0,0,0,0,0,0,0,0,0,0,0,0,0,0,0,0,0,0 |
| ENSMUSG00000002222,"ENSMUSG00000002222",1897,1815,2051,2141,1858,1613,1749,1410,1807,1753,2141,1682,2012,1618,1739,1887,1657,2061,1401,1667 | ENSMUSG00000027596,"ENSMUSG00000027596",3,25,33,20,23,27,16,19,19,27,31,20,36,30,21,42,12,41,22,36 | ENSMUSG00000115540,"ENSMUSG00000115540",0,0,0,0,0,0,0,0,0,0,0,0,0,0,0,0,0,0,0,0 |
| ENSMUSG00000020120,"ENSMUSG00000020120",7839,8493,9940,7268,9712,11162,8771,8455,7702,13030,7796,5500,10191,11159,8408,10942,9227,11183,5320,8964 | ENSMUSG00000092072,"ENSMUSG00000092072",7,9,8,4,7,12,7,4,7,7,8,6,8,4,5,3,12,11,4,10 | ENSMUSG00000000606,"ENSMUSG00000000606",0,0,0,0,0,0,0,0,0,0,0,0,0,0,0,0,0,0,0,0 |
| ENSMUSG00000052296,"ENSMUSG00000052296",1597,2198,2941,2794,2673,2505,2636,2408,2317,2812,2874,2038,2831,2565,2482,2846,2568,3296,2406,2683 | ENSMUSG00000034762,"ENSMUSG00000034762",14,25,29,17,20,28,26,21,19,23,23,11,26,18,16,15,25,20,16,28 | ENSMUSG00000106620,"ENSMUSG00000106620",0,0,0,0,0,0,0,0,0,0,0,0,0,0,0,0,0,0,0,0 |
| ENSMUSG00000007880,"ENSMUSG00000007880",1642,2349,2935,2578,2570,2702,2762,2257,2559,2653,2854,2110,3058,2613,2511,2815,2513,3304,2339,2512 | ENSMUSG00000097603,"ENSMUSG00000097603",0,0,0,0,0,0,0,0,3,0,0,0,0,0,0,0,2,1,0,0 | ENSMUSG00000098961,"ENSMUSG00000098961",0,0,0,0,0,0,1,0,0,0,0,0,2,0,0,0,0,0,0,0 |
| ENSMUSG00000046805,"ENSMUSG00000046805",3899,5069,5263,6939,5906,5413,4491,5592,4949,5508,5644,3789,6546,5913,5318,7908,5837,9205,4802,5500 | ENSMUSG00000034416,"ENSMUSG00000034416",11,15,20,13,18,15,8,15,15,8,12,12,15,10,15,20,9,14,7,17 | ENSMUSG00000115595,"ENSMUSG00000115595",0,0,0,0,0,0,2,0,0,0,0,0,1,0,0,0,0,0,0,0 |
| ENSMUSG00000054693,"ENSMUSG00000054693",4160,3862,4675,4561,4467,3904,4098,3417,4034,4203,4383,3511,3907,4167,3937,4568,3983,4788,3190,3943 | ENSMUSG00000115426,"ENSMUSG00000115426",22,13,23,8,23,20,17,21,27,30,36,20,23,29,26,26,24,34,28,25 | ENSMUSG00000092995,"ENSMUSG00000092995",0,0,0,0,0,0,0,0,0,0,0,0,0,0,0,1,0,0,0,0 |
| ENSMUSG00000026687,"ENSMUSG00000026687",565,669,916,786,781,742,735,735,722,705,852,604,722,771,690,777,685,968,607,740 | ENSMUSG00000027530,"ENSMUSG00000027530",6,7,17,5,12,6,9,12,20,12,9,11,10,6,12,10,12,20,11,9 | ENSMUSG00000114440,"ENSMUSG00000114440",0,0,1,0,0,0,0,0,0,0,0,0,0,1,0,0,0,0,0,0 |
| ENSMUSG00000020640,"ENSMUSG00000020640",5502,5444,5775,6225,6431,5436,5517,4543,5248,6382,5609,4875,5305,6124,5300,6168,5713,6189,3727,4995 | ENSMUSG00000106980,"ENSMUSG00000106980",2,0,3,0,1,1,0,4,1,1,2,2,5,0,0,4,3,4,1,0 | ENSMUSG00000114258,"ENSMUSG00000114258",0,0,0,0,0,0,0,0,0,0,0,0,0,0,0,0,3,0,0,0 |
| ENSMUSG00000044894,"ENSMUSG00000044894",709,1678,2455,1742,2040,1738,1864,1563,1652,1943,2023,1727,2236,1934,1808,1997,1828,2724,1852,2197 | ENSMUSG00000088054,"ENSMUSG00000088054",2,0,2,1,1,0,0,0,0,1,0,0,0,1,0,0,0,0,0,0 | ENSMUSG00000114844,"ENSMUSG00000114844",0,0,0,0,0,0,0,0,0,0,0,0,0,0,0,0,0,0,0,0 |
| ENSMUSG00000018583,"ENSMUSG00000018583",2607,2767,3203,3597,2932,2972,3296,2444,2834,2853,3621,2850,2827,3121,2801,2921,2523,3197,2788,2876 | ENSMUSG00000103473,"ENSMUSG00000103473",9,8,17,15,17,19,15,15,3,15,9,10,5,7,16,7,11,14,4,9 | ENSMUSG00000114514,"ENSMUSG00000114514",0,0,0,0,0,0,0,0,0,0,0,0,0,0,0,0,0,0,0,0 |
| ENSMUSG00000053113,"ENSMUSG00000053113",8087,13503,14126,13157,15796,15931,14869,12173,14199,16105,11671,9811,12884,17262,13217,17422,11893,16322,10309,12818 | ENSMUSG00000065036,"ENSMUSG00000065036",22,14,12,14,14,13,21,5,12,10,12,15,7,21,9,5,12,10,3,10 | ENSMUSG00000097511,"ENSMUSG00000097511",0,0,0,0,0,0,0,0,0,0,0,0,0,0,0,0,0,0,0,0 |
| ENSMUSG00000031696,"ENSMUSG00000031696",4241,3155,4041,3887,4028,3526,3474,3033,3244,3682,4011,2833,3444,3319,3323,3583,3311,3866,2527,3193 | ENSMUSG00000097702,"ENSMUSG00000097702",1,1,0,0,0,0,0,0,0,0,2,0,0,0,0,1,0,0,0,0 | ENSMUSG00000115304,"ENSMUSG00000115304",0,0,0,0,0,0,0,0,0,0,0,0,0,0,0,0,0,0,0,0 |
| ENSMUSG00000047187,"ENSMUSG00000047187",2718,3027,4035,3775,3523,3272,3298,2456,3067,3590,3579,2823,3172,3422,2976,3306,2944,3779,2598,3231 | ENSMUSG00000032318,"ENSMUSG00000032318",0,0,4,5,2,5,0,0,3,2,0,0,0,2,0,1,0,0,0,2 | ENSMUSG00000115530,"ENSMUSG00000115530",0,0,0,0,0,1,1,0,0,0,0,0,0,0,0,0,0,0,0,0 |
| ENSMUSG00000021236,"ENSMUSG00000021236",520,488,617,720,576,622,632,508,559,505,721,626,568,515,482,630,472,741,519,566 | ENSMUSG00000112189,"ENSMUSG00000112189",0,0,0,0,0,0,1,0,0,0,0,0,0,0,0,0,0,0,0,0 | ENSMUSG00000115088,"ENSMUSG00000115088",0,0,0,0,0,0,0,0,0,0,0,0,0,0,1,0,2,0,0,0 |
| ENSMUSG00000022175,"ENSMUSG00000022175",2625,3509,4317,4489,4202,3895,4107,3574,3880,4198,3968,3355,4089,4458,3805,4650,4000,5038,3386,4098 | ENSMUSG00000078627,"ENSMUSG00000078627",4,5,14,10,8,5,10,1,1,6,4,15,9,8,3,6,5,6,2,10 | ENSMUSG00000115288,"ENSMUSG00000115288",0,0,0,0,0,0,0,0,0,0,0,0,0,0,0,0,0,0,0,0 |
| ENSMUSG00000033499,"ENSMUSG00000033499",3168,3245,3534,3964,3841,3041,3302,2697,3184,3666,3676,3223,3167,3366,3162,3660,3134,4047,2442,3051 | ENSMUSG00000090327,"ENSMUSG00000090327",0,11,8,8,6,10,7,5,8,1,11,14,10,10,15,7,4,5,9,15 | ENSMUSG00000102774,"ENSMUSG00000102774",0,0,0,1,0,0,0,0,0,0,0,0,0,0,0,0,0,0,0,0 |
| ENSMUSG00000029994,"ENSMUSG00000029994",2075,2072,2974,3115,2847,2259,2245,2455,2279,2429,2817,2090,2802,2559,2541,2774,2418,3094,2243,2630 | ENSMUSG00000104108,"ENSMUSG00000104108",1,10,6,2,2,0,4,2,2,2,4,1,6,5,0,6,1,5,1,1 | ENSMUSG00000115438,"ENSMUSG00000115438",0,0,0,0,0,0,0,0,0,0,0,0,0,0,0,0,0,0,0,0 |
| ENSMUSG00000028416,"ENSMUSG00000028416",2150,2985,3736,4207,3363,2865,3065,2392,2830,3118,3722,3231,2944,3158,3111,3469,2814,3381,2392,2747 | ENSMUSG00000001168,"ENSMUSG00000001168",1,13,10,4,4,1,11,5,9,2,8,3,8,4,5,4,3,4,11,3 | ENSMUSG00000096997,"ENSMUSG00000096997",0,0,0,0,0,0,0,0,0,0,0,0,0,0,0,0,0,0,0,0 |
| ENSMUSG00000021792,"ENSMUSG00000021792",186,228,268,291,205,288,236,151,176,254,273,176,251,214,178,217,165,286,158,238 | ENSMUSG00000097674,"ENSMUSG00000097674",6,9,7,20,12,12,16,3,16,7,12,7,9,10,11,14,8,14,3,11 | ENSMUSG00000099911,"ENSMUSG00000099911",0,0,0,0,0,0,0,0,0,0,0,0,0,0,0,0,0,0,0,0 |
| ENSMUSG00000043962,"ENSMUSG00000043962",2172,2918,3433,3619,3386,3064,3048,2435,3048,3279,3591,2845,2896,3321,2833,3163,2916,3719,2530,2804 | ENSMUSG00000082465,"ENSMUSG00000082465",2,8,11,13,12,3,3,6,4,9,11,5,9,9,7,7,8,5,6,7 | ENSMUSG00000100274,"ENSMUSG00000100274",0,0,0,0,0,0,0,0,0,0,0,0,0,0,0,0,0,0,0,0 |
| ENSMUSG00000060586,"ENSMUSG00000060586",1099,2066,2471,2866,2262,2332,2102,2498,2207,2633,2946,2223,3380,2876,2254,3317,2143,4435,2957,2649 | ENSMUSG00000099384,"ENSMUSG00000099384",1,4,0,0,2,2,3,0,3,0,3,1,6,1,1,2,1,3,0,0 | ENSMUSG00000115639,"ENSMUSG00000115639",0,0,0,0,0,0,0,0,0,0,0,0,0,0,0,0,0,0,0,0 |
| ENSMUSG00000030605,"ENSMUSG00000030605",951,1518,1650,2198,1654,1654,1923,1260,1498,1793,1799,1628,1504,1710,1430,1586,1288,1601,1450,1408 | ENSMUSG00000099760,"ENSMUSG00000099760",2,0,2,1,3,3,1,6,4,1,2,0,0,1,1,2,1,3,0,4 | ENSMUSG00000099994,"ENSMUSG00000099994",0,0,0,0,0,0,0,0,0,0,0,0,0,0,0,0,0,0,0,0 |
| ENSMUSG00000033060,"ENSMUSG00000033060",426,497,591,685,569,457,550,448,494,651,686,553,729,535,538,596,511,767,414,579 | ENSMUSG00000063314,"ENSMUSG00000063314",2,8,2,4,5,8,7,7,9,8,9,5,8,10,8,8,7,6,2,7 | ENSMUSG00000110660,"ENSMUSG00000110660",0,0,0,0,0,0,0,0,0,0,0,0,0,0,0,0,0,0,0,0 |
| ENSMUSG00000032193,"ENSMUSG00000032193",2442,3177,3311,4402,4052,3138,3966,2566,3813,3388,3183,3689,2411,3694,2919,3672,3057,3525,3008,3241 | ENSMUSG00000087020,"ENSMUSG00000087020",1,0,1,1,1,2,3,5,0,0,0,2,1,0,5,1,1,1,2,0 | ENSMUSG00000092896,"ENSMUSG00000092896",0,0,0,0,0,0,0,0,0,0,0,0,0,0,0,0,0,0,0,0 |
| ENSMUSG00000023959,"ENSMUSG00000023959",343,258,262,459,355,136,337,272,279,341,337,250,284,308,335,342,316,329,194,314 | ENSMUSG00000083064,"ENSMUSG00000083064",8,5,3,8,9,7,12,11,7,8,6,3,9,6,8,9,6,5,4,8 | ENSMUSG00000111353,"ENSMUSG00000111353",0,0,0,1,0,0,0,0,0,0,0,0,0,0,0,0,0,0,0,0 |
| ENSMUSG00000074280,"ENSMUSG00000074280",17229,12089,15017,14296,13568,10722,10704,10867,12630,11916,14165,12711,14104,12695,14757,17933,10772,18305,16084,15083 | ENSMUSG00000082399,"ENSMUSG00000082399",1,2,7,6,6,5,4,5,7,6,4,7,9,4,2,11,4,4,1,3 | ENSMUSG00000111786,"ENSMUSG00000111786",0,0,0,0,0,0,0,0,0,0,0,0,0,0,0,0,0,0,0,0 |
| ENSMUSG00000030713,"ENSMUSG00000030713",13390,12279,14598,20366,14248,11032,13088,13016,12635,12255,17958,15406,16645,13826,16536,18018,12806,21209,15451,17233 | ENSMUSG00000046637,"ENSMUSG00000046637",20,11,22,4,6,10,8,3,8,12,17,2,12,4,15,10,7,15,15,8 | ENSMUSG00000110944,"ENSMUSG00000110944",0,0,0,0,0,0,0,0,0,0,0,0,0,0,0,0,1,0,0,0 |
| ENSMUSG00000021488,"ENSMUSG00000021488",2235,2439,3072,2934,3076,2846,2549,2244,2421,2669,3211,2576,3062,2673,2308,2724,2411,3170,1937,2391 | ENSMUSG00000042371,"ENSMUSG00000042371",39,15,19,25,26,18,36,12,16,16,24,25,19,29,25,19,21,24,17,16 | ENSMUSG00000045204,"ENSMUSG00000045204",0,0,0,0,0,0,0,0,0,0,0,0,0,0,0,0,0,0,0,0 |
| ENSMUSG00000039682,"ENSMUSG00000039682",954,1119,1638,2043,1335,1573,1347,1414,1206,1223,1689,1237,1391,1425,1451,1443,1303,1986,1350,1499 | ENSMUSG00000044951,"ENSMUSG00000044951",13,32,8,27,28,14,22,22,17,33,48,21,69,24,34,31,40,59,40,41 | ENSMUSG00000059303,"ENSMUSG00000059303",0,0,0,0,0,0,0,0,0,0,0,0,0,0,0,0,0,0,0,0 |
| ENSMUSG00000025860,"ENSMUSG00000025860",2501,1875,2227,2300,2173,2142,1825,1662,2001,2256,2189,1608,1918,2006,1964,2170,2002,2283,1297,1759 | ENSMUSG00000100277,"ENSMUSG00000100277",0,0,0,0,0,0,0,0,0,0,0,0,0,0,0,0,0,0,0,2 | ENSMUSG00000051118,"ENSMUSG00000051118",0,0,0,0,0,0,0,0,0,0,0,0,0,0,0,0,0,0,0,0 |
| ENSMUSG00000068452,"ENSMUSG00000068452",61,86,84,233,90,60,82,63,84,95,76,75,63,99,110,126,75,151,48,92 | ENSMUSG00000033569,"ENSMUSG00000033569",1,0,0,2,0,0,0,0,2,0,0,1,2,0,0,0,2,0,0,0 | ENSMUSG00000098907,"ENSMUSG00000098907",0,0,0,0,0,0,0,0,0,0,0,0,0,0,0,0,0,0,0,0 |
| ENSMUSG00000036751,"ENSMUSG00000036751",710,2088,2617,2527,2272,2155,2232,1923,2102,2479,2472,1865,2379,2339,2084,2758,2114,2753,2531,2424 | ENSMUSG00000106290,"ENSMUSG00000106290",13,17,24,18,27,24,16,11,12,19,11,7,28,10,16,10,13,24,8,10 | ENSMUSG00000090067,"ENSMUSG00000090067",0,0,0,0,0,0,0,0,0,0,0,0,0,0,0,0,0,0,0,0 |
| ENSMUSG00000021591,"ENSMUSG00000021591",6749,7679,8378,9670,8752,7448,7880,7106,7622,8774,8272,7250,8733,8791,8938,10942,7941,10812,7304,8841 | ENSMUSG00000108033,"ENSMUSG00000108033",6,5,8,16,18,6,15,9,7,7,8,7,11,14,9,10,15,2,9,10 | ENSMUSG00000111459,"ENSMUSG00000111459",0,0,0,0,0,0,0,0,0,0,0,0,0,0,0,0,0,2,0,0 |
| ENSMUSG00000069917,"ENSMUSG00000069917",85,65,175,120,181,60,86,49,42,48,97,143,130,75,94,139,180,135,31,24 | ENSMUSG00000104769,"ENSMUSG00000104769",0,0,0,0,0,0,0,0,0,0,0,0,0,0,0,0,0,0,0,0 | ENSMUSG00000111558,"ENSMUSG00000111558",0,0,0,0,0,0,0,0,0,1,0,0,0,0,0,0,0,0,0,0 |
| ENSMUSG00000004530,"ENSMUSG00000004530",2420,4071,4973,5334,4807,4133,4396,3369,4270,4808,4692,4207,4127,4625,3871,4723,3882,5198,3703,4276 | ENSMUSG00000085360,"ENSMUSG00000085360",2,6,15,5,7,3,6,8,11,2,4,11,10,3,6,2,0,16,4,1 | ENSMUSG00000110901,"ENSMUSG00000110901",0,0,0,0,0,0,0,0,0,0,0,0,0,0,0,0,0,0,0,0 |
| ENSMUSG00000050912,"ENSMUSG00000050912",3300,3075,4084,3748,3953,3823,3045,3207,3351,3742,4204,2976,4166,3424,3377,3910,3333,4263,2943,3347 | ENSMUSG00000107876,"ENSMUSG00000107876",0,0,0,0,0,1,3,3,0,0,1,0,0,1,0,0,0,2,0,0 | ENSMUSG00000098796,"ENSMUSG00000098796",0,0,0,0,0,0,0,0,0,0,0,0,0,0,0,0,0,0,0,0 |
| ENSMUSG00000027465,"ENSMUSG00000027465",1912,2090,2660,2590,2501,2452,2343,1927,2228,2386,2557,1962,2562,2569,2038,2610,2071,2897,1973,2232 | ENSMUSG00000107909,"ENSMUSG00000107909",0,4,5,4,4,1,2,1,4,5,8,7,4,1,5,3,3,4,1,0 | ENSMUSG00000091174,"ENSMUSG00000091174",0,0,0,0,0,0,0,0,0,0,0,0,0,0,0,0,0,0,0,0 |
| ENSMUSG00000025332,"ENSMUSG00000025332",3020,3226,3987,4347,4065,3599,4196,3456,3515,3822,4079,3321,3832,3920,3821,4215,3609,4532,3390,3688 | ENSMUSG00000046378,"ENSMUSG00000046378",1,4,2,1,2,1,4,5,4,2,6,11,5,5,4,1,7,12,8,7 | ENSMUSG00000111023,"ENSMUSG00000111023",0,0,0,0,0,0,0,0,0,0,0,0,0,0,0,0,0,0,0,0 |
| ENSMUSG00000055782,"ENSMUSG00000055782",203,195,233,173,269,227,164,183,188,311,230,164,277,213,216,203,226,237,130,218 | ENSMUSG00000091514,"ENSMUSG00000091514",15,2,1,5,8,2,11,7,3,1,4,9,3,3,6,4,7,9,8,5 | ENSMUSG00000110535,"ENSMUSG00000110535",0,0,0,0,0,0,0,0,0,0,0,0,0,0,0,0,0,0,0,0 |
| ENSMUSG00000024772,"ENSMUSG00000024772",2555,4327,5568,4458,5136,5238,4422,3943,4356,5621,4245,3416,4803,5368,4575,5352,4863,5943,3451,5059 | ENSMUSG00000088769,"ENSMUSG00000088769",0,3,1,0,4,1,1,0,3,2,0,1,6,3,2,0,4,2,1,0 | ENSMUSG00000111462,"ENSMUSG00000111462",0,0,0,0,0,0,0,0,0,0,0,0,0,0,0,0,0,0,0,0 |
| ENSMUSG00000047731,"ENSMUSG00000047731",3115,4466,6029,5627,5592,4986,4634,4365,4628,5582,5240,4482,5309,5581,4843,6129,4823,6428,4103,4847 | ENSMUSG00000110291,"ENSMUSG00000110291",7,11,12,8,9,7,8,6,4,14,11,10,19,15,7,20,13,19,3,2 | ENSMUSG00000111107,"ENSMUSG00000111107",0,0,0,0,0,0,0,0,0,0,0,0,0,0,0,0,0,0,0,0 |
| ENSMUSG00000026615,"ENSMUSG00000026615",2846,3086,3713,4069,3438,3378,3370,2436,2927,3075,4003,3081,2935,3337,2959,3249,2958,3630,2424,2879 | ENSMUSG00000091968,"ENSMUSG00000091968",0,4,1,2,0,0,2,1,1,0,2,0,2,2,1,1,1,2,0,0 | ENSMUSG00000110727,"ENSMUSG00000110727",0,0,0,0,0,0,0,0,0,0,2,0,0,0,0,0,0,1,0,0 |
| ENSMUSG00000058793,"ENSMUSG00000058793",1396,1674,2124,1779,1891,2209,1818,1756,1643,2240,2062,1435,2323,2096,1723,2056,1926,2365,1468,1849 | ENSMUSG00000104016,"ENSMUSG00000104016",19,4,3,4,3,5,7,3,6,15,6,6,6,9,6,11,7,5,2,4 | ENSMUSG00000110862,"ENSMUSG00000110862",0,0,0,0,0,0,0,0,0,0,0,0,0,0,0,0,0,0,0,0 |
| ENSMUSG00000074656,"ENSMUSG00000074656",3494,2993,3559,3721,3272,3297,3309,2401,2891,3390,3253,2909,2626,2983,2866,3447,2989,3559,2373,2940 | ENSMUSG00000102923,"ENSMUSG00000102923",1,1,2,3,2,1,0,3,0,0,1,1,0,5,1,1,1,5,1,1 | ENSMUSG00000097074,"ENSMUSG00000097074",0,0,0,0,0,0,0,0,0,0,0,0,0,0,0,0,0,0,0,0 |
| ENSMUSG00000068036,"ENSMUSG00000068036",2621,2915,3023,3797,3411,2937,3083,2409,2986,2900,3505,3056,2630,3119,2823,3255,2816,3564,2271,2898 | ENSMUSG00000031230,"ENSMUSG00000031230",4,12,25,7,16,9,13,8,9,11,6,12,7,6,12,10,8,14,2,10 | ENSMUSG00000110864,"ENSMUSG00000110864",0,0,0,0,0,0,0,0,0,0,0,0,0,0,0,0,0,0,0,0 |
| ENSMUSG00000027893,"ENSMUSG00000027893",2566,2601,3231,3248,3146,2931,2682,2362,2476,2604,3194,2403,2867,2762,2614,2954,2480,3258,2360,2504 | ENSMUSG00000038997,"ENSMUSG00000038997",1,0,0,1,0,1,10,1,2,1,1,0,5,1,0,0,2,1,0,2 | ENSMUSG00000110802,"ENSMUSG00000110802",0,0,0,0,0,0,0,0,0,0,0,0,0,0,0,0,0,0,0,0 |
| ENSMUSG00000031700,"ENSMUSG00000031700",658,784,1106,1200,979,930,815,898,917,948,1175,799,986,958,932,1080,831,1025,698,884 | ENSMUSG00000073607,"ENSMUSG00000073607",0,3,4,2,0,0,0,0,1,0,0,0,0,0,2,0,0,1,3,0 | ENSMUSG00000111682,"ENSMUSG00000111682",0,0,0,0,0,0,0,0,0,0,0,3,0,0,0,0,0,0,0,0 |
| ENSMUSG00000050390,"ENSMUSG00000050390",1619,2279,2655,2828,2494,2249,2636,1670,2615,2701,2470,2291,1968,2397,2014,2818,2041,2852,1809,2131 | ENSMUSG00000109679,"ENSMUSG00000109679",7,8,6,7,6,2,5,13,7,16,12,3,0,4,9,6,5,11,8,7 | ENSMUSG00000111693,"ENSMUSG00000111693",0,0,0,0,0,0,0,0,0,0,0,0,2,0,0,0,0,0,0,0 |
| ENSMUSG00000079523,"ENSMUSG00000079523",480,3628,4927,6537,4424,4020,3677,4823,4241,3949,5528,4159,5608,4464,4608,6583,4007,7360,6595,5407 | ENSMUSG00000098076,"ENSMUSG00000098076",6,8,5,8,6,7,7,2,8,7,7,4,8,11,5,9,2,13,10,1 | ENSMUSG00000111444,"ENSMUSG00000111444",0,0,0,0,0,0,0,0,0,0,0,0,0,0,0,0,0,0,0,0 |
| ENSMUSG00000051339,"ENSMUSG00000051339",699,958,984,1347,983,922,924,629,918,877,1055,976,841,907,761,877,765,941,712,685 | ENSMUSG00000112971,"ENSMUSG00000112971",8,8,9,12,10,15,12,5,7,12,12,13,10,14,8,8,10,14,6,11 | ENSMUSG00000111805,"ENSMUSG00000111805",0,0,0,0,0,0,0,0,0,0,0,0,0,0,0,0,0,0,0,0 |
| ENSMUSG00000054072,"ENSMUSG00000054072",9204,5879,5888,12056,7273,6106,6608,7184,7897,7269,10181,7500,6175,6291,7209,8334,6946,10456,5706,5842 | ENSMUSG00000068141,"ENSMUSG00000068141",4,5,7,7,1,5,5,8,10,10,17,4,6,13,8,6,9,8,9,7 | ENSMUSG00000097623,"ENSMUSG00000097623",0,0,0,0,0,0,0,0,0,0,1,0,0,0,1,0,0,1,0,0 |
| ENSMUSG00000026479,"ENSMUSG00000026479",9524,11730,16424,14087,17049,12261,14192,10594,13141,14646,11470,12912,10574,14714,13884,17471,13248,16894,7763,14023 | ENSMUSG00000087129,"ENSMUSG00000087129",2,3,5,8,3,11,1,0,4,2,1,3,8,2,2,5,2,4,0,0 | ENSMUSG00000088284,"ENSMUSG00000088284",0,0,0,0,0,0,0,0,0,0,0,0,2,0,0,0,0,0,0,0 |
| ENSMUSG00000028163,"ENSMUSG00000028163",6390,7093,7716,7496,7851,7674,8009,5591,6840,8563,6246,5973,5451,8366,7097,8439,7213,7838,5337,6779 | ENSMUSG00000058625,"ENSMUSG00000058625",3,2,4,3,4,11,0,2,7,5,2,4,9,2,3,6,8,4,2,6 | ENSMUSG00000095555,"ENSMUSG00000095555",0,0,0,0,0,0,0,0,0,0,0,0,0,0,0,0,0,0,0,0 |
| ENSMUSG00000024388,"ENSMUSG00000024388",0,0,0,0,0,0,0,1,0,1,1,1,0,0,0,0,0,0,0,0 | ENSMUSG00000085129,"ENSMUSG00000085129",1,1,0,0,0,0,0,2,5,0,4,0,0,0,0,0,0,1,1,1 | ENSMUSG00000110839,"ENSMUSG00000110839",0,0,0,2,0,0,0,0,0,0,0,0,0,0,0,0,0,0,0,0 |
| ENSMUSG00000002565,"ENSMUSG00000002565",583,579,624,953,830,566,627,428,508,642,701,651,461,575,487,627,456,598,434,480 | ENSMUSG00000087795,"ENSMUSG00000087795",9,1,5,7,3,6,6,3,8,3,2,2,4,4,1,1,4,3,3,4 | ENSMUSG00000111697,"ENSMUSG00000111697",0,0,0,0,0,0,0,0,0,0,0,0,0,0,0,0,0,0,0,0 |
| ENSMUSG00000007739,"ENSMUSG00000007739",2794,2571,3035,3084,2791,2785,2912,2120,2593,2917,3041,2422,2393,2700,2501,2793,2496,2819,2001,2403 | ENSMUSG00000107915,"ENSMUSG00000107915",0,5,6,6,3,1,3,1,1,3,8,1,3,1,4,5,3,0,2,0 | ENSMUSG00000099718,"ENSMUSG00000099718",0,0,0,0,0,0,0,0,0,0,0,0,0,0,0,0,0,0,0,0 |
| ENSMUSG00000028339,"ENSMUSG00000028339",566,907,1294,1068,1038,1285,1052,896,852,1262,1106,631,1186,1151,990,1173,1017,1359,792,993 | ENSMUSG00000053395,"ENSMUSG00000053395",0,1,6,6,5,13,2,1,5,2,7,0,7,4,1,5,3,8,5,5 | ENSMUSG00000093074,"ENSMUSG00000093074",0,0,0,0,0,0,0,0,0,0,0,0,0,0,0,0,0,0,0,0 |
| ENSMUSG00000056999,"ENSMUSG00000056999",7756,7265,8737,9553,9373,7745,7797,7543,8218,6859,10409,8497,9509,7470,8101,10219,6774,10813,7115,8325 | ENSMUSG00000092509,"ENSMUSG00000092509",5,6,3,5,3,8,6,3,3,4,2,4,6,5,5,9,2,3,2,5 | ENSMUSG00000101370,"ENSMUSG00000101370",0,0,2,0,0,0,0,0,0,0,0,0,0,0,0,0,0,0,0,0 |
| ENSMUSG00000030007,"ENSMUSG00000030007",1958,2623,3474,3335,3006,2966,3154,2325,2625,2955,3346,2640,2762,2904,2821,2915,2635,3474,2678,2955 | ENSMUSG00000044034,"ENSMUSG00000044034",0,2,1,0,2,0,2,1,5,1,1,1,2,1,0,3,3,2,4,2 | ENSMUSG00000100294,"ENSMUSG00000100294",0,0,0,0,0,0,0,0,0,0,0,0,0,0,0,0,0,0,0,0 |
| ENSMUSG00000012350,"ENSMUSG00000012350",4548,4746,4171,5777,5422,3752,5627,3166,4909,4967,4934,5013,3652,4443,4704,5397,4487,5342,3588,4113 | ENSMUSG00000105044,"ENSMUSG00000105044",0,0,5,0,1,1,0,2,1,3,2,1,0,3,1,0,0,1,0,1 | ENSMUSG00000087167,"ENSMUSG00000087167",0,0,0,0,0,0,0,0,0,0,0,0,0,0,0,0,0,0,0,0 |
| ENSMUSG00000079435,"ENSMUSG00000079435",1150,2750,3853,3373,2737,2872,2768,2270,2658,3290,3345,2696,3148,3170,2847,3535,2604,3851,3464,3125 | ENSMUSG00000105636,"ENSMUSG00000105636",0,3,3,1,9,3,2,6,4,3,3,3,2,3,4,3,1,7,1,4 | ENSMUSG00000102013,"ENSMUSG00000102013",0,0,0,0,0,0,0,0,0,0,0,0,0,0,0,0,0,0,0,0 |
| ENSMUSG00000025085,"ENSMUSG00000025085",1867,2576,3196,3800,3456,2274,2918,3121,3215,2685,3770,3048,3984,3039,3261,3871,2794,4233,3447,3197 | ENSMUSG00000031026,"ENSMUSG00000031026",3,8,1,3,6,6,0,0,1,4,4,4,3,2,7,2,2,3,6,3 | ENSMUSG00000102889,"ENSMUSG00000102889",0,0,0,0,0,0,0,0,0,1,0,0,0,0,0,0,0,0,0,0 |
| ENSMUSG00000028248,"ENSMUSG00000028248",1536,1745,2226,2138,2229,2145,1917,1686,1853,2146,2477,1757,2259,2079,1884,2260,2000,2488,1833,1846 | ENSMUSG00000053519,"ENSMUSG00000053519",0,2,1,0,0,0,1,0,2,0,0,0,2,1,0,1,0,2,0,3 | ENSMUSG00000101584,"ENSMUSG00000101584",0,0,0,0,0,0,0,0,0,0,0,0,0,0,0,0,0,0,0,0 |

| ENSMUSG00000033955,"ENSMUSG00000033955",2703,4983,6138,7049,5567,4863,5918,4299,5258,5492,5871,5636,4611,5808,5578,6574,4988,6652,4901,5155 | ENSMUSG00000107195,"ENSMUSG00000107195",3,3,2,4,10,3,14,4,2,6,4,5,4,2,13,1,2,2,3,2 | ENSMUSG00000086225,"ENSMUSG00000086225",0,0,0,0,0,0,0,0,0,0,0,0,0,0,0,0,0,0,0,0 |
| --- | --- | --- |
| ENSMUSG00000020570,"ENSMUSG00000020570",4503,3180,3839,4151,4031,3612,3625,2673,3526,3980,3691,3026,2875,3647,3286,3449,3506,3534,2597,3062 | ENSMUSG00000112214,"ENSMUSG00000112214",0,0,0,0,0,0,0,0,0,0,0,0,1,0,0,0,0,0,0,0 | ENSMUSG00000101648,"ENSMUSG00000101648",0,0,0,0,0,0,0,0,0,0,0,0,0,0,0,0,0,0,0,0 |
| ENSMUSG00000009863,"ENSMUSG00000009863",1315,1299,1707,1511,1611,1337,1312,1284,1353,1505,1726,1204,1637,1407,1433,1552,1381,1771,1448,1543 | ENSMUSG00000096630,"ENSMUSG00000096630",2,2,0,3,6,3,3,3,1,0,2,0,2,7,8,1,2,6,7,1 | ENSMUSG00000100279,"ENSMUSG00000100279",0,0,0,0,0,0,0,0,0,0,0,0,0,0,0,0,0,3,0,0 |
| ENSMUSG00000028415,"ENSMUSG00000028415",4,11,13,16,4,3,7,4,3,13,9,14,4,7,6,13,2,13,5,3 | ENSMUSG00000115454,"ENSMUSG00000115454",0,0,0,0,0,2,1,0,0,1,0,0,0,1,0,0,0,0,1,0 | ENSMUSG00000100795,"ENSMUSG00000100795",0,0,0,0,0,0,0,0,0,0,0,0,0,0,0,0,0,0,0,0 |
| ENSMUSG00000026853,"ENSMUSG00000026853",589,663,1038,823,906,717,837,783,768,911,908,679,1073,845,798,786,836,984,803,848 | ENSMUSG00000018238,"ENSMUSG00000018238",5,4,4,7,4,10,5,7,7,0,9,8,12,7,3,5,8,2,11,8 | ENSMUSG00000091816,"ENSMUSG00000091816",0,0,0,0,2,0,0,0,0,0,0,0,0,0,0,0,0,0,0,0 |
| ENSMUSG00000026103,"ENSMUSG00000026103",3157,2502,2786,2512,2648,2782,2554,1953,2454,2977,2527,2020,2276,2503,2235,2440,2315,2661,1666,2111 | ENSMUSG00000092097,"ENSMUSG00000092097",12,13,5,8,10,9,16,9,3,5,4,9,11,5,13,5,8,16,3,5 | ENSMUSG00000105162,"ENSMUSG00000105162",0,0,0,0,0,0,0,0,0,0,0,0,0,0,0,0,0,0,0,0 |
| ENSMUSG00000033184,"ENSMUSG00000033184",3519,2664,3001,3021,3420,3094,2594,2258,2569,2922,3009,2420,2491,2615,2464,2828,2479,3261,1899,2543 | ENSMUSG00000086843,"ENSMUSG00000086843",0,0,0,0,0,0,0,0,0,0,0,0,1,1,0,1,1,1,1,3 | ENSMUSG00000104567,"ENSMUSG00000104567",0,0,0,0,0,0,0,0,0,0,0,0,0,0,0,0,0,0,0,0 |
| ENSMUSG00000032035,"ENSMUSG00000032035",1770,2063,2745,2414,2667,2601,2288,2301,2059,2828,2611,1645,2661,2554,2437,2759,2289,3042,2096,2405 | ENSMUSG00000074627,"ENSMUSG00000074627",2,5,3,0,0,3,2,1,2,3,1,2,7,0,2,6,0,5,1,1 | ENSMUSG00000059547,"ENSMUSG00000059547",0,0,0,0,0,0,0,0,0,0,0,0,0,0,0,0,0,0,0,0 |
| ENSMUSG00000020923,"ENSMUSG00000020923",1560,2920,3497,3686,3235,2968,3301,2471,2985,3231,3533,2900,2947,3238,2907,3364,2727,3586,2675,3078 | ENSMUSG00000098489,"ENSMUSG00000098489",21,27,33,30,30,29,24,23,29,29,30,24,26,32,43,34,24,41,5,34 | ENSMUSG00000088296,"ENSMUSG00000088296",0,0,3,0,0,0,0,0,0,0,0,0,0,0,0,0,0,0,0,0 |
| ENSMUSG00000031532,"ENSMUSG00000031532",1412,1577,2114,2223,2025,1829,1784,1641,1663,1932,2021,1610,2138,2014,1931,1952,1737,2253,1622,1889 | ENSMUSG00000081355,"ENSMUSG00000081355",0,0,5,6,4,5,4,3,1,7,2,5,3,3,4,11,5,5,1,1 | ENSMUSG00000085512,"ENSMUSG00000085512",0,0,0,0,0,0,0,0,0,0,0,0,0,0,0,0,0,0,0,0 |
| ENSMUSG00000050440,"ENSMUSG00000050440",5,5,3,10,8,5,14,3,17,3,3,12,0,13,3,6,9,3,8,2 | ENSMUSG00000091890,"ENSMUSG00000091890",0,0,0,0,0,0,0,0,0,0,0,0,0,0,0,0,0,0,0,1 | ENSMUSG00000081691,"ENSMUSG00000081691",0,1,0,0,0,0,0,0,0,0,0,0,0,0,0,0,0,0,0,0 |
| ENSMUSG00000033004,"ENSMUSG00000033004",3821,3782,4427,4602,4437,4437,3751,3544,3635,3878,4772,3371,4320,4106,3847,4362,3948,5063,2998,3529 | ENSMUSG00000074108,"ENSMUSG00000074108",20,33,23,13,30,35,25,31,35,43,18,12,51,23,23,40,25,37,30,42 | ENSMUSG00000080833,"ENSMUSG00000080833",0,0,1,0,0,0,0,0,0,0,1,0,0,0,0,0,0,0,0,0 |
| ENSMUSG00000005981,"ENSMUSG00000005981",1033,1234,1576,1755,1462,1325,1553,1216,1356,1329,1746,1337,1438,1423,1396,1418,1313,1626,1355,1374 | ENSMUSG00000111481,"ENSMUSG00000111481",9,3,1,0,3,2,1,7,1,4,2,2,2,1,7,4,0,0,2,2 | ENSMUSG00000104137,"ENSMUSG00000104137",0,0,0,0,0,0,0,0,0,0,0,0,0,0,0,0,0,0,0,0 |
| ENSMUSG00000000184,"ENSMUSG00000000184",1074,1149,1315,1550,1479,1474,1239,1083,1191,1365,1319,1169,1349,1178,1238,1491,1178,1623,984,1240 | ENSMUSG00000037196,"ENSMUSG00000037196",2,0,7,7,3,3,1,1,2,6,2,3,2,12,2,8,1,6,0,6 | ENSMUSG00000083752,"ENSMUSG00000083752",0,0,0,0,0,0,0,0,0,0,0,0,0,0,0,0,0,0,0,0 |
| ENSMUSG00000021814,"ENSMUSG00000021814",4693,5465,5689,6636,5882,5300,6329,3762,5401,5878,5461,5042,4100,5345,4688,5881,4915,5817,4154,4851 | ENSMUSG00000040138,"ENSMUSG00000040138",19,29,50,18,24,31,27,32,35,18,28,32,30,21,31,31,39,33,33,47 | ENSMUSG00000084259,"ENSMUSG00000084259",0,0,0,0,0,0,0,0,0,0,2,0,0,0,1,0,0,0,0,0 |
| ENSMUSG00000025162,"ENSMUSG00000025162",2662,3455,4354,4474,4118,3706,4109,3238,3658,4034,3915,3275,3520,3980,3585,4081,3447,4619,3497,3674 | ENSMUSG00000044751,"ENSMUSG00000044751",3,6,3,7,3,5,1,5,3,4,6,3,2,12,2,7,5,15,1,3 | ENSMUSG00000098650,"ENSMUSG00000098650",0,0,0,0,0,0,0,0,0,0,0,0,0,0,0,0,0,0,0,0 |
| ENSMUSG00000030002,"ENSMUSG00000030002",3255,2996,3507,3462,3495,3309,3212,2584,3071,3302,3558,2750,2818,3076,2750,3138,2770,3467,2242,2727 | ENSMUSG00000062252,"ENSMUSG00000062252",3,4,5,6,7,8,3,6,7,2,3,6,2,4,6,6,7,17,6,4 | ENSMUSG00000089103,"ENSMUSG00000089103",0,0,0,0,0,0,0,0,0,0,0,0,0,0,0,0,0,0,0,1 |
| ENSMUSG00000020432,"ENSMUSG00000020432",590,1154,1794,1257,1361,1380,1160,1301,1126,1439,1309,815,1760,1450,1184,1572,1244,1572,1153,1433 | ENSMUSG00000056073,"ENSMUSG00000056073",8,5,5,7,2,8,3,4,15,13,10,0,8,3,3,11,7,10,1,12 | ENSMUSG00000103635,"ENSMUSG00000103635",0,0,0,0,0,0,0,0,0,0,0,0,0,1,0,0,0,0,0,0 |
| ENSMUSG00000041408,"ENSMUSG00000041408",2950,2611,2940,3092,3313,2827,2725,2345,2666,3075,2918,2450,2565,2797,2600,3113,2500,3288,2039,2683 | ENSMUSG00000042567,"ENSMUSG00000042567",3,9,8,7,6,11,9,11,8,3,11,14,8,14,10,7,11,12,6,12 | ENSMUSG00000087328,"ENSMUSG00000087328",0,0,0,0,0,0,0,0,0,0,0,0,0,0,0,0,0,0,0,0 |
| ENSMUSG00000029313,"ENSMUSG00000029313",3425,4495,4449,4286,4787,5212,4558,3536,4425,5828,4178,3528,3976,5595,3858,5183,4341,4620,2602,3801 | ENSMUSG00000079186,"ENSMUSG00000079186",15,14,25,21,26,17,19,45,21,17,42,19,43,24,39,54,24,61,20,42 | ENSMUSG00000085476,"ENSMUSG00000085476",0,0,3,0,0,0,0,0,0,0,0,0,0,0,0,0,0,0,0,0 |
| ENSMUSG00000001416,"ENSMUSG00000001416",3277,3523,4293,4518,3941,3788,4066,2896,3450,3839,4324,3327,3373,3813,3655,3770,3324,4168,3092,3539 | ENSMUSG00000086395,"ENSMUSG00000086395",2,5,2,6,2,0,0,1,0,0,1,1,4,1,1,7,3,2,3,4 | ENSMUSG00000086998,"ENSMUSG00000086998",0,0,0,0,0,0,0,1,0,0,0,0,0,0,0,0,0,0,0,0 |
| ENSMUSG00000031613,"ENSMUSG00000031613",371,276,369,308,279,313,226,307,312,269,491,301,384,267,235,330,248,304,337,288 | ENSMUSG00000086837,"ENSMUSG00000086837",12,9,5,5,14,3,7,1,2,3,12,1,5,2,9,4,10,10,2,5 | ENSMUSG00000082749,"ENSMUSG00000082749",0,0,0,0,1,0,0,0,0,0,0,0,0,0,0,2,0,0,0,0 |
| ENSMUSG00000017778,"ENSMUSG00000017778",1324,1623,2150,2055,1563,1431,1448,1449,1846,2163,2353,1797,1862,1881,1644,2070,1737,2143,1695,1979 | ENSMUSG00000116861,"ENSMUSG00000116861",3,6,11,13,5,8,12,8,4,11,18,12,11,10,3,17,8,9,7,15 | ENSMUSG00000064010,"ENSMUSG00000064010",0,0,0,0,1,0,0,1,0,0,0,0,0,0,0,0,0,1,0,0 |
| ENSMUSG00000020790,"ENSMUSG00000020790",4879,3975,5254,5769,4935,4710,4532,3999,4359,4452,4877,3849,4510,4588,4355,5184,4570,5115,3488,4353 | ENSMUSG00000086607,"ENSMUSG00000086607",2,1,6,2,10,8,2,3,2,3,1,1,0,1,5,2,1,1,1,5 | ENSMUSG00000107573,"ENSMUSG00000107573",0,0,0,0,0,1,0,0,0,0,0,0,0,0,0,0,0,0,0,0 |
| ENSMUSG00000034484,"ENSMUSG00000034484",7454,5820,9005,8326,8158,8222,6293,6177,6144,7869,7638,5183,7039,7634,6982,7087,7285,7043,5638,6563 | ENSMUSG00000086862,"ENSMUSG00000086862",0,11,4,5,11,13,8,8,1,12,3,4,10,3,9,12,8,8,10,12 | ENSMUSG00000081791,"ENSMUSG00000081791",0,0,0,0,0,0,0,0,0,0,0,0,0,0,0,0,0,0,0,0 |
| ENSMUSG00000019302,"ENSMUSG00000019302",4052,4481,5066,4860,5411,5182,5181,3535,4243,5907,4622,3790,4398,5487,4280,5094,4331,5086,3482,3787 | ENSMUSG00000094613,"ENSMUSG00000094613",0,0,0,0,0,0,0,0,0,1,0,0,0,0,0,0,1,0,0,0 | ENSMUSG00000080918,"ENSMUSG00000080918",0,0,0,0,0,0,0,0,0,0,0,0,0,0,0,0,0,0,0,0 |
| ENSMUSG00000022419,"ENSMUSG00000022419",1493,1362,1423,1563,1586,1405,1641,838,1306,1488,1344,1196,953,1290,1127,1181,1180,1390,779,1039 | ENSMUSG00000031727,"ENSMUSG00000031727",0,1,1,0,0,0,0,0,0,0,0,0,3,0,0,0,0,0,2,1 | ENSMUSG00000093006,"ENSMUSG00000093006",0,0,0,0,0,1,0,0,0,0,0,0,0,0,1,0,0,0,0,0 |
| ENSMUSG00000054619,"ENSMUSG00000054619",240,277,435,279,315,255,269,280,274,258,416,286,477,321,280,394,317,387,360,365 | ENSMUSG00000065669,"ENSMUSG00000065669",5,10,9,7,4,4,5,12,6,5,8,9,9,8,2,11,7,6,3,2 | ENSMUSG00000081107,"ENSMUSG00000081107",0,0,0,0,0,0,0,0,0,0,0,0,0,0,0,0,0,0,0,0 |
| ENSMUSG00000001211,"ENSMUSG00000001211",842,1276,1800,1678,1530,1405,1493,1382,1497,1436,1956,1327,1852,1491,1524,1567,1443,1740,1526,1594 | ENSMUSG00000039315,"ENSMUSG00000039315",1,3,2,5,10,9,7,15,4,3,14,2,15,11,10,3,7,9,8,4 | ENSMUSG00000081749,"ENSMUSG00000081749",0,0,0,0,0,0,0,0,0,0,0,0,0,0,0,0,0,0,0,0 |
| ENSMUSG00000038023,"ENSMUSG00000038023",1887,1488,1987,2133,1989,1771,2001,1608,1764,1779,2119,1738,1964,1852,1806,1977,1730,2125,1713,1793 | ENSMUSG00000056771,"ENSMUSG00000056771",1,5,3,3,2,0,5,3,0,1,9,3,0,3,4,6,5,5,2,1 | ENSMUSG00000083234,"ENSMUSG00000083234",0,0,0,0,0,0,1,0,0,0,0,0,0,1,0,0,0,0,0,0 |
| ENSMUSG00000000171,"ENSMUSG00000000171",1202,1599,2008,1863,1786,1568,1609,1523,1565,1767,2093,1578,1868,1721,1616,1945,1612,1994,1909,1665 | ENSMUSG00000022039,"ENSMUSG00000022039",0,1,0,0,0,2,0,1,0,2,2,0,2,3,0,3,2,0,0,0 | ENSMUSG00000020548,"ENSMUSG00000020548",0,0,0,0,0,0,0,0,0,0,0,0,0,0,0,0,0,0,0,0 |
| ENSMUSG00000029860,"ENSMUSG00000029860",3040,5123,6180,5296,5484,6486,5360,4885,5260,7297,5450,3951,5601,6834,5017,6281,5581,6602,3788,5612 | ENSMUSG00000111686,"ENSMUSG00000111686",9,14,10,17,9,9,7,8,8,10,12,10,24,20,14,17,20,13,5,18 | ENSMUSG00000064899,"ENSMUSG00000064899",0,0,0,0,0,0,0,0,0,0,0,0,0,0,0,0,0,0,0,0 |
| ENSMUSG00000032966,"ENSMUSG00000032966",2032,2491,3340,2905,2928,2908,2688,2252,2604,3234,3106,2237,2855,2871,2534,2742,2581,2860,2213,2740 | ENSMUSG00000097482,"ENSMUSG00000097482",5,2,6,3,5,3,6,6,7,1,2,7,3,3,4,8,1,2,3,2 | ENSMUSG00000081515,"ENSMUSG00000081515",0,0,0,0,0,0,0,0,0,0,0,0,0,0,0,0,0,0,2,0 |
| ENSMUSG00000001891,"ENSMUSG00000001891",2140,1570,1989,1995,2286,1695,1648,1596,1738,1897,2122,1756,1929,1663,1686,1842,1703,2027,1476,1815 | ENSMUSG00000084131,"ENSMUSG00000084131",1,7,2,6,2,0,5,4,4,4,6,3,8,4,1,3,3,7,6,3 | ENSMUSG00000065411,"ENSMUSG00000065411",0,0,0,0,0,0,0,0,0,0,0,0,0,0,0,0,0,0,0,0 |
| ENSMUSG00000003623,"ENSMUSG00000003623",640,464,697,571,663,543,470,554,539,561,721,424,739,478,493,661,535,696,503,630 | ENSMUSG00000091989,"ENSMUSG00000091989",2,1,2,2,2,2,1,2,2,2,4,5,0,4,1,3,3,5,1,3 | ENSMUSG00000109833,"ENSMUSG00000109833",0,0,0,0,0,0,0,0,2,0,0,0,0,0,0,0,0,1,0,0 |
| ENSMUSG00000029482,"ENSMUSG00000029482",583,892,985,1454,1131,978,1096,834,1013,912,1199,1182,1052,1097,992,1013,914,1126,1168,1000 | ENSMUSG00000109957,"ENSMUSG00000109957",4,0,0,0,0,0,0,0,0,1,1,0,0,0,0,1,0,0,0,0 | ENSMUSG00000063881,"ENSMUSG00000063881",0,0,0,0,0,1,0,0,0,0,0,0,0,0,0,0,0,0,0,0 |
| ENSMUSG00000039197,"ENSMUSG00000039197",628,521,652,565,572,582,568,485,568,591,606,467,685,551,497,521,501,599,476,564 | ENSMUSG00000085078,"ENSMUSG00000085078",0,1,4,0,0,0,0,0,0,2,0,3,2,1,0,0,3,3,4,3 | ENSMUSG00000081932,"ENSMUSG00000081932",0,0,0,0,0,0,0,0,0,0,0,1,0,0,0,0,1,0,0,0 |
| ENSMUSG00000003528,"ENSMUSG00000003528",416,861,1129,1116,1015,1028,868,762,864,894,1031,920,1019,949,820,986,815,1116,852,986 | ENSMUSG00000115667,"ENSMUSG00000115667",7,15,11,13,16,9,19,18,23,29,17,9,20,16,20,9,22,16,8,12 | ENSMUSG00000088879,"ENSMUSG00000088879",0,0,0,0,0,0,0,0,0,0,0,0,0,0,0,0,0,0,0,0 |
| ENSMUSG00000034075,"ENSMUSG00000034075",3353,4307,4472,5133,4909,4086,5096,3513,4652,4604,4611,4436,4052,4474,4295,4833,4241,4998,3316,4230 | ENSMUSG00000117187,"ENSMUSG00000117187",2,1,3,1,5,0,2,1,2,3,4,1,3,4,5,4,1,5,3,0 | ENSMUSG00000081342,"ENSMUSG00000081342",0,0,0,0,0,1,0,0,0,0,0,0,0,0,0,0,0,0,2,0 |
| ENSMUSG00000007817,"ENSMUSG00000007817",1570,2759,3349,3264,3534,2987,2745,2842,3004,3060,3226,2549,3621,3148,2844,3603,3062,4166,2469,3099 | ENSMUSG00000086935,"ENSMUSG00000086935",6,10,8,13,13,17,14,22,13,14,29,5,16,14,9,24,8,17,8,19 | ENSMUSG00000081304,"ENSMUSG00000081304",0,0,0,0,0,0,0,0,0,0,0,0,0,0,0,0,0,0,0,0 |
| ENSMUSG00000029417,"ENSMUSG00000029417",11663,10418,9863,17112,14605,12788,12696,14271,14952,12159,13444,14148,11489,12093,13079,20633,12479,21290,12307,14540 | ENSMUSG00000079554,"ENSMUSG00000079554",5,11,9,14,9,13,8,5,11,13,16,4,14,7,9,9,10,7,8,6 | ENSMUSG00000088798,"ENSMUSG00000088798",0,0,0,0,0,0,0,0,0,0,0,0,0,0,0,0,0,0,0,0 |
| ENSMUSG00000040488,"ENSMUSG00000040488",1320,1600,1794,2091,1914,1722,1918,1279,1580,2248,1988,1677,1827,1760,1649,1684,1933,2396,1383,1706 | ENSMUSG00000082345,"ENSMUSG00000082345",9,14,1,13,12,7,10,6,10,17,12,6,3,14,6,2,16,8,7,8 | ENSMUSG00000082945,"ENSMUSG00000082945",0,0,0,0,0,0,0,0,0,0,0,0,0,0,0,0,0,0,0,0 |
| ENSMUSG00000029169,"ENSMUSG00000029169",3667,3439,3667,4118,3973,3659,3836,2603,3334,3979,4003,3291,3032,3511,3206,3506,3260,3663,2709,3033 | ENSMUSG00000031326,"ENSMUSG00000031326",5,2,0,0,0,0,0,0,0,3,0,0,0,3,0,3,1,1,1,0 | ENSMUSG00000078258,"ENSMUSG00000078258",0,0,0,0,0,0,0,0,0,0,0,0,0,0,0,3,0,0,0,0 |
[truncated: 4,721,840 more chars]
